# Comparative Proteomic Analysis of Buffalo Oocytes Matured *in vitro* Using iTRAQ Technique

**DOI:** 10.1038/srep31795

**Published:** 2016-08-26

**Authors:** Lingsheng Chen, Linhui Zhai, Chunfeng Qu, Chengpu Zhang, Sheng Li, Feilin Wu, Yingzi Qi, Fenghua Lu, Ping Xu, Xiangping Li, Deshun Shi

**Affiliations:** 1 State Key Laboratory for Conservation and Utilization of Subtropical Agro-Bioresources, Guangxi University, Nanning 530005, China; 2 State Key Laboratory of Proteomics, Beijing Proteome Research Center, National Engineering Research Center for Protein Drugs, National Center for Protein Sciences, Beijing Institute of Radiation Medicine, Beijing 102206, China; 3 Chemical Proteomics Center & State Key Laboratory of Drug Research, Shanghai Institute of Materia Medical, Chinese Academy of Sciences, Shanghai, 201203, China; 4 Department of reproductive medicine, Hechi People’s hospital of Guangxi, Hechi 547000, China; 5 Key Laboratory of Combinatorial Biosynthesis and Drug Discovery (Wuhan University), Ministry of Education, and Wuhan University School of Pharmaceutical Sciences, Wuhan 430071, China

## Abstract

To investigate the protein profiling of buffalo oocytes at the germinal vesicle (GV) stage and metaphase II (MII) stage, an iTRAQ-based strategy was applied. A total of 3,763 proteins were identified, which representing the largest buffalo oocytes proteome dataset to date. Among these proteins identified, 173 proteins were differentially expressed in GV oocytes and competent MII oocytes, and 146 proteins were differentially abundant in competent and incompetent matured oocytes. Functional and KEGG pathway analysis revealed that the up-regulated proteins in competent MII oocytes were related to chromosome segregation, microtubule-based process, protein transport, oxidation reduction, ribosome, and oxidative phosphorylation, etc., in comparison with GV and incompetent MII oocytes. This is the first proteomic report on buffalo oocytes from different maturation stages and developmental competent status. These data will provide valuable information for understanding the molecular mechanism underlying buffalo oocyte maturation, and these proteins may potentially act as markers to predict developmental competence of buffalo oocyte during *in vitro* maturation.

*In vitro* maturation (IVM) of mammalian oocytes is an important technique for animal embryo technology, which directly affects the efficiencies of *in vitro* fertilization (IVF), nuclear transfer and transgenic etc. The developmental competence of oocytes matured *in vitro* is generally lower than those matured *in vivo*^1^,^2^, which are caused by the desynchronization of nuclear and cytoplasmic maturation^3^, and insufficient cytoplasmic maturation^4^. Although lots of efforts have been made to investigate factors affecting oocyte maturation in mouse and other animals, the molecular mechanism related to the oocyte maturation of important livestock is seldom reported. To improve the quality of oocytes matured *in vitro*, we must understand the molecular mechanism of oocytes during maturation, determine key genes and proteins associated with oocyte maturation, and then find a reliable molecular marker to predict developmental competence of oocytes matured *in vitro*.

During mammalian oogenesis, the oocyte undergoes a protracted period arrest at dictyate or germinal vesicle (GV) stage, subsequently undergoes germinal vesicle breakdown (GVBD) and proceed to the metaphase I (MI) stage, followed by extrusion of the first polar body and arrest at metaphase II (MII) stage (Fig. 1A). Only developmental competent MII oocyte enable reprogram sperm DNA and initiate subsequent embryo development. In order to investigate the gene expression profile of oocytes during maturation process and identify differential expression genes/transcripts in oocyte at different developmental stages and with different competence, various approaches, such as genomics and transcriptomics have been performed in the last decade. Although transcriptomics-based strategy had been employed in oocytes or embryos of some species, it was found that there was no strict linear correlation between mRNA and protein expression level^5^, especially in oocytes^6^, since a large number of stored maternal mRNAs existed in oocytes was not polyadenylated, and cannot be translated into proteins^7^. More importantly, most of proteins acquire their functions following modification after translation, such as phosphorylation, ubiquitination and sumoylation, etc. Therefore, proteomic approaches are indispensable to elucidate the molecular process in regulating oocyte maturation.

Proteomics have been applied in the research of mammalian oocytes and embryos, including mouse^8^,^9^,^10^,^11^,^12^, bovine^13^,^14^,^15^, pig^6^,^16^,^17^,^18^, and numbers of proteomics data have been obtained. The related studies were mainly focused on identifying protein expression profile of embryos at different developmental stages, and maternal proteins in oocytes. In addition, substantial studies have been performed to reveal signal transduction pathways during oocyte maturation and important transcription factors related to reprogramming and chromosome reconstruction in oocytes. Although numerous valuable protein information about the growth and development of mammalian oocyte/embryo was obtained from proteomics data, there was still some problems to be solved. First, many proteins, especially low molecular weight and low abundance proteins is difficult to be identified due to the limitation of 2-DE platform used in those studies, which result in limited proteome coverage. Second, the classic proteomics quantification methods, including 2-DE, or label free method are unfit for accurate quantification between samples, especially those proteins with small fold change ratios in different groups.

Isobaric tags for relative and absolute quantitation (iTRAQ) is a widely used stable isotope-based approach for quantitative proteomics, which allows simultaneous identification and quantification. The same peptide from different samples display a single peak in MS scans, thus reducing the complexity of parent ion spectra. And quantification is performed via reporter ion intensity from the low mass range at MS/MS level, which improves the accuracy of quantitation. In addition, this method could simultaneously analyze up to eight different samples in one experiment.

Buffalo (*Bubalus bubalis*) is an important domestic animal distributed in the tropical and subtropical region, providing high quality of milk, meat, and work power^19^. However, there were very few reports on protein dynamic changes during oocyte maturation using proteomics techniques in buffalos due to their special distribution region. The efficiency of buffalo blastocysts produced *in vitro* is reported to be low in comparison with bovine blastocysts produced *in vitro*. Thus, the present study was undertaken to investigate the protein expression profile of buffalo oocytes during IVM, identify the differentially proteins in oocytes at GV and MII stages with different competence using iTRAQ quantitative proteomics technology. This research will provide useful information for understanding the changes in protein profiling of buffalo oocyte during maturation, and then set up a foundations for further exploring the molecular mechanism of buffalo oocytes matured *in vitro*.

## Results

### Quantitative proteomics analysis of buffalo oocytes

**Figure 1:**
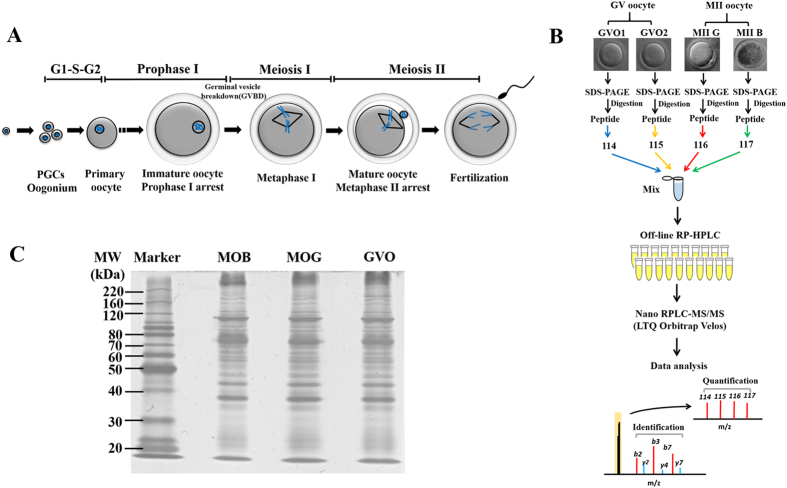
Overview of mammalian oocyte maturation process and experimental workflow. **(A**) Schematic representation of maturation in mammalian oocyte before fertilization. (**B**) Flow chart for the establishing of buffalo oocyte proteome. Samples from germinal vesicle (GV), competent and incompetent metaphase II (MII) buffalo oocytes were collected in two biological replicates. A similar amounts of proteins were digested into peptides using trypsin. The resulting peptides were subsequently extracted and desalted. All samples were pooled together after iTRAQ labeling and separated by RP-HPLC, and then analyzed using LC-MS/MS. Tag 113 and 114 for GV oocytes, 115 and 116 for competent and incompetent MII oocytes, respectively. (**C**) SDS-PAGE of buffalo oocytes proteins. Buffalo oocytes proteins were separated using a 10%SDS-PAGE gel and then stained with Coomassie brilliant blue stain.

**Figure 2:**
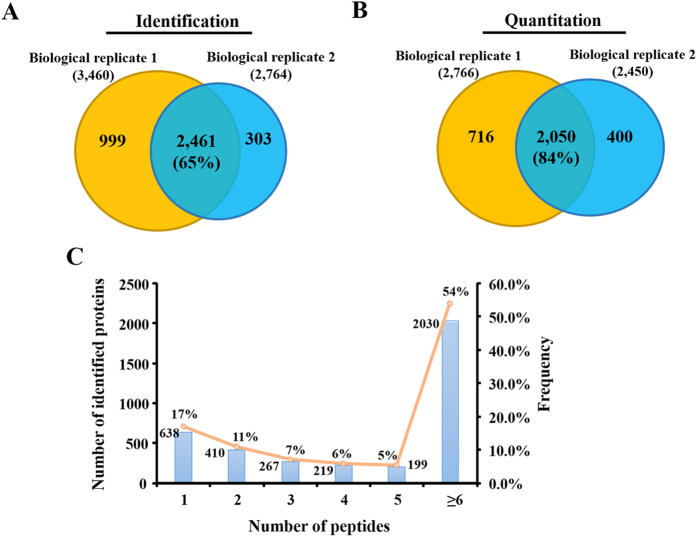
Overview of identified and quantified proteins. Veen diagram representing the overlap of identified (**A**) and quantified (**B**) proteins in two biological replicates, respectively. The number in brackets indicates the percentage of common identified proteins. (**C**) Histogram displaying the number of peptides matched to proteins. The x-axis illustrates the number of identified peptides. The primary y-axis indicates the number of identified proteins (bars). The second y-axis represents the percent (lines).

To identify the differentially expressed proteins in buffalo oocytes before and after maturation, GV and MII stage oocytes were selected for quantitative proteomics analysis by iTRAQ. The experimental workflow was depicted in Fig. 1B. After separated by SDS-PAGE (Fig. 1C) and in-gel digested with trypsin, the peptides were labeled with iTRAQ regents. Labeled peptides were then pooled and separated into 20 fractions by high-pH reverse-phase high performance liquid chromatography, followed by nano-UPLC-MS/MS analysis with LTQ-Orbitrap Velos mass spectrometer. Two biological experiments were carried out and the LC-MS/MS identification was repeated twice for each biological replicate. The representative identification results of mass spectrometer were showed in Supplementary Information Figure S1. A total of 3,763 proteins (FDR < 1%) were identified from the labled samples, among which 2,461 proteins were found in both biological replicates (65% of the proteome) (Fig. 2A). Among the identified proteins, 3,166 (84%) proteins were quantified, of which 2,050 proteins were found to co-exist in two biological replicates (Fig. 2B). The complete list of all peptide and protein identifications of buffalo oocytes was showed in Supplementary Information Table S1. Among the identified proteins, 17%, 11%, 7%, 6%, 5% and 54% of proteins comprised of 1-peptide, 2-peptides, 3-peptides, 4-peptides, 5-peptides and at least 6 unique peptides respectively (Fig. 2C).

### Comparison of mammalian oocyte/embryo proteomics datasets

**Figure 3:**
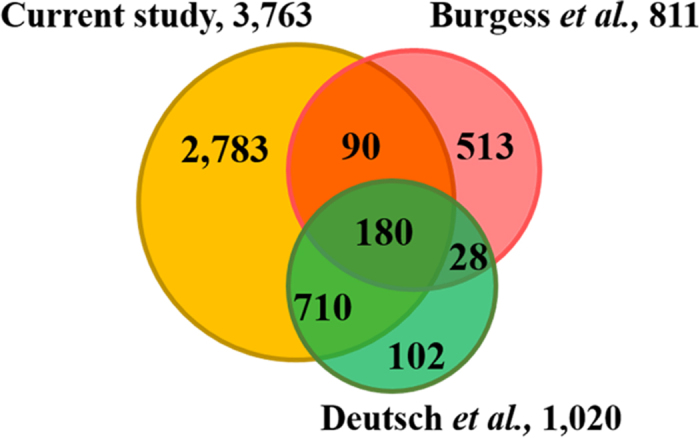
Comparisons of different mammalian oocyte/embryo proteome datasets. Veen diagram showing the overlap between proteins identified in the present study and two published bovine oocyte/embryo proteomic datasets.

To find out the similarity and diversity of expression proteins in oocytes**/**embryos from different species, the iTRAQ quantitative results were compared with published bovine proteome datasets, including bovine GV stage oocytes, cumulus cells (Burgess *et al.*) and embryos (Deutsch *et al.*). Proteome differences in the bovine and buffalo oocytes were showed as Venn diagram of absolute protein numbers (Fig. 3). Seven hundred and ten proteins were communal found in Deutsch’s and our dataset, only 90 proteins were common identified in Burgess’s and ours. A very few proteins (28) were existed in Deutsch and Burgess’s results. We speculated that the differences may be caused by the samples of which oocytes and cumulus cells were used in Deutsch, while MII oocytes and embryos were used in Burgess. It is well known that cumulus cells express many specific proteins to support oocyte growth and maturation, while oocytes do not. The development of bovine embryos before genomic activation at the 8-cell stage is dependent on the maternal proteins stored in oocytes during growing and maturation. Thus, oocytes and early embryos may have similar protein expression patterns.

### Gene Ontology (GO) categorization analysis of buffalo oocyte proteins

To understand the biological functions of identified proteins in the buffalo oocytes, GO categorization analysis was performed using DAVID Bioinformatics Resources. Of the 3,763 identified proteins in buffalo oocytes, 3,184 proteins were annotated to DAVID GO term, and then 277 categorization groups were obtained. According to the GO analysis results, the proteins related to the biological process of generating metabolite precursor, energy metabolism, translation, oxidation reduction and structure were significant enriched in buffalo oocytes (Supplementary Information Figure S2 and Table S2).

### Statistical analysis of mass spectrometry data

**Figure 4:**
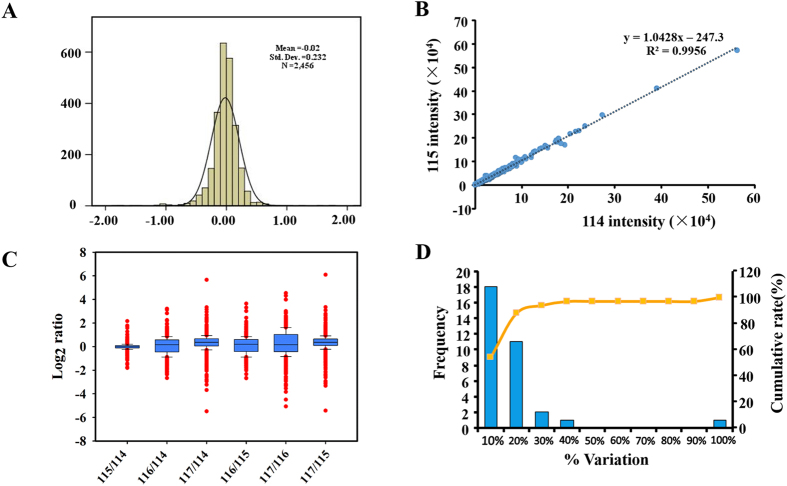
Evaluation of iTRAQ quantification proteomic experiments and determination of the cutoff value for differentially expressed proteins. (**A**) Histogram distribution of quantified proteins log-transformed ratio between technical replicates fits a normal distribution with a standard deviation of 0.16. (**B**) Scatter plots of the two technical replicates, iTRAQ 116,117-labeled cytoplasm abnormal MII oocytes. (**C**) Box-plots analysis of ratios of peptides tagged with 4 tags from 4-plex kit and mixed with an equal amount of non-labeled peptides. Ratios were calculated relative to 114, 115 and 116 iTRAQ tag, respectively. (**D**) The percent variations for the common quantified proteins from two technical replicates. The primary vertical axis represents the number of proteins (bars), and the horizontal axis defines % variation. The secondary vertical axis represents cumulative % of the counted proteins (line).

The distribution of log_2_ ratio between two technical replicates of samples in one biological experiment was normal distribution with standard deviation 0.16 (Fig. 4A), indicating that the large majority of identified proteins were unchanged and the quantitative accuracy of the experiment was high. To evaluate the quantification reproducibility of the iTRAQ experiment, a linear regression analysis of proteins in two replicates was performed. The slope of the linear regression fit to the technical replicates of samples was 1.0428. iTRAQ reporter ion intensities between the two technical replicates of samples showed high correlation (Pearson R^2^ = 0.9956, Fig. 4B), which demonstrated a good reproducibility. In current study, equivalent amount of peptides of four samples were used and mixed in an equal ratio. We performed a comparison of log-transformed ratios from as a box-plot analysis, and the result derived from one biological replicate was showed in Fig. 4C. The ratios were calculated from random two tags. Ideally we observed the ratios of each group matched as expected values, indicating samples were mixed in equal amount. Next, the technical variations and determined the threshold for differentially expressed proteins were evaluated. Accordingly, around 90% of the common identified proteins fell within 30% of the variation in the LC-MS/MS identification replicates (Fig. 4D). Thus, the cutoff point for differentially expressed protein in our study was considered as fold change of ≥2 or ≤ 0.50. Furthermore, iTRAQ ratios also required the P value less than 0.05 (95% confidence limit of proteins considered to change). A minimum of one unique peptide was required to identify and relatively quantify a protein.

**Figure 5:**
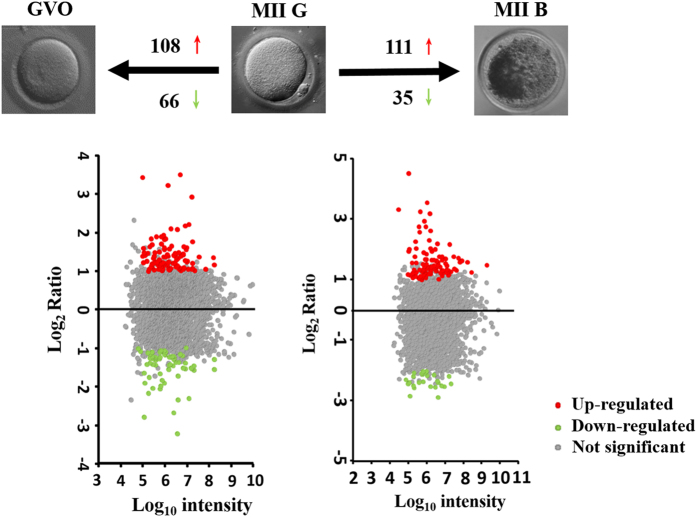
Scatterplot of log_2_ transformed iTRAQ ratio data (left for MII G vs GVO and right for MII G vs MII B). The x-axis shows the log_10_ of the protein intensity. The y-axis shows the log_2_ ratios between MII G and GVO or MII G and MII B, respectively. The red and green dots represent up-regulated and down-regulated proteins, respectively. And the gray dots were those not significant proteins.

Based on the screening criteria, a total of 173 significant differentially expressed proteins were found in competent MII oocytes (MII G) compared to GV stage oocyte (GVO). Among these differentially expressed proteins, 108 and 65 proteins were up-regulated and down-regulated respectively. When MII G were compared with incompetent MII oocytes (MII B), 146 differentially expressed proteins (111 up- and 35 down-regulated) were found to be mapped the cut-off criteria (Table 1, Fig. 5). The complete list of differentially expressed proteins was shown in Supplementary Information Table S3.

### Hierarchical clustering analysis of differentially expressed proteins

**Figure 6:**
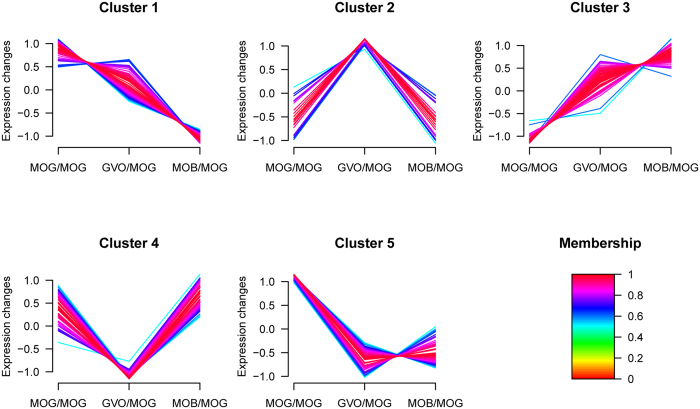
Hierarchical cluster analysis of differentially expressed proteins. Proteins clustered were those significantly differentially expressed at least in one of the two pairwise comparisons. A total of 265 proteins were clustered into five distinct groups.

To understand the dynamic changes of proteins expressed differentially during buffalo oocyte maturation, hierarchical cluster was performed. Proteins clustered were those differentially expressed at least in one of the two pairwise comparisons. As showed in Fig. 6, a total of 265 proteins were classified into five different expression clusters. Then, each cluster proteins were further subjected to gene ontology (GO) annotations using DAVID software. Cluster 1 contained 73 proteins enriched for biological process related to electron transport chain, oxidation reduction, protein transport, oxidative phosphorylation etc. Cluster 2 included 21 proteins that were involved in angiogenesis and blood vessel morphogenesis. Cluster 3 (57 proteins) was related with the heterocycle biosynthetic process, pigment biosynthetic process, macromolecular complex assembly etc. Proteins (54) of Cluster 4 were related to the microtubule-based process, nuclear division, mitosis, chromosome segregation etc. Enrichment of cluster 5 (60 proteins) were proteins involved in oxidation reduction, transmembrane transport, protein location etc. Details for GO annotation of differentially expressed proteins in five clusters were listed in Supplementary Information Table S4.

### Analysis of KEGG pathway related to proteins expressed differentially

**Figure 7:**
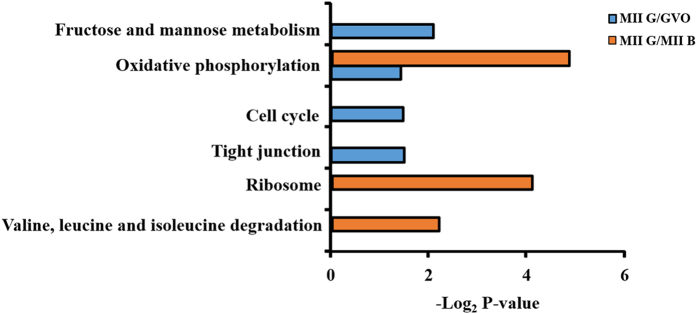
DAVID analysis for KEGG pathway.

To further reveal the signaling pathways related to the maturation of buffalo oocytes, proteins related KEGG pathway were analyzed. As shown in Fig. 7, 173 proteins expressed differentially in MII G and GVO were found to be related to the metabolism of fructose and mannose, oxidative phosphorylation, cell cycle and tight junction. One hundred and forty-six proteins expressed differentially in MII G and MII B were involved in oxidative phosphorylation, ribosome and valine, leucine and isoleucine degradation. Thus, the oxidative phosphorylation was the common pathway, suggesting that high expression proteins related to oxidative phosphorylation pathway may play an important role during *in vitro* maturation of buffalo oocytes.

### Analysis of gene expression by quantitative RT-PCR

To further demonstrate the proteins expressed differentially in buffalo oocytes during *in vitro* maturation, quantitative RT-PCR was performed to check expression of five genes (KIF20A, KIF2C, MYH10, MYH9, and DYNLL2). As shown in Supplementary Information Figure S3, the relative expression patterns of five genes in GVO and MII G oocytes were not in accordance with the results of proteomics analysis, suggesting that post-transcriptional mechanism may involve in the regulation of the expression of these proteins.

## Discussion

*In vitro* maturation of oocytes is an important technology for providing matured oocytes that are utilized in IVF, *in vitro* production (IVP), and somatic cell nuclear transfer. In the past decade, although many efforts have been made to improve the efficiency of the IVM, the development competence of oocytes matured *in vitro* is lower than oocytes matured *in vivo*^1^,^2^. Elucidation of the molecular mechanisms regulating the oocyte maturation and identification of potential predictors related to oocyte developmental competence will help us to improve the quality of oocytes matured *in vitro*. Proteomic approaches allow us to monitor dynamic changes at protein expression level and identify proteins that are functionally associated with a special cell or tissue phenotype. Knowledge of protein expression profiles occurred during the process of oocyte maturation will provide new insights into the molecular mechanisms regulating oocyte maturation.

In the present study, we applied the iTRAQ-based quantitative proteomic strategy to study the protein expression profile of buffalo oocytes during *in vitro* maturation and a total of 3,763 proteins were identified, which represented the largest buffalo oocyte proteome dataset so far. The identified proteins were known to be essential for oogenesis and embryo development, which previously detected in other species, such as NLRP5/MATER, OOEP/FLOPED, PADI6, PRDX1, GDF9, NPM2, TLE6. Novel proteins were also identified in buffalo oocytes, such as ZAR1, BMP15, DNMT1, and PTPN1 (undetected in ref. 15). However, some oocyte-specific proteins (STELLA, SMARCA4, DPPA3, and PMS2) reported in bovine^13^,^14^,^15^ were not detected in current study. A total of 1,264 proteins (34%) were annotated as “uncharacterized proteins” in our report.

As shown in Figure S2, more than half of significantly enriched categories in buffalo oocytes were related to metabolism pathway, including glycolysis, oxidative phosphorylation, tricarboxylic acid cycle (TCA cycle), fatty acid metabolic, lipid biosynthetic and steroid metabolic. These results indicated that oocytes may require different metabolites (such as amino acids, purines and fatty acids) to support their growth and maturation. During the maturation process, oocytes synthesized and stored a large amounts of mRNA and proteins in the cytoplasm, and this materials were utilized later in embryo development until the embryonic genome was activated^20^. Although transcription decreased in oocytes after GVBD, polyadenlyated RNA synthesis was still observed in fully grown mouse oocyte^21^,^22^. In this study, a large number of proteins was involved in mRNA processing, indicating that these proteins should be essential to maintain growth and genome activation for buffalo oocyte. In addition, large numbers of proteins were involved in cell cycle, which were associated with the growth and meiotic maturation occurring during maturation. These results indicate that the cell cycle progression in buffalo oocytes may be driven by the regulation of protein expression.

The important purpose of this work was to investigate the protein expression difference in buffalo oocytes with different maturity or quality. A total of 173 proteins differentially expressed was identified in immature and mature oocytes, and 146 proteins differentially expressed were identified between competent and incompetent matured oocytes. Among these differentially expressed proteins, proteins involved in the oxidative phosphorylation pathway and enriched in mitochondria were up-regulated in MII G compared to GVO and MII B oocytes. Oxidative phosphorylation (OXPHOS), one main pathway occurred in mitochondrial, is the important physiology process to generate ATP^23^. The increase of transcripts/proteins related to OXPHOS pathway would result in the high ATP synthesis. During oocyte maturation, mitochondria could produce ATP mainly through OXPHOS pathway which was used for spindle organization, chromosomal segregation, organelle redistribution, protein transport and other cellular processes^24^,^25^, which was essential for oocyte maturation^26^. The ATP content in morphologically normal or *in vivo*-derived oocytes was significantly higher than that of poor or matured *in vitro* oocytes^27^,^28^. Moreover, oocytes with high ATP content could result in higher morulae and blastocyst development^27^,^29^. In addition, the transcripts and encoded proteins associated with OXPHOS were down-regulated during oocyte maturation, which may reflect the decrease of energy production and utilization in MII oocytes^30^. Thus, the level of OXPHOS may be related to the oocyte quality and the mitochondria activity may have certain roles in regulating the IVM of buffalo oocytes.

In the present study, a large group of proteins associated with protein transport and transmembrane transport were also found to be up-regulated in MII G oocytes compared to GVO and MII B oocytes, indicating that substrates transport were necessary for the buffalo oocyte meiotic maturation. RAB family proteins included RAB2A, RAB3A and RAB21, which were associated with signal transduction, intracellular vesicular transport^31^. RAB3A has been detected in mouse oocytes during meiotic maturation, which was implicated in the regulation of cortical granules migration, polarity establishment and asymmetric division^32^. SEC61α1 and SEC61α2 are the two subgroups of SEC61α, which are localized in ER and ER-Golgi intermediate compartment. SEC61α with other two subunits, SEC61β and SEC61γ, comprise the SEC61 complex, and have function in proteins translocation across endoplasmic Reticulum (ER) membrane^33^. In addition, the members of solute-carrier (SLC) superfamily, SLC12A6, SLC35B2 and SLC25A15, are membrane-bound transporters, which play essential roles in transporting variety of substrates (such as amino acids, glucose, sugar, inorganic cations and anions) across the membranes of cell^34^.

Three major classes of molecular motor are kinesins, dyneins and myosin^35^. They are required for a series of cellular events, including chromosome segregation, spindle assembly, migration and anchoring, cytoplasmic organelles redistribution, mRNA position and cortical reorganization^36^. In the present study, several molecular motor proteins (KIF20A, KIF2C, MYH10, MYH9 and DYNLL2) were found to be up-regulated in MII oocytes compared with GV oocytes, suggesting that they may have potential important roles in the maturation of buffalo oocytes. For example, KIF2C (a member of kinesin-13 family) is an ATP-dependent microtubule depolymerase and involved in resolution of incorrect microtubule attachments in mitosis^37^. Studies in mouse oocytes showed that knockdown of KIF2C led to a delay in chromosome congression and meiosis I arrest, but did not prevent bipolar spindle assembly^37^. Similarly, KIF20A (Kinesin-6 family member, also named as MKlp2) was found to be involved in the cytokinesis^38^. Moreover, KIF20A was proved to be localized at oocyte microtubules and involved in polar body extrusion during mouse oocyte maturation^39^. Inhibition of KIA20A in porcine oocyte led to failure of polar body extrusion, but did not affect spindle morphology^38^. The members of myosin superfamily, MYH10 and MYH9 were also found to be involved in cell migration, adhesion, movement of vesicles and cytokinesis^40^. Recently, Simerly^41^ found that MYH10 and MYH9 were crucial factors for meiotic maturation, fertilization and mitosis in mouse oocytes and embryos. Inactivation of MYH10 or MYH9 led to mouse embryonic lethality^42^. DYNLL1 is one of two cytoplasmic dyneins, which engages in various cellular processes, such as mitosis^43^, chromosome segregation^44^, mRNA position^45^,^46^ and vesicles transport^43^,^47^. Racedo^48^ revealed that the higher mRNA expression of DYNLL1 was related to the developmental competence of bovine oocytes. Yao^49^ revealed that dynein light chain was a regulatory gene related to follicular development and developmental competence of bovine oocytes. Therefore, all of these motor proteins may have crucial roles in maintaining proper nuclear and cytoplasmic maturation of oocytes.

Ribosome is ribonucleoprotein complexes comprising RNA and protein, whose major function is responsible for protein synthesis. The protein synthesis is essential for oocyte meiotic maturation and subsequently embryo development^50^. The high development capacity of oocytes is related to their high rates of protein synthesis^51^. A previously study indicated that differentially expressed genes engaged in protein biosynthesis were more abundant in the competent oocytes^52^. In this study, a large number of proteins (RPL30, RPL18A, RPL13A, RPL34, RPL26, RPS10, and RPL4) enriched in ribosome were found to be up-regulated in MII G oocytes compared to MII B oocytes, indicating that the protein synthesis was more active in MII G oocytes and level of protein synthesis in oocytes might be related to their developmental competence.

Furthermore, several proteins (UHRF1, UBE2C, USP28, UBE2H, UBE2L3, and UBE2K) related to ubiquitin-proteasome proteolytic pathway were found to be up-regulated in MII G oocytes in the present study. Ubiquitin-proteasome proteolytic pathway (UPP) is the main routes for intracellular protein degradation in eukaryotic cells^53^. Ubiquitin attached with substrate proteins and subsequently degraded by the 26S proteasome complex^53^. Numerous meiotic proteins involved in the regulation of cell cycle were found to be degraded by the UPP, such as cyclin B1, Cdc20, Cdc25, mos, and securin^54^. In rat oocytes, proteasomal catalytic activity was essential for the inactivity of MPF and completion of first meiosis^55^. Moreover, Huo *et al.* demonstrated that inhibition of UPP prevented cyclin B1 degradation, inhibited PB2 extrusion and pronuclear formation^56^. Degradation of cyclin B1 and securin mediated by UPP was required for disjunction of pairs of homologous chromosomes during the first meiotic division in mouse oocytes^57^. Therefore, UPP may play an essential role in the regulation of buffalo oocyte meiotic maturation.

## Conclusions

In conclusions, the expression level of proteins in buffalo oocytes is related to their physiological states, and the up-regulated proteins in competent MII oocytes compared to GV and incompetent MII oocytes are related to chromosome segregation, microtubule-based process, protein transport, ribosome, UPP, and OXPHOX. The oxidative phosphorylation activity may be important for the meiotic resumption and competent acquisition of buffalo oocytes during maturation.

## Materials and Methods

### Reagents and Media

Tissue culture medium 199 (TCM 199) and fetal calf serum were purchased from Gibco BRL (Paisley, Scotland, UK). Follicle-stimulating hormone, formic acid (FA), bis-acrylamide and acetonitrile (ACN) were supplied by Sigma (St, Louis, MO, USA). Urea, iodoacet-amide (IAA), ammonium bicarbonate (NH_4_HCO_3_) were obtained from Amresco (Solon, OH, USA). Protease inhibitor complete tablets was purchased from Roche. iTRAQ-4plex Regent kit was obtained from Applied Biosystems (Forster City, CA, USA). Trypsin was from Promega (Madison, WI, USA). All the water was prepared using a MilliQ system (Bedford, MA, USA).

### Oocyte collection

Buffalo ovaries were collected from the local slaughter house and transported to the laboratory in physiological saline at 25 °C. After washing in saline solution, cumulus-oocyte complexes (COCs) were aspirated from 2 to 6 mm follicles of ovaries and COCs with compact cumulus cell layers were selected for *in vitro* maturation (IVM). Then, COCs were cultured in droplets of TCM 199 medium supplemented with 10% fetal calf serum and antibiotics at 38.5 °C in an atmosphere of 5% CO_2_ for 22–24 h. The GV and M II oocytes of which cumulus cells were removed by vortexing and pipetting were washed three times in PBS buffer and stored at −80 °C until use. According to the morphological evaluation, Oocytes were divided into three kinds, I: immature oocytes with intact germinal vesicle and multilayered, compacted cumulus (GVO); II: competent matured oocytes with homogenous cytoplasm, at least three cumulus layers, and the first polar body (MII G); III: incompetent matured oocytes with heterogeneous cytoplasm, incompact and heterogeneously pigmented, and surrounding with few cumulus cells (MII B).

### Protein extraction and separation

All of the GV, competent and incompetent MII oocytes were lysed in 20 μL of lysis buffer (8 M urea, 50 mM IAA, and 1% (v/v) protease inhibitor cocktail). The samples were vortexed for 1 min and incubated on ice for 30 s with a total of 15 cycles. The lysates were centrifuged at 13,800× g for 3 min and the supernatants were collected. After adding of SDS buffer, the proteins of oocyte lysates (20 μg of each sample) were separated by 10% SDS-PAGE, stained with Coomassie brilliant blue. The gels were scanned with a Scanjet image system (HP Scanjet G4050), the gel image was analyzed by Scion Image (http://rsb.info.nih.gov/nihimage/).

### In-gel tryptic digestion

Gel lanes containing protein were sliced into 1 mm^3^ small pieces. Gel pieces were washed with 50 mM NH_4_HCO_3_, 30% ACN and dried in a speedavc followed by in-gel digestion with trypsin at 37 °C for 16 h. NH_4_HCO_3_ was added to a final concentration of 50 mM to stop the digestive reaction. Peptides were extracted from the gel pieces and desalted as described. Eluted peptides were dried with speedavc and stored at −80 °C until use.

### Peptides labeling using iTRAQ reagent

The peptides from each sample were first resuspended in 100 mM trithylammonium bicarbonate (TEAB). Then, iTRAQ regents were dissolved in 70 μL of ethanol by vortexing for 1 min. Oocytes at GV stage were labeled with iTRAQ tags 114 and 115. MII G and MII B samples were labeled with iTRAQ tags 116 and 117, respectively. Tubes were incubated for 2 h at room temperature. The reaction was stopped by adding 120 μL H_2_O, followed by centrifugation at 13,800 × g for 1 min. The samples were then pooled together into one fresh tube and dried with the speedavc.

### Peptide separation by high pH RP HPLC

The pooled peptides were resuspended in 80 μL of buffer A (98% ddH_2_0, 2% ACN, pH 10.0) and separated by hiph-pH reverse-phase LC column (2.1 × 100 mm, 3 μm, 150 Å, C18). The 60 min liner gradient was composed of 96% buffer A for 1 min; 4–19% buffer B (98% ACN, 2% ddH_2_0, pH 10.0) for 30 min; then 19–95% buffer B for 23 min; followed by 95% buffer B for 5 min. The eluted fractions were collected in every 1.5 min, and then pooled into 20 fractions based on the peak intensity. The collected fractions were then lyophilized and stored at −80 °C until MS analysis.

### Peptide identification by nano UPLC-MS/MS

Peptide fractions were suspended in buffer A (0.1% FA, 2% ACN) and analyzed by LTQ-Orbitrap Velos mass spectrometer (Thermo Fisher Scientific. San Jose. CA). Peptide mixtures were injected into the capillary column (75 μm × 15 cm) and separated by a 3 μm C18 column using NanoAcquity ultra performance liquid chromatography (UPLC) system (Waters, Milford, MA). Peptides were eluted with a linear gradient of 8–40% buffer B (0.1% FA in ACN) at a flow rate of 0.3 μL/min for 60 min. The mass spectrometer was operated in positive ion mode (source voltage 2 KV) and data-dependent manner. The full MS scans were performed in the Orbitrap at the range of 400–1,800 m/z at a resolution of 30,000. For MS/MS scans, the 10 most abundant ions with multiple charge states were selected for higher energy collisional dissociation (HCD) fragmentation following one MS full scan. Isolation window was set as 2.0 m/z. The dynamic exclusion was 35 s, and normalized collision energy of 40% was applied.

### Data processing and protein identification

The MS/MS spectra were obtained and searched using MaxQuant (version 1.5.1.2, Martinsried, Germany) against Uniprot *Bos taurus* protein database (version 13122013, 24,210 protein sequences). False discovery rate (FDR) was estimated by using target-decoy based strategy. The proteins and peptides were filtered with a FDR < 0.01. The enzyme parameter was limited to semi-tryptic peptides with a maximum miscleavage of 2. Peptides with at least six amino acids were accepted. For protein identification, at least one identified peptide was required. Peptides precursor mass tolerance was 20 ppm, and fragment mass tolerance was 0.1 Da. Variable modifications of oxidation (+15.9949 Da) of methionine was selected, and Carbamidomethylated cysteine (+57.0215 Da), iTRAQ 4-plex (K) and iTRAQ 4-plex (N-term) were set as fixed modification.

### Protein quantification

Protein with at least one unique peptide was validated and selected for further quantitative analysis. Only the peptide-spectrum matched (PSM) with complete reporter ion was allowed and the reporter ion intensity was picked up using the central MGF files. The isotopic correction was applied to reporter ion intensities according to the correction matrix provided by the manufacturer (Applied Biosystems). Peptide intensities were calculated by averaging the intensity of all the high confident PSMs of the peptide. The ratio of a protein is computed as the geometric mean of corresponding unique peptides ratios belonging to the protein. Significance B approach was performed to determine the p-value. Protein ratios greater than or equal to 2 or less than 0.5 (P-value less than 0.05 with at least two unique peptides) were considered to be differentially expressed proteins.

### Real-time Quantitative RT-PCR

Total RNA was extracted from five oocytes at different stages (GVO, MII G) using Cells-to-cDNA II Kit (Ambion Co., America). The cDNAs were reverse-transcribed using Takara RNA PCR kit (Takara) according to manufacturer’s instruction. Quantitative real-time RT-PCR analysis was performed using ABI 7500 PRISM system (Applied Biosystems, Singapore). The reaction system (20 μL) was consisted of 1 μL cDNA, 10 μL FastStart Universal SYBR Green Master (ROX) Mix, 0.5 μL of up and down primer (10 nM) and 8.5 μL ddH_2_O. The relative expression levels of targeted genes were calculated using the 2-^ΔΔ^CT method. Three replicates were carried out for each gene using different sets of oocytes. The primer sequences used for the qRT-PCR analysis were showed in Supplementary Information Table S5.

### Bioinformatics and statistical analysis

GProX^58^ was used for hierarchical clustering analysis. Gene Ontology and KEGG pathway were analyzed using the DAVID software 6.7 (http://david.abcc.ncifcrf.gov/). SPSS 17.0 was applied to evaluate the statistical significance of mean values. Probability values less than 0.05 was considered to be statistically significant.

## Additional Information

**How to cite this article**: Chen, L. *et al.* Comparative Proteomic Analysis of Buffalo Oocytes Matured *in vitro* Using iTRAQ Technique. *Sci. Rep.* **6**, 31795; doi: 10.1038/srep31795 (2016).

## Supplementary Material

Supplementary Information

## Figures and Tables

**Table 1:** Differential expression protein between different groups.

	MII G/GVO	MII G/MII B
Up-regulated	108	111
Down-regulated	65	35
Total	173	146
