# Supplementary material for: Comparative Proteomic Analysis of Buffalo Oocytes Matured in vitro Using iTRAQ Technique
Source: Sci Rep. 2016 Aug 26;6:31795. doi: 10.1038/srep31795 (PMC4999887; doi:10.1038/srep31795)
Supplement: Supplementary Information [file srep31795-s1.pdf]

Supplementary Information

Comparative Proteomics Analysis of Buffalo Oocyte Matured in vitro Using iTRAQ technique

Lingsheng Chen[1,2], Linhui Zhai[2,3], Chunfeng Qu[1,4], Chengpu Zhang[2], Sheng Li[1], Feilin Wu[2], Yingzi Qi[2], Fenghua Lu[1], Ping Xu[2,5,*], Xiangping Li[1,*] and Deshun Shi[1,*]

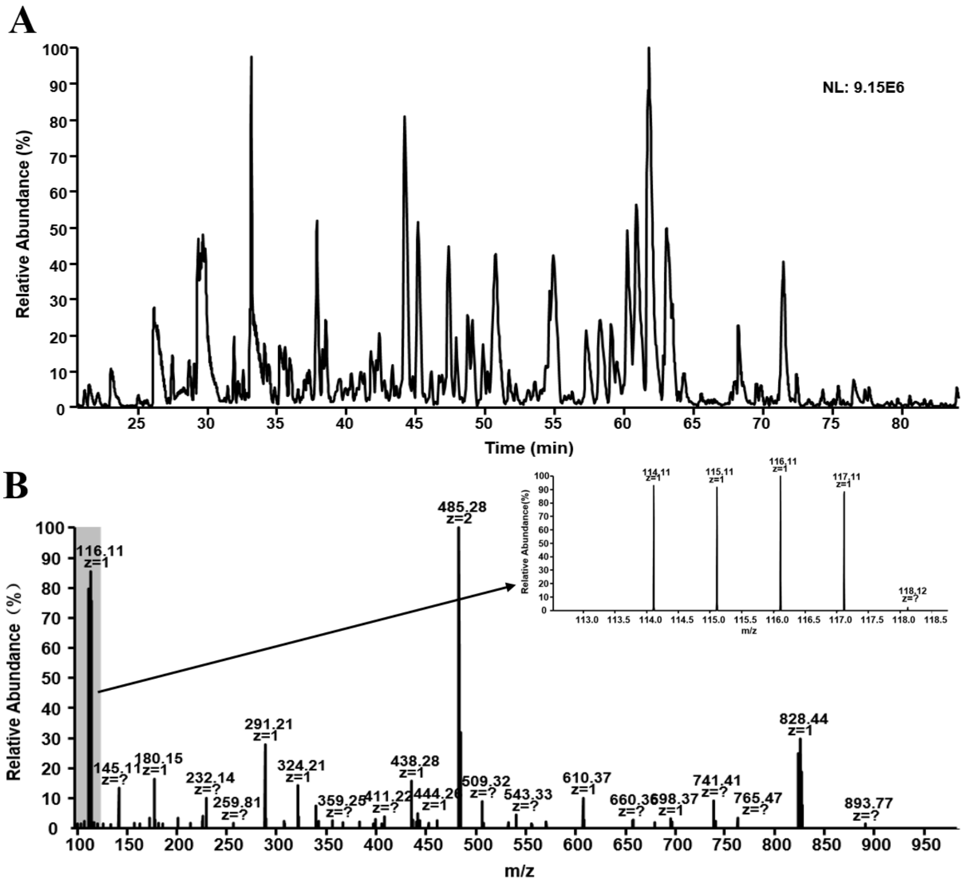

Figure S1. Identification of mass spectrometer. A. RP-HPLC/MS base peak ion chromatograms from iTRAQ-labeled buffalo oocytes. B. Representative MS/MS spectrum of peptide LYLTNSK. The insert demonstrated iTRAQ reporter ions regions.

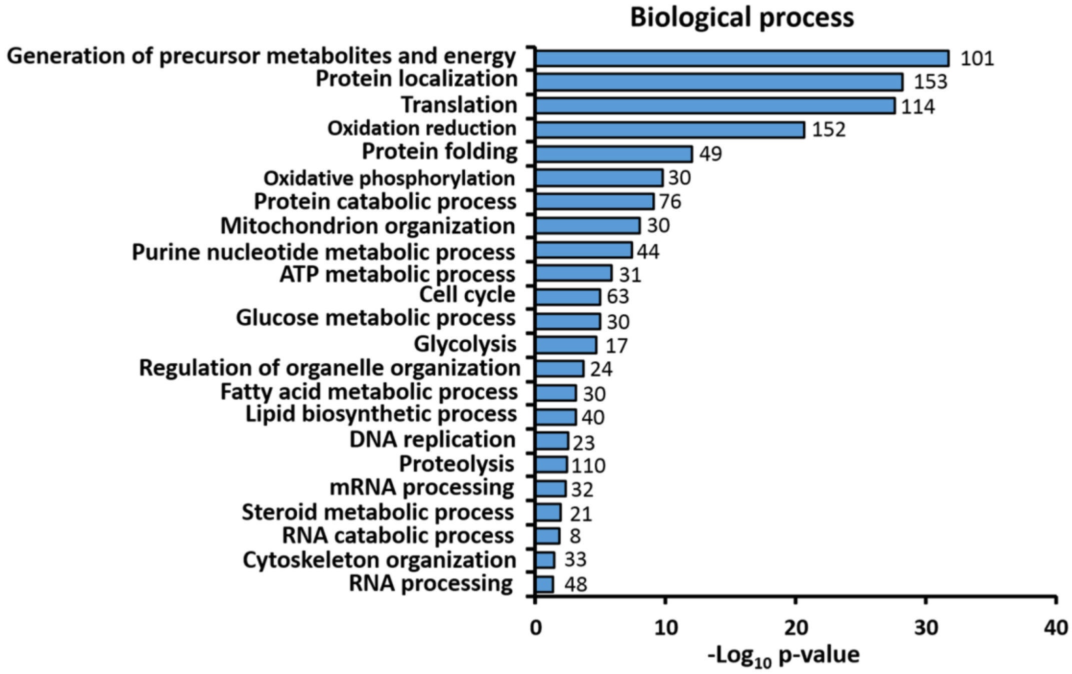

Figure S2. GO categorization of the total identified buffalo oocyte proteins.

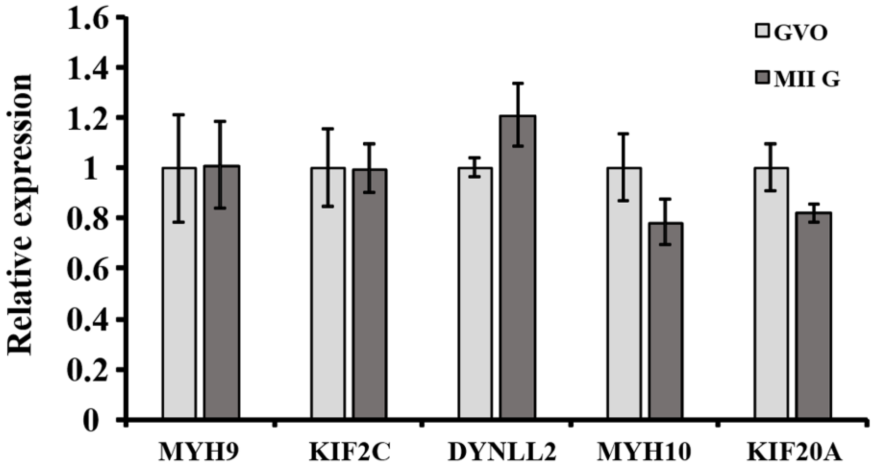

Figure S3.Validations of proteins by quantitative real-time PCR analysis. Bars represent standard errors (n=3).

Table S1. All proteins identified in the two Biological Replicates analyzed in the iTRAQ experiment (FDR < 1%).

				Biological Replicate 1			Biological Replicate 2		
Accession number	Gene symbol	Discription	MW(kDa)	Sequence coverage(%)	Unique peptides	MS/MS count	Sequence coverage(%)	Unique peptides	MS/MS count
Q0VC02	AAAS	Achalasia, adrenocortical insufficiency, alacrimia (Allgrove,	60.725	37.1	17	42	24.6	12	34
F1N412	AACS	Uncharacterized protein	75.02	17.9	11	17	2.5	2	2
Q3SZK1	AAMP	Angio-associated migratory cell	46.828	1.8	1	1	1.8	1	1
A6QLT9	AARS	AARS protein	106.65	19.2	17	23	6	6	13
F1MKA6	AARS2	Uncharacterized protein	106.36	4.8	4	4	#N/A	#N/A	#N/A
F1MS62	AASDHPPT	Uncharacterized protein	35.686	21.7	6	10	#N/A	#N/A	#N/A
G3X771	ABCB1	Uncharacterized protein (Fragment)	141.87	19.5	8	38	14	13	44
E1BLT5	ABCB4	Uncharacterized protein	140.38	8.2	1	1	5.5	1	4
A5D7P7	ABCB6	ABCB6 protein	93.677	1.3	1	1	#N/A	#N/A	#N/A
E1BPL3	ABCB7	Uncharacterized protein	83.084	12.6	8	10	5.7	5	10
G3MYQ9	ABCB8	Uncharacterized protein	77.314	13.7	8	11	11.1	8	15
F1MY01	ABCC1	Multidrug resistance-associated protein 1 (Fragment)	169.78	4.4	5	7	2.9	5	7
F1N3Y6	ABCC3	Uncharacterized protein	169.68	0.9	1	2	#N/A	#N/A	#N/A
F1MUC1	ABCC4	Uncharacterized protein (Fragment)	146.97	7.1	3	13	6.4	7	16
A7Z038	ABCD3	ABCD3 protein	75.358	11.9	8	13	16.9	11	19
A4IFE6	ABCE1	ABCE1 protein	67.288	25	14	20	9.5	7	13
E1BGH0	ABCF1	Uncharacterized protein	95.975	17.9	13	21	7.9	7	14
Q2KJA2	ABCF2	ATP-binding cassette sub-family F	71.475	15.7	9	17	2.9	2	3
A4FUE2	ABCF3	ABCF3 protein	79.765	3	1	1	2.4	2	2
Q4GZT4	ABCG2	ATP-binding cassette sub-family G	72.724	6	4	9	3.5	3	5
Q5E9H9	ABHD10	Mycophenolic acid acyl-glucuronide esterase, mitochondrial	33.982	28.4	7	10	19.6	6	11
Q3SZ73	ABHD11	Alpha/beta hydrolase domain-containing protein 11	33.548	6.9	2	3	2.3	1	2
Q08DW9	ABHD12	Monoacylglycerol lipase ABHD12	45.219	39.4	13	30	13.8	6	18
F1MR63	ABHD14A	Uncharacterized protein	55.519	17.4	7	10	#N/A	#N/A	#N/A
A7YY28	ABHD14B	Alpha/beta hydrolase domain-containing protein 14B	22.455	24.3	5	6	19.5	4	9

				Biological Replicate 1			Biological Replicate 2		
Accession number	Gene symbol	Discription	MW(kDa)	Sequence coverage(%)	Unique peptides	MS/MS count	Sequence coverage(%)	Unique peptides	MS/MS count
Q1JPD2	ABHD16A	Abhydrolase domain-containing	63.304	7	4	5	5.9	3	5
Q5EA59	ABHD4	Abhydrolase domain-containing	38.797	3.5	1	1	#N/A	#N/A	#N/A
Q1LZ86	ABHD6	Monoacylglycerol lipase ABHD6	38.352	6.2	2	3	6.2	3	3
Q0VCW2	ABI1	Abl-interactor 1	38.83	8.5	2	6	9.3	2	6
A7MB24	ABI2	ABI2 protein	52.372	4	1	2	#N/A	#N/A	#N/A
F1MWH8	ABL2	Uncharacterized protein	128.04	1.6	2	2	#N/A	#N/A	#N/A
Q3ZBN0	ABRACL	Costars family protein ABRACL	9.0464	16	1	1	#N/A	#N/A	#N/A
Q3ZC41	ACAA1	Acetyl-Coenzyme A acyltransferase 1	44.333	53.3	15	51	18.4	6	21
Q3T0R7	ACAA2	3-ketoacyl-CoA thiolase,	42.131	51.4	18	61	33.8	11	38
E1BGS2	ACAD10	Uncharacterized protein	117.62	2.6	3	7	2.5	3	5
Q0NXR6	ACAD8	Isobutyryl-CoA dehydrogenase, mitochondrial	45.307	16.1	6	10	14.4	6	16
F1N0A4	ACAD9	Uncharacterized protein	68.189	40.2	24	41	32.1	19	44
Q3SZB4	ACADM	Medium-chain specific acyl-CoA dehydrogenase, mitochondrial	46.573	34.9	12	23	19.7	9	16
F1MVL2	ACADS	Short-chain-specific acyl-CoA dehydrogenase, mitochondrial	44.387	35	1	40	21.1	1	38
Q5EAD4	ACADSB	Short/branched chain specific acyl-CoA dehydrogenase, mitochondrial	47.123	29.4	11	25	15.3	6	17
P48818	ACADVL	Very long-chain specific acyl-CoA dehydrogenase, mitochondrial	70.648	42.7	21	34	20.6	12	18
Q29RZ0	ACAT1	Acetyl-CoA acetyltransferase, mitochondrial	44.889	52.8	17	87	30.6	12	64
Q17QI3	ACAT2	Acetyl-Coenzyme A acetyltransferase	41.17	52.4	17	60	23.4	9	25
F1MRE5	ACBD3	Uncharacterized protein	60.581	17.6	8	13	8.7	5	10
F1MBE7	ACCSL	Uncharacterized protein (Fragment)	66.603	66.2	32	146	22.3	15	70
F1MQJ0	ACE	Angiotensin-converting enzyme	141.24	1.7	2	2	#N/A	#N/A	#N/A
Q32PF2	ACLY	ATP-citrate synthase	119.79	25.9	27	48	9.4	12	22
Q0VCU1	ACO1	Cytoplasmic aconitate hydratase	98.203	10.1	8	8	#N/A	#N/A	#N/A
P20004	ACO2	Aconitate hydratase, mitochondrial	85.358	59	39	103	39.7	27	75
E1BMF5	ACOT11	Uncharacterized protein (Fragment)	66.986	11.5	6	7	3	2	5
F1N234	ACOT2	Uncharacterized protein	50.89	#N/A	#N/A	#N/A	4.1	2	2

				Biological Replicate 1			Biological Replicate 2		
Accession number	Gene symbol	Discription	MW(kDa)	Sequence coverage(%)	Unique peptides	MS/MS count	Sequence coverage(%)	Unique peptides	MS/MS count
F6Q5U0	ACOT7	Uncharacterized protein	40.838	2.9	1	1	#N/A	#N/A	#N/A
F6RWU6	ACOT8	Uncharacterized protein	35.679	10.7	3	4	#N/A	#N/A	#N/A
Q3SWX2	ACOT9	Acyl-coenzyme A thioesterase 9, mitochondrial	49.852	29.5	12	24	16.7	8	16
Q3SZP5	ACOX1	Peroxisomal acyl-coenzyme A oxidase	74.378	4.8	3	3	7.1	5	9
F1MQG3	ACOX3	Acyl-coenzyme A oxidase	70.656	2.5	1	1	#N/A	#N/A	#N/A
A5PK96	ACP1	ACP1 protein	18.169	44.9	2	27	22.2	2	23
A6H757	ACP6	Lysophosphatidic acid phosphatase	48.944	6.5	3	3	2.3	1	2
Q58DN7	ACSF3	Acyl-CoA synthetase family member 3, mitochondrial	64.989	3.4	2	2	1.7	1	1
F1MEX9	ACSL3	Uncharacterized protein	80.353	41.9	21	55	21.5	11	33
F1MBW3	ACSL4	Uncharacterized protein (Fragment)	80.609	36.1	20	34	21.1	13	60
Q1LZF6	ACSL5	Acyl-CoA synthetase long-chain family member 5	76.027	34.4	20	41	16.8	11	36
Q2TA22	ACSL6	Acyl-CoA synthetase long-chain family member 6	78.042	18.9	11	20	14.6	9	19
F1MQV8	ACSS3	Acyl-CoA synthetase short-chain family member 3, mitochondrial	74.744	7.6	4	6	5.2	4	5
Q3ZC07	ACTC1	Actin, alpha cardiac muscle 1	42.019	34	1	21	32.4	1	7
P63258	ACTG1	Actin, cytoplasmic 2	41.792	74.7	12	609	61.1	9	320
A4IFJ8	ACTL6A	ACTL6A protein	47.444	8.2	3	3	4.7	2	4
Q3B7N2	ACTN1	Alpha-actinin-1	102.98	54.7	23	61	29.8	13	29
Q3ZC55	ACTN2	Alpha-actinin-2	103.78	19.1	1	1	#N/A	#N/A	#N/A
A5D7D1	ACTN4	Alpha-actinin-4	104.93	61.3	37	165	37.5	19	93
Q3ZBD2	ACTR10	Actin-related protein 10	46.296	19.9	7	12	3.6	2	5
F2Z4F0	ACTR1A	Uncharacterized protein	42.613	35.4	6	17	15.4	2	4
A4IFE3	ACTR1B	Beta-centractin	42.294	27.4	4	6	20.2	3	14
A7MB62	ACTR2	Actin-related protein 2	44.76	43.4	15	51	26.4	11	43
P61157	ACTR3	Actin-related protein 3	47.371	63.2	18	92	33.5	11	50
F1N6J8	ACTR3C	Uncharacterized protein (Fragment)	46.546	18.4	3	7	12.5	2	2
Q3T0V2	ACY1	Aminoacylase 1	41.753	#N/A	#N/A	#N/A	8.8	4	9
P56658	ADA	Adenosine deaminase	40.919	13.5	5	6	8	3	5

				Biological Replicate 1			Biological Replicate 2		
**Accession number**	**Gene symbol**	**Discription**	**MW(kDa)**	**Sequence coverage(%)**	**Unique peptides**	**MS/MS count**	**Sequence coverage(%)**	**Unique peptides**	**MS/MS count**
F1N4S1	ADAD2	Uncharacterized protein (Fragment)	61.818	1.7	1	1	#N/A	#N/A	#N/A
Q0VC13	ADAL	Adenosine deaminase-like protein	39.646	9.7	3	4	#N/A	#N/A	#N/A
Q10741	ADAM10	Disintegrin and metalloproteinase domain-containing protein 10	84.188	4.4	3	10	1.3	1	2
E1B867	ADAM17	Uncharacterized protein	92.861	1.6	1	1	#N/A	#N/A	#N/A
F1ME22	ADAM19	Uncharacterized protein	99.846	#N/A	#N/A	#N/A	2.1	2	2
Q29RI0	ADCK3	Chaperone activity of bc1 complex-like, mitochondrial	72.082	4	2	3	#N/A	#N/A	#N/A
Q3ZC42	ADH5	Alcohol dehydrogenase class-3	39.677	46.8	15	61	26.2	10	56
F1N468	ADK	Uncharacterized protein	38.532	6.4	2	3	#N/A	#N/A	#N/A
A2VE47	ADPGK	ADP-dependent glucokinase	54.085	7.6	3	5	4.8	2	5
Q3SYV9	ADPRHL2	Poly(ADP-ribose) glycohydrolase	39.221	10.7	3	7	2.7	1	1
A1L5A6	ADRM1	Proteasomal ubiquitin receptor	41.954	14.7	6	11	10.1	4	7
F1MHP6	ADSL	Adenylosuccinate lyase	55.467	58	21	48	10	5	17
A7MBG0	ADSS	Adenylosuccinate synthetase isozyme	50.156	45	21	71	28.3	13	42
F1MXK1	AES	Uncharacterized protein (Fragment)	35.043	9.4	3	3	#N/A	#N/A	#N/A
Q2KJI7	AFG3L2	AFG3-like protein 2	89.387	48.1	34	93	26.7	22	74
E1BLL5	AFMID	Kynurenine formamidase	34.334	3.9	1	1	#N/A	#N/A	#N/A
F1MGY9	AGA	Uncharacterized protein (Fragment)	37.107	10.3	3	5	#N/A	#N/A	#N/A
Q2TA45	AGFG1	Arf-GAP domain and FG repeat-containing protein 1	58.133	16	5	12	7.3	4	4
F1MS35	AGFG2	Uncharacterized protein	48.182	3.6	1	1	#N/A	#N/A	#N/A
A6QR07	AGK	AGK protein	46.745	38.6	11	28	18.8	8	19
Q95JH2	AGPAT1	1-acyl-sn-glycerol-3-phosphate acyltransferase alpha	32.005	19.9	4	6	16	4	8
G5E5P6	AGPAT3	Uncharacterized protein	43.391	5.9	2	4	#N/A	#N/A	#N/A
Q0IID8	AGPAT5	1-acylglycerol-3-phosphate O-acyltransferase 5 (Lysophosphatidic acid acyltransferase, epsilon)	33.571	22.3	7	11	6.2	2	2
A6H7D4	AGPAT6	1-acylglycerol-3-phosphate O-acyltransferase 6 (Lysophosphatidic acid acyltransferase, zeta)	52.065	9.2	3	3	4.6	2	2

				Biological Replicate 1			Biological Replicate 2		
Accession number	Gene symbol	Discription	MW(kDa)	Sequence coverage(%)	Unique peptides	MS/MS count	Sequence coverage(%)	Unique peptides	MS/MS count
E1BGF8	AGPAT9	Uncharacterized protein	49.254	3	1	1	#N/A	#N/A	#N/A
E1BPV2	AGPS	Uncharacterized protein (Fragment)	64.874	29.9	16	28	14.6	8	18
Q17QI4	AGTRAP	Angiotensin II receptor-associated	16.552	#N/A	#N/A	#N/A	5.4	1	4
Q3MHL4	AHCY	Adenosylhomocysteinase	47.637	51.6	24	159	44.2	20	116
F1MWH2	AHCYL1	Adenosylhomocysteinase	58.951	17.2	2	3	6.4	2	8
A6QLP2	AHCYL2	Adenosylhomocysteinase	66.773	13.7	1	17	#N/A	#N/A	#N/A
Q3T0G3	AHSA1	AHA1, activator of heat shock 90kDa protein ATPase homolog 1 (Yeast)	38.241	37.9	11	20	16.9	6	12
E1BG08	AIDA	Uncharacterized protein	34.995	23.5	9	15	11.1	3	3
E1BJA2	AIFM1	Uncharacterized protein	66.871	35.2	18	46	25	14	50
F1MW53	AIG1	Uncharacterized protein	27.326	#N/A	#N/A	#N/A	3.8	1	1
E1BH02	AIM1	Uncharacterized protein	187.58	1.8	2	2	1.9	3	4
Q0II26	AIMP2	Aminoacyl tRNA synthase complex-interacting multifunctional protein	35.178	25	7	13	7.2	3	5
Q3SZ99	AIP	AH receptor-interacting protein	37.59	29.4	9	18	17.9	5	6
P00570	AK1	Adenylate kinase isoenzyme 1	21.664	55.2	10	38	27.8	5	10
P08760	AK3	GTP:AMP phosphotransferase AK3, mitochondrial	25.671	68.3	15	32	34.4	8	19
Q0VCP1	AK4	Adenylate kinase 4, mitochondrial	25.348	53.4	13	62	37.2	9	45
E1BM36	AKAP1	Uncharacterized protein	88.593	17.2	12	20	9.6	7	11
A5D7T9	AKAP10	AKAP10 protein	67.914	11.6	6	10	3.3	2	2
A8YXY8	AKIRIN2	Akirin-2	22.509	5.9	1	2	10.8	2	2
Q3ZCJ2	AKR1A1	Alcohol dehydrogenase [NADP(+)]	36.617	69.5	22	91	45.2	17	55
P16116	AKR1B1	Aldose reductase	35.919	85.4	1	1	60.3	1	202
F1N678	AKR1B10	Uncharacterized protein	35.549	5.5	2	19	2.6	1	16
F1MZC0	AKR7A2	Uncharacterized protein (Fragment)	30.045	55.3	12	43	24.4	6	21
F1MYJ4	AKT3	Uncharacterized protein	55.774	1.9	1	1	#N/A	#N/A	#N/A
Q58DK5	ALAD	Delta-aminolevulinic acid	36.081	20.4	5	8	11.2	3	4
Q2KJH7	ALDH18A1	Aldehyde dehydrogenase 18 family, member A1	87.193	17.4	13	20	7.3	6	12
P48644	ALDH1A1	Retinal dehydrogenase 1	54.805	28.9	11	22	9	4	9
P20000	ALDH2	Aldehyde dehydrogenase,	56.652	37.3	14	27	9.8	5	19

				Biological Replicate 1			Biological Replicate 2		
Accession number	Gene symbol	Discription	MW(kDa)	Sequence coverage(%)	Unique peptides	MS/MS count	Sequence coverage(%)	Unique peptides	MS/MS count
A6QQT4	ALDH3A2	Aldehyde dehydrogenase	54.075	25.8	10	10	21.2	9	26
A7YWE4	ALDH4A1	Delta-1-pyrroline-5-carboxylate dehydrogenase, mitochondrial	61.495	40	22	49	25.9	15	42
E1BDP3	ALDH5A1	Uncharacterized protein	56.096	1.7	1	1	#N/A	#N/A	#N/A
E1BFG0	ALDH7A1	Alpha-aminoadipic semialdehyde dehydrogenase	58.595	54	29	130	43	21	85
A6QLL8	ALDOA	Fructose-bisphosphate aldolase	39.436	69.5	16	63	54.7	17	60
Q3ZBY4	ALDOC	Fructose-bisphosphate aldolase	39.381	71.7	18	69	33.2	12	32
F1N2J7	ALG1	Uncharacterized protein	51.899	#N/A	#N/A	#N/A	2.8	1	1
E1B756	ALG11	Uncharacterized protein (Fragment)	56.529	3	2	4	2.8	1	1
G3MWZ3	ALG12	Uncharacterized protein	54.553	3.5	1	2	#N/A	#N/A	#N/A
Q17QD5	ALG14	Asparagine-linked glycosylation 14	18.799	11.6	2	2	4.6	1	2
A4FUG6	ALG2	ALG2 protein	46.831	22.4	8	22	8.4	4	9
A4FV21	ALG3	ALG3 protein	49.889	6.8	3	3	2.3	1	1
F6QF86	ALG6	Uncharacterized protein	58.304	3.9	2	4	4.7	3	8
Q0P5D9	ALG8	Probable dolichyl pyrophosphate Glc1Man9GlcNAc2 alpha-1,3-glucosyltransferase	59.963	6.7	4	4	3.2	2	2
E1BA58	ALG9	Uncharacterized protein	69.851	10.6	4	4	#N/A	#N/A	#N/A
P09487	ALPL	Alkaline phosphatase, tissue-nonspecific isozyme	57.192	4.8	2	3	8	4	8
F1MCN8	AMACR	Uncharacterized protein	42.126	16.5	7	12	11.5	4	8
A7MBC0	AMDHD2	Putative N-acetylglucosamine-6-phosphate deacetylase	43.495	4.6	2	3	2.4	1	2
A6QPH6	AMICA1	AMICA1 protein	43.026	#N/A	#N/A	#N/A	6.8	3	6
E1BEQ5	AMOTL1	Angiomotin-like protein 1	106.67	#N/A	#N/A	#N/A	1.5	1	2
E1BLG8	AMPD2	Uncharacterized protein	92.125	7	5	6	2.3	2	3
E1BA23	AMPD3	Uncharacterized protein (Fragment)	89.265	4.3	4	4	#N/A	#N/A	#N/A
P25285	AMT	Aminomethyltransferase,	42.868	2	1	2	#N/A	#N/A	#N/A
E1B9T4	AMZ1	Uncharacterized protein	54.157	1.6	1	1	#N/A	#N/A	#N/A
F1MH25	AMZ2	Uncharacterized protein	38.099	2.4	1	1	#N/A	#N/A	#N/A
E1BAB6	ANAPC1	Uncharacterized protein	217.03	#N/A	#N/A	#N/A	0.5	1	1

				Biological Replicate 1			Biological Replicate 2		
Accession number	Gene symbol	Discription	MW(kDa)	Sequence coverage(%)	Unique peptides	MS/MS count	Sequence coverage(%)	Unique peptides	MS/MS count
E1B945	ANAPC4	Uncharacterized protein	91.976	2	1	1	#N/A	#N/A	#N/A
G3N3N0	ANK2	Uncharacterized protein (Fragment)	154.12	5.3	5	7	2.4	2	2
F1MD79	ANKFY1	Uncharacterized protein (Fragment)	130.05	28.4	29	68	9.8	12	30
E1BFD1	ANKLE2	Uncharacterized protein	106.24	1.3	1	1	#N/A	#N/A	#N/A
Q0VCS9	ANKMY2	Ankyrin repeat and MYND domain-containing protein 2	49.407	#N/A	#N/A	#N/A	2.5	1	1
E1BAT5	ANKRD11	Uncharacterized protein	290.46	#N/A	#N/A	#N/A	0.3	1	1
F1N545	ANKRD13A	Uncharacterized protein	67.497	2	1	2	#N/A	#N/A	#N/A
F1MI56	ANKRD17	Uncharacterized protein	275.01	1.1	2	2	#N/A	#N/A	#N/A
F1N642	ANKRD27	Uncharacterized protein	117.12	1.2	1	1	#N/A	#N/A	#N/A
G3X683	ANKRD29	Uncharacterized protein (Fragment)	31.733	2.7	1	2	2.7	1	4
A6QQP9	ANKRD40	ANKRD40 protein	40.558	5.5	2	2	#N/A	#N/A	#N/A
Q5EA33	ANKRD49	Ankyrin repeat domain-containing protein 49	27.196	5.5	1	1	#N/A	#N/A	#N/A
F1MBT6	ANLN	Uncharacterized protein (Fragment)	124.56	2.1	2	2	0.9	1	1
F1MX70	ANO10	Anoctamin (Fragment)	64.039	23.3	13	23	14.8	10	19
F1MZ46	ANP32B	Acidic leucine-rich nuclear phosphoprotein 32 family member B	17.31	50	7	14	12.3	2	5
P46193	ANXA1	Annexin A1	38.951	11.8	4	5	#N/A	#N/A	#N/A
F1MB53	ANXA10	Uncharacterized protein	36.975	3.1	1	1	#N/A	#N/A	#N/A
P27214	ANXA11	Annexin A11	54.018	39.2	20	46	30.6	14	44
P04272	ANXA2	Annexin A2	38.612	49	14	25	19.8	7	14
P13214	ANXA4	Annexin A4	35.888	20.1	5	5	#N/A	#N/A	#N/A
F6QVC9	ANXA5	Annexin	36.074	72.3	1	81	32.1	10	23
P79134	ANXA6	Annexin A6	75.907	18.3	12	21	9.4	6	8
P20072	ANXA7	Annexin A7	49.94	17.3	7	9	8.9	3	4
F1MIF2	AP1B1	Uncharacterized protein	104.15	43.2	18	31	16.2	9	19
F1MF68	AP1G1	Uncharacterized protein	91.396	20.1	16	30	12.9	12	24
A8E4N0	AP1G2	AP1G2 protein	72.355	8.8	5	7	1.5	1	2
Q2KJ81	AP1M1	AP-1 complex subunit mu-1	48.586	14.2	2	2	#N/A	#N/A	#N/A
Q3SYW1	AP1M2	AP-1 complex subunit mu-2	48.149	31.9	10	25	13	6	8
Q1JQ98	AP1S1	AP-1 complex subunit sigma-1A	18.602	29.9	5	8	5.7	1	1

				Biological Replicate 1			Biological Replicate 2		
Accession number	Gene symbol	Discription	MW(kDa)	Sequence coverage(%)	Unique peptides	MS/MS count	Sequence coverage(%)	Unique peptides	MS/MS count
Q0VCK5	AP2A2	AP-2 complex subunit alpha-2	103.77	47.2	25	101	26.4	14	64
P63009	AP2B1	AP-2 complex subunit beta	104.57	46.4	22	102	17.8	10	59
Q3ZC13	AP2M1	AP-2 complex subunit mu	49.654	47.1	20	42	31.5	14	41
Q17QC5	AP2S1	AP-2 complex subunit sigma	17.018	29.6	4	8	4.9	1	1
Q32PG1	AP3B1	AP-3 complex subunit beta-1	119.97	18.7	17	30	10.9	9	20
E1BME2	AP3B2	Uncharacterized protein	119.52	2.8	1	1	2.8	1	2
Q865S1	AP3D1	AP-3 complex subunit delta-1	136.39	15.1	12	15	2.5	3	3
Q24K11	AP3M1	AP-3 complex subunit mu-1	46.897	#N/A	#N/A	#N/A	6.7	3	3
E1B763	AP3M2	Uncharacterized protein	47.005	10.5	2	5	#N/A	#N/A	#N/A
Q2YDH6	AP3S1	AP-3 complex subunit sigma-1	21.732	29	5	11	19.2	4	6
F1MUW4	APAF1	Uncharacterized protein (Fragment)	142.04	7.6	9	13	2	3	4
Q3T004	APCS	Serum amyloid P-component	25.183	5.8	2	2	#N/A	#N/A	#N/A
P80227	APEH	Acylamino-acid-releasing enzyme	81.092	31.9	18	33	12.3	9	17
P23196	APEX1	DNA-(apurinic or apyrimidinic site)	35.569	8.5	2	4	#N/A	#N/A	#N/A
Q0VCJ2	APIP	Methylthioribulose-1-phosphate	27.095	16.5	3	3	#N/A	#N/A	#N/A
A0JNH9	APLF	Aprataxin and PNK-like factor	54.325	2.5	1	2	6	3	5
F1N226	APLP2	Uncharacterized protein (Fragment)	83.898	5.1	3	4	4.9	4	8
Q3T0E5	APMAP	Adipocyte plasma membrane-associated protein	46.09	50.5	21	58	37.1	16	43
Q6QRN6	APOA1BP	NAD(P)H-hydrate epimerase	31.363	37.2	10	41	29.9	8	41
Q3SYR3	APOBEC2	Probable C->U-editing enzyme	25.962	3.1	1	1	#N/A	#N/A	#N/A
P19035	APOC3	Apolipoprotein C-III	10.692	#N/A	#N/A	#N/A	16.7	1	1
Q148H0	APOO	Apolipoprotein O	22.562	24.2	5	11	14.1	3	18
Q1RMW4	APPL2	Adaptor protein, phosphotyrosine interaction, PH domain and leucine zipper containing 2	73.972	11.8	6	6	3	2	2
Q56JW4	APRT	Adenine phosphoribosyltransferase	19.537	20	4	7	12.8	2	4
Q2KJA0	ARAF	ARAF protein	68.145	#N/A	#N/A	#N/A	3.1	2	2
P53619	ARCN1	Coatomer subunit delta	57.274	45.8	26	64	29.5	17	36
P84081	ARF2	ADP-ribosylation factor 2	20.746	58.6	1	2	#N/A	#N/A	#N/A
Q5E9I6	ARF3	ADP-ribosylation factor 3	20.601	58.6	1	54	35.9	4	18
Q3SZF2	ARF4	ADP-ribosylation factor 4	20.527	64.4	4	7	33.3	3	12

				Biological Replicate 1			Biological Replicate 2		
Accession number	Gene symbol	Discription	MW(kDa)	Sequence coverage(%)	Unique peptides	MS/MS count	Sequence coverage(%)	Unique peptides	MS/MS count
A4IFP7	ARF5	ARF5 protein	20.529	56.1	3	13	33.3	2	53
G3N3N1	ARF6	Uncharacterized protein	20.082	33.1	4	5	#N/A	#N/A	#N/A
A6QR32	ARFGAP2	ARFGAP2 protein	53.256	1.6	1	1	#N/A	#N/A	#N/A
Q08DK2	ARFGAP3	ADP-ribosylation factor GTPase activating protein 3	44.287	4.2	2	3	4.2	2	3
O46382	ARFGEF1	Brefeldin A-inhibited guanine nucleotide-exchange protein 1	208.71	#N/A	#N/A	#N/A	1.4	1	4
E1BKI9	ARFGEF2	Uncharacterized protein	202.48	4.3	7	7	#N/A	#N/A	#N/A
F1MAV2	ARFIP1	Uncharacterized protein	41.729	41.8	14	56	18	7	21
Q3ZCL5	ARFIP2	Arfaptin-2	37.71	12.9	5	8	8.5	3	5
Q32LJ2	ARFRP1	ADP-ribosylation factor-related	22.644	#N/A	#N/A	#N/A	10.4	2	4
Q58DL1	ARG2	Arginase-2, mitochondrial	38.504	18.4	5	8	15.8	5	8
F6RWK1	ARHGAP1	Uncharacterized protein	50.476	6.2	3	4	4.8	2	3
Q08DP6	ARHGAP10	Rho GTPase-activating protein 10	89.404	1.4	1	1	#N/A	#N/A	#N/A
A6QPU2	ARHGAP12	ARHGAP12 protein	89.818	6.4	3	3	2.9	2	3
F1N458	ARHGAP17	Uncharacterized protein (Fragment)	91.813	3.3	3	4	#N/A	#N/A	#N/A
E1BIH5	ARHGAP18	Uncharacterized protein	59.62	2.4	1	1	#N/A	#N/A	#N/A
F1MJ96	ARHGEF37	Uncharacterized protein	76.148	41.2	24	47	11.3	8	18
F1MBM9	ARHGEF7	Uncharacterized protein	97.196	11	8	10	8.8	8	11
A2VEA3	ARIH1	E3 ubiquitin-protein ligase ARIH1	64.016	11.7	6	9	#N/A	#N/A	#N/A
Q2YDM1	ARL1	ADP-ribosylation factor-like	20.427	55.2	7	16	6.1	1	3
Q1RMP1	ARL10	ADP-ribosylation factor-like 10	24.305	15.1	2	5	5.5	1	6
Q2TA37	ARL2	ADP-ribosylation factor-like	20.907	49.5	9	20	28.8	5	10
G1K192	ARL3	ADP-ribosylation factor-like protein 3 (Fragment)	20.373	32	5	8	10.5	2	2
Q0IIM2	ARL6	ADP-ribosylation factor-like	21.055	8.6	2	3	#N/A	#N/A	#N/A
F1MBM4	ARL6IP1	Uncharacterized protein	16.74	16.7	3	5	16.7	3	19
Q5E9M1	ARL6IP5	PRA1 family protein 3	21.666	5.9	1	2	5.9	1	1
G3X6L5	ARL8A	Uncharacterized protein (Fragment)	20.484	25.1	1	4	15.6	1	1
Q2KI07	ARL8B	ADP-ribosylation factor-like	21.511	49.5	5	32	22.6	2	21
Q3ZBE1	ARMC1	Armadillo repeat-containing protein	31.279	31.2	11	21	13.8	5	13

				Biological Replicate 1			Biological Replicate 2		
Accession number	Gene symbol	Discription	MW(kDa)	Sequence coverage(%)	Unique peptides	MS/MS count	Sequence coverage(%)	Unique peptides	MS/MS count
E1B717	ARMC10	Uncharacterized protein	33.644	44.6	9	41	27	6	15
A6H718	ARMC6	ARMC6 protein	50.482	2.8	1	3	#N/A	#N/A	#N/A
Q2KI54	ARMC8	Armadillo repeat-containing protein	75.45	4.5	3	4	#N/A	#N/A	#N/A
Q1JP79	ARPC1A	Actin-related protein 2/3 complex subunit 1A	41.539	50.3	16	41	25.4	10	26
Q58CQ2	ARPC1B	Actin-related protein 2/3 complex subunit 1B	40.975	38.4	15	34	18	8	29
Q3MHR7	ARPC2	Actin-related protein 2/3 complex subunit 2	34.349	69.7	22	63	31.7	10	43
Q3T035	ARPC3	Actin-related protein 2/3 complex subunit 3	20.546	27.5	5	24	15.7	3	11
Q148J6	ARPC4	Actin-related protein 2/3 complex subunit 4	19.667	42.9	7	21	28	5	24
Q3SYX9	ARPC5	Actin-related protein 2/3 complex subunit 5	16.23	43	6	15	48.3	7	19
Q5E963	ARPC5L	Actin-related protein 2/3 complex subunit 5-like protein	16.941	46.4	7	15	27.5	5	8
Q17QU3	ARPIN	Arpin	24.886	11.9	2	3	#N/A	#N/A	#N/A
Q0P5B0	ARRDC1	Arrestin domain containing 1	45.688	7.3	3	3	#N/A	#N/A	#N/A
Q08DD1	ARSA	Arylsulfatase A	53.806	11.8	5	15	9.1	5	9
A6QLZ3	ARSB	ARSB protein	59.355	2.1	1	1	#N/A	#N/A	#N/A
Q58DQ0	AS3MT	Arsenic (+3 oxidation state) methyltransferase	41.742	35.6	11	21	15.8	6	19
Q17QB3	ASAH1	Acid ceramidase	44.965	20.8	8	23	8.6	4	7
E1BJU5	ASAH2	Uncharacterized protein	83.5	5.8	4	6	2.6	2	4
E1BEH6	ASB1	Uncharacterized protein	36.626	3.3	1	1	#N/A	#N/A	#N/A
F1N2K6	ASCC2	Uncharacterized protein	53.183	1.7	1	1	#N/A	#N/A	#N/A
Q17QJ0	ASF1B	Histone chaperone ASF1B	22.421	28.2	3	7	4	1	4
Q3SZJ0	ASL	Argininosuccinate lyase	52.742	17.1	7	7	#N/A	#N/A	#N/A
A5PJI5	ASNA1	ATPase ASNA1	38.792	17.5	6	11	7.2	3	4
Q1LZA3	ASNS	Asparagine synthetase [glutamine-hydrolyzing]	64.219	46.3	24	63	18.7	11	27

				Biological Replicate 1			Biological Replicate 2		
Accession number	Gene symbol	Discription	MW(kDa)	Sequence coverage(%)	Unique peptides	MS/MS count	Sequence coverage(%)	Unique peptides	MS/MS count
P62285	ASPM	Abnormal spindle-like microcephaly-associated protein homolog	395.09	0.2	1	1	0.2	1	2
F1MTZ8	ASPSCR1	Uncharacterized protein	61.346	12.4	5	9	8.6	4	11
Q32LE5	ASRGL1	Isoaspartyl peptidase/L-	32.05	51.9	12	36	22.7	7	34
F1MP41	ASTL	Uncharacterized protein (Fragment)	39.959	65	18	184	31.9	11	99
F6QV99	ATAD1	ATPase family AAA domain-containing protein 1	40.774	14.7	4	7	8	3	7
A7YWC4	ATAD3	ATPase family AAA domain-containing protein 3	66.106	9.2	6	8	13.7	9	11
E1BI60	ATE1	Arginyl-tRNA--protein transferase 1	59.452	9.7	5	7	1.5	1	2
F1MZE2	ATG12	Ubiquitin-like protein ATG12	13.924	20.3	3	6	#N/A	#N/A	#N/A
E1BGS8	ATG16L1	Uncharacterized protein	70.017	3.9	3	4	2.4	2	4
Q0VCL3	ATG3	Ubiquitin-like-conjugating enzyme	35.752	26.4	8	19	10.5	3	6
Q3MQ24	ATG5	Autophagy protein 5	32.431	6.9	2	4	#N/A	#N/A	#N/A
G5E627	ATG7	Uncharacterized protein	77.496	8.7	6	8	5.9	4	5
Q0VCK0	ATIC	Bifunctional purine biosynthesis protein PURH	64.482	68.4	36	106	30.4	16	56
Q58D72	ATL1	Atlastin-1	63.534	5.7	2	2	#N/A	#N/A	#N/A
E1BE28	ATL2	Uncharacterized protein	66.347	11.3	5	10	6.2	4	7
A5PJD6	ATL3	ATL3 protein	60.311	26.2	10	15	8.1	5	8
Q3T0E0	ATOX1	Copper transport protein ATOX1	7.3654	11.8	1	2	11.8	1	3
F1MSL3	ATP10D	Uncharacterized protein	159.94	2.6	4	4	0.5	1	1
F1MW32	ATP11B	Uncharacterized protein (Fragment)	134.27	3	2	2	1.4	2	2
F1N3G6	ATP11C	Uncharacterized protein (Fragment)	129.64	20.2	19	38	11.6	14	29
F1MYA8	ATP13A1	Uncharacterized protein	132.59	4.3	5	5	2.1	3	3
Q08DA1	ATP1A1	Sodium/potassium-transporting ATPase subunit alpha-1	112.64	25.8	25	48	24.5	22	64
G3MWR4	ATP1B1	Uncharacterized protein	35.023	19.8	4	9	14.2	4	11
Q3T0C6	ATP1B3	Sodium/potassium-transporting ATPase subunit beta-3	31.521	9	3	5	13.3	4	10
Q0VCY0	ATP2A1	Sarcoplasmic/endoplasmic reticulum calcium ATPase 1	109.29	#N/A	#N/A	#N/A	4.9	1	3

				Biological Replicate 1			Biological Replicate 2		
Accession number	Gene symbol	Discription	MW(kDa)	Sequence coverage(%)	Unique peptides	MS/MS count	Sequence coverage(%)	Unique peptides	MS/MS count
F1MPR3	ATP2A2	Uncharacterized protein	111.93	17.5	14	18	10	7	18
F1N7J2	ATP2B1	Uncharacterized protein	134.78	6.9	8	10	6.4	4	11
F1MS16	ATP2B3	Uncharacterized protein	132.88	#N/A	#N/A	#N/A	3.3	1	1
P57709	ATP2C1	Calcium-transporting ATPase type 2C member 1	104.78	19.8	15	24	14	13	22
P00829	ATP5B	ATP synthase subunit beta,	56.283	71.6	26	158	38.6	16	99
P05631	ATP5C1	ATP synthase subunit gamma,	33.072	42.3	13	31	36.2	12	51
P05630	ATP5D	ATP synthase subunit delta,	17.612	13.7	2	6	13.7	2	4
P13619	ATP5F1	ATP synthase F(0) complex subunit B1, mitochondrial	28.821	26.2	8	14	17.2	6	17
P32876	ATP5G1	ATP synthase F(0) complex subunit C1, mitochondrial	14.222	#N/A	#N/A	#N/A	5.1	1	8
P13620	ATP5H	ATP synthase subunit d,	18.692	38.5	7	26	28.6	6	11
Q00361	ATP5I	ATP synthase subunit e,	8.3206	42.3	3	4	42.3	3	10
Q28851	ATP5J2	ATP synthase subunit f,	10.297	27.3	2	7	#N/A	#N/A	#N/A
Q28852	ATP5L	ATP synthase subunit g,	11.417	29.1	3	6	#N/A	#N/A	#N/A
P13621	ATP5O	ATP synthase subunit O,	23.319	53.5	11	44	16.9	4	18
P22027	ATP5S	ATP synthase subunit s,	23.293	8.5	2	2	#N/A	#N/A	#N/A
P40682	ATP6AP1	V-type proton ATPase subunit S1	51.781	3.2	2	3	#N/A	#N/A	#N/A
P81134	ATP6AP2	Renin receptor	39.491	6.8	3	3	6.8	3	5
O97681	ATP6V0A2	V-type proton ATPase 116 kDa subunit a isoform 2	98.009	5.3	4	5	1.3	1	1
E1BGJ7	ATP6V0A4	Uncharacterized protein	95.748	3	2	2	#N/A	#N/A	#N/A
P61420	ATP6V0D1	V-type proton ATPase subunit d 1	40.329	36.5	11	23	19.7	8	17
Q2KJB6	ATP6V0D2	V-type proton ATPase subunit d 2	40.497	4.3	1	1	#N/A	#N/A	#N/A
P31404	ATP6V1A	V-type proton ATPase catalytic	68.343	47.3	25	67	18.5	10	23
P31408	ATP6V1B2	V-type proton ATPase subunit B, brain isoform	56.576	49.3	18	50	19.4	9	23
P21282	ATP6V1C1	V-type proton ATPase subunit C 1	43.985	53.7	21	46	30.4	15	36
F1N270	ATP6V1D	V-type proton ATPase subunit D	28.248	#N/A	#N/A	#N/A	11.3	3	3
P11019	ATP6V1E1	V-type proton ATPase subunit E 1	26.139	43.8	12	18	21.2	6	7

				Biological Replicate 1			Biological Replicate 2		
Accession number	Gene symbol	Discription	MW(kDa)	Sequence coverage(%)	Unique peptides	MS/MS count	Sequence coverage(%)	Unique peptides	MS/MS count
E1BN98	ATP8A1	Probable phospholipid-transporting ATPase IA	130.34	8.6	9	10	6.5	7	8
F1MG81	ATPAF1	Uncharacterized protein	36.009	18.6	6	12	#N/A	#N/A	#N/A
P01096	ATPIF1	ATPase inhibitor, mitochondrial	12.301	15.6	2	3	16.5	4	11
Q8MJ16	ATRN	Attractin	157.45	1.1	2	2	#N/A	#N/A	#N/A
Q2TBW0	ATXN10	Ataxin-10	53.102	19.8	8	13	4.4	2	3
E1BJ59	ATXN2L	Uncharacterized protein	113.36	2.1	3	3	2.6	3	3
F1MGJ7	AUH	Uncharacterized protein	31.284	6.6	3	3	#N/A	#N/A	#N/A
Q2TA06	AURKA	Aurora kinase A	45.462	57.2	21	83	39.1	14	54
E1BMD5	AVEN	Uncharacterized protein	37.862	#N/A	#N/A	#N/A	3.1	1	1
F1MMN9	AVIL	Uncharacterized protein	91.569	18.9	14	21	12.7	10	28
F1N025	AVL9	Uncharacterized protein (Fragment)	68.367	7	4	6	3.9	2	2
F1MKA0	B3GALTL	Uncharacterized protein (Fragment)	46.685	20.4	9	15	5.6	3	6
Q3SZB0	B3GAT3	Beta-1,3-glucuronyltransferase 3 (Glucuronosyltransferase I)	37.051	9	3	6	#N/A	#N/A	#N/A
Q5EA01	B3GNT1	N-acetyllactosaminide beta-1,3-N-acetylglucosaminyltransferase	47.231	9.9	4	5	9.4	4	5
A4IFK5	B3GNT3	B3GNT3 protein	42.679	8.6	3	8	9.1	3	3
F1MSS4	B3GNT4	Uncharacterized protein (Fragment)	40.885	14.4	6	11	3	1	1
Q32LF7	B4GALT4	UDP-Gal:betaGlcNAc beta 1,4-galactosyltransferase, polypeptide	39.46	20.8	6	13	11.1	4	8
Q08E57	BABAM1	BRISC and BRCA1-A complex member 1	36.886	6	2	3	3.3	1	2
Q3ZBG5	BAG2	BAG family molecular chaperone	23.649	6.6	2	2	8.5	2	3
F1MY28	BAG6	Uncharacterized protein	118.75	13.8	12	20	5.8	6	10
Q5EAD0	BAIAP2	Brain-specific angiogenesis inhibitor 1-associated protein 2	57.565	2.7	1	2	#N/A	#N/A	#N/A
F1MXR0	BAIAP2L1	Uncharacterized protein (Fragment)	55.301	7.3	4	4	#N/A	#N/A	#N/A
F1MAV4	BARD1	Uncharacterized protein	85.728	1.2	1	4	1.2	1	2
P80724	BASP1	Brain acid soluble protein 1	23.01	#N/A	#N/A	#N/A	32.2	6	13
O02703	BAX	Apoptosis regulator BAX	21.259	5.7	1	1	#N/A	#N/A	#N/A
Q32KL9	BCAP29	B-cell receptor-associated protein	28.212	15.4	4	5	8.3	2	2
Q5E9F1	BCAP31	B-cell receptor-associated protein	27.901	44.9	15	47	38.4	16	68

				Biological Replicate 1			Biological Replicate 2		
Accession number	Gene symbol	Discription	MW(kDa)	Sequence coverage(%)	Unique peptides	MS/MS count	Sequence coverage(%)	Unique peptides	MS/MS count
F1N6R8	BCAS3	Uncharacterized protein	78.693	17.7	11	18	5.9	4	6
A4IFQ7	BCAT1	Branched-chain-amino-acid	43.185	9.1	4	7	#N/A	#N/A	#N/A
Q5EA40	BCAT2	Branched-chain-amino-acid aminotransferase, mitochondrial	44.648	11.5	4	8	7.4	3	4
Q2NL37	BCCIP	BRCA2 and CDKN1A-interacting	35.183	2.6	1	1	#N/A	#N/A	#N/A
P11178	BCKDHA	2-oxoisovalerate dehydrogenase subunit alpha, mitochondrial	51.678	9.5	4	4	4.6	2	2
P21839	BCKDHB	2-oxoisovalerate dehydrogenase subunit beta, mitochondrial	42.935	13.8	3	3	#N/A	#N/A	#N/A
F1MNK0	BCL2L13	Uncharacterized protein	30.347	22.9	6	7	#N/A	#N/A	#N/A
Q5E9L4	BCL2L14	Apoptosis facilitator Bcl-2-like protein 14	36.838	#N/A	#N/A	#N/A	4.3	2	2
Q0II48	BCL2L15	Bcl-2-like protein 15	17.717	34.6	4	8	15.4	2	3
Q5E9H5	BCS1L	Mitochondrial chaperone BCS1	47.504	37.7	14	21	13.4	5	11
Q2TBV0	BEX2	Protein BEX2	15.236	7.8	1	1	#N/A	#N/A	#N/A
Q08DY1	BIN3	Bridging integrator 3	29.681	37.9	10	14	6.7	2	3
E1BL29	BLMH	Uncharacterized protein	52.93	36.8	16	38	19.4	9	23
A5D7K0	BLVRA	BLVRA protein	33.643	16.2	4	11	7.4	2	4
P52556	BLVRB	Flavin reductase (NADPH)	22.132	#N/A	#N/A	#N/A	3.9	1	2
E1BEV7	BMP1	Uncharacterized protein	110.77	#N/A	#N/A	#N/A	6.1	1	2
Q6PX77	BMP15	Bone morphogenetic protein 15	45.025	27.2	10	30	18.3	7	20
F1MKS4	BMP6	Uncharacterized protein	54.372	#N/A	#N/A	#N/A	3	1	2
F1N353	BNIP1	Uncharacterized protein	26.141	8.3	2	2	15.4	4	8
F1MYN0	BOLA2B	Uncharacterized protein (Fragment)	10.344	47.2	3	3	#N/A	#N/A	#N/A
F1MJU5	BOLADRB3	Uncharacterized protein	30.554	9	2	2	12.4	3	4
F1MX69	BPGM	Uncharacterized protein	30.07	58.3	16	72	41.7	11	58
Q3ZCK3	BPNT1	3(2),5-bisphosphate nucleotidase 1	33.328	38.6	10	28	19.8	6	23
A6H716	BRAP	BRAP protein	67.21	3.9	2	2	1.5	1	1
G3X874	BRCC3	Lys-63-specific deubiquitinase	33.265	4.5	1	1	#N/A	#N/A	#N/A
A6QQW8	BRE	BRCA1-A complex subunit BRE	43.56	6.3	2	2	#N/A	#N/A	#N/A
A5PJF5	BRI3BP	BRI3BP protein	26.937	#N/A	#N/A	#N/A	4.5	1	2
A6QLS8	BRWD2	BRWD2 protein	136.06	5.3	5	8	2.7	3	4

				Biological Replicate 1			Biological Replicate 2		
Accession number	Gene symbol	Discription	MW(kDa)	Sequence coverage(%)	Unique peptides	MS/MS count	Sequence coverage(%)	Unique peptides	MS/MS count
Q5E9P6	BSCL2	Seipin	44.016	8.4	3	3	7.4	3	3
Q3SX22	BSDC1	BSD domain-containing protein 1	50.342	#N/A	#N/A	#N/A	5	2	2
Q3ZBX0	BSG	Basigin	29.772	21.4	4	8	11.4	3	8
F1MKU3	BSPRY	Uncharacterized protein (Fragment)	49.698	14.7	7	11	8.8	4	8
Q0VCV8	BTBD10	BTB (POZ) domain containing 10	54.432	4.8	2	2	#N/A	#N/A	#N/A
Q56JY8	BTF3	Btf3 protein	17.713	35.2	5	6	#N/A	#N/A	#N/A
E1BMC2	BTG4	Uncharacterized protein	26.804	#N/A	#N/A	#N/A	8.3	2	2
F1MWP1	BUB1B	Uncharacterized protein	120.87	20.4	17	23	0.8	1	1
Q1JQB2	BUB3	Mitotic checkpoint protein BUB3	36.954	32.5	10	12	14.1	5	7
F1MZK4	BZW1	Uncharacterized protein	51.419	8.8	3	5	8	4	5
Q3T018	C10H14ORF1	Chromosome 14 open reading frame 1 ortholog	15.759	16.4	2	3	5.7	1	1
Q3T0S7	C10H14ORF166	Chromosome 14 open reading frame 166 ortholog	28.209	44.5	10	17	23.7	6	9
A7YWQ2	C10H15orf24	Chromosome 15 open reading frame 24 ortholog	26.502	16.2	3	4	#N/A	#N/A	#N/A
F1MSE5	C10orf76	Uncharacterized protein	67.504	#N/A	#N/A	#N/A	1.5	1	2
A1A4K7	C13H20ORF108	Chromosome 20 open reading frame 108 ortholog	20.447	#N/A	#N/A	#N/A	5.2	1	2
Q2HJH8	C15H11orf70	Uncharacterized protein C11orf70	30.691	29.6	6	6	#N/A	#N/A	#N/A
G3MX25	C15orf61	Uncharacterized protein (Fragment)	18.062	22.2	3	4	#N/A	#N/A	#N/A
F1MPA1	C16orf62	Uncharacterized protein	109.47	10.3	9	12	10	10	13
F1MQB5	C16orf70	Uncharacterized protein	47.592	6.9	2	3	4.3	2	2
E1BCP5	C17orf75	Uncharacterized protein	44.314	4	1	1	#N/A	#N/A	#N/A
Q3SZM3	C19H17orf62	Uncharacterized protein C17orf62	20.905	20.3	5	7	#N/A	#N/A	#N/A
E1B9I1	C19orf70	Uncharacterized protein	13.317	89.8	9	17	19.5	3	8
Q0VC84	C1GALT1	Glycoprotein-N-acetylgalactosamine 3-beta-galactosyltransferase 1	43.04	3.5	1	2	#N/A	#N/A	#N/A
Q3SX46	C1GALT1C1	C1GALT1-specific chaperone 1	36.441	#N/A	#N/A	#N/A	5	2	3
F1N582	C1orf27	Uncharacterized protein	50.98	11.7	4	5	4.6	2	4
Q3T0B6	C1QBP	Complement component 1 Q subcomponent-binding protein,	30.606	30.6	5	33	15.8	3	5

				Biological Replicate 1			Biological Replicate 2		
Accession number	Gene symbol	Discription	MW(kDa)	Sequence coverage(%)	Unique peptides	MS/MS count	Sequence coverage(%)	Unique peptides	MS/MS count
Q0VCX1	C1S	Complement C1s subcomponent	76.608	2.3	1	1	#N/A	#N/A	#N/A
Q3ZBP8	C21H15orf40	UPF0235 protein C15orf40 homolog	13.417	10.3	1	1	#N/A	#N/A	#N/A
Q32KX8	C25H16orf13	UPF0585 protein C16orf13 homolog	22.714	26	5	10	19.1	4	5
A6H794	C28H10ORF35	C28H10ORF35 protein	13.144	20.8	2	2	#N/A	#N/A	#N/A
A4IFA8	C29H11orf68	UPF0696 protein C11orf68 homolog	27.398	8	2	2	7.2	2	3
A5PK60	C2H2orf33	C2H2orf33 protein	29.7	#N/A	#N/A	#N/A	7.6	3	5
A3KN05	C2H2orf47	Uncharacterized protein C2orf47 homolog, mitochondrial	32.94	3.4	1	2	8.6	3	4
Q32KX9	C2H2orf76	UPF0538 protein C2orf76 homolog	14.666	20.6	2	2	#N/A	#N/A	#N/A
E1BDF4	C2orf44	Uncharacterized protein	74.293	2.7	2	2	#N/A	#N/A	#N/A
Q17Q88	C3H1ORF41	Chromosome 1 open reading frame 41 ortholog	16.399	11.9	1	1	#N/A	#N/A	#N/A
F1MZT6	C3H1orf50	Uncharacterized protein	22.098	15.9	2	5	#N/A	#N/A	#N/A
Q0P5L7	C3H1orf85	Lysosomal protein NCU-G1	43.53	8.7	2	3	#N/A	#N/A	#N/A
E1B722	C3orf33	Uncharacterized protein	34.127	14.6	4	5	9.5	3	5
Q1LZE8	C4H7orf25	UPF0415 protein C7orf25 homolog	46.427	2.1	1	1	#N/A	#N/A	#N/A
E1BMH3	C4orf33	Uncharacterized protein	23.181	29.1	5	7	#N/A	#N/A	#N/A
Q29RU4	C6	Complement component C6	104.54	1	1	1	1.9	2	3
P62248	C7H19orf10	UPF0556 protein C19orf10 homolog	19.03	30.5	5	21	15.5	3	5
G3N3L6	C7H19orf52	Uncharacterized protein	28.909	12.7	4	4	#N/A	#N/A	#N/A
Q32L09	C7H19orf66	UPF0515 protein C19orf66 homolog	33.205	12.4	3	4	16.6	5	9
A8YXZ2	C8G	C8G protein	25.288	#N/A	#N/A	#N/A	3.8	1	2
A2VDY4	C8H4orf27	UPF0609 protein C4orf27 homolog	39.09	7.8	3	7	#N/A	#N/A	#N/A
Q1JP73	C8H9orf64	UPF0553 protein C9orf64 homolog	39.036	44.6	15	32	15.5	6	11
F1N1V6	C9H6orf120	Uncharacterized protein	20.202	34.2	2	3	#N/A	#N/A	#N/A
E1BJC0	C9orf41	Uncharacterized protein	47.532	2.9	1	2	#N/A	#N/A	#N/A
F1N0H3	CA2	Carbonic anhydrase 2 (Fragment)	27.75	11.2	3	8	#N/A	#N/A	#N/A
E1BAD9	CA5A	Uncharacterized protein	34.906	4.8	1	2	#N/A	#N/A	#N/A
Q29RI6	CAB39	Calcium-binding protein 39	39.869	43.1	13	22	11.7	4	10
F1MMV6	CAB39L	Uncharacterized protein (Fragment)	39.05	9.5	2	4	#N/A	#N/A	#N/A
Q3T168	CACYBP	Calcyclin-binding protein	26.332	45.2	9	12	13.9	4	7
F1MKZ3	CADPS	Uncharacterized protein (Fragment)	137.65	4.4	2	3	#N/A	#N/A	#N/A

				Biological Replicate 1			Biological Replicate 2		
Accession number	Gene symbol	Discription	MW(kDa)	Sequence coverage(%)	Unique peptides	MS/MS count	Sequence coverage(%)	Unique peptides	MS/MS count
A7MB25	CADPS2	CADPS2 protein	143.62	9	6	15	2.7	4	7
F1MJW8	CALB2	Calretinin	31.35	46.1	12	29	26.2	7	11
Q2KJ21	CALCOCO1	Calcium-binding and coiled-coil domain-containing protein 1	75.66	#N/A	#N/A	#N/A	1.5	1	1
P62157	CALM	Calmodulin	16.837	50.3	5	11	26.8	4	8
P52193	CALR	Calreticulin	48.038	51.3	26	196	32.4	16	109
Q08DQ1	CAMK1	Calcium/calmodulin-dependent protein kinase I	41.398	10.8	4	8	4.3	1	2
F1MG86	CAMK2B	Uncharacterized protein (Fragment)	53.592	21.1	5	7	13.1	4	17
A5D9F0	CAMK2D	Calcium/calmodulin-dependent protein kinase II delta	54.113	20.7	5	12	10.7	3	6
E1BPZ9	CAMSAP3	Uncharacterized protein	134.88	#N/A	#N/A	#N/A	0.7	1	1
A7MBJ5	CAND1	Cullin-associated NEDD8-dissociated protein 1	136.37	56.9	60	272	33.3	37	199
E1BNE2	CAND2	Uncharacterized protein	134.57	24.3	19	34	8.2	6	14
E1BGL5	CANT1	Uncharacterized protein	44.705	8	2	3	9	4	7
A7Z066	canx	Canx protein	67.775	39	30	105	21.1	14	49
Q27970	CAPN1	Calpain-1 catalytic subunit	82.206	21.8	14	24	8	5	9
E1BIX3	CAPN7	Uncharacterized protein	92.556	5	3	6	1.4	1	3
P13135	CAPNS1	Calpain small subunit 1	27.931	9.1	3	7	9.1	3	6
F1MCP8	CAPRIN2	Uncharacterized protein (Fragment)	121.48	11	11	14	9	10	22
A4FUA8	CAPZA1	F-actin-capping protein subunit	32.931	70.6	10	35	22.7	4	13
Q5E997	CAPZA2	F-actin-capping protein subunit	32.979	51.4	7	15	17.1	3	3
F1MW90	CARD10	Uncharacterized protein	115.62	1.1	1	1	#N/A	#N/A	#N/A
Q2NKU4	CARHSP1	Calcium regulated heat stable protein 1, 24kDa	15.891	38.8	6	18	12.9	2	3
E1BNQ4	CARKD	ATP-dependent (S)-NAD(P)H-hydrate dehydratase	34.809	41.6	12	39	28.6	9	27
F1MBG0	CARM1	Uncharacterized protein (Fragment)	58.762	8.2	4	4	#N/A	#N/A	#N/A
A4FUC8	CARS	CARS protein	94.132	16.3	13	17	11.3	9	18
Q2KIF8	CARS2	Cysteine--tRNA ligase,	61.198	40.4	20	52	30.8	14	41
A5D7H5	CASC3	Protein CASC3	76.146	1.3	1	2	1.6	1	1

				Biological Replicate 1			Biological Replicate 2		
Accession number	Gene symbol	Discription	MW(kDa)	Sequence coverage(%)	Unique peptides	MS/MS count	Sequence coverage(%)	Unique peptides	MS/MS count
Q08DY9	CASP3	Caspase-3	31.135	6.9	2	2	4	1	2
Q3T0P5	CASP6	Caspase-6	33.35	16	5	12	#N/A	#N/A	#N/A
P00432	CAT	Catalase	59.915	12.5	5	6	10.6	6	8
P79132	CAV1	Caveolin-1	20.594	#N/A	#N/A	#N/A	5.6	1	1
E1BJS3	CBL	Uncharacterized protein (Fragment)	99.598	13.1	9	12	1.9	2	2
Q3SZD7	CBR1	Carbonyl reductase [NADPH] 1	30.533	45.8	7	19	7.9	1	2
Q0VC97	CBR3	Carbonyl reductase 3	30.762	14.1	2	5	16.6	3	12
F1MEW4	CBS	Uncharacterized protein	62.867	18.2	11	17	8.9	5	11
E1BFK3	CBWD2	Uncharacterized protein	44.636	30.2	10	13	6.3	3	4
G5E6N7	CBX1	Uncharacterized protein	21.418	13	2	2	#N/A	#N/A	#N/A
A5PKI6	CC2D1A	CC2D1A protein	103.93	10.8	8	10	3.7	3	4
F1N7R5	CC2D1B	Uncharacterized protein	93.4	3.3	3	4	#N/A	#N/A	#N/A
E1BJ81	CCAR1	Uncharacterized protein (Fragment)	58.242	1.7	1	1	#N/A	#N/A	#N/A
E1B9H3	CCAR2	Uncharacterized protein	101.93	5.5	4	6	3.9	4	6
F1MW71	CCBL1	Uncharacterized protein (Fragment)	40.348	4.8	1	2	4.8	1	2
Q0P5G4	CCBL2	Kynurenine--oxoglutarate	51.471	24.8	8	15	11.4	4	8
Q3ZBZ7	CCDC109B	Coiled-coil domain containing 109B	39.024	12.5	4	4	#N/A	#N/A	#N/A
Q2TBV6	CCDC124	Coiled-coil domain-containing	26.005	7.6	2	3	12.1	3	6
F1ML08	CCDC132	Uncharacterized protein	111.15	11	9	13	2.2	2	4
E1BFW1	CCDC134	Uncharacterized protein	26.375	25.3	4	5	19.2	3	8
G3MWY8	CCDC167	Uncharacterized protein	9.7784	#N/A	#N/A	#N/A	9.5	1	2
Q1RMI8	CCDC22	Coiled-coil domain-containing	67.041	34.3	16	40	13.1	7	16
Q3SZX8	CCDC25	Coiled-coil domain-containing	24.618	3.8	1	1	#N/A	#N/A	#N/A
Q05B58	CCDC53	WASH complex subunit CCDC53	21.345	20.6	4	7	11.3	3	5
A4FUI1	CCDC58	Coiled-coil domain-containing	16.708	#N/A	#N/A	#N/A	18.1	2	2
F1N091	CCDC6	Uncharacterized protein (Fragment)	43.105	36.7	11	23	12.1	5	12
F1MAY4	CCDC88A	Uncharacterized protein	216.58	#N/A	#N/A	#N/A	3.9	9	11
E1B6Y1	CCDC90A	Uncharacterized protein	38.687	12.7	7	9	12.7	6	11
A6QPA1	CCDC90B	CCDC90B protein	28.675	29.8	7	13	15.3	5	14
F1MDJ4	CCDC93	Uncharacterized protein	73.061	13.9	8	10	7.6	6	10
Q1LZG6	CCNB1	G2/mitotic-specific cyclin-B1	47.657	3.7	2	2	#N/A	#N/A	#N/A
E1BC90	CCNI2	Uncharacterized protein	40.46	55.1	17	65	29.6	9	24

				Biological Replicate 1			Biological Replicate 2		
Accession number	Gene symbol	Discription	MW(kDa)	Sequence coverage(%)	Unique peptides	MS/MS count	Sequence coverage(%)	Unique peptides	MS/MS count
E1BBI6	CCNYL1	Uncharacterized protein	41.127	6.9	2	2	4.7	2	4
A2VE74	CCRN4L	CCR4 carbon catabolite repression 4-like (S. cerevisiae)	47.923	7.5	2	2	1.9	1	2
E1BE86	CCS	Uncharacterized protein	29.185	9.1	3	4	5.5	2	3
Q3ZBH0	CCT2	T-complex protein 1 subunit beta	57.475	72	35	125	40.7	21	62
Q3T0K2	CCT3	T-complex protein 1 subunit gamma	60.585	67	41	117	38.5	26	89
F1MWD3	CCT5	Uncharacterized protein	59.614	67.7	36	145	33.8	23	85
Q3MHL7	CCT6A	T-complex protein 1 subunit zeta	57.956	49.7	25	82	37.9	21	92
F1MWR8	CCT7	T-complex protein 1 subunit eta	59.18	54.2	27	92	43.8	21	98
Q3ZCI9	CCT8	T-complex protein 1 subunit theta	59.609	77.9	39	112	55.3	28	120
Q0VD30	CCZ1	Vacuolar fusion protein CCZ1	55.501	10.4	5	6	#N/A	#N/A	#N/A
Q3ZBH3	CD151	CD151 antigen	27.986	4	1	1	#N/A	#N/A	#N/A
Q3ZC60	CD200	CD200 molecule	30.093	3.7	1	2	3.7	1	2
F1N2H6	CD276	Uncharacterized protein (Fragment)	56.477	#N/A	#N/A	#N/A	3.8	1	1
F1MM14	CD2AP	Uncharacterized protein	71.203	2.2	1	1	#N/A	#N/A	#N/A
A6QNY1	CD320	CD320 antigen	26.934	5.9	3	6	3.1	2	3
Q28203	CD40	Tumor necrosis factor receptor superfamily member 5	31.061	3.2	1	1	#N/A	#N/A	#N/A
F1N4W5	CD46	Membrane cofactor protein	39.943	3.5	1	2	3.5	1	3
Q45VK8	CD55	DAF-2	40.466	3.8	3	5	3.5	1	4
Q9XSK2	CD63	CD63 antigen	25.752	8.9	3	3	#N/A	#N/A	#N/A
G3MYH4	CD81	CD81 antigen (Fragment)	23.226	9.3	1	5	9.3	1	3
G8JKX6	CD9	CD9 antigen (Fragment)	22.665	3.9	1	3	3.9	1	3
Q8SQA4	CD97	CD97 antigen	80.321	#N/A	#N/A	#N/A	2.9	2	5
Q2YDG3	CDC123	Cell division cycle protein 123	38.922	8.4	3	3	4.2	2	2
Q5EAC6	CDC37	Hsp90 co-chaperone Cdc37	44.58	38.7	17	48	23.9	9	34
Q2KJ93	CDC42	Cell division control protein 42	21.258	48.2	7	15	31.4	4	18
F1N619	CDH1	Cadherin-1 (Fragment)	91.858	2.7	2	3	2.1	2	2
E1BGT1	CDH3	Cadherin-3	90.99	8.2	4	5	3	2	3
Q3T103	CDIPT	CDP-diacylglycerol--inositol 3-phosphatidyltransferase (Phosphatidylinositol synthase)	23.597	13.1	3	4	8	2	5

				Biological Replicate 1			Biological Replicate 2		
Accession number	Gene symbol	Discription	MW(kDa)	Sequence coverage(%)	Unique peptides	MS/MS count	Sequence coverage(%)	Unique peptides	MS/MS count
P48734	CDK1	Cyclin-dependent kinase 1	34.025	67.3	16	71	34	9	24
Q02399	CDK5	Cyclin-dependent kinase 5	33.288	71.9	19	77	38.7	12	50
F1MM23	CDK5RAP3	Uncharacterized protein	56.915	7.5	5	6	3.4	2	4
Q08DX5	CDK7	Cyclin-dependent kinase 7	38.946	5.5	1	2	4.9	2	3
F1MAW5	CDKAL1	Uncharacterized protein	65.481	6.4	3	3	#N/A	#N/A	#N/A
Q3SZU4	CDO1	Cysteine dioxygenase type 1	23.013	29	6	15	11.5	3	6
G3MZU2	CDS1	Phosphatidate cytidylyltransferase	53.128	#N/A	#N/A	#N/A	3.7	3	5
A0JNC1	CDS2	Phosphatidate cytidylyltransferase	51.356	#N/A	#N/A	#N/A	1.8	1	3
F1MGI9	CECR5	Uncharacterized protein	42.565	8.7	2	6	7.7	3	5
Q2TBH1	CENPM	Centromere protein M	19.635	6.7	1	1	#N/A	#N/A	#N/A
Q3ZBK8	CENPO	Centromere protein O	33.892	7.4	1	1	#N/A	#N/A	#N/A
G3N1U1	CENPV	Uncharacterized protein	28.839	4.2	1	1	#N/A	#N/A	#N/A
Q2HJG1	CEP27	Centrosomal protein 27kDa	26.688	#N/A	#N/A	#N/A	8.1	2	5
E1B8M0	CEP55	Uncharacterized protein	54.333	2.8	1	1	#N/A	#N/A	#N/A
F1N1V2	CEPT1	Uncharacterized protein	46.45	2.6	1	1	#N/A	#N/A	#N/A
Q3ZBF8	CERS2	Ceramide synthase 2	44.903	6.6	2	3	2.4	1	1
Q5E9R6	CERS4	Ceramide synthase 4	46.363	12.5	4	10	2.8	1	1
Q2TBN3	CETN2	Centrin-2	19.809	17.4	2	3	#N/A	#N/A	#N/A
F1N547	CETN3	Uncharacterized protein	19.563	7.2	1	1	13.2	3	3
Q6B857	CFAP20	UPF0468 protein C16orf80 homolog	22.748	29	5	10	15	3	6
Q5E9F7	CFL1	Cofilin-1	18.518	72.3	10	43	36.1	5	21
Q148F1	CFL2	Cofilin-2	18.736	66.9	8	23	61.4	7	22
F1MYN2	CGN	Uncharacterized protein	136.09	7	9	12	2.8	4	6
A6QLA6	CHAF1A	Chromatin assembly factor 1 subunit	107.26	#N/A	#N/A	#N/A	2	2	2
A5D9H4	CHAF1B	Chromatin assembly factor 1 subunit	62.768	1.9	1	1	1.9	1	1
Q5E9D3	CHCHD3	Coiled-coil-helix-coiled-coil-helix domain-containing protein 3,	26.1	34.4	10	26	31.3	10	30
Q32L35	CHCHD6	Coiled-coil-helix-coiled-coil-helix domain-containing protein 6,	26.439	22.5	6	12	24.2	6	12
E1BES2	CHDH	Choline dehydrogenase	65.517	8.1	5	6	4.4	3	11
A5D7V1	CHEK1	CHEK1 protein	54.47	2.3	1	1	#N/A	#N/A	#N/A
Q5EAB4	CHID1	Chitinase domain-containing protein	44.562	21.1	8	19	11.5	5	12

				Biological Replicate 1			Biological Replicate 2		
Accession number	Gene symbol	Discription	MW(kDa)	Sequence coverage(%)	Unique peptides	MS/MS count	Sequence coverage(%)	Unique peptides	MS/MS count
G3X782	CHKB	Uncharacterized protein	45.186	40.5	13	29	12.7	4	8
F1MW33	CHL1	Uncharacterized protein (Fragment)	133.59	3.7	3	3	3.8	5	7
F1MS24	CHM	Uncharacterized protein (Fragment)	69.173	2.9	2	4	#N/A	#N/A	#N/A
Q32KR9	CHMP1A	Chromatin modifying protein 1A	21.712	20.9	6	8	24.5	7	14
Q5E994	CHMP1B	Charged multivesicular body protein	22.022	12.1	3	4	4.5	1	2
Q3T0U5	CHMP2A	Chromatin modifying protein 2A	25.132	24.3	8	16	7.7	3	9
Q3SX42	CHMP2B	Charged multivesicular body protein	23.95	19.7	5	12	16.4	4	6
Q58CS7	CHMP3	Charged multivesicular body protein	25.017	12.2	3	7	17.1	5	11
Q08E32	CHMP4B	CHMP4B protein	24.936	#N/A	#N/A	#N/A	13.8	3	8
F1MZV2	CHMP5	Uncharacterized protein	24.589	10.5	2	4	14.6	2	3
Q148L0	CHMP6	Chromatin modifying protein 6	23.507	7.5	2	2	3.5	1	2
A6H704	CHMP7	CHMP7 protein	50.644	12.2	3	3	9.3	5	7
Q29RL2	CHORDC1	Cysteine and histidine-rich domain-containing protein 1	37.426	47.6	12	20	16.6	5	8
Q3SYS6	CHP1	Calcineurin B homologous protein 1	22.442	39	7	14	24.6	4	6
F1MG93	CHRD	Chordin (Fragment)	99.785	#N/A	#N/A	#N/A	2	2	2
F6RFX1	CHST12	Uncharacterized protein	48.496	11.8	4	4	9.1	3	3
E1BK55	CHST6	Uncharacterized protein	44.263	5.6	3	6	5.6	3	3
Q32PJ6	CIAO1	Probable cytosolic iron-sulfur protein assembly protein CIAO1	37.773	10.6	3	6	2.4	1	1
Q5EAC7	CIAPIN1	Anamorsin	33.204	44.8	12	30	6.1	2	3
E1BIR5	CILP	Uncharacterized protein	128.27	4.4	5	7	1.9	2	4
Q3ZBU2	CISD1	CDGSH iron-sulfur domain-containing protein 1	11.983	44.3	4	17	45.3	5	18
Q05B71	CISD2	CDGSH iron-sulfur domain-containing protein 2	15.278	55.6	9	25	45.9	6	22
G3MWJ2	CISD3	Uncharacterized protein (Fragment)	14.941	#N/A	#N/A	#N/A	23.7	4	7
F1ME65	CKAP4	Uncharacterized protein	64.529	8	5	7	5.8	3	4
E1B7K5	CKAP5	Uncharacterized protein	225.12	16.2	28	40	7.4	16	33
Q5EA61	CKB	Creatine kinase B-type	42.719	78.5	23	209	47.8	15	82
Q9TTK8	CKMT1	Creatine kinase U-type,	46.896	62	22	184	40.9	17	219

				Biological Replicate 1			Biological Replicate 2		
Accession number	Gene symbol	Discription	MW(kDa)	Sequence coverage(%)	Unique peptides	MS/MS count	Sequence coverage(%)	Unique peptides	MS/MS count
Q2KJI1	CKS2	Cyclin-dependent kinases regulatory subunit 2	9.8602	11.4	1	1	11.4	1	1
F1MFY6	CL43	Collectin-43	24.921	#N/A	#N/A	#N/A	8.2	2	3
E1BQ15	CLASP2	Uncharacterized protein	168.18	5.8	7	7	4.6	6	9
Q1LZF8	CLCC1	Chloride channel CLIC-like protein	61.811	11.8	6	8	5.7	3	6
Q4F894	CLCN4	Chloride channel 4	84.892	7.8	4	7	5.8	4	7
F1MN45	CLCN5	Uncharacterized protein (Fragment)	90.782	3.9	2	2	2.2	1	1
Q4PKH3	CLCN7	H(+)/Cl(-) exchange transporter 7	88.83	1.5	1	1	#N/A	#N/A	#N/A
Q3SYT6	CLGN	Calmegin	69.514	20.1	9	27	5.3	3	16
Q5E9B7	CLIC1	Chloride intracellular channel	26.992	39.8	7	9	13.7	3	4
Q9XSA7	CLIC4	Chloride intracellular channel	28.727	66.8	13	53	36	8	25
A7Z035	CLINT1	Clathrin interactor 1	70.521	13.4	7	8	4.2	2	4
E3W9A2	CLIP1	Uncharacterized protein	160.62	5.7	7	8	8.2	13	16
E1BMT3	CLN6	Uncharacterized protein	35.784	10.6	2	3	6.4	1	2
Q1LZ90	CLNS1A	Chloride channel, nucleotide-	26.207	5.5	1	1	#N/A	#N/A	#N/A
Q5E9N5	CLPB	Caseinolytic peptidase B protein	75.439	1.9	1	1	#N/A	#N/A	#N/A
F1N779	CLPTM1	Cleft lip and palate transmembrane protein 1 homolog	65.497	8	3	4	4.5	2	4
F1N155	CLPX	Uncharacterized protein (Fragment)	66.825	17.5	9	12	9.4	5	6
P49951	CLTC	Clathrin heavy chain 1	191.59	61.6	101	440	39.8	64	355
E1BIA7	CLUH	Clustered mitochondria protein	147.14	2.6	3	3	2.4	3	4
A6QL68	CLYBL	CLYBL protein	37.549	2.6	1	2	8.5	3	5
Q2KIW9	CMPK1	UMP-CMP kinase	22.279	28.1	5	10	5.1	1	1
A4IFB9	CMTM4	CMTM4 protein	22.877	#N/A	#N/A	#N/A	9.1	2	6
F1N680	CMTM6	Uncharacterized protein	20.209	6	1	1	#N/A	#N/A	#N/A
A2VE39	CMTR1	Cap-specific mRNA (nucleoside-2-O-)-methyltransferase 1	95.141	15.7	13	19	9.1	9	15
Q3T0Q6	CNBP	Cellular nucleic acid-binding	18.742	25.3	3	6	12.9	2	3
F1MWP9	CNDP1	Uncharacterized protein	56.69	12.4	5	5	#N/A	#N/A	#N/A
Q3ZC84	CNDP2	Cytosolic non-specific dipeptidase	52.655	42.7	19	46	16	7	22
Q3T126	CNIH4	Protein cornichon homolog 4	16.049	14.4	1	3	#N/A	#N/A	#N/A
E1B9A8	CNKSR1	Uncharacterized protein	53.509	10.3	3	4	#N/A	#N/A	#N/A

				Biological Replicate 1			Biological Replicate 2		
Accession number	Gene symbol	Discription	MW(kDa)	Sequence coverage(%)	Unique peptides	MS/MS count	Sequence coverage(%)	Unique peptides	MS/MS count
Q32L92	CNN3	Calponin-3	36.357	38.9	11	36	19.8	6	18
F1MD84	CNNM1	Uncharacterized protein (Fragment)	103.2	1.3	1	8	#N/A	#N/A	#N/A
E1BIL3	CNNM2	Uncharacterized protein	94.346	2.9	1	2	#N/A	#N/A	#N/A
F1MK24	CNNM4	Uncharacterized protein (Fragment)	72.172	#N/A	#N/A	#N/A	1.4	1	1
F1MHU9	CNOT1	Uncharacterized protein	266.31	0.9	2	2	0.5	1	1
A4IFB6	CNOT10	CCR4-NOT transcription complex	81.983	7.7	6	8	5.7	5	6
E1BHL0	CNOT11	Uncharacterized protein	55.242	16.2	5	7	3.9	2	4
E1BCS1	CNOT3	Uncharacterized protein	80.786	2.4	2	5	3.5	3	7
F1MWA7	CNOT4	Uncharacterized protein	70.88	6.5	4	5	4	2	3
A6QR51	CNOT6	CNOT6 protein	63.277	1.8	1	1	#N/A	#N/A	#N/A
E1BE73	CNOT6L	Uncharacterized protein (Fragment)	47.802	2.4	1	1	#N/A	#N/A	#N/A
Q3ZC01	CNOT7	CCR4-NOT transcription complex	32.718	4.9	1	2	#N/A	#N/A	#N/A
E1BJH0	CNOT8	Uncharacterized protein	33.54	4.8	1	1	3.8	1	1
P06623	CNP	2,3-cyclic-nucleotide 3-	44.875	19	8	14	16.5	7	15
Q1LZ72	CNPY2	Canopy 2 homolog (Zebrafish)	20.668	14.3	2	3	5.5	1	3
Q3SWX1	CNPY4	Protein canopy homolog 4	27.368	4.2	1	1	#N/A	#N/A	#N/A
Q17QM9	CNRIP1	CB1 cannabinoid receptor-interacting protein 1	18.664	39	5	7	11.6	2	3
E1BC04	CNST	Uncharacterized protein	79.437	1.2	1	4	#N/A	#N/A	#N/A
Q3T0E3	COA3	Cytochrome c oxidase assembly protein 3 homolog, mitochondrial	11.707	17.9	2	4	8.5	1	3
E1BEL9	COASY	Uncharacterized protein	61.799	27.8	10	22	5.3	3	5
F1N290	COBLL1	Uncharacterized protein (Fragment)	52.228	5.2	2	3	#N/A	#N/A	#N/A
E1BK29	COG1	Uncharacterized protein	108.26	8.3	6	7	2.5	3	3
F1MQ89	COG2	Uncharacterized protein	83.423	7.3	4	8	3.4	3	5
E1BGA6	COG3	Uncharacterized protein	94.284	9.7	6	10	6.9	6	6
Q3MHG0	COG4	Conserved oligomeric Golgi complex subunit 4	88.855	8.3	5	7	4.1	4	4
F1N1T8	COG5	Conserved oligomeric Golgi complex subunit 5 (Fragment)	91.195	6.9	5	9	9.7	8	11
Q3SZI7	COG6	Conserved oligomeric Golgi complex subunit 6	73.301	13.4	7	9	2.9	2	5

				Biological Replicate 1			Biological Replicate 2		
Accession number	Gene symbol	Discription	MW(kDa)	Sequence coverage(%)	Unique peptides	MS/MS count	Sequence coverage(%)	Unique peptides	MS/MS count
Q58CW1	COQ7	COQ7 protein	20.246	20.7	4	6	9.5	2	6
Q2NL34	COQ9	Ubiquinone biosynthesis protein COQ9, mitochondrial	35.778	17.6	5	8	2.8	1	5
A6QLZ8	CORO6	Coronin	48.067	22.7	8	23	13.5	5	13
Q0V8F1	CORO7	Coronin-7	99.251	1.7	1	1	#N/A	#N/A	#N/A
F1N5P2	COX18	Uncharacterized protein	37.319	13	4	5	#N/A	#N/A	#N/A
Q6QTG5	COX3	Cytochrome c oxidase subunit 3	29.845	5.4	1	4	#N/A	#N/A	#N/A
P00423	COX4I1	Cytochrome c oxidase subunit 4 isoform 1, mitochondrial	19.571	37.9	7	33	29.6	6	17
P00426	COX5A	Cytochrome c oxidase subunit 5A, mitochondrial	16.735	21.7	4	8	26.3	5	11
P00428	COX5B	Cytochrome c oxidase subunit 5B, mitochondrial	13.834	18.6	3	6	27.9	4	11
P00429	COX6B1	Cytochrome c oxidase subunit 6B1	10.156	11.6	1	1	#N/A	#N/A	#N/A
P04038	COX6C	Cytochrome c oxidase subunit 6C	8.6101	20.3	2	2	20.3	2	6
Q3T061	COX7A2L	Cytochrome c oxidase subunit 7A-related protein, mitochondrial	12.543	#N/A	#N/A	#N/A	14	2	2
P00730	CPA1	Carboxypeptidase A1	47.081	26.7	11	25	19.8	9	40
F1MSH0	CPEB1	Uncharacterized protein (Fragment)	55.722	27.7	9	15	8.7	5	14
A7MBD9	CPM	CPM protein	50.318	5.9	2	6	1.6	1	3
Q08DB4	CPNE1	Copine I	58.922	7.1	3	6	#N/A	#N/A	#N/A
F1MLJ3	CPNE4	Uncharacterized protein	28.104	#N/A	#N/A	#N/A	3.5	1	1
A5D784	CPNE8	CPNE8 protein	63.086	3.5	2	2	#N/A	#N/A	#N/A
E1BKY9	CPOX	Uncharacterized protein	50.38	24.1	9	21	3.8	2	3
Q58DC0	CPPED1	Calcineurin-like phosphoesterase domain-containing protein 1	35.409	24.9	6	11	11.8	4	9
Q10568	CPSF2	Cleavage and polyadenylation specificity factor subunit 2	88.416	3.2	3	3	0.9	1	2
P79101	CPSF3	Cleavage and polyadenylation specificity factor subunit 3	77.487	2.8	2	2	2.9	2	2
Q0P5D2	CPSF6	Cleavage and polyadenylation specificity factor subunit 6	59.255	5.4	2	2	#N/A	#N/A	#N/A

				Biological Replicate 1			Biological Replicate 2		
Accession number	Gene symbol	Discription	MW(kDa)	Sequence coverage(%)	Unique peptides	MS/MS count	Sequence coverage(%)	Unique peptides	MS/MS count
A2VDR8	COG7	Conserved oligomeric Golgi complex subunit 7	86.526	10.8	7	8	4.8	4	4
Q2TBH9	COG8	Conserved oligomeric Golgi complex subunit 8	69.781	7.8	5	7	5.4	4	5
F1MSR8	COL2A1	Collagen alpha-1(II) chain	134.43	2.5	4	7	2.5	4	9
A4FUB5	COMMD10	COMMD10 protein	22.935	10.4	2	3	4	1	1
A7YWP6	COMMD2	COMMD2 protein	22.773	27.1	7	11	18.1	4	16
Q5E9K1	COMMD5	COMM domain-containing protein 5	24.502	23.2	5	7	5.4	1	1
Q3MHX1	COMMD7	COMM domain-containing protein 7	22.534	24.5	5	6	11.5	3	4
F1N7D3	COMMD8	Uncharacterized protein	21.128	15.3	4	6	16.9	4	6
Q2TBN5	COMMD9	COMM domain-containing protein 9	21.781	16.7	2	3	9.6	2	3
Q27954	COPA	Coatomer subunit alpha	138.36	54.9	59	138	25.7	34	89
A0JN39	COPB1	Coatomer subunit beta	107.13	47.1	38	81	19.9	15	30
P35605	COPB2	Coatomer subunit beta	102.39	49.1	36	75	22.3	19	39
Q28104	COPE	Coatomer subunit epsilon	34.481	22.7	8	15	10.4	3	4
P53620	COPG1	Coatomer subunit gamma-1	97.385	49.8	29	72	11.4	8	29
A2VE21	COPG2	Coatomer subunit gamma-2	97.564	11.9	6	7	6.7	4	5
G3X736	COPS2	Uncharacterized protein	52.404	31.6	13	25	10.9	5	15
A6H7B5	COPS3	COP9 signalosome complex subunit 3	47.799	24.1	8	15	9.9	4	8
Q3SZA0	COPS4	COP9 signalosome complex subunit 4	46.284	60.6	18	37	27.8	11	33
F1MBP8	COPS5	Uncharacterized protein	37.564	39.5	12	21	5.7	2	2
F1MG10	COPS6	COP9 signalosome complex subunit 6	35.966	53.1	13	22	14.2	5	9
Q2KI56	COPS7B	COP9 signalosome complex subunit 7b	29.595	21.2	5	12	#N/A	#N/A	#N/A
A4FV74	COPS8	COPS8 protein	23.201	42.6	5	12	20.1	3	6
P35604	COPZ1	Coatomer subunit zeta-1	20.228	21.5	4	11	19.2	3	5
F1MXE6	COPZ2	Uncharacterized protein (Fragment)	21.005	5.9	1	2	5.9	1	1
F1MK72	COQ10B	Uncharacterized protein	27.785	#N/A	#N/A	#N/A	3.3	1	1
Q3T131	COQ3	Hexaprenyldihydroxybenzoate methyltransferase, mitochondrial	41.49	#N/A	#N/A	#N/A	6.8	2	3
Q0P5A2	COQ5	2-methoxy-6-polyprenyl-1,4-benzoquinol methylase,	37.591	7.9	2	3	#N/A	#N/A	#N/A
Q2KIL4	COQ6	Ubiquinone biosynthesis	51.072	4.7	2	2	7.7	3	4

				Biological Replicate 1			Biological Replicate 2		
Accession number	Gene symbol	Discription	MW(kDa)	Sequence coverage(%)	Unique peptides	MS/MS count	Sequence coverage(%)	Unique peptides	MS/MS count
Q58DK1	CPT1B	Carnitine O-palmitoyltransferase 1, muscle isoform	88.511	#N/A	#N/A	#N/A	1.2	1	2
Q2KJB7	CPT2	Carnitine O-palmitoyltransferase 2, mitochondrial	74.482	13.4	7	11	3.5	3	3
Q0VCQ0	CPTP	Ceramide-1-phosphate transfer	24.574	8.9	2	3	#N/A	#N/A	#N/A
Q5PXY7	CRABP2	Cellular retinoic acid-binding	15.735	6.5	1	1	6.5	1	1
F1MX50	CREG1	Uncharacterized protein	23.958	26.2	4	8	11.3	2	9
Q5EA46	CRELD1	Cysteine-rich with EGF-like domain protein 1	45.368	#N/A	#N/A	#N/A	1.9	1	1
Q2KIT5	CRELD2	Cysteine-rich with EGF-like domain protein 2	38.229	#N/A	#N/A	#N/A	5.4	2	6
E1BQ32	CRK	Uncharacterized protein	33.83	28	7	13	8.6	3	4
G3N016	CRKL	Uncharacterized protein	33.386	11	2	2	#N/A	#N/A	#N/A
E1BCF2	CRLF3	Uncharacterized protein	49.406	15.7	6	11	8.9	5	14
O19094	CROT	Peroxisomal carnitine O-octanoyltransferase	70.263	47.9	23	51	15.8	11	32
C4T8B4	CRP	C-reactive protein	25.292	14.3	3	5	#N/A	#N/A	#N/A
E1BDQ1	CRYGN	Uncharacterized protein	21.371	26.9	4	5	7.7	2	2
O97764	CRYZ	Zeta-crystallin	35.382	55.8	15	46	29.7	9	50
Q59A28	CRYZL1	Quinone oxidoreductase-like protein	38.811	16.3	5	8	2.9	1	2
Q29RK1	CS	Citrate synthase, mitochondrial	51.772	35.2	15	52	27.5	13	61
F1MS94	CSDE1	Uncharacterized protein	85.803	22.9	18	32	10.2	9	14
F1MWN1	CSE1L	Exportin-2	110.4	42	41	110	14.3	16	47
Q0VBZ0	CSK	Tyrosine-protein kinase CSK	50.633	1.8	1	2	#N/A	#N/A	#N/A
P67827	CSNK1A1	Casein kinase I isoform alpha	37.567	#N/A	#N/A	#N/A	7.4	3	4
E1BGK5	CSNK1D	Casein kinase I isoform delta	21.271	6.4	1	3	#N/A	#N/A	#N/A
E1BNW0	CSNK1G1	Casein kinase I isoform gamma-1	50.385	4.1	2	2	#N/A	#N/A	#N/A
P68399	CSNK2A1	Casein kinase II subunit alpha	45.143	47.8	14	30	17.6	5	9
P20427	CSNK2A2	Casein kinase II subunit alpha	41.27	56	16	28	22.6	7	13
P67868	CSNK2B	Casein kinase II subunit beta	24.942	33.5	8	28	19.5	4	12
Q32LE9	CSRP2	Cysteine and glycine-rich protein 2	20.954	5.2	1	1	#N/A	#N/A	#N/A
E1BE91	CSRP2BP	Uncharacterized protein	82.434	#N/A	#N/A	#N/A	1.1	1	3

				Biological Replicate 1			Biological Replicate 2		
Accession number	Gene symbol	Discription	MW(kDa)	Sequence coverage(%)	Unique peptides	MS/MS count	Sequence coverage(%)	Unique peptides	MS/MS count
P25417	CSTB	Cystatin-B	11.139	50	4	16	37.8	4	10
F1MDR5	CTAGE5	Uncharacterized protein	92.323	1.5	1	1	2.8	2	3
F1MYP4	CTBP1	Uncharacterized protein	45.303	#N/A	#N/A	#N/A	1.9	1	1
Q58DW2	CTH	Cystathionine gamma-lyase	44.405	49.6	14	37	10.6	4	16
Q3MHM6	CTNNA1	Catenin alpha-1	100.13	26.4	22	29	17.7	14	23
G3N018	CTNNA3	Uncharacterized protein	99.932	#N/A	#N/A	#N/A	2.2	1	1
Q0VCX4	CTNNB1	Catenin beta-1	85.51	29.7	15	42	17.9	13	30
E1BQ11	CTNND1	Uncharacterized protein	106.86	24.6	21	37	11.5	12	17
F1MM16	CTNND2	Uncharacterized protein (Fragment)	122.98	0.9	1	2	#N/A	#N/A	#N/A
A0JNE9	CTPS	CTP synthase	66.753	33.8	14	34	13.4	7	19
Q1RMS2	CTPS2	CTP synthase 2	65.48	7.3	1	1	3.6	1	1
P07688	CTSB	Cathepsin B	36.661	20.6	6	15	14	5	12
Q3ZCJ8	CTSC	Dipeptidyl peptidase 1	51.948	2.4	1	3	#N/A	#N/A	#N/A
F1MMR6	CTSD	Cathepsin D	44.704	29.4	12	44	18.7	9	36
Q0VCU3	CTSF	Cathepsin F	50.893	4.6	2	2	2.2	1	1
Q3T0I2	CTSH	Pro-cathepsin H	37.351	4.5	2	2	#N/A	#N/A	#N/A
P05689	CTSZ	Cathepsin Z	33.886	14.1	4	9	9.5	3	10
Q1RMR3	CTTN	Cortactin	60.265	18.4	11	23	22.9	13	21
A7MB95	CUGBP1	CUGBP1 protein	51.949	10.1	5	13	5.3	3	9
F1MYD0	CUL1	Uncharacterized protein	89.66	34	23	36	15.1	10	20
Q08DE9	CUL2	CUL2 protein	86.955	23.9	15	31	5.6	5	8
E1BIN5	CUL3	Uncharacterized protein	88.958	55.1	40	94	21.7	18	44
F1N3S4	CUL4A	Uncharacterized protein (Fragment)	83.885	17.9	4	8	6.7	2	3
E1BFD5	CUL4B	Uncharacterized protein (Fragment)	109.85	25.2	15	33	5.4	3	14
F1N7B5	CUL5	Uncharacterized protein	88.5	17.5	13	22	3.6	3	4
F1MTI7	CUTA	Protein CutA	16.446	9.1	1	1	#N/A	#N/A	#N/A
Q8WMV3	CXADR	Coxsackievirus and adenovirus receptor homolog	40.153	#N/A	#N/A	#N/A	4.9	2	2
P25930	CXCR4	C-X-C chemokine receptor type 4	39.938	3.4	1	2	#N/A	#N/A	#N/A
G3X6Y2	CXorf38	Uncharacterized protein	36.471	13.8	5	6	6.3	2	3
A5D9A7	CYB561A3	Cytochrome b ascorbate-dependent	29.803	3.4	1	1	#N/A	#N/A	#N/A
P00171	CYB5A	Cytochrome b5	15.329	47.8	5	16	25.4	3	10

				Biological Replicate 1			Biological Replicate 2		
**Accession number**	**Gene symbol**	**Discription**	**MW(kDa)**	**Sequence coverage(%)**	**Unique peptides**	**MS/MS count**	**Sequence coverage(%)**	**Unique peptides**	**MS/MS count**
Q0P5F6	CYB5B	CYB5B protein	16.278	10.3	2	4	#N/A	#N/A	#N/A
G5E5N5	CYB5R1	NADH-cytochrome b5 reductase 1	33.678	48.8	12	29	13.2	5	16
P07514	CYB5R3	NADH-cytochrome b5 reductase 3	34.121	62.1	20	79	22.6	8	39
P00125	CYC1	Cytochrome c1, heme protein,	35.296	34.5	10	23	24.9	7	24
E1BN47	CYFIP1	Uncharacterized protein	148.79	17.7	11	30	4.6	6	10
F1MX60	CYFIP2	Uncharacterized protein	148.47	9.9	1	2	#N/A	#N/A	#N/A
Q1RMU2	CYLD	Ubiquitin carboxyl-terminal	106.8	2.1	2	2	#N/A	#N/A	#N/A
Q2KI41	CYTH2	Cytohesin-2	47.492	10.5	3	7	#N/A	#N/A	#N/A
Q1JPD3	D2HGDH	D-2-hydroxyglutarate dehydrogenase, mitochondrial	59.056	24.6	12	35	17.6	9	30
A2VDK3	DAAM1	Dishevelled associated activator of morphogenesis 1	122.29	0.8	1	1	1.6	2	2
Q5E9C2	DAD1	Dolichyl-diphosphooligosaccharide--protein glycosyltransferase subunit	12.495	28.3	3	4	8.8	1	2
P82922	DAP3	28S ribosomal protein S29,	45.565	3.5	1	1	#N/A	#N/A	#N/A
F1MTX7	DARS	Aspartate--tRNA ligase, cytoplasmic	57.051	33.1	1	3	30.1	1	38
F1MEQ5	DARS2	Uncharacterized protein	73.747	4.3	2	3	#N/A	#N/A	#N/A
E1BAK6	DAZAP1	Uncharacterized protein (Fragment)	42.367	14.4	4	4	7.8	2	2
P07107	DBI	Acyl-CoA-binding protein	10.044	20.7	1	2	11.5	2	2
Q1RMT6	DBN1	Drebrin 1	72.16	3.5	2	2	#N/A	#N/A	#N/A
A6H7G2	DBNL	Drebrin-like protein	47.722	16.1	7	12	7.1	3	5
F1N3H0	DCAF8	Uncharacterized protein	66.07	10.8	6	8	4.1	3	6
Q3ZBS0	DCAKD	Dephospho-CoA kinase domain-containing protein	26.384	24.7	6	10	#N/A	#N/A	#N/A
E1BLC5	DCLK2	Uncharacterized protein	77.465	#N/A	#N/A	#N/A	4.1	2	2
F1N4I7	DCP1A	Uncharacterized protein	62.782	4	2	4	#N/A	#N/A	#N/A
Q8MJJ7	DCPS	m7GpppX diphosphatase	38.402	19.6	5	5	#N/A	#N/A	#N/A
F1MIC9	DCTN1	Uncharacterized protein	136.89	34.9	33	58	18.2	21	43
Q3ZCF0	DCTN2	Dynactin subunit 2	44.294	26.8	10	19	19.6	8	32
Q0P5A1	DCTN3	Dynactin subunit 3	21.191	33.3	7	12	19.4	4	8
E1BBI8	DCTN4	Uncharacterized protein	53.223	22.1	8	13	7.1	3	6
Q148G7	DCTN6	Dynactin subunit 6	20.675	#N/A	#N/A	#N/A	5.3	1	3

				Biological Replicate 1			Biological Replicate 2		
Accession number	Gene symbol	Discription	MW(kDa)	Sequence coverage(%)	Unique peptides	MS/MS count	Sequence coverage(%)	Unique peptides	MS/MS count
F1MDM6	DCUN1D1	DCN1-like protein	28.278	16.8	4	7	11.9	3	3
Q1RMX9	DCUN1D5	DCN1-like protein 5	27.362	4.2	1	1	#N/A	#N/A	#N/A
A1A4K3	DDB1	DNA damage-binding protein 1	126.93	38.7	40	72	15.1	18	36
F1MSS2	DDHD2	Uncharacterized protein	80.95	23.3	14	29	6.2	5	12
P31228	DDO	D-aspartate oxidase	37.659	38.7	11	36	21.1	6	11
F1N632	DDOST	Uncharacterized protein	47.303	45.3	17	43	18.9	9	14
Q1LZB0	DDRGK1	DDRGK domain-containing protein 1	35.667	19.8	4	6	6.4	2	2
A5PK65	DDT	D-dopachrome decarboxylase	12.877	79.7	8	21	28.8	3	7
Q0IIK5	DDX1	ATP-dependent RNA helicase DDX1	82.413	30.1	20	33	18.5	15	34
Q3ZBV2	DDX19A	ATP-dependent RNA helicase DDX19A	53.985	20.9	9	14	10.5	5	11
F1MRW7	DDX20	Uncharacterized protein	92.665	1.8	1	2	1.2	1	2
Q2TBP1	DDX25	ATP-dependent RNA helicase DDX25	54.618	2.1	1	1	#N/A	#N/A	#N/A
Q3T147	DDX39B	Spliceosome RNA helicase DDX39B	48.964	21.3	9	15	4.7	2	4
G5E631	DDX3X	Uncharacterized protein	73.127	47.5	26	77	28.7	18	50
Q5W5U4	DDX4	Probable ATP-dependent RNA helicase	79.443	22.8	11	17	6.2	3	3
Q29S22	DDX47	Probable ATP-dependent RNA helicase	50.912	2.2	1	1	#N/A	#N/A	#N/A
F1MBQ8	DDX5	Uncharacterized protein	69.147	13.5	7	8	7.8	5	6
G3MYF0	DDX53	Uncharacterized protein (Fragment)	68.457	7.4	5	6	4.3	3	6
E1BDM8	DDX6	Uncharacterized protein	54.376	61.7	27	98	36.9	15	58
F1N5J8	DECR1	Uncharacterized protein	35.352	39.5	9	22	28	8	20
Q3ZBW6	DECR2	2,4-dienoyl CoA reductase 2,	24.027	12.4	2	3	7.5	2	2
A5PK34	DEF6	DEF6 protein	73.73	1.6	1	1	#N/A	#N/A	#N/A
Q3ZBY7	DEGS1	Sphingolipid delta(4)-desaturase	38.07	14.9	3	4	4.6	1	1
F1N032	DENND4C	Uncharacterized protein	216.28	0.3	1	1	#N/A	#N/A	#N/A
E1BIJ1	DEPDC7	Uncharacterized protein	58.804	35.8	18	29	12.1	7	15
Q71SS4	DERL1	Derlin-1	28.864	19.1	5	7	11.6	3	7
F6PVF9	DERL2	Uncharacterized protein	27.567	17.2	2	4	#N/A	#N/A	#N/A
Q0P5E4	DERL3	Derlin-3	26.248	#N/A	#N/A	#N/A	3.5	1	1
Q0VC37	DFFA	DNAation factor, 45kDa, alpha	36.432	#N/A	#N/A	#N/A	5.2	2	6
Q58CZ0	DFFB	DNA fragmentation factor subunit	38.967	1.8	1	1	1.8	1	2
E1BFC3	DFNA5	Uncharacterized protein	54.888	2.6	1	1	#N/A	#N/A	#N/A
F1MIW3	DGAT1	O-acyltransferase	54.896	3.9	2	3	#N/A	#N/A	#N/A

				Biological Replicate 1			Biological Replicate 2		
Accession number	Gene symbol	Discription	MW(kDa)	Sequence coverage(%)	Unique peptides	MS/MS count	Sequence coverage(%)	Unique peptides	MS/MS count
A0JN54	DGKA	Diacylglycerol kinase alpha	82.671	3	1	1	#N/A	#N/A	#N/A
Q2KIN2	DGUOK	Deoxyguanosine kinase	31.984	34.7	7	20	5.8	2	7
A6QR14	DHCR24	DHCR24 protein	60.173	1.7	1	2	6	3	4
Q5E9J5	DHCR7	7-dehydrocholesterol reductase	54.271	11.8	5	13	4.8	3	8
Q148L6	DHDH	Trans-1,2-dihydrobenzene-1,2-diol dehydrogenase	36.583	6.3	2	3	#N/A	#N/A	#N/A
P00376	DHFR	Dihydrofolate reductase	21.604	62.6	12	22	34.8	7	11
Q3ZBV9	DHRS11	Dehydrogenase/reductase SDR family member 11	27.81	3.1	1	1	#N/A	#N/A	#N/A
Q17QU7	DHRS13	Dehydrogenase/reductase SDR family member 13	40.86	7.7	3	3	6.6	2	3
Q3T0R4	DHRS7B	Dehydrogenase/reductase SDR family member 7B	35.031	32.3	9	17	16.9	5	7
E1BFI2	DHTKD1	Uncharacterized protein	103.11	2	2	4	2	2	4
A5D7D9	DHX15	DHX15 protein	90.932	8.7	6	7	#N/A	#N/A	#N/A
Q2NKY8	DHX30	Putative ATP-dependent RNA helicase	135.94	3	4	5	#N/A	#N/A	#N/A
Q05B79	DHX36	DEAH (Asp-Glu-Ala-His) box	114.82	21.4	19	38	12	11	26
F1N1A2	DHX57	Uncharacterized protein	155.73	1.5	1	1	#N/A	#N/A	#N/A
Q28141	DHX9	ATP-dependent RNA helicase A	141.94	10.1	11	18	2.1	3	6
Q2NL36	DIABLO	Diablo homolog (Drosophila)	26.971	27.3	6	10	10.1	3	11
F1N0W1	DIAPH1	Uncharacterized protein	143.03	16.6	18	25	2.8	4	8
E1BMN2	DIAPH3	Uncharacterized protein	135.29	1.3	1	1	#N/A	#N/A	#N/A
F1MLE1	DIP2B	Uncharacterized protein	171.48	0.8	1	1	#N/A	#N/A	#N/A
F1N2Q9	DIRAS2	Uncharacterized protein	22.53	6	1	1	#N/A	#N/A	#N/A
A6QL81	DKK3	DKK3 protein	38.391	7.4	2	2	#N/A	#N/A	#N/A
F1N690	DLAT	Dihydrolipoyllysine-residue acetyltransferase component of pyruvate dehydrogenase complex	69.066	39.4	22	143	23	13	76
F1N206	DLD	Dihydrolipoyl dehydrogenase	54.187	55.6	23	91	25.3	13	78
Q08DQ9	DLGAP5	Discs, large (Drosophila) homolog-associated protein 5	95.428	20.7	18	30	9.6	8	17

				Biological Replicate 1			Biological Replicate 2		
Accession number	Gene symbol	Discription	MW(kDa)	Sequence coverage(%)	Unique peptides	MS/MS count	Sequence coverage(%)	Unique peptides	MS/MS count
P11179	DLST	Dihydrolipoyllysine-residue succinyltransferase component of 2-oxoglutarate dehydrogenase complex, mitochondrial	48.972	42	18	90	31	15	86
Q1LZ99	DMAP1	DNA methyltransferase 1 associated protein 1	53.079	3.4	1	1	#N/A	#N/A	#N/A
F1MDJ6	DMGDH	Uncharacterized protein	97.269	2.1	2	2	#N/A	#N/A	#N/A
E1BMP7	DNA2	DNA replication ATP-dependent helicase/nuclease DNA2	120.48	1.1	1	2	#N/A	#N/A	#N/A
Q5E954	DNAJA1	DnaJ homolog subfamily A member 1	44.882	37.3	11	23	20.7	9	21
Q2HJ94	DNAJA2	DnaJ homolog subfamily A member 2	45.761	25.2	9	16	13.6	6	22
A1A4J9	DNAJA3	DnaJ (Hsp40) homolog, subfamily A, member 3	49.253	22.1	12	22	14.8	7	20
F1N6Q0	DNAJA4	Uncharacterized protein	44.753	11.8	5	9	6.3	3	6
Q3MI00	DNAJB1	DnaJ homolog subfamily B member 1	38.217	47.4	18	35	23.5	8	22
Q3ZBA6	DNAJB11	DnaJ homolog subfamily B member 11	40.503	30.2	11	18	16.8	7	22
F1MII1	DNAJB12	DnaJ homolog subfamily B member 12	41.275	3	1	1	8.7	3	4
Q0III6	DNAJB6	DnaJ homolog subfamily B member 6	26.943	12.8	3	3	16.1	4	5
G3MZ88	DNAJB9	Uncharacterized protein	25.715	6.3	1	1	#N/A	#N/A	#N/A
F1N151	DNAJC10	Uncharacterized protein	91.221	13.1	10	17	2	2	3
Q2NL21	DNAJC11	DnaJ homolog subfamily C member 11	63.236	31.5	15	36	11.6	7	8
F1MDR7	DNAJC13	Uncharacterized protein	254.44	2.5	5	8	0.4	1	1
A1A4R3	DNAJC15	DnaJ (Hsp40) homolog, subfamily C, member 15	15.85	6	1	1	#N/A	#N/A	#N/A
Q3ZBN8	DNAJC19	Mitochondrial import inner membrane translocase subunit TIM14	12.498	7.8	1	1	#N/A	#N/A	#N/A
Q1RMH9	DNAJC2	DnaJ homolog subfamily C member 2	71.785	1.9	1	1	#N/A	#N/A	#N/A
Q27968	DNAJC3	DnaJ homolog subfamily C member 3	57.704	39.1	19	37	20.6	12	38
Q29455	DNAJC5	DnaJ homolog subfamily C member 5	22.133	37.4	4	9	#N/A	#N/A	#N/A
F1N7H0	DNAJC7	Uncharacterized protein	57.068	23.4	12	18	8.4	5	7
E1BCE0	DNAJC8	Uncharacterized protein (Fragment)	30.722	6.5	2	2	3.4	1	2
F1MTK0	DNAJC9	Uncharacterized protein (Fragment)	30.306	41.4	10	23	19.5	6	11

				Biological Replicate 1			Biological Replicate 2		
Accession number	Gene symbol	Discription	MW(kDa)	Sequence coverage(%)	Unique peptides	MS/MS count	Sequence coverage(%)	Unique peptides	MS/MS count
P56541	DNASE2	Deoxyribonuclease-2-alpha	40.282	18.6	7	14	6.8	4	9
E1BGR3	DNLZ	Uncharacterized protein	18.485	5.3	1	1	5.3	1	3
Q2KIA5	DNM1L	Dynamin-1-like protein	83.351	53	35	95	24.2	20	54
E1BA62	DNM3	Uncharacterized protein	58.805	12.3	1	1	#N/A	#N/A	#N/A
E1BIL8	DNMBP	Uncharacterized protein	178.08	0.9	1	2	#N/A	#N/A	#N/A
Q24K09	DNMT1	DNA (cytosine-5)-methyltransferase	182.84	8.2	12	13	1.7	3	3
Q2HJH1	DNPEP	Aspartyl aminopeptidase	51.828	51	16	49	18.3	8	15
E1BM28	DNPH1	2-deoxynucleoside 5-phosphate N-hydrolase 1	18.373	20.6	2	3	#N/A	#N/A	#N/A
F1MJ73	DOCK5	Uncharacterized protein (Fragment)	210.71	8.5	14	19	3.6	7	8
Q0VC53	DOHH	Deoxyhypusine hydroxylase	33.26	6.3	2	2	#N/A	#N/A	#N/A
Q58CR4	DOLK	Dolichol kinase	59.189	4.1	2	4	#N/A	#N/A	#N/A
F1MW49	DOLPP1	Uncharacterized protein	27.039	10.1	2	2	#N/A	#N/A	#N/A
Q59A29	donson	DONSON protein	62.237	4.1	1	1	#N/A	#N/A	#N/A
F1MPE2	DOPEY1	Uncharacterized protein	276.41	#N/A	#N/A	#N/A	0.7	2	2
E1BAK4	DOPEY2	Uncharacterized protein	255.55	#N/A	#N/A	#N/A	0.3	1	2
Q5EA65	DPAGT1	UDP-N-acetylglucosamine--dolichyl-phosphate N-acetylglucosaminephosphotransferase	46.044	4.7	2	4	#N/A	#N/A	#N/A
Q5E982	DPH5	Diphthine synthase	31.662	3.2	1	1	#N/A	#N/A	#N/A
F2Z4F5	DPP3	Uncharacterized protein	82.031	18.6	8	9	3.4	3	3
P81425	DPP4	Dipeptidyl peptidase 4	88.368	8.1	6	9	8.4	7	12
H9GW42	DPP7	Uncharacterized protein (Fragment)	35.813	3	1	1	#N/A	#N/A	#N/A
F1N1L4	DPP8	Uncharacterized protein	103.35	23.1	16	27	5.5	5	7
E1BI31	DPP9	Uncharacterized protein (Fragment)	98.93	10	8	9	6.7	6	8
F1N3H6	DPY19L1	Uncharacterized protein	84.603	5.1	3	3	#N/A	#N/A	#N/A
Q3MHP5	DRG1	Developmentally-regulated GTP-binding protein 1	40.542	27.8	8	16	13.6	5	8
Q58D56	DRG2	Developmentally-regulated GTP-binding protein 2	40.749	9.1	2	3	#N/A	#N/A	#N/A
F1MTU0	DSCC1	Uncharacterized protein	44.991	5.6	2	2	#N/A	#N/A	#N/A
E1BKT9	DSP	Uncharacterized protein	332.38	2.1	6	7	1	3	4

				Biological Replicate 1			Biological Replicate 2		
Accession number	Gene symbol	Discription	MW(kDa)	Sequence coverage(%)	Unique peptides	MS/MS count	Sequence coverage(%)	Unique peptides	MS/MS count
F1MPT5	DST	Uncharacterized protein (Fragment)	844.19	#N/A	#N/A	#N/A	0.1	1	4
Q5E9D5	DSTN	Destrin	18.506	83.6	19	113	59.4	13	59
Q2T9V8	DTD1	D-tyrosyl-tRNA(Tyr) deacylase 1	23.232	44.5	9	23	18.7	5	13
E1BMA8	DTNA	Dystrobrevin	83.773	1.7	1	4	#N/A	#N/A	#N/A
Q5BIS8	DTX2	Deltex 2	61.918	10.5	6	12	5.8	4	6
F1MJB1	DTYMK	Uncharacterized protein	23.847	42.9	10	21	37.3	9	25
F1MW70	DUSP12	Uncharacterized protein	38.265	3.5	1	1	#N/A	#N/A	#N/A
F1ME76	DUSP13	Uncharacterized protein	22.341	19.7	3	4	8.6	2	3
Q2NKU1	DUT	Deoxyuridine triphosphatase	20.017	41.1	6	10	11.9	2	5
Q5E9Y5	DXO	Decapping and exoribonuclease	45.168	10.6	3	3	#N/A	#N/A	#N/A
E1BE17	DYM	Uncharacterized protein	75.943	10.2	8	11	9.4	7	10
E1BDX8	DYNC1H1	Uncharacterized protein	532.05	0.4	2	2	2.6	12	15
Q0III3	DYNC1I2	Cytoplasmic dynein 1 intermediate	68.376	21.7	9	16	14.2	7	12
Q2KII9	DYNC1LI1	Dynein, cytoplasmic 1, light intermediate chain 1	56.492	45	17	40	33.4	15	35
F1MBQ0	DYNC1LI2	Uncharacterized protein (Fragment)	54.665	3.4	1	2	#N/A	#N/A	#N/A
P61285	DYNLL1	Dynein light chain 1, cytoplasmic	10.366	65.2	4	101	64	4	52
Q3MHR3	DYNLL2	Dynein light chain 2, cytoplasmic	10.35	65.2	4	13	33.7	2	11
Q3T140	DYNLRB1	Dynein light chain roadblock-type 1	10.99	12.5	1	3	#N/A	#N/A	#N/A
P63171	DYNLT1	Dynein light chain Tctex-type 1	12.452	14.2	1	1	14.2	1	1
A6QLR8	DYNLT3	DYNLT3 protein	13.037	8.6	1	2	8.6	1	1
G3N260	EARS2	Uncharacterized protein	58.426	4.4	2	5	4.2	2	3
F6PRB5	ECH1	Uncharacterized protein	36.041	27.7	9	15	18	6	14
Q58DM8	ECHS1	Enoyl-CoA hydratase, mitochondrial	31.243	36.9	10	58	25.2	7	32
F1MTT7	ECI1	3,2-trans-enoyl-CoA isomerase, mitochondrial	33.216	9.2	3	5	12.2	4	9
F1MWY9	ECI2	Uncharacterized protein (Fragment)	40.736	37.2	13	49	23.8	8	36
F1MJ58	ECT2	Uncharacterized protein	103.59	7.2	5	6	#N/A	#N/A	#N/A
E1BEA2	EDC3	Uncharacterized protein	55.89	9	4	6	6.7	3	5
E1BJH1	EDC4	Uncharacterized protein	152.02	9.5	10	16	7.8	11	19
E1B789	EDEM3	Uncharacterized protein	104.34	1.3	1	1	#N/A	#N/A	#N/A

				Biological Replicate 1			Biological Replicate 2		
Accession number	Gene symbol	Discription	MW(kDa)	Sequence coverage(%)	Unique peptides	MS/MS count	Sequence coverage(%)	Unique peptides	MS/MS count
Q3T0V7	EDF1	Endothelial differentiation-related factor 1	16.368	#N/A	#N/A	#N/A	16.9	3	3
E1BPX2	EDIL3	Uncharacterized protein	53.719	11.9	6	8	7.7	4	11
F1MN61	EEA1	Uncharacterized protein (Fragment)	137.18	13.1	14	17	14.5	19	34
P68103	EEF1A1	Elongation factor 1-alpha 1	50.14	53.2	11	144	21.9	13	125
Q5E983	EEF1B	Elongation factor 1-beta	24.804	56.4	10	39	40.9	9	31
A5D989	EEF1D	Elongation factor 1-delta	31.141	66.8	14	46	42.1	12	58
Q3SZ15	EEF1E1	Eukaryotic translation elongation factor 1 epsilon 1	19.756	17.2	3	8	#N/A	#N/A	#N/A
Q3SZV3	EEF1G	Elongation factor 1-gamma	50.377	69.8	30	131	36.1	21	71
Q3SYU2	EEF2	Elongation factor 2	95.367	66.2	50	213	25.9	22	67
A3KN48	EEFSEC	EEFSEC protein	63.632	19	10	15	5.3	3	4
Q3MHJ7	EEPD1	Endonuclease/exonuclease/phosphatase family domain-containing protein	62.786	2.3	1	6	#N/A	#N/A	#N/A
E1BCB9	EFCAB12	Uncharacterized protein	66.697	1.4	1	1	#N/A	#N/A	#N/A
G3X7C2	EFCAB4A	Uncharacterized protein (Fragment)	45.046	#N/A	#N/A	#N/A	3	1	4
A5D7A0	EFHD2	EF-hand domain-containing protein	26.918	43.8	9	22	24.4	5	11
A6H7H9	EFS	EFS protein	58.483	2	1	1	#N/A	#N/A	#N/A
E1BH79	EFTUD1	Uncharacterized protein	126.15	8.6	7	9	2.1	3	3
F1N6D5	EFTUD2	116 kDa U5 small nuclear ribonucleoprotein component	109.44	12.7	9	12	2.7	2	2
Q5E9R3	EHD1	EH domain-containing protein 1	60.681	41.8	20	45	14.6	8	20
E1BJV0	EHD4	Uncharacterized protein	61.024	8.7	2	3	#N/A	#N/A	#N/A
Q08DE5	EI24	Etoposide-induced protein 2.4	38.921	7.9	3	3	2.4	1	2
Q32LC3	EIF1AX	Eukaryotic translation initiation factor 1A, X-linked	16.46	50.7	7	28	35.4	5	16
Q32LJ9	EIF1B	Eukaryotic translation initiation factor 1B	12.823	25.7	2	2	#N/A	#N/A	#N/A
F1MBF0	EIF2A	Eukaryotic translation initiation factor 2 subunit 1	65.25	8.9	6	7	2.6	2	3
Q0IIF2	EIF2B1	Translation initiation factor eIF-2B subunit alpha	33.87	#N/A	#N/A	#N/A	6.2	2	2

				Biological Replicate 1			Biological Replicate 2		
**Accession number**	**Gene symbol**	**Discription**	**MW(kDa)**	**Sequence coverage(%)**	**Unique peptides**	**MS/MS count**	**Sequence coverage(%)**	**Unique peptides**	**MS/MS count**
Q5E9B4	EIF2B2	Translation initiation factor eIF-2B subunit beta	38.994	6.8	3	3	5.7	2	2
A5PJI7	EIF2B3	Translation initiation factor eIF-2B subunit gamma	50.218	4	2	2	5.8	3	4
Q3T058	EIF2B4	Translation initiation factor eIF-2B subunit delta	57.468	2.3	1	1	5.5	2	2
E1BPB3	EIF2B5	Uncharacterized protein	80.913	4.9	4	6	#N/A	#N/A	#N/A
P68102	EIF2S1	Eukaryotic translation initiation factor 2 subunit 1	36.108	32.4	10	18	19.4	7	9
Q5E9D0	EIF2S2	Eukaryotic translation initiation factor 2 subunit 2	38.286	24.3	8	14	13.5	5	8
Q2KHU8	EIF2S3	Eukaryotic translation initiation factor 2 subunit 3	51.065	31.6	13	21	16.7	9	20
E1B7R4	EIF3A	Eukaryotic translation initiation factor 3 subunit A	166.27	16.5	23	38	10.4	17	29
A7MB16	EIF3B	Eukaryotic translation initiation factor 3 subunit B	88.918	31.3	19	38	13.6	10	26
Q3SYW6	EIF3C	Eukaryotic translation initiation factor 3 subunit C	105.36	12.7	14	19	11.7	12	22
Q3T122	EIF3D	Eukaryotic translation initiation factor 3 subunit D	63.93	27.4	9	12	#N/A	#N/A	#N/A
Q3T102	EIF3E	Eukaryotic translation initiation factor 3 subunit E	52.19	29.2	12	15	15.3	7	19
E1BLZ8	EIF3F	Uncharacterized protein (Fragment)	40.188	20.6	7	15	11.5	4	11
Q56JZ5	EIF3H	Eukaryotic translation initiation factor 3 subunit H	39.906	19.9	5	9	13.9	4	12
Q5E966	EIF3I	Eukaryotic translation initiation factor 3 subunit I	36.472	31.7	9	17	26.8	8	22
Q0VCU8	EIF3J	Eukaryotic translation initiation factor 3 subunit J	28.951	21.1	6	9	15.6	5	8
Q3T0V3	EIF3K	Eukaryotic translation initiation factor 3 subunit K	25.073	14.7	3	5	9.6	2	4

				Biological Replicate 1			Biological Replicate 2		
Accession number	Gene symbol	Discription	MW(kDa)	Sequence coverage(%)	Unique peptides	MS/MS count	Sequence coverage(%)	Unique peptides	MS/MS count
Q3ZCK1	EIF3L	Eukaryotic translation initiation factor 3 subunit L	66.726	23.2	14	20	24.1	13	21
Q3T148	EIF3M	Eukaryotic translation initiation factor 3 subunit M	42.415	23.9	8	13	8.8	4	7
Q3SZ54	EIF4A1	Eukaryotic initiation factor 4A-I	46.153	58.1	13	116	34	7	60
Q3SZ65	EIF4A2	Eukaryotic initiation factor 4A-II	46.402	50.1	10	20	26.3	3	11
Q2NL22	EIF4A3	Eukaryotic initiation factor 4A-III	46.84	20.2	6	6	12.9	3	3
F1MYS7	EIF4B	Uncharacterized protein	68.817	5.9	4	4	9	5	10
Q9N0T5	EIF4E	Eukaryotic translation initiation factor 4E	25.079	26.7	6	12	18.4	4	9
F1N030	EIF4E1B	Uncharacterized protein (Fragment)	26.302	36.1	7	17	14.3	3	12
Q0II31	EIF4E2	Eukaryotic translation initiation factor 4E family member 2	27.192	5.9	1	1	#N/A	#N/A	#N/A
E1BG99	EIF4ENIF1	Uncharacterized protein	108.43	28.2	22	53	17.8	15	33
F1MX04	EIF4G1	Uncharacterized protein (Fragment)	178.59	2.2	3	3	5	7	7
Q95L46	EIF4G2	Eukaryotic translation initiation factor 4 gamma 2	102.36	27.1	23	34	8.9	8	16
F1N0F7	EIF5	Uncharacterized protein	48.971	17.5	7	8	4.9	2	2
Q6EWQ7	EIF5A	Eukaryotic translation initiation factor 5A-1	16.832	56.5	10	36	20.8	4	15
F1N6Y7	EIF5B	Uncharacterized protein (Fragment)	138.28	2	2	2	0.8	1	1
Q9TU47	EIF6	Eukaryotic translation initiation	26.513	38.4	7	12	4.1	1	3
Q3ZCE2	ELAVL1	ELAV (Embryonic lethal, abnormal vision, Drosophila)-like 1 (Hu	36.163	26.7	8	11	15.6	4	7
G3N063	ELAVL2	Uncharacterized protein	42.545	13.7	2	12	19.3	2	12
F1MUZ4	ELMO2	Engulfment and cell motility	82.545	14.7	7	14	4.3	4	7
Q0IIE6	ELMOD1	ELMO domain-containing protein 1	38.036	#N/A	#N/A	#N/A	1.8	1	1
Q08DZ3	ELMOD2	ELMO domain-containing protein 2	34.83	#N/A	#N/A	#N/A	9.9	3	3
Q2TBH6	ELP4	Elongator complex protein 4	46.429	2.8	1	1	#N/A	#N/A	#N/A
E1BBT8	EMC1	Uncharacterized protein	111.82	7.1	6	7	2.4	3	5
A1A4M2	EMC10	ER membrane protein complex subunit	27.425	#N/A	#N/A	#N/A	3.1	1	2
Q5E993	EMC2	ER membrane protein complex subunit	34.804	18.9	5	6	10.1	3	6

				Biological Replicate 1			Biological Replicate 2		
Accession number	Gene symbol	Discription	MW(kDa)	Sequence coverage(%)	Unique peptides	MS/MS count	Sequence coverage(%)	Unique peptides	MS/MS count
Q3ZCB8	EMC3	ER membrane protein complex subunit	29.92	6.5	1	1	5.4	1	2
Q3T0K8	EMC4	ER membrane protein complex subunit	20.086	7.1	1	1	13.7	2	4
Q32KL5	EMC8	ER membrane protein complex subunit	23.777	10.5	2	3	12.9	3	7
Q6XZP8	EMD	Emerin	29.389	4.6	1	1	#N/A	#N/A	#N/A
F1MNZ8	EMID1	Uncharacterized protein	47.51	#N/A	#N/A	#N/A	4.1	2	4
F1N6P6	EML1	Uncharacterized protein	89.997	1.3	1	1	#N/A	#N/A	#N/A
E1BB08	EML2	Uncharacterized protein	89.144	20.8	13	28	10	8	15
F1N1R8	EML4	Uncharacterized protein	110.36	15.6	14	19	10.5	9	15
F1N214	ENAH	Uncharacterized protein	86.231	10.7	8	11	10.2	8	16
A7YY77	ENDOD1	ENDOD1 protein	54.868	14.6	6	9	5.6	2	2
P38447	ENDOG	Endonuclease G, mitochondrial	32.261	5	1	1	#N/A	#N/A	#N/A
Q9XSJ4	ENO1	Alpha-enolase	47.326	81.6	25	114	44.7	19	86
Q3ZC09	ENO3	Beta-enolase	47.095	33.2	3	6	#N/A	#N/A	#N/A
Q0VD27	ENOPH1	Enolase-phosphatase E1	28.929	18.4	4	10	7.7	2	3
F1MNS5	ENPP1	Uncharacterized protein	104.39	18.3	14	24	4	4	14
F1N5W4	ENPP5	Uncharacterized protein (Fragment)	41.751	9.4	3	10	#N/A	#N/A	#N/A
E1BDB0	EPB41L2	Uncharacterized protein	111.84	37.7	33	67	25.8	18	59
Q58CU2	EPB41L5	Band 4.1-like protein 5	57.168	10.6	5	5	#N/A	#N/A	#N/A
Q3T0L5	EPCAM	Epithelial cell adhesion molecule	34.859	5.1	1	2	#N/A	#N/A	#N/A
A6QLI0	EPDR1	Mammalian ependymin-related protein	26.485	4.7	1	1	#N/A	#N/A	#N/A
F1N579	EPN1	Uncharacterized protein	60.095	14.1	4	12	3	2	2
F1N4F8	EPN3	Uncharacterized protein	64.97	4.8	1	1	#N/A	#N/A	#N/A
G3X6L9	EPRS	Uncharacterized protein	169.92	26.7	38	68	13.2	21	44
A5D7I1	EPS15	EPS15 protein	100.21	4.1	3	4	4.3	4	6
A7MB30	EPS15L1	EPS15L1 protein	88.003	11.7	8	8	11.7	8	14
G3X8H0	EPSTI1	Uncharacterized protein	27.951	11.6	2	3	#N/A	#N/A	#N/A
E1BPW2	ERC1	Uncharacterized protein	128.17	2.5	3	3	#N/A	#N/A	#N/A
E1BFL2	ERCC6	Uncharacterized protein	166.8	#N/A	#N/A	#N/A	0.7	1	1
F6QNX4	ERGIC1	Uncharacterized protein	32.585	19.3	4	5	6.2	2	6
Q0IIJ1	ERGIC2	ERGIC and golgi 2	42.613	18.6	6	8	10.6	4	6
Q5EAE0	ERGIC3	Endoplasmic reticulum-Golgi intermediate compartment protein 3	43.374	10.4	5	7	15.4	7	21

				Biological Replicate 1			Biological Replicate 2		
Accession number	Gene symbol	Discription	MW(kDa)	Sequence coverage(%)	Unique peptides	MS/MS count	Sequence coverage(%)	Unique peptides	MS/MS count
F1ML00	ERI3	ERI1 exoribonuclease 3	18.185	10	2	3	5.6	1	1
F1MGK3	ERLEC1	Uncharacterized protein	48.806	20.5	7	13	10.7	4	8
G3N0U8	ERLIN1	Uncharacterized protein	39.067	37.6	10	16	22.1	5	15
Q1RMU4	ERLIN2	Erlin-2	37.73	61.5	17	72	32.8	9	53
F1MI95	ERMP1	Uncharacterized protein (Fragment)	98.637	24.4	18	37	13.8	12	36
A5PJN2	ERO1L	ERO1-like protein alpha	54.296	21.6	9	14	13.5	6	14
F1MW08	ERO1LB	Uncharacterized protein	53.724	4	1	1	#N/A	#N/A	#N/A
P81623	ERP29	Endoplasmic reticulum resident	28.806	55.8	13	64	37.6	10	37
Q3T0L2	ERP44	Endoplasmic reticulum resident	46.836	25.1	10	25	14.5	7	19
Q3T0U3	ES1	Es1 protein	28.699	73.4	14	60	35	9	50
Q08E20	ESD	S-formylglutathione hydrolase	31.548	63.1	14	41	18.4	5	14
F1MW01	ESPL1	Uncharacterized protein	230.94	3.6	6	6	#N/A	#N/A	#N/A
F1MVN3	ESRP1	Uncharacterized protein	74.933	8.7	4	6	10.5	7	10
F1MN29	ESYT2	Uncharacterized protein (Fragment)	97.22	13.9	11	14	7.3	6	15
Q0VCX5	ETF1	Eukaryotic peptide chain release factor subunit 1	49.03	18.1	7	18	6.9	3	4
F1MWR3	ETFA	Electron transfer flavoprotein subunit alpha, mitochondrial	34.957	49.5	14	43	46.2	13	75
Q2TBV3	ETFB	Electron transfer flavoprotein subunit beta	27.699	59.2	17	54	58.4	14	60
Q2KIG0	ETFDH	Electron transfer flavoprotein-ubiquinone oxidoreductase,	68.611	35	19	34	24.3	14	47
Q3T094	ETHE1	Persulfide dioxygenase ETHE1, mitochondrial	27.9	26.8	5	6	13	3	5
F1MQT2	ETV6	Transcription factor ETV6	46.851	5.8	3	5	#N/A	#N/A	#N/A
A2VDX7	EXO5	Exonuclease V	41.511	2.7	1	3	#N/A	#N/A	#N/A
F1MC63	EXOC1	Uncharacterized protein	102.85	#N/A	#N/A	#N/A	3.2	3	4
E1BDW9	EXOC2	Uncharacterized protein	103.34	0.9	1	1	#N/A	#N/A	#N/A
Q0V8C2	EXOC3	Exocyst complex component 3	85.58	6.2	4	5	1.2	1	1
A6QLD1	EXOC4	EXOC4 protein	110.48	2.2	2	2	5.2	5	6
E1BAZ9	EXOG	Uncharacterized protein	41.076	55.2	14	42	31.8	9	20
P31976	EZR	Ezrin	68.759	70.9	39	250	48.9	27	181

				Biological Replicate 1			Biological Replicate 2		
Accession number	Gene symbol	Discription	MW(kDa)	Sequence coverage(%)	Unique peptides	MS/MS count	Sequence coverage(%)	Unique peptides	MS/MS count
P00743	F10	Coagulation factor X	54.51	#N/A	#N/A	#N/A	1.8	1	3
Q9XT56	F11R	Junctional adhesion molecule A	32.455	7.7	2	2	2.7	1	2
F1MBC5	F9	Coagulation factor IX	36.365	6.2	2	3	#N/A	#N/A	#N/A
P10790	FABP3	Fatty acid-binding protein, heart	14.779	61.7	10	45	46.6	8	30
P55052	FABP5	Fatty acid-binding protein,	15.074	74.8	13	54	54.8	8	25
Q645M6	FADD	Protein FADD	23.002	17.2	3	4	6.7	1	2
Q29RM7	FAF1	Fas (TNFRSF6) associated factor 1	73.868	#N/A	#N/A	#N/A	1.7	1	2
Q2HJD0	FAF2	FAS-associated factor 2	52.663	16.6	6	10	#N/A	#N/A	#N/A
Q2HJ98	FAHD1	Acylpyruvase FAHD1, mitochondrial	24.499	29.9	5	9	9.5	2	10
Q2KIB0	FAHD2	Fumarylacetoacetate hydrolase domain-containing protein 2	34.556	33.8	9	28	24.8	7	16
Q2KI00	FAM107B	Protein FAM107B	15.586	6.1	1	1	#N/A	#N/A	#N/A
Q2T9N1	FAM114A1	Protein FAM114A2	54.824	41.2	18	44	22.2	11	24
A6QNT4	FAM120B	Constitutive coactivator of peroxisome proliferator-activated	79.075	1.4	1	1	#N/A	#N/A	#N/A
F1N2F1	FAM129A	Uncharacterized protein	103.57	7.4	6	9	5.7	5	7
F1MD34	FAM129B	Uncharacterized protein (Fragment)	84.038	22.1	15	25	6.1	5	11
F1MUR7	FAM134A	Uncharacterized protein	57.647	4.6	1	1	1.7	1	2
E1BBW7	FAM134C	Uncharacterized protein	51.303	9.9	4	6	#N/A	#N/A	#N/A
Q2HJI3	FAM136A	Protein FAM136A	15.683	5.8	1	1	5.8	1	2
F1MLL5	FAM160A2	FTS and Hook-interacting protein (Fragment)	85.637	2.4	3	4	#N/A	#N/A	#N/A
F1N3S2	FAM162A	Protein FAM162A (Fragment)	16.582	25.5	5	14	7.6	1	3
E1BM49	FAM187B	Uncharacterized protein	41.94	#N/A	#N/A	#N/A	2.5	1	1
Q0IIH8	FAM188A	Protein FAM188A	49.678	16.2	6	9	4.9	2	2
F1MW97	FAM203A	Uncharacterized protein (Fragment)	43.575	2.5	1	1	#N/A	#N/A	#N/A
Q05B67	FAM210A	Protein FAM210A	31.025	10.3	2	2	13.9	4	5
Q3ZBK2	FAM213A	Redox-regulatory protein FAM213A	24.355	50.9	13	31	37.6	11	35
F1MTG3	FAM21A	Uncharacterized protein	145.5	9.4	9	16	4.4	5	10
A5PKI3	FAM3C	Protein FAM3C	24.796	11.9	3	4	18.9	5	10
I6L9J8	FAM45A	FAM45A protein	27.841	21.5	5	6	10.2	3	5
E1BAJ4	FAM47ESTBD1	Uncharacterized protein	36.556	17.4	6	10	19.5	6	14

				Biological Replicate 1			Biological Replicate 2		
Accession number	Gene symbol	Discription	MW(kDa)	Sequence coverage(%)	Unique peptides	MS/MS count	Sequence coverage(%)	Unique peptides	MS/MS count
Q2KJI3	FAM49B	Protein FAM49B	36.748	28.1	7	11	2.5	1	1
A0JN43	FAM62A	Family with sequence similarity 62 (C2 domain containing), member A	123.03	36	33	79	16.6	17	41
A2VE72	FAM8A1	Family with sequence similarity 8, member A1	44.283	6.2	2	2	3.3	1	2
F1N0S9	FAM98A	Uncharacterized protein	55.12	11.8	5	8	3.1	2	2
E1BJ38	FAM98C	Uncharacterized protein	38.275	3.7	1	3	#N/A	#N/A	#N/A
F1MFC6	FARP1	Uncharacterized protein	118.35	5.9	7	8	3.3	4	5
A7MBD4	FARSA	FARSA protein	57.494	26.6	11	21	10.4	6	15
A8E4P2	FARSB	FARSB protein	66.339	32.6	18	41	16.8	9	26
F1N647	FASN	Fatty acid synthase	274.26	0.6	1	1	0.9	3	4
F1N1T6	FASTKD2	Uncharacterized protein	78.216	4.9	3	7	3.6	3	7
F1MM59	FBL	Uncharacterized protein	33.962	5.6	2	2	#N/A	#N/A	#N/A
Q3SZB7	FBP1	Fructose-1,6-bisphosphatase 1	36.728	11.5	3	3	2.1	1	1
Q58DG6	FBXL20	F-box/LRR-repeat protein 20	48.423	#N/A	#N/A	#N/A	4.4	2	3
Q0VD31	FBXL4	F-box/LRR-repeat protein 4	70.294	1.1	1	2	#N/A	#N/A	#N/A
F1MSC9	FBXO10	Uncharacterized protein	104.22	2.4	2	2	3.2	3	4
E1BAY5	FBXO30	Uncharacterized protein	82.375	19.9	14	23	8.6	6	8
G5E639	FBXO45	Uncharacterized protein (Fragment)	30.673	8	2	2	7	2	3
Q3SX24	FBXO6	F-box only protein 6	30.767	13.6	4	5	7.2	2	3
Q2T9S7	FBXO7	F-box only protein 7	57.965	4.2	2	3	3.4	2	2
F1MLG0	FBXW11	Uncharacterized protein	61	55.7	31	71	33.6	9	54
F1MNN4	FBXW7	Uncharacterized protein	70.319	2.6	2	2	2.9	2	2
Q8WMY2	FDPS	Farnesyl pyrophosphate synthase	40.51	27.2	3	26	24.1	7	21
F1N3J0	FDX1	Adrenodoxin, mitochondrial	18.057	5.5	1	1	11	2	4
Q05B51	FDX1L	Adrenodoxin-like protein,	19.946	#N/A	#N/A	#N/A	5.9	1	4
G3N2Y7	FDXR	NADPH:adrenodoxin oxidoreductase, mitochondrial	55.035	31.3	0	1	22.3	0	20
P22600	FECH	Ferrochelatase, mitochondrial	46.934	36.8	12	22	17.3	7	11
Q58DH8	FEN1	Flap endonuclease 1	42.509	11.8	3	3	#N/A	#N/A	#N/A
E1BNE0	FER	Tyrosine-protein kinase	94.754	4.3	3	5	2.3	2	3
A7MB21	FERMT2	FERMT2 protein	77.873	3.1	2	2	#N/A	#N/A	#N/A

				Biological Replicate 1			Biological Replicate 2		
Accession number	Gene symbol	Discription	MW(kDa)	Sequence coverage(%)	Unique peptides	MS/MS count	Sequence coverage(%)	Unique peptides	MS/MS count
F1N1J5	FGFR1OP	FGFR1 oncogene partner (Fragment)	39.333	#N/A	#N/A	#N/A	3.5	1	3
Q148D3	FH	Fumarate hydratase	54.69	69	30	156	40.6	21	147
Q1KZG4	FHIT	Bis(5-adenosyl)-triphosphatase	16.951	73.2	11	38	28.9	5	16
G3MZ95	FHL1	Uncharacterized protein	31.91	15.4	4	4	#N/A	#N/A	#N/A
Q2YDE0	FIBP	Fibroblast growth factor (Acidic) intracellular binding protein	41.157	9.2	3	3	#N/A	#N/A	#N/A
E1BIR0	FICD	Uncharacterized protein	51.771	#N/A	#N/A	#N/A	1.7	1	3
E1BKM0	FIP1L1	Uncharacterized protein	67.471	#N/A	#N/A	#N/A	4.7	2	3
Q3T0I5	FIS1	Mitochondrial fission 1 protein	16.937	40.8	6	10	21.1	3	8
F1MNV8	FKBP15	Peptidyl-prolyl cis-trans isomerase	130.85	12.6	12	23	13	14	21
P18203	FKBP1A	Peptidyl-prolyl cis-trans isomerase	11.909	25	2	6	25	2	4
Q32PA9	FKBP2	Peptidyl-prolyl cis-trans isomerase	15.404	53.6	6	15	#N/A	#N/A	#N/A
P26884	FKBP3	Peptidyl-prolyl cis-trans isomerase	25.191	13.4	3	4	3.6	1	1
Q9TRY0	FKBP4	Peptidyl-prolyl cis-trans isomerase	51.529	57.3	26	83	29.8	15	49
G3MXV0	FKBP5	Uncharacterized protein	51.12	#N/A	#N/A	#N/A	2.2	1	1
F1N2P6	FKBP8	Uncharacterized protein	44.359	30.9	11	22	23.6	9	18
Q2KJC8	FKBP9	Peptidyl-prolyl cis-trans isomerase	63.534	1.7	1	1	3.1	2	2
F6R6Q1	FLAD1	Uncharacterized protein	54.272	56.1	22	36	11.6	6	13
Q3B7L5	FLCN	Folliculin	64.203	1.6	1	1	#N/A	#N/A	#N/A
F1N365	FLII	Uncharacterized protein (Fragment)	144.23	5.1	6	9	2.9	4	5
E1BKX7	FLNB	Uncharacterized protein	281.61	#N/A	#N/A	#N/A	2.8	6	11
E1BE25	FLNC	Uncharacterized protein	290.78	3.4	6	7	2.1	4	6
Q08DN8	FLOT1	Flotillin-1	47.353	26.7	9	16	10.8	5	8
G3X6T9	FLOT2	Flotillin-2 (Fragment)	42.327	31.4	10	12	21	8	15
E1B718	FMNL3	Uncharacterized protein	111.13	4.9	1	1	#N/A	#N/A	#N/A
F1MXQ7	FMR1	Uncharacterized protein	71.187	34.8	15	36	15	7	26
Q0P592	FN3KRP	Fructosamine 3 kinase related	34.414	21	7	13	13.3	4	7
F1MSM9	FNBP1L	Uncharacterized protein (Fragment)	63.709	2.6	2	3	3.7	2	4
P29702	FNTA	Protein farnesyltransferase/geranylgeranyltransferase type-1 subunit alpha	43.845	15.5	5	5	#N/A	#N/A	#N/A
P49355	FNTB	Protein farnesyltransferase subunit	48.767	3.7	2	4	#N/A	#N/A	#N/A

				Biological Replicate 1			Biological Replicate 2		
Accession number	Gene symbol	Discription	MW(kDa)	Sequence coverage(%)	Unique peptides	MS/MS count	Sequence coverage(%)	Unique peptides	MS/MS count
F1MQ56	FOCAD	Uncharacterized protein	201.16	0.4	1	1	#N/A	#N/A	#N/A
Q1RMQ1	FRG1	FSHD region gene 1	29.157	8.9	2	2	#N/A	#N/A	#N/A
Q95117	FRZB	Secreted frizzled-related protein 3	36.234	2.5	1	2	8.3	3	7
Q3MHK9	FSCN1	Fascin	54.785	3.7	2	2	#N/A	#N/A	#N/A
Q05B84	FSD1	Fibronectin type III and SPRY domain-containing protein 1	55.719	27.6	13	24	11.5	6	9
F1MAU6	FSD1L	Uncharacterized protein	55.807	4.2	2	2	#N/A	#N/A	#N/A
O46415	FTL	Ferritin light chain	19.987	61.7	8	22	51.4	8	65
F1MX51	FUBP1	Uncharacterized protein (Fragment)	63.697	6	3	5	5.1	3	4
Q2KIM0	FUCA1	Tissue alpha-L-fucosidase	54.088	12.4	6	11	11.3	6	12
A6QP44	FUK	FUK protein	116.95	41.3	39	125	17.4	21	74
Q8MJN0	FUNDC2	FUN14 domain-containing protein 2	20.577	22.6	5	10	11.6	2	4
Q28009	FUS	RNA-binding protein FUS	52.31	2.7	1	2	#N/A	#N/A	#N/A
Q3ZBU5	FUSIP1	FUS interacting protein (Serine/arginine-rich) 1	22.121	6	1	1	#N/A	#N/A	#N/A
Q05B87	FXN	Frataxin, mitochondrial	23.566	#N/A	#N/A	#N/A	10.6	2	2
E1B9L5	FXR2	Uncharacterized protein (Fragment)	71.61	21.1	8	21	8.5	3	4
Q32LC7	G3BP	Ras GTPase-activating protein-binding protein 1	52.121	11.8	5	6	6.7	2	7
Q2KJ17	G3BP2	GTPase activating protein (SH3 domain) binding protein 2	50.681	12.7	5	10	9.4	3	5
F1MMK2	G6PD	Glucose-6-phosphate 1-dehydrogenase	59.336	72.4	32	125	34.4	18	55
Q9MYM4	GAA	Lysosomal alpha-glucosidase	104.76	27.3	21	45	8.6	7	16
P60519	GABARAPL2	Gamma-aminobutyric acid receptor-associated protein-like 2	13.667	40.2	6	9	23.1	4	7
F1MIB2	GAK	Uncharacterized protein	138.09	14.1	15	26	6.2	8	16
G1K1R6	GALK1	Galactokinase	42.242	53.1	20	50	21.9	7	32
F1MFK7	GALK2	Uncharacterized protein	49.191	14.3	7	11	5.6	3	3
F1MU84	GALNS	Uncharacterized protein (Fragment)	58.578	9.9	5	13	#N/A	#N/A	#N/A
G3X6R0	GALNT12	Uncharacterized protein (Fragment)	54.38	4.5	2	2	3.2	2	2
E1BB48	GALNT2	Uncharacterized protein	64.757	28	14	21	15.4	10	16
Q58CX1	GALT	Galactose-1-phosphate	41.287	16.7	4	5	#N/A	#N/A	#N/A

				Biological Replicate 1			Biological Replicate 2		
Accession number	Gene symbol	Discription	MW(kDa)	Sequence coverage(%)	Unique peptides	MS/MS count	Sequence coverage(%)	Unique peptides	MS/MS count
A6H7J3	GAN	GAN protein	67.602	24.5	13	18	5.4	3	5
F1N6Y1	GANAB	Uncharacterized protein	109.47	51.3	41	199	28.7	25	117
P10096	GAPDH	Glyceraldehyde-3-phosphate	35.868	80.2	24	446	57.4	18	325
Q2KJE5	GAPDHS	Glyceraldehyde-3-phosphate dehydrogenase, testis-specific	43.287	13.2	2	7	#N/A	#N/A	#N/A
A5D794	GAPVD1	GTPase-activating protein and VPS9 domain-containing protein 1	157.37	3.4	4	6	1	2	3
A5D7A2	GARS	GARS protein	83.117	41.3	25	60	17.5	13	34
Q59A32	GART	Trifunctional purine biosynthetic protein adenosine-3	107.91	34.6	31	63	15.7	14	31
F1N308	GATSL2	Uncharacterized protein (Fragment)	36.116	10.9	2	6	#N/A	#N/A	#N/A
Q0V8A3	GATSL3	GATS-like protein 3	36.041	16.4	5	12	12.8	3	6
Q2KHZ8	GBA	Glucosylceramidase	59.854	28.2	10	14	13.2	6	11
Q3SWX4	GBAS	Glioblastoma amplified sequence	33.419	35	15	46	24.5	12	40
F1MZP0	GBE1	Uncharacterized protein (Fragment)	75.793	20	13	16	2.6	2	3
F1N7I6	GCA	Uncharacterized protein	22.29	12.9	2	2	#N/A	#N/A	#N/A
Q0P5L8	GCAT	2-amino-3-ketobutyrate coenzyme A ligase, mitochondrial	45.165	43.9	15	91	16.5	8	31
E1BJZ4	GCC1	Uncharacterized protein	87.602	2.5	2	2	2.1	2	4
E1BB16	GCC2	Uncharacterized protein	195.46	#N/A	#N/A	#N/A	0.8	1	1
Q2KHZ9	GCDH	Glutaryl-CoA dehydrogenase,	48.471	21.9	7	14	3.7	2	2
Q32S38	GCLC	Glutamate-cysteine ligase catalytic subunit	69.022	49.6	26	89	26.3	14	42
Q2T9Y6	GCLM	Glutamate--cysteine ligase regulatory subunit	30.533	36.1	8	21	10.2	3	7
P20821	GCSH	Glycine cleavage system H protein, mitochondrial	18.791	17.9	2	4	#N/A	#N/A	#N/A
F1MLX9	GDA	Graves disease carrier protein	51.059	39.2	16	39	22	10	19
A6QQZ0	GDAP1	Ganglioside-induced differentiation-associated protein	41.201	10.1	4	7	10.6	5	6
Q2KIX2	GDAP2	Ganglioside-induced differentiation-associated protein	56.262	37.4	16	24	18.7	10	19

				Biological Replicate 1			Biological Replicate 2		
Accession number	Gene symbol	Discription	MW(kDa)	Sequence coverage(%)	Unique peptides	MS/MS count	Sequence coverage(%)	Unique peptides	MS/MS count
Q3T0T0	GDE1	Glycerophosphodiester	37.653	26.9	9	22	7.6	2	4
F1MKJ7	GDF3	Uncharacterized protein	40.662	19.5	7	33	4.9	2	7
Q9GK68	GDF9	Growth/differentiation factor 9	51.932	42.2	17	65	30.9	13	63
P21856	GDI1	Rab GDP dissociation inhibitor	50.565	69.4	19	64	29.5	7	24
P50397	GDI2	Rab GDP dissociation inhibitor beta	50.488	73.9	21	112	34.4	9	41
F1N2J0	GDPD1	Uncharacterized protein	33.537	5.5	1	1	5.2	2	2
Q5E9T1	GDPGP1	GDP-D-glucose phosphorylase 1	42.332	14	3	3	#N/A	#N/A	#N/A
F1MAW0	GEMIN4	Uncharacterized protein (Fragment)	120.41	1.7	2	2	2.7	3	4
E1BJG3	GEMIN5	Uncharacterized protein	168.8	1.2	2	3	1.1	2	3
E1BEJ3	GFM1	Uncharacterized protein	83.842	21.8	15	18	3	3	4
A6QNM2	GFM2	Ribosome-releasing factor 2,	86.03	14.3	10	18	5.8	6	8
F1MZY2	GFPT1	Uncharacterized protein (Fragment)	63.502	61.4	29	121	27.4	14	39
F6RN30	GGA1	Uncharacterized protein	69.738	7.3	3	5	2.7	2	4
F1MKY0	GGA2	Uncharacterized protein (Fragment)	65.965	7.2	3	3	#N/A	#N/A	#N/A
G5E690	GGA3	Uncharacterized protein	62.246	3.3	1	1	#N/A	#N/A	#N/A
Q32LE4	GGCT	Gamma-glutamylcyclotransferase	21.177	5.3	1	1	#N/A	#N/A	#N/A
A7YWG4	GGH	Gamma-glutamyl hydrolase	35.683	15.1	5	11	8.5	3	9
P56966	GGPS1	Geranylgeranyl pyrophosphate	34.9	32	9	29	13.3	4	15
F1MRP0	GHDC	Uncharacterized protein	57.621	19.1	8	11	15.7	7	21
Q3SZK3	GHITM	Growth hormone inducible transmembrane protein	37.029	5.8	2	2	2.9	1	4
F1MBG6	GIT1	Uncharacterized protein	83.936	14.6	8	12	8.3	7	14
E1BI41	GIT2	Uncharacterized protein	84.728	5.9	4	5	3.4	3	5
P18246	GJA1	Gap junction alpha-1 protein	43.187	8.6	3	4	12.8	4	5
A4IFL1	GJA4	Gap junction alpha-4 protein	37.499	19.5	6	12	11.4	4	6
E1B725	GLA	Uncharacterized protein	49.795	16.9	5	6	13.7	5	6
O18756	GLCE	D-glucuronyl C5-epimerase	69.984	23.2	14	20	10.2	7	15
E1BJQ1	GLDC	Uncharacterized protein	112.89	7.9	6	7	5.5	6	9
E1BDY3	GLG1	Uncharacterized protein	134.63	6.8	9	12	7.6	11	17
E1BA27	GLMN	Uncharacterized protein	67.689	37.5	20	47	15.9	9	18
A4FUZ1	GLO1	GLO1 protein	20.765	59.8	11	44	22.3	4	10
E1BBM1	GLOD4	Uncharacterized protein	33.23	33.6	11	29	22.5	7	20

				Biological Replicate 1			Biological Replicate 2		
Accession number	Gene symbol	Discription	MW(kDa)	Sequence coverage(%)	Unique peptides	MS/MS count	Sequence coverage(%)	Unique peptides	MS/MS count
P10575	GLRX	Glutaredoxin-1	11.783	36.8	1	5	18.9	1	2
Q58DA7	GLRX3	Glutaredoxin-3	37.297	12.3	3	5	5.7	2	3
A6QLZ6	GLRX5	GLRX5 protein	16.775	12	1	1	#N/A	#N/A	#N/A
E1BHZ6	GLS2	Uncharacterized protein	63.507	15.5	7	11	2.8	2	3
Q5E9E7	GLT8D1	Glycosyltransferase 8 domain-containing protein 1	41.958	11.1	4	5	2.4	1	2
Q3MHF5	GLTP	GLTP protein	19.232	18.9	2	2	5.3	1	1
P00366	GLUD1	Glutamate dehydrogenase 1,	61.511	34.1	19	44	20.6	10	27
A4FUF0	GLYR1	Putative oxidoreductase GLYR1	60.556	27.8	11	21	6.7	3	7
E1BI28	GM2A	Uncharacterized protein	20.903	4.1	1	1	#N/A	#N/A	#N/A
F1MBF1	GMDS	Uncharacterized protein (Fragment)	16.033	45.1	5	6	19.7	3	5
P60984	GMFB	Glia maturation factor beta	16.713	14.1	2	3	#N/A	#N/A	#N/A
Q56JZ9	GMFG	Glia maturation factor gamma	16.77	#N/A	#N/A	#N/A	6.3	1	1
E1BEN4	GMPPA	Uncharacterized protein	46.207	29.8	9	22	8.1	3	8
Q2YDJ9	GMPPB	Mannose-1-phosphate	39.837	31.4	9	16	14.4	5	13
F1MC86	GMPS	Uncharacterized protein (Fragment)	76.854	52.2	28	75	19.2	13	29
G5E6P3	GNA13	Uncharacterized protein (Fragment)	33.383	32	9	13	25	8	12
P38408	GNA14	Guanine nucleotide-binding protein subunit alpha-14	41.498	#N/A	#N/A	#N/A	5.6	1	4
P63097	GNAI1	Guanine nucleotide-binding protein G(i) subunit alpha-1	40.361	28	2	3	17.8	1	1
A7MBH9	GNAI2	GNAI2 protein	40.477	45.1	5	25	#N/A	#N/A	#N/A
Q3ZCA7	GNAI3	Guanine nucleotide binding protein (G protein), alpha inhibiting activity polypeptide 3	40.536	39.3	4	8	22	2	34
P08239	GNAO1	Guanine nucleotide-binding protein G(o) subunit alpha	40.066	20.1	3	4	14.4	3	4
P62871	GNB1	Guanine nucleotide-binding protein G(I)/G(S)/G(T) subunit beta-1	37.377	46.8	12	29	27.6	4	50
P11017	GNB2	Guanine nucleotide-binding protein G(I)/G(S)/G(T) subunit beta-2	37.331	#N/A	#N/A	#N/A	21.8	2	2

				Biological Replicate 1			Biological Replicate 2		
Accession number	Gene symbol	Discription	MW(kDa)	Sequence coverage(%)	Unique peptides	MS/MS count	Sequence coverage(%)	Unique peptides	MS/MS count
P63243	GNB2L1	Guanine nucleotide-binding protein subunit beta-2-like 1	35.076	65	16	36	27.8	8	22
Q28024	GNG12	Guanine nucleotide-binding protein G(I)/G(S)/G(O) subunit gamma-12	8.0572	23.6	1	2	#N/A	#N/A	#N/A
F1MWB9	GNL1	Uncharacterized protein	54.815	23.5	11	23	7.9	4	5
F1N1S9	GNMT	Glycine N-methyltransferase	32.879	10.8	2	3	#N/A	#N/A	#N/A
A4IF87	GNPAT	Dihydroxyacetone phosphate	77.663	29.3	19	31	10.4	8	11
Q17QL1	GNPDA2	Glucosamine-6-phosphate isomerase 2	31.164	85.1	16	57	18.8	5	5
Q1LZH9	GNS	N-acetylglucosamine-6-sulfatase	62.775	29.1	14	44	16.4	9	45
E1BLT0	GOLGA2	Uncharacterized protein	113.17	3.6	3	4	2.1	2	4
E1B970	GOLGA3	Uncharacterized protein	167.46	6.9	8	9	9.2	13	18
E1B7E3	GOLGA4	Uncharacterized protein	259.18	#N/A	#N/A	#N/A	0.7	2	3
A5D7A5	GOLGA5	GOLGA5 protein	82.678	3.4	2	7	#N/A	#N/A	#N/A
G3N3R5	GOLIM4	Uncharacterized protein	77.472	11.8	9	11	8.3	6	14
E1BLA8	GOLM1	Uncharacterized protein	45.536	4.2	2	2	11.6	5	7
Q1RMW9	GOLPH3	Golgi phosphoprotein 3 (Coat-	33.725	15.8	4	5	5	2	3
E1BAX1	GOPC	Uncharacterized protein	51.02	18.9	7	12	12.5	6	7
F1MSB5	GORASP2	Uncharacterized protein	47.577	18.7	5	16	4.4	2	5
G5E571	GOSR1	Golgi SNAP receptor complex member 1 (Fragment)	27.919	20.5	6	11	12.7	4	9
F1N652	GOSR2	Golgi SNAP receptor complex member	24.677	#N/A	#N/A	#N/A	5.2	1	3
P33097	GOT1	Aspartate aminotransferase,	46.398	59.1	24	51	31.5	12	28
P12344	GOT2	Aspartate aminotransferase,	47.513	67.2	31	160	41.6	23	108
Q58CZ3	GPAA1	Anchor attachment protein 1	61.988	10	6	6	6.3	4	7
F1MCX1	GPC4	Uncharacterized protein (Fragment)	56.92	6.9	3	5	6.9	3	6
A6QQR7	GPD1L	GPD1L protein	38.238	11.1	4	4	10	4	5
E1BN14	GPKOW	Uncharacterized protein	53.443	3.3	1	3	#N/A	#N/A	#N/A
P80109	GPLD1	Phosphatidylinositol-glycan-specific phospholipase D	92.601	#N/A	#N/A	#N/A	1.1	1	2
A6H7F2	GPN2	GPN-loop GTPase 2	34.464	5.2	1	1	#N/A	#N/A	#N/A
F1MT29	GPR107	Uncharacterized protein	62.335	1.8	1	2	2.7	2	3
E1B9D3	GPR179	Uncharacterized protein	255.4	0.4	1	2	0.4	1	10

				Biological Replicate 1			Biological Replicate 2		
Accession number	Gene symbol	Discription	MW(kDa)	Sequence coverage(%)	Unique peptides	MS/MS count	Sequence coverage(%)	Unique peptides	MS/MS count
F1N5D2	GPR180	Uncharacterized protein	49.301	#N/A	#N/A	#N/A	2	1	1
F1MP10	GPS1	Uncharacterized protein (Fragment)	54.15	34.5	14	29	12.8	6	13
E1BF40	GPT2	Uncharacterized protein	58.027	5	2	3	3.6	2	3
P00435	GPX1	Glutathione peroxidase 1	22.659	60	11	240	33.7	7	16
G3X8D7	GPX3	Glutathione peroxidase	25.341	10.7	2	3	#N/A	#N/A	#N/A
Q2NL01	GPX8	Probable glutathione peroxidase 8	23.983	39.7	6	10	26.8	6	25
Q3T0F9	GRB2	Growth factor receptor-bound	25.206	#N/A	#N/A	#N/A	11.1	3	4
F1MB84	GRHPR	Uncharacterized protein	35.769	57.9	19	79	20.1	9	33
E1BPP9	GRIPAP1	Uncharacterized protein	96.233	3.1	2	2	#N/A	#N/A	#N/A
Q3SZC1	GRPEL1	GrpE protein homolog 1,	24.306	44.2	9	19	34.6	9	15
Q0P5N5	GRPEL2	GrpE protein homolog 2,	25.119	#N/A	#N/A	#N/A	11.2	3	5
F6Q087	GSPT1	G1 to S phase transition 1	66.015	33.4	16	28	12.9	9	19
E1BKZ1	GSR	Uncharacterized protein (Fragment)	46.238	65.7	24	98	36.2	15	63
Q5EAC2	GSS	Glutathione synthetase	52.065	19.4	8	16	3.4	2	3
Q28035	GSTA1	Glutathione S-transferase A1	25.451	7.7	2	2	#N/A	#N/A	#N/A
F1MX44	GSTA5	Glutathione S-transferase	25.368	#N/A	#N/A	#N/A	7.7	2	4
F1MRG0	GSTK1	Uncharacterized protein	25.666	3.5	1	2	#N/A	#N/A	#N/A
Q9N0V4	GSTM1	Glutathione S-transferase Mu 1	25.635	46.3	1	15	#N/A	#N/A	#N/A
F6Q751	GSTM2	Uncharacterized protein	25.656	46.3	2	4	25.2	3	32
F1MUX6	GSTM3	Uncharacterized protein (Fragment)	25.067	84.7	24	671	78	18	196
P28801	GSTP1	Glutathione S-transferase P	23.613	85.2	13	109	30.5	6	44
F1MSE9	GTF2A1L	Uncharacterized protein	42.904	22.9	7	10	#N/A	#N/A	#N/A
Q32L01	GTF2A2	Transcription initiation factor IIA subunit 2	12.457	14.7	2	2	14.7	2	3
A6QLI8	GTF2E1	General transcription factor IIE	49.21	6.2	3	5	#N/A	#N/A	#N/A
Q2KJF9	GTF2E2	General transcription factor IIE	32.731	5.5	2	3	#N/A	#N/A	#N/A
F1MYY8	GTF3C5	Uncharacterized protein	60.076	5.2	2	2	#N/A	#N/A	#N/A
Q3MHG6	GTPBP10	GTP-binding protein 10	42.931	18.1	6	8	4.7	2	2
A6QLJ3	GUF1	Translation factor GUF1,	74.765	4.2	2	3	2.8	2	3
P46195	GUK1	Guanylate kinase	21.91	36.9	6	13	9.6	2	3
A3KMY8	GUSB	GUSB protein	74.469	38.7	23	61	13.2	11	26
E1BHD8	GXYLT1	Uncharacterized protein	50.689	6.6	3	6	3	1	1

				Biological Replicate 1			Biological Replicate 2		
Accession number	Gene symbol	Discription	MW(kDa)	Sequence coverage(%)	Unique peptides	MS/MS count	Sequence coverage(%)	Unique peptides	MS/MS count
F6QLM5	GYG1	Uncharacterized protein (Fragment)	37.298	36	10	27	11.2	4	11
F6QC58	GYPC	Uncharacterized protein	9.9012	#N/A	#N/A	#N/A	10	1	1
A7MB78	GYS1	Glycogen [starch] synthase, muscle	83.813	8	5	7	4.1	3	7
Q3HNG7	H1FOO	Histone H1oo	35.435	23.9	9	20	14.6	5	12
Q17QG8	H2AFX	Histone H2A	15.128	17.5	3	6	17.5	3	7
Q32S29	H2B	Histone H2B	13.926	46.8	4	10	34.1	1	13
Q5E9F8	H3F3A;H3F3B	Histone H3.3	15.328	31.6	2	11	17.6	2	12
A2VDN1	HABP4	Hyaluronan binding protein 4	46.017	9.4	3	4	1.9	1	2
H9GW30	HACE1	E3 ubiquitin-protein ligase HACE1	100.98	6.3	5	5	4.2	4	9
F1MVP8	HACL1	Uncharacterized protein	63.577	16.9	7	11	9.8	6	9
F1N338	HADH	Uncharacterized protein	34.39	42.4	15	41	17.2	7	19
Q3SZ00	HADHA	HADHA protein	83.248	49.1	32	116	27.4	20	101
O46629	HADHB	Trifunctional enzyme subunit beta, mitochondrial	51.345	63.8	25	92	25.3	13	70
F6QLF1	HAPLN3	Uncharacterized protein	41.127	28.9	10	27	21.4	8	29
Q2KI84	HARS	Histidine--tRNA ligase, cytoplasmic	57.285	33.2	10	21	18.1	5	19
F1N0T6	HARS2	Probable histidine--tRNA ligase, mitochondrial	56.88	11.5	2	4	#N/A	#N/A	#N/A
Q58DR1	HAT1	Histone acetyltransferase 1	48.836	34.6	11	16	6.3	2	4
E1BG87	HAUS6	Uncharacterized protein	107.72	#N/A	#N/A	#N/A	1.2	1	2
Q2KIE2	HAX1	HCLS1-associated protein X-1	31.415	3.2	1	1	#N/A	#N/A	#N/A
Q2KHZ2	HBS1L	HBS1-like protein	75.849	21.9	13	18	6.6	6	8
Q0VD49	HDAC10	Histone deacetylase 10	71.492	1.8	1	2	#N/A	#N/A	#N/A
F1MQP3	HDAC6	Uncharacterized protein	123.82	2.3	2	3	#N/A	#N/A	#N/A
Q2KJ86	HDHD1A	Haloacid dehalogenase-like hydrolase domain containing 1A	25.298	26.8	4	4	#N/A	#N/A	#N/A
Q3ZCH9	HDHD2	Haloacid dehalogenase-like hydrolase domain-containing protein	28.594	57.1	10	36	7.7	2	6
F1MT25	HDLBP	Uncharacterized protein	139.59	6.8	8	9	4.9	6	9
G3N314	HEATR2	Uncharacterized protein (Fragment)	92.048	4.3	2	2	#N/A	#N/A	#N/A
E1BHR5	HEATR6	Uncharacterized protein (Fragment)	130.08	2.1	2	2	#N/A	#N/A	#N/A
Q148C9	HEBP1	Heme-binding protein 1	21.23	44.5	6	9	26.7	5	7

				Biological Replicate 1			Biological Replicate 2		
Accession number	Gene symbol	Discription	MW(kDa)	Sequence coverage(%)	Unique peptides	MS/MS count	Sequence coverage(%)	Unique peptides	MS/MS count
E1BLD1	HECTD1	Uncharacterized protein	289.34	#N/A	#N/A	#N/A	0.3	1	1
E1BCV0	HELLS	Uncharacterized protein	94.816	2.5	2	2	#N/A	#N/A	#N/A
E1BAV2	HERC4	Uncharacterized protein	118.59	6.5	6	6	1.1	1	1
Q0V8R6	HEXA	Beta-hexosaminidase subunit alpha	60.352	20	10	19	4.2	3	8
H7BWW2	HEXB	Beta-hexosaminidase	61.261	3.5	2	3	#N/A	#N/A	#N/A
Q0V8S0	HGS	Hepatocyte growth factor-regulated tyrosine kinase substrate	85.785	1.9	2	4	3.9	4	5
F1MF45	HGSNAT	Uncharacterized protein (Fragment)	66.076	6.8	4	7	6.4	4	8
Q2HJD7	HIBADH	3-hydroxyisobutyrate dehydrogenase, mitochondrial	35.41	47.9	13	74	31.2	7	28
Q2HJ73	HIBCH	3-hydroxyisobutyryl-CoA hydrolase, mitochondrial	43.349	40.9	16	63	16.6	8	29
Q0II23	HICE1	HEC1/NDC80 interacting, centrosome associated 1	34.728	3.9	1	1	#N/A	#N/A	#N/A
F1MG82	HIF1AN	Uncharacterized protein	40.361	10	2	6	#N/A	#N/A	#N/A
Q05AT5	HIGD2A	HIG1 domain family, member 2A	11.575	23.6	1	2	#N/A	#N/A	#N/A
P62958	HINT1	Histidine triad nucleotide-binding protein 1	13.779	78.6	6	14	17.5	2	8
Q8SQ21	HINT2	Histidine triad nucleotide-binding protein 2, mitochondrial	17.147	47.2	5	10	28.8	4	11
F1MWF0	HIP1	Uncharacterized protein	116.49	11.8	11	14	4	3	5
F1MUI0	HIP1R	Uncharacterized protein (Fragment)	120.34	5.3	6	7	6.5	7	10
Q5W5U3	HK1	Hexokinase 1	102.21	21.2	22	37	16.7	13	25
E1BME6	HK2	Uncharacterized protein (Fragment)	102.08	#N/A	#N/A	#N/A	3.5	1	2
F1MYW7	HM13	Uncharacterized protein	41.552	14.6	4	18	7.4	3	4
Q2KIN5	HMBS	Porphobilinogen deaminase	39.515	4.7	2	2	6.1	2	2
P10103	HMGB1	High mobility group protein B1	24.908	12.6	1	1	#N/A	#N/A	#N/A
P40673	HMGB2	High mobility group protein B2	24.033	16.3	2	6	#N/A	#N/A	#N/A
Q32L31	HMGB3	High mobility group protein B3	22.896	#N/A	#N/A	#N/A	18	3	5
Q29448	HMGCL	Hydroxymethylglutaryl-CoA lyase, mitochondrial	34.168	30.5	8	18	9.5	4	10
F6RJG0	HMGCS1	Uncharacterized protein (Fragment)	62.517	20.4	10	17	10.8	6	15

				Biological Replicate 1			Biological Replicate 2		
Accession number	Gene symbol	Discription	MW(kDa)	Sequence coverage(%)	Unique peptides	MS/MS count	Sequence coverage(%)	Unique peptides	MS/MS count
F1MTY9	HMOX2	Heme oxygenase (Decycling) 2	37.555	45.2	13	27	17.1	6	11
P09867	HNRNPA1	Heterogeneous nuclear	34.196	18.4	4	6	#N/A	#N/A	#N/A
Q2HJ60	HNRNPA2B1	Heterogeneous nuclear ribonucleoproteins A2/B1	36.006	25.5	7	11	19.4	6	9
E1BEG2	HNRNPA3	Uncharacterized protein	39.65	20.5	6	11	#N/A	#N/A	#N/A
Q3ZC44	HNRNPAB	Heterogeneous nuclear ribonucleoprotein A/B	30.653	10.5	3	7	#N/A	#N/A	#N/A
Q5E9J1	HNRNPF	Heterogeneous nuclear	45.688	44.9	10	25	14.3	4	12
E1BF20	HNRNPH1	Uncharacterized protein	51.25	13.8	1	4	5.7	1	6
Q3SZF3	HNRNPH2	Heterogeneous nuclear	49.264	13.6	2	2	#N/A	#N/A	#N/A
E1BB17	HNRNPH3	Uncharacterized protein	36.926	18.8	4	5	8.4	2	2
Q3T0D0	HNRNPK	Heterogeneous nuclear	51.019	22.4	8	11	19.4	9	20
E1BIB4	HNRNPL	Uncharacterized protein	64.075	13.8	8	12	6.5	4	8
F1MY44	HNRNPM	Uncharacterized protein	77.482	12.7	8	12	5.1	4	5
A3KMV6	HNRNPR	HNRNPR protein	70.955	10.3	2	4	#N/A	#N/A	#N/A
F1MFT4	HNRNPU	Uncharacterized protein (Fragment)	77.551	8.3	6	13	4.3	4	7
Q3SX47	HNRPC	Heterogeneous nuclear ribonucleoprotein C (C1/C2)	32.427	18.4	6	8	6.8	2	4
A5D9H5	HNRPD	Heterogeneous nuclear	32.848	24.8	7	10	7.8	2	5
E1BAT1	HOOK1	Uncharacterized protein	84.822	1.2	1	1	3.4	3	6
E1BI15	HOOK2	Uncharacterized protein	82.877	#N/A	#N/A	#N/A	1.8	1	2
E1B7Q0	HOOK3	Protein Hook homolog 3	83.145	15.5	10	16	6.5	4	6
Q2TBU0	HP	Haptoglobin	44.859	3.5	1	1	#N/A	#N/A	#N/A
Q4PL64	HPCA	Neuron-specific calcium-binding protein hippocalcin	22.427	4.1	1	1	#N/A	#N/A	#N/A
Q3SZ18	HPRT1	Hypoxanthine-guanine phosphoribosyltransferase	24.498	45	7	13	17.4	3	4
G3MXH2	HRAS	Uncharacterized protein	21.272	18.5	3	4	#N/A	#N/A	#N/A
Q5E949	HRMT1L2	HMT1 hnRNP methyltransferase-like 2 isoform 3	40.548	26.9	10	16	9.3	3	6
Q3T114	HRSP12	Ribonuclease UK114	14.272	56.2	6	12	15.3	2	6
F1MLK9	HSCB	Uncharacterized protein	27.026	12.8	4	4	#N/A	#N/A	#N/A

				Biological Replicate 1			Biological Replicate 2		
Accession number	Gene symbol	Discription	MW(kDa)	Sequence coverage(%)	Unique peptides	MS/MS count	Sequence coverage(%)	Unique peptides	MS/MS count
F1N3U0	HSD11B2	Corticosteroid 11-beta-dehydrogenase isozyme 2	43.992	37.1	11	44	20	7	37
Q1JQD0	HSD17B1	Hydroxysteroid (17-beta)	33.974	10.7	4	6	22.4	6	13
O02691	HSD17B10	3-hydroxyacyl-CoA dehydrogenase	27.14	64.8	13	44	28.7	6	24
Q5E9H7	HSD17B12	Estradiol 17-beta-dehydrogenase 12	34.495	9.6	1	2	4.8	1	5
Q0IIL6	HSD17B4	Hydroxysteroid (17-beta)	79.557	39.9	25	55	23.6	15	46
A4FUD2	HSD17B7	HSD17B7 protein	37.66	#N/A	#N/A	#N/A	3.3	1	4
A5PJF6	HSDL1	Inactive hydroxysteroid dehydrogenase-like protein 1	37.013	39.1	11	28	16.1	5	12
F1MF48	HSDL2	Hydroxysteroid dehydrogenase-like protein 2 (Fragment)	45.232	14.1	5	5	6	3	5
Q76LV2	HSP90AA1	Heat shock protein HSP 90-alpha	84.73	69.2	43	692	43.7	27	397
Q76LV1	HSP90AB1	Heat shock protein HSP 90-beta	83.252	56.8	26	100	38	14	102
Q95M18	HSP90B1	Endoplasmin	92.426	65.3	60	507	44.8	39	248
Q2YDD0	HSPA14	Heat shock 70 kDa protein 14	54.929	2.4	1	1	#N/A	#N/A	#N/A
Q27975	HSPA1A	Heat shock 70 kDa protein 1A	70.258	#N/A	#N/A	#N/A	43.7	16	188
Q27965	HSPA1B	Heat shock 70 kDa protein 1B	70.228	66	23	216	#N/A	#N/A	#N/A
P0CB32	HSPA1L	Heat shock 70 kDa protein 1-like	70.388	#N/A	#N/A	#N/A	25.9	1	1
P34933	HSPA2	Heat shock-related 70 kDa protein 2	69.739	52.7	17	81	45.4	17	82
E1BBY7	HSPA4	Uncharacterized protein	94.508	60.1	46	184	38.9	29	102
E1BBT9	HSPA4L	Uncharacterized protein (Fragment)	97.591	54	42	110	29.8	22	55
Q0VCX2	HSPA5	78 kDa glucose-regulated protein	72.399	62.3	47	418	55.6	40	422
F1MWU9	HSPA6	Uncharacterized protein	70.955	17.4	2	27	19.3	3	22
P19120	HSPA8	Heat shock cognate 71 kDa protein	71.24	60.9	31	341	56	26	353
Q3ZCH0	HSPA9	Stress-70 protein, mitochondrial	73.741	56.4	38	126	42.7	29	129
Q3T149	HSPB1	Heat shock protein beta-1	22.393	54.2	8	18	50.7	9	27
Q2KJ77	HSPBP1	HSPA (Heat shock 70kDa) binding protein, cytoplasmic cochaperone 1	39.219	56.6	15	59	11.2	4	11
F1MUZ9	HSPD1	60 kDa heat shock protein,	60.977	68.8	39	207	45	28	128
P61603	HSPE1	10 kDa heat shock protein,	10.932	70.6	9	31	57.8	7	24
Q0IIM3	HSPH1	Heat shock protein 105 kDa	96.725	57.2	37	123	29.9	22	73
Q3SX02	HTATIP2	HIV-1 Tat interactive protein 2,	27.132	73.6	19	41	44.6	12	50

				Biological Replicate 1			Biological Replicate 2		
Accession number	Gene symbol	Discription	MW(kDa)	Sequence coverage(%)	Unique peptides	MS/MS count	Sequence coverage(%)	Unique peptides	MS/MS count
G3N370	HTATSF1	Uncharacterized protein	86.103	2.5	2	2	#N/A	#N/A	#N/A
F1N152	HTRA1	Serine protease HTRA1	51.906	28.5	10	18	5.5	3	4
A0JNK3	HTRA2	Serine protease HTRA2,	49.163	2.2	1	2	2.8	1	1
E1BNY9	HUWE1	Uncharacterized protein	482.19	#N/A	#N/A	#N/A	0.6	3	3
F1MEW9	HVCN1	Uncharacterized protein (Fragment)	31.139	#N/A	#N/A	#N/A	12.4	3	5
E1B748	HYOU1	Uncharacterized protein	111.68	51.7	51	229	32.1	33	152
Q3SZ16	IAH1	Isoamyl acetate-hydrolyzing esterase 1 homolog	27.578	11.2	2	4	#N/A	#N/A	#N/A
A7MBC5	IARS	IARS protein	144.64	31	34	72	9.9	14	33
G3MWG4	IARS2	Uncharacterized protein (Fragment)	103.67	29.7	24	60	17.2	14	28
E1BC94	IBA57	Uncharacterized protein	38.231	19.6	5	7	9.8	3	3
E1BF83	ICA1L	Uncharacterized protein	54.059	5.6	3	5	#N/A	#N/A	#N/A
G3N1V4	ICMT	Uncharacterized protein (Fragment)	32.103	3.5	2	3	#N/A	#N/A	#N/A
Q3T116	ICT1	Peptidyl-tRNA hydrolase ICT1, mitochondrial	23.306	10.2	2	2	#N/A	#N/A	#N/A
Q24K02	IDE	Insulin-degrading enzyme	118.1	4.1	4	4	#N/A	#N/A	#N/A
Q9XSG3	IDH1	Isocitrate dehydrogenase [NADP] cytoplasmic	46.785	59.4	23	63	30.9	14	44
F1MN74	IDH3A	Isocitrate dehydrogenase [NAD] subunit alpha, mitochondrial	39.669	48.9	14	56	25.7	10	43
O77784	IDH3B	Isocitrate dehydrogenase [NAD] subunit beta, mitochondrial	42.497	28.3	10	26	#N/A	#N/A	#N/A
Q58D96	IDH3G	Isocitrate dehydrogenase 3 (NAD+) gamma isoform a	42.392	35.6	10	23	16.8	7	15
Q1LZ95	IDI1	Isopentenyl-diphosphate Delta-	26.467	36.1	8	21	13.7	3	5
E1BJZ0	IFIH1	Uncharacterized protein	115.34	#N/A	#N/A	#N/A	1.3	1	4
E1BFI4	IGF2BP2	Uncharacterized protein	66.009	38.4	17	39	19.5	9	18
F1MPB2	IGF2BP3	Uncharacterized protein	63.856	50.2	26	94	45.5	22	138
F1MTR4	IGSF8	Uncharacterized protein	64.416	11	5	9	6.7	4	5
Q95KV0	IKBKB	Inhibitor of nuclear factor kappa-B kinase subunit beta	86.646	12.4	9	16	1.5	1	1
Q95KU9	IKBKG	NF-kappa-B essential modulator	48.569	3.8	1	1	#N/A	#N/A	#N/A

				Biological Replicate 1			Biological Replicate 2		
Accession number	Gene symbol	Discription	MW(kDa)	Sequence coverage(%)	Unique peptides	MS/MS count	Sequence coverage(%)	Unique peptides	MS/MS count
A6QNQ9	ILDR1	ILDR1 protein	63.378	2.2	1	1	#N/A	#N/A	#N/A
F2Z4E7	ILF2	Uncharacterized protein	43.062	10	4	6	4.1	1	2
F1MMA0	ILF3	Uncharacterized protein	95.392	5.5	5	9	3.2	3	5
Q0IIF0	ILKAP	Integrin-linked kinase-associated serine/threonine phosphatase 2C	40.621	3.2	1	1	#N/A	#N/A	#N/A
A6QQT9	ILVBL	Acetolactate synthase-like protein	68.109	11.7	5	9	6.2	3	3
F1MXX0	IMMT	Uncharacterized protein	83.043	58.3	46	150	46.6	36	164
P20456	IMPA1	Inositol monophosphatase 1	30.055	49.5	17	49	36.1	12	50
Q2KJ53	IMPAD1	Inositol monophosphatase 3	38.923	4.1	2	2	#N/A	#N/A	#N/A
A0JNA3	IMPDH1	Inosine-5-monophosphate	55.423	3.7	2	2	#N/A	#N/A	#N/A
Q3SWY3	IMPDH2	Inosine-5-monophosphate	55.762	14.6	5	8	8.9	4	7
P07994	INHA	Inhibin alpha chain	38.809	6.9	2	5	2.8	1	3
P21327	INPP1	Inositol polyphosphate 1-	43.965	51.5	18	46	22.2	9	20
F1MCQ6	INPP5F	Uncharacterized protein (Fragment)	58.198	11.6	6	10	11.6	6	9
E1BBJ7	INPPL1	Uncharacterized protein	139	#N/A	#N/A	#N/A	1	1	1
F1MC23	INSRR	Tyrosine-protein kinase receptor	144.55	0.9	2	4	#N/A	#N/A	#N/A
E1BN70	INTS3	Uncharacterized protein	117.89	3.4	2	3	#N/A	#N/A	#N/A
Q2KJA6	INTS9	Integrator complex subunit 9	73.837	18.4	11	14	#N/A	#N/A	#N/A
E1BEW3	IPO11	Uncharacterized protein	112.24	7.3	6	8	#N/A	#N/A	#N/A
A7YWD2	IPO13	Importin-13	108.14	4.3	4	4	1.1	1	1
F1MPE5	IPO5	Uncharacterized protein (Fragment)	125.02	50.6	44	113	13.8	15	45
E1BGE5	IPO7	Uncharacterized protein	119.53	23	19	42	6.5	6	10
G5E567	IPO9	Uncharacterized protein (Fragment)	110.01	4.6	4	4	#N/A	#N/A	#N/A
Q29RS3	IQCB1	IQ motif containing B1	68.507	1.7	1	1	#N/A	#N/A	#N/A
F1MC48	IQGAP1	Uncharacterized protein	182.78	6.6	7	9	2.1	4	5
F1MTR1	IQGAP2	Uncharacterized protein	178.5	14.1	18	24	4.7	7	10
F1MYE9	IQSEC1	Uncharacterized protein (Fragment)	120.06	1.6	1	1	#N/A	#N/A	#N/A
Q1RMT8	IRAK4	Interleukin-1 receptor-associated	51.574	14.1	6	6	7.2	3	4
Q4JF28	IRF3	Interferon regulatory factor 3	46.645	#N/A	#N/A	#N/A	2.2	1	1
Q08DD6	IRF6	Interferon regulatory factor 6	53.104	5.8	2	2	#N/A	#N/A	#N/A
Q3SZG8	ISCA1	Iron-sulfur cluster assembly 1 homolog, mitochondrial	14.179	9.3	1	1	9.3	1	2

				Biological Replicate 1			Biological Replicate 2		
Accession number	Gene symbol	Discription	MW(kDa)	Sequence coverage(%)	Unique peptides	MS/MS count	Sequence coverage(%)	Unique peptides	MS/MS count
Q2TBG7	ISCA2	Iron-sulfur cluster assembly 2 homolog, mitochondrial	16.435	#N/A	#N/A	#N/A	15.8	2	2
Q17QE6	ISCU	Iron-sulfur cluster scaffold homolog (E. coli)	17.852	9	2	2	9.6	2	3
A6QLY4	ISOC1	Isochorismatase domain-containing protein 1	32.115	37.2	11	20	19.8	7	13
E1BCH6	ISPD	Isoprenoid synthase domain-containing protein	49.51	7	3	3	13	6	10
Q3ZBV1	IST1	IST1 homolog	39.79	26.9	10	20	13.5	5	16
Q2NL29	ISYNA1	Inositol-3-phosphate synthase 1	60.761	17.6	9	16	4.7	3	3
F1MGQ5	ITCH	E3 ubiquitin-protein ligase	98.64	14.3	8	15	2.9	3	4
Q2HJE5	ITFG3	Protein ITFG3	58.766	1.8	1	1	#N/A	#N/A	#N/A
P53710	ITGA2	Integrin alpha-2 (Fragment)	128.93	7.6	8	12	4.7	5	9
E1BFQ6	ITGA6	Uncharacterized protein	119.23	22.5	20	39	8.6	10	30
P80746	ITGAV	Integrin alpha-V	116.13	15	15	27	8.1	10	16
P53712	ITGB1	Integrin beta-1	88.093	15.8	9	20	4.9	4	15
F1MTN1	ITGB3	Integrin beta	86.536	6	4	4	2.8	2	4
E1B864	ITGB4	Integrin beta	200.31	11.9	17	22	7.1	12	22
F1MK32	ITGB5	Integrin beta (Fragment)	82.596	11.6	7	10	2.7	2	9
Q2KIC5	ITPA	Inosine triphosphate	23.061	18.8	3	7	7.7	2	4
P0C0T1	ITPK1	Inositol-tetrakisphosphate 1-kinase	45.842	8.1	2	3	#N/A	#N/A	#N/A
F1MX21	ITPR1	Inositol 1,4,5-trisphosphate receptor type 1 (Fragment)	302.89	3.7	7	13	4.7	13	15
Q8WN95	ITPR3	Inositol 1,4,5-trisphosphate receptor type 3	303.04	1.7	2	3	#N/A	#N/A	#N/A
F1MC50	ITSN2	Uncharacterized protein (Fragment)	190.87	6.3	9	12	4	7	10
Q3SZI8	IVD	Isovaleryl-CoA dehydrogenase, mitochondrial	46.497	34.7	12	26	14.6	7	20
Q2NKY9	JAGN1	Protein jagunal homolog 1	21.102	#N/A	#N/A	#N/A	6.6	1	2
A6QR54	JAKMIP1	Janus kinase and microtubule-interacting protein 1	73.27	1.4	1	1	#N/A	#N/A	#N/A
A7YW22	JAM3	JAM3 protein	33.824	#N/A	#N/A	#N/A	3.3	1	2

				Biological Replicate 1			Biological Replicate 2		
Accession number	Gene symbol	Discription	MW(kDa)	Sequence coverage(%)	Unique peptides	MS/MS count	Sequence coverage(%)	Unique peptides	MS/MS count
F1MEH3	JMJD7	Uncharacterized protein	36.043	29.1	6	11	7.3	2	3
F1MPX6	JMJD8	Uncharacterized protein (Fragment)	32.483	#N/A	#N/A	#N/A	7.7	2	3
F1ME39	JOSD2	Uncharacterized protein (Fragment)	22.663	4.8	1	1	#N/A	#N/A	#N/A
Q8SPJ1	JUP	Junction plakoglobin	81.82	8.6	2	2	#N/A	#N/A	#N/A
F1MMK8	KARS	Lysine--tRNA ligase	71.346	31.5	20	40	17.7	13	38
E1BH39	KATNA1	Katanin p60 ATPase-containing	55.92	4.9	2	4	#N/A	#N/A	#N/A
E1BHF2	KATNAL1	Uncharacterized protein	55.382	4.7	1	1	#N/A	#N/A	#N/A
E1BAN2	KATNAL2	Uncharacterized protein (Fragment)	59.893	2.2	1	2	2.2	1	12
A6QQU1	KATNB1	Katanin p80 WD40 repeat-containing subunit B1	73.018	2.4	1	1	#N/A	#N/A	#N/A
A7MB51	KBTBD2	KBTBD2 protein	53.575	#N/A	#N/A	#N/A	4.1	2	2
G3N1I7	KBTBD7	Uncharacterized protein	76.741	#N/A	#N/A	#N/A	1.5	1	1
F1N465	KBTBD8	Uncharacterized protein	68.882	9.3	5	6	3.5	2	2
Q1LZE1	KCMF1	E3 ubiquitin-protein ligase KCMF1	41.947	4.5	1	1	2.9	1	2
A6QLV6	KCTD10	KCTD10 protein	35.717	3.5	2	4	#N/A	#N/A	#N/A
Q2T9W0	KCTD13	BTB/POZ domain-containing adapter for CUL3-mediated RhoA degradation	36.425	3	1	1	#N/A	#N/A	#N/A
A5PKG7	KCTD5	BTB/POZ domain-containing protein	25.986	5.1	1	2	#N/A	#N/A	#N/A
A6QLT0	KCTD6	KCTD6 protein	27.578	5.9	1	1	#N/A	#N/A	#N/A
F1MZX7	KDELC2	Uncharacterized protein (Fragment)	59.462	1.5	1	1	#N/A	#N/A	#N/A
Q2KJ37	KDELR2	ER lumen protein retaining receptor	24.454	12.7	2	4	3.8	1	4
F1MBS5	KDM1A	Uncharacterized protein	94.564	15.7	11	17	2.9	3	4
E1BPZ5	KDM1B	Uncharacterized protein	91.988	1.5	1	1	#N/A	#N/A	#N/A
F1MLE5	KDSR	3-ketodihydrosphingosine reductase (Fragment)	34.905	7.8	2	2	#N/A	#N/A	#N/A
E1BPE9	KHDC3L	Uncharacterized protein	24.933	44.9	9	89	36.4	7	88
Q29RQ2	KHDRBS1	KH domain containing, RNA binding, signal transduction associated 1	48.207	#N/A	#N/A	#N/A	4.1	2	4
F1MHR6	KHSRP	Uncharacterized protein (Fragment)	73.523	1.4	1	1	#N/A	#N/A	#N/A
F1MKJ5	KIAA0196	Uncharacterized protein (Fragment)	134.81	31.5	37	65	18.4	22	39
F1ML49	KIAA0319L	Uncharacterized protein (Fragment)	115.9	5.1	6	7	3.6	4	6
F1MGK9	KIAA0368	Uncharacterized protein	203.94	6.9	12	14	0.9	2	3

				Biological Replicate 1			Biological Replicate 2		
Accession number	Gene symbol	Discription	MW(kDa)	Sequence coverage(%)	Unique peptides	MS/MS count	Sequence coverage(%)	Unique peptides	MS/MS count
E1BFB5	KIAA0391	Uncharacterized protein	67.73	5	3	5	#N/A	#N/A	#N/A
G5E600	KIAA1033	Uncharacterized protein	136.5	18.8	23	36	8.9	11	15
E1BJ48	KIAA1045	Uncharacterized protein	45.111	3.5	1	1	#N/A	#N/A	#N/A
F1MN85	KIAA1147	Uncharacterized protein (Fragment)	42.137	17.4	5	16	3.5	1	1
F1MLS8	KIAA1217	Uncharacterized protein	199.17	#N/A	#N/A	#N/A	1.7	2	2
E1BH07	KIAA1274	Uncharacterized protein	84.843	1.3	1	6	#N/A	#N/A	#N/A
F1MKR0	KIAA1432	Uncharacterized protein	158.91	2.4	3	4	3.4	4	5
G5E512	KIAA1462	Uncharacterized protein	140.06	#N/A	#N/A	#N/A	1.1	1	2
E1BM42	KIAA1468	Uncharacterized protein	133.96	#N/A	#N/A	#N/A	0.9	1	1
F1MUA7	KIAA1598	Uncharacterized protein (Fragment)	69.025	34.5	22	44	18.9	11	25
F6RBJ1	KIAA1715	Uncharacterized protein	48.143	14.7	6	9	10.7	4	6
E1BMF4	KIDINS220	Uncharacterized protein	196.14	16.8	26	43	6.3	12	16
E1BF29	KIF11	Uncharacterized protein	119	2.7	2	2	2.6	3	3
E1BBY6	KIF12	Uncharacterized protein	70.735	2.8	2	2	#N/A	#N/A	#N/A
E1BGB0	KIF13B	Uncharacterized protein	207.15	4.7	7	9	1.3	3	4
G5E5S3	KIF14	Uncharacterized protein (Fragment)	141.49	#N/A	#N/A	#N/A	1.1	2	3
E1BDH4	KIF18A	Uncharacterized protein	101.69	1	1	1	#N/A	#N/A	#N/A
E1BBN7	KIF1B	Uncharacterized protein	204.53	1.2	3	4	#N/A	#N/A	#N/A
Q29RT6	KIF20A	Kinesin-like protein KIF20A	100.04	13.7	10	11	#N/A	#N/A	#N/A
A5D7N6	KIF23	KIF23 protein	109.08	12.3	10	14	8.7	8	16
F1MNK8	KIF26B	Uncharacterized protein (Fragment)	163.95	1.4	2	2	#N/A	#N/A	#N/A
Q2NL05	KIF2A	Kinesin-like protein KIF2A	74.993	5.8	2	2	#N/A	#N/A	#N/A
A6QPE8	KIF2C	KIF2C protein	81.023	13.7	7	10	6.6	5	6
E1B999	KIF3A	Uncharacterized protein	82.796	11.7	6	8	7.6	4	9
F1N020	KIF3B	Uncharacterized protein	84.908	17.4	11	19	9	7	12
F1MCP5	KIF4A	Uncharacterized protein (Fragment)	140.45	3.1	3	4	#N/A	#N/A	#N/A
F1N1G7	KIF5B	Uncharacterized protein	109.78	25.6	16	37	16.2	12	33
F6RAG5	KIF5C	Uncharacterized protein	109.19	11	4	6	#N/A	#N/A	#N/A
Q3MHI2	KIFAP3	Kinesin-associated protein 3	91.174	10.8	7	9	3.9	2	2
A7MBA1	KIFC1	KIFC1 protein	73.533	#N/A	#N/A	#N/A	3.3	2	2
E1BND1	KIN	Uncharacterized protein	45.217	3.1	1	1	5.6	2	3
P43481	KIT	Mast/stem cell growth factor	109.68	4.4	4	4	5.9	6	10

				Biological Replicate 1			Biological Replicate 2		
Accession number	Gene symbol	Discription	MW(kDa)	Sequence coverage(%)	Unique peptides	MS/MS count	Sequence coverage(%)	Unique peptides	MS/MS count
A6H7H2	KLC1	KLC1 protein	70.35	13.2	3	8	#N/A	#N/A	#N/A
Q08DH4	KLC2	Kinesin light chain 2	68.71	6.8	1	1	6.1	2	6
Q2TBQ9	KLC3	Kinesin light chain 3	55.583	7.1	2	3	1.8	1	2
E1BGH2	KLHDC4	Uncharacterized protein	65.555	5.6	3	4	#N/A	#N/A	#N/A
E1BN42	KLHDC9	Uncharacterized protein	37.519	5.2	2	3	#N/A	#N/A	#N/A
A7MBJ7	KLHL18	KLHL18 protein	64.354	8.6	5	9	6.2	4	6
F1N592	KLHL25	Uncharacterized protein	65.776	25.1	15	26	12.2	8	18
Q0VCQ5	KLHL7	Kelch-like 7 (Drosophila)	65.982	3.6	2	4	4.1	2	3
A5D7H6	KLRAQ1	KLRAQ1 protein	88.353	4.6	4	4	2.8	2	3
E1BBQ0	KNTC1	Uncharacterized protein	254.91	3	7	7	2.5	7	8
Q3SYV6	KPNA2	Importin subunit alpha	57.925	22.3	9	16	3.4	1	2
F1MIZ9	KPNA3	Importin subunit alpha	55.019	15.7	1	1	4.2	2	3
C1JZ67	KPNA4	Importin subunit alpha	57.9	27.8	4	16	#N/A	#N/A	#N/A
G5E536	KPNA6	Importin subunit alpha (Fragment)	59.749	39.8	17	40	16.4	7	21
C1JZ66	KPNA7	Importin subunit alpha-8	57.362	45.4	17	49	13	6	20
E1BFV0	KPNB1	Uncharacterized protein	97.226	44.6	31	137	22.3	18	65
F6S1Q0	KRT18	Uncharacterized protein	47.964	3.7	1	5	5.6	2	7
E1B991	KRT2	Uncharacterized protein	64.333	7.9	2	2	9.6	1	1
A6QNX5	KRT78	Keratin, type II cytoskeletal 78	57.463	#N/A	#N/A	#N/A	3	1	2
G3MX98	KRT9	Uncharacterized protein	54.654	3.1	1	1	#N/A	#N/A	#N/A
F1MLU7	KTN1	Uncharacterized protein	150.66	18	21	33	10.2	14	25
P83095	LACTB	Serine beta-lactamase-like protein LACTB, mitochondrial	61.682	24.8	12	25	11.2	6	10
Q0VCZ7	LAD1	Ladinin 1	50.172	13.5	8	25	22.9	11	36
F1MDD5	LAMA3	Uncharacterized protein (Fragment)	357.11	#N/A	#N/A	#N/A	0.6	2	2
Q05204	LAMP1	Lysosome-associated membrane glycoprotein 1	44.152	12.7	6	18	8.6	4	29
Q3SZJ7	LAMP2	Lysosomal-associated membrane	44.542	12.3	7	16	8.6	5	19
Q3T0D8	LAMTOR1	Ragulator complex protein LAMTOR1	17.731	15.5	2	4	15.5	3	12
Q3T132	LAMTOR2	Ragulator complex protein LAMTOR2	13.507	35.2	3	7	16.8	2	3
Q17QQ1	LAMTOR3	Ragulator complex protein LAMTOR3	13.65	39.5	5	8	8.1	1	1
F1MVX2	LANCL1	Uncharacterized protein	45.308	41.4	13	48	24.1	8	16

				Biological Replicate 1			Biological Replicate 2		
Accession number	Gene symbol	Discription	MW(kDa)	Sequence coverage(%)	Unique peptides	MS/MS count	Sequence coverage(%)	Unique peptides	MS/MS count
A6QPG6	LANCL2	LANCL2 protein	49.166	25.9	9	16	6.5	3	5
F1MZU1	LARP1	Uncharacterized protein (Fragment)	108.78	10.7	8	11	6.1	7	12
F1MHX7	LARP4B	Uncharacterized protein	80.133	4.8	3	5	7	5	6
A6QLR2	LARS	LARS protein	134.81	25.2	26	45	11.4	14	27
Q3B7M5	LASP1	LIM and SH3 domain protein 1	29.677	35.8	9	14	19.2	6	15
A6QQ18	LASS5	LASS5 protein	44.717	5.2	3	5	#N/A	#N/A	#N/A
Q3T0H0	LCMT1	Leucine carboxyl methyltransferase	38.009	19	8	17	8.4	3	7
G3N0F1	LCMT2	Uncharacterized protein	75.549	8.8	5	5	#N/A	#N/A	#N/A
F1MYX5	LCP1	Uncharacterized protein	70.111	6.4	2	3	#N/A	#N/A	#N/A
P19858	LDHA	L-lactate dehydrogenase A chain	36.597	77.1	23	186	37.7	13	84
Q5E9B1	LDHB	L-lactate dehydrogenase B chain	36.723	56.3	15	105	31.1	10	62
A4FUW5	LEO1	LEO1 protein	66.095	4.3	2	4	#N/A	#N/A	#N/A
Q0VCA3	LETM1	LETM1 and EF-hand domain-containing protein 1, mitochondrial	81.817	23.1	15	18	12.7	10	18
F1N3F1	LETMD1	LETM1 domain-containing protein 1	41.928	3.9	1	2	2.5	1	1
P11116	LGALS1	Galectin-1	14.744	51.1	7	23	58.5	8	25
A6QLZ0	LGALS3	Galectin	27.646	29.4	7	15	17.4	5	18
Q2YDD7	LGALS8	Galectin	40.127	6.4	2	4	8.1	3	10
Q95M12	LGMN	Legumain	49.283	10.2	4	4	5.1	2	2
Q5E9G8	LGP2	Putative uncharacterized protein	77.169	9.3	4	4	5.1	3	6
Q5BIP7	LIAS	Lipoyl synthase, mitochondrial	42.035	20.4	6	7	6.7	2	2
A4IFC8	LIG1	DNA ligase	101.31	45.4	39	115	21	20	57
F1MQY1	LIG3	DNA ligase	105.12	#N/A	#N/A	#N/A	1.2	1	2
E1BHM3	LIN28A	Uncharacterized protein	22.388	6.8	1	1	#N/A	#N/A	#N/A
Q0P5F3	LIN7C	Protein lin-7 homolog C	21.834	24.4	5	6	5.1	1	1
F1N110	LIPA	Lipase	45.67	7.8	3	11	2.8	1	7
O46419	LIPT1	Lipoyltransferase 1, mitochondrial	42.072	#N/A	#N/A	#N/A	3.2	1	1
E1BI46	LIPT2	Putative lipoyltransferase 2, mitochondrial (Fragment)	24.421	36.3	7	14	13.3	4	13
Q08E42	LLRC8A	Leucine rich repeat containing 8 family, member A	94.009	4	3	7	1.1	1	1
F1ME46	LMAN1	Uncharacterized protein	58.188	11.2	7	10	8.3	5	9

				Biological Replicate 1			Biological Replicate 2		
Accession number	Gene symbol	Discription	MW(kDa)	Sequence coverage(%)	Unique peptides	MS/MS count	Sequence coverage(%)	Unique peptides	MS/MS count
A6QP36	LMAN2	LMAN2 protein	40.401	40.7	13	31	23.1	9	33
Q2HJD1	LMAN2L	VIP36-like protein	39.991	3.7	2	2	#N/A	#N/A	#N/A
Q3SYY9	LMBRD1	Probable lysosomal cobalamin	62.022	7.4	4	14	#N/A	#N/A	#N/A
A1L504	LMF2	Lipase maturation factor 2	79.265	11	6	12	6.7	4	5
F1MYG5	LMNA	Uncharacterized protein	74.126	21.7	14	21	23.2	15	25
E1B8N6	LMNB1	Uncharacterized protein	66.125	1.7	1	1	#N/A	#N/A	#N/A
A8E4L8	LMO7	LMO7 protein	157.57	17.2	22	37	14.6	21	34
F1N1Q8	LNPEP	Uncharacterized protein (Fragment)	116.85	9.7	9	12	1.8	2	2
E1BIB8	LOC100139891	Uncharacterized protein (Fragment)	15.309	18.9	3	4	13.3	2	2
F1MNH8	LOC100140907	Uncharacterized protein	77.328	3.4	2	3	#N/A	#N/A	#N/A
F1MNF8	LOC100141266	Uncharacterized protein	49.902	70.8	2	192	38.3	1	14
F1MUX5	LOC100295687	Uncharacterized protein (Fragment)	26.724	45.4	5	35	21	1	3
G3MZ99	LOC100299732	Uncharacterized protein	35.863	3.7	1	1	#N/A	#N/A	#N/A
G3MZH8	LOC100301381	Uncharacterized protein (Fragment)	10.257	20.9	2	2	30.8	3	6
F1N453	LOC100336115	Histone H2B	13.919	#N/A	#N/A	#N/A	34.1	1	1
G3MW12	LOC100337053	Uncharacterized protein (Fragment)	129.72	4.3	0	5	#N/A	#N/A	#N/A
G3N081	LOC100337473	Histone H4	10.451	#N/A	#N/A	#N/A	31.9	3	7
G3X795	LOC100847883	Uncharacterized protein (Fragment)	63.525	7	4	4	#N/A	#N/A	#N/A
F1MYH7	LOC100848970	Uncharacterized protein (Fragment)	69.614	1.5	1	1	#N/A	#N/A	#N/A
Q0G846	LOC100851274	LOC615521	21.971	9.8	2	3	#N/A	#N/A	#N/A
F1MIC3	LOC101902556	Uncharacterized protein (Fragment)	16.41	19.3	1	1	#N/A	#N/A	#N/A
F1N3X9	LOC101904184	Uncharacterized protein (Fragment)	52.185	#N/A	#N/A	#N/A	2.2	1	1
F1N5F3	LOC101904455	Histone H2B (Fragment)	14.002	28	2	3	21.6	2	3
F1N5S6	LOC101906511	Uncharacterized protein (Fragment)	47.106	39	13	20	7.6	3	6
G3MZD8	LOC101907314	Uncharacterized protein (Fragment)	47.216	2.1	1	6	#N/A	#N/A	#N/A
G3MYB8	LOC101907815	Uncharacterized protein (Fragment)	19.495	19.5	2	7	#N/A	#N/A	#N/A
G8JKV3	LOC101909651	Uncharacterized protein	11.722	45.3	1	8	#N/A	#N/A	#N/A
G3X6W2	LOC101909711	Uncharacterized protein	153.7	#N/A	#N/A	#N/A	2.5	4	4
F1N0T3	LOC404051	Uncharacterized protein	42.592	20.1	3	4	6.9	1	2
Q2KHV9	LOC506454	Uncharacterized protein KIAA2013	69.586	12.3	7	10	7.7	5	13
E1BN36	LOC507383	Olfactory receptor	34.544	2.9	1	1	2.9	1	1
G3MYS0	LOC508073	Uncharacterized protein	37.655	56.8	16	44	26.8	8	17

				Biological Replicate 1			Biological Replicate 2		
Accession number	Gene symbol	Discription	MW(kDa)	Sequence coverage(%)	Unique peptides	MS/MS count	Sequence coverage(%)	Unique peptides	MS/MS count
A6QLU9	LOC508963	LOC508963 protein	40.028	4	2	3	7.1	3	4
F1N429	LOC512176	Uncharacterized protein (Fragment)	46.861	9.9	4	7	#N/A	#N/A	#N/A
F1MGC3	LOC512672	Uncharacterized protein	41.386	#N/A	#N/A	#N/A	3.7	1	3
A6QLG1	LOC515358	LOC515358 protein	20.59	14.4	2	2	#N/A	#N/A	#N/A
A6QLT3	LOC520387	LOC520387 protein	115.76	1.4	2	2	1	1	1
G3X6B1	LOC525842	Uncharacterized protein	78.981	#N/A	#N/A	#N/A	1.3	1	3
E1B805	LOC528040	Uncharacterized protein (Fragment)	187.27	0.6	1	1	1	2	2
F1MPH8	LOC528262	Uncharacterized protein	47.946	2	1	1	2	1	2
E1B952	LOC528343	Olfactory receptor	34.921	1.9	1	1	#N/A	#N/A	#N/A
E1BEY9	LOC529294	Uncharacterized protein (Fragment)	41.777	42.9	16	59	34.8	14	55
E1BFQ0	LOC532875	Uncharacterized protein	87.867	5.2	2	4	4.2	1	10
Q2NKZ8	LOC538702	Glycerol kinase	60.601	6.1	4	4	4.5	3	5
A7MBF7	LOC616332	LOC616332 protein	22.906	18.6	3	15	3.8	1	1
E1BAC6	LOC617785	Uncharacterized protein (Fragment)	36.817	3	1	2	#N/A	#N/A	#N/A
E1BLG3	LOC781298	Uncharacterized protein	24.347	7	1	1	#N/A	#N/A	#N/A
A6QLQ4	LOC782821	LOC782821 protein	19.667	4	1	1	#N/A	#N/A	#N/A
F1MR68	LOC785899	Olfactory receptor	35.069	#N/A	#N/A	#N/A	2.2	1	1
F6QK33	LOC787087	Uncharacterized protein	88.495	3.6	3	6	4.3	4	4
G3X7D5	LOC787803	Uncharacterized protein (Fragment)	15.575	48.9	6	13	7.8	1	6
P84227	7; LOC504599;	Histone H3.2	15.388	30.9	1	1	17.6	2	2
A6H7H3	LOC789567	Estradiol 17-beta-dehydrogenase 12	34.928	41	9	23	15.4	4	19
Q59HJ6	LONP1	Lon protease homolog, mitochondrial	106.67	26.1	23	50	8.9	10	30
Q3SX23	LONP2	Lon protease homolog 2, peroxisomal	94.309	1.4	1	1	#N/A	#N/A	#N/A
F1MTK4	LPCAT1	Uncharacterized protein (Fragment)	54.33	5.9	3	4	#N/A	#N/A	#N/A
E1B770	LPCAT2	Uncharacterized protein	60.332	#N/A	#N/A	#N/A	5.3	3	4
Q3SZL3	LPCAT3	Lysophospholipid acyltransferase 5	55.833	9.3	4	5	6	3	4
E1BCN1	LPIN2	Uncharacterized protein	98.544	22.7	15	25	12.6	12	35
F1MV58	LRCH4	Uncharacterized protein	70.945	2.4	1	3	1.2	1	2
E1BN69	LRIG3	Uncharacterized protein	123.11	1.3	1	1	#N/A	#N/A	#N/A
F6RBT3	LRPAP1	Uncharacterized protein	41.893	26.2	13	24	23.5	10	20
E1BPX9	LRPPRC	Uncharacterized protein	158.23	26.8	35	52	16.9	25	44
A5PJJ5	LRRC14	Leucine-rich repeat-containing	54.917	7.5	3	4	#N/A	#N/A	#N/A

				Biological Replicate 1			Biological Replicate 2		
Accession number	Gene symbol	Discription	MW(kDa)	Sequence coverage(%)	Unique peptides	MS/MS count	Sequence coverage(%)	Unique peptides	MS/MS count
F1MMJ6	LRRC16A	Uncharacterized protein	147.36	3	4	4	#N/A	#N/A	#N/A
F1MHE7	LRRC40	Uncharacterized protein	68.431	17.1	10	10	5.6	4	7
A6QR01	LRRC47	LRRC47 protein	63.635	6.7	3	3	#N/A	#N/A	#N/A
Q5E9X4	LRRC59	Leucine-rich repeat-containing	34.879	36.3	9	15	6.2	2	4
Q2T9W6	LRRFIP2	Leucine-rich repeat flightless-interacting protein 2	45.493	#N/A	#N/A	#N/A	5.2	2	2
Q08E63	LRSAM1	Leucine rich repeat and sterile alpha motif containing 1	83.666	3.9	2	2	#N/A	#N/A	#N/A
Q2YDM7	LSG1	Large subunit GTPase 1 homolog	74.248	#N/A	#N/A	#N/A	3.1	2	3
Q5E9Z8	LSM1	U6 snRNA-associated Sm-like protein	15.193	46.6	5	9	#N/A	#N/A	#N/A
Q0VCF9	LSM12	Protein LSM12 homolog	21.701	#N/A	#N/A	#N/A	6.7	1	2
Q3MHF8	LSM14A	Protein LSM14 homolog A	50.635	10.2	5	8	4.8	3	3
A6QQV3	LSM2	LSM2 protein	10.834	46.3	4	7	20	1	1
Q32PE9	LSM3	U6 snRNA-associated Sm-like protein	11.845	25.5	3	5	17.6	2	10
Q3ZBK6	LSM4	U6 snRNA-associated Sm-like protein	15.35	31.7	5	14	13.7	2	2
Q148G9	LSM6	LSM6 homolog, U6 small nuclear RNA associated (S. cerevisiae)	9.1275	46.2	4	6	23.8	2	6
F1N6T3	LSM7	Uncharacterized protein	11.602	39.8	3	6	14.6	2	2
Q3ZCE0	LSM8	N-alpha-acetyltransferase 38, NatC auxiliary subunit	10.403	36.5	3	5	27.1	2	2
P84466	LSS	Lanosterol synthase	83.183	23.2	14	25	14.8	11	28
Q0VC06	LTV1	Protein LTV1 homolog	54.787	1.7	1	1	#N/A	#N/A	#N/A
F1MYQ8	LUC7L2	Uncharacterized protein	46.522	#N/A	#N/A	#N/A	3.8	1	1
Q3MHR0	LYPLA1	Acyl-protein thioesterase 1	24.595	25.2	4	5	13.5	3	3
F1N7F4	LYPLA2	Uncharacterized protein	6.205	44.1	1	1	#N/A	#N/A	#N/A
A5PK90	LYPLAL1	LYPLAL1 protein	25.621	10.8	2	4	#N/A	#N/A	#N/A
E1BFE1	LYRM1	Uncharacterized protein	14.315	20.5	3	5	21.3	3	4
Q0VCG0	LYRM4	LYR motif-containing protein 4	10.711	15.4	1	4	42.9	4	8
Q0VCR0	LYRM5	LYR motif-containing protein 5	10.704	10.2	1	2	#N/A	#N/A	#N/A
P11456	M6PR	Cation-dependent mannose-6-phosphate receptor	31.201	11.5	2	3	11.1	3	7
Q3SX32	M6PRBP1	Perilipin	47.602	39	13	31	21.2	9	21

				Biological Replicate 1			Biological Replicate 2		
Accession number	Gene symbol	Discription	MW(kDa)	Sequence coverage(%)	Unique peptides	MS/MS count	Sequence coverage(%)	Unique peptides	MS/MS count
E1BL65	MAD2L1	Uncharacterized protein	23.494	#N/A	#N/A	#N/A	6.8	1	5
Q3ZBV3	MAGOH	Protein mago nashi homolog	17.163	10.3	1	2	#N/A	#N/A	#N/A
G3MY77	MAGT1	Uncharacterized protein (Fragment)	41.343	11.4	4	7	11.1	4	7
A2VE13	MAL2	Protein MAL2	19.146	6.2	1	3	6.2	1	3
F1N7T2	MAN2A1	Uncharacterized protein	130.59	3.1	3	4	2.9	3	5
Q29451	MAN2B1	Lysosomal alpha-mannosidase	112.92	11.5	11	21	5.2	6	12
F1MWT0	MAN2C1	Uncharacterized protein	115.86	22.1	17	26	5.4	5	8
P80513	MANF	Mesencephalic astrocyte-derived neurotrophic factor	20.356	13.4	2	4	5.6	1	2
F1N1S2	MAP1B	Uncharacterized protein	270.27	0.8	2	3	0.8	2	6
O41515	MAP1LC3B	Microtubule-associated proteins 1A/1B light chain 3B	14.704	16.8	2	2	#N/A	#N/A	#N/A
F1N1H2	MAP1S	Microtubule-associated protein 1S	112.48	13.4	12	28	8.5	9	23
Q0VD16	MAP2K1	MAP2K1 protein	43.439	46.1	10	47	16	4	26
Q17QH2	MAP2K2	MAP2K2 protein	44.502	36.2	7	8	16	4	6
A5PJP8	MAP2K4	MAP2K4 protein	45.61	39.3	13	30	21.6	8	19
A6QQK2	MAP3K7IP1	MAP3K7IP1 protein	42.329	5.4	2	2	#N/A	#N/A	#N/A
E1B902	MAP4K2	Mitogen-activated protein kinase kinase kinase kinase	91.517	11.3	7	7	#N/A	#N/A	#N/A
P46196	MAPK1	Mitogen-activated protein kinase 1	41.375	58.9	17	69	33.1	8	27
A4FV00	MAPK10	MAPK10 protein	48.128	14.1	3	3	#N/A	#N/A	#N/A
A6QLR9	MAPK14	MAPK14 protein	41.493	13.6	4	4	#N/A	#N/A	#N/A
E1B8P9	MAPK3	Uncharacterized protein	41.496	39.5	10	18	20.4	4	6
F1MM73	MAPK8	Uncharacterized protein	44.022	#N/A	#N/A	#N/A	4.2	1	4
Q2KI72	MAPK9	Mitogen-activated protein kinase 9	38.225	19.8	3	11	5.3	1	2
Q3SYZ2	MAPKAPK3	MAP kinase-activated protein kinase	43.316	3.1	1	1	#N/A	#N/A	#N/A
F1MH23	MAPKAPK5	Uncharacterized protein (Fragment)	52.876	1.7	1	3	#N/A	#N/A	#N/A
Q3ZBD9	MAPRE1	Microtubule-associated protein RP/EB family member 1	30.113	25	6	15	25.7	6	16
Q3SZP2	MAPRE2	Microtubule-associated protein RP/EB family member 2	36.988	28.8	9	25	15.3	4	10

				Biological Replicate 1			Biological Replicate 2		
Accession number	Gene symbol	Discription	MW(kDa)	Sequence coverage(%)	Unique peptides	MS/MS count	Sequence coverage(%)	Unique peptides	MS/MS count
Q0VC55	MAPRE3	Microtubule-associated protein, RP/EB family, member 3	30.394	11.3	1	2	#N/A	#N/A	#N/A
G5E5D4	MARK1	Uncharacterized protein	87.933	5.6	0	1	6	3	6
A6QNL2	MARK2	MARK2 protein	77.576	#N/A	#N/A	#N/A	6.5	3	3
F1MAZ8	MARK3	Uncharacterized protein	88.943	11.8	4	13	#N/A	#N/A	#N/A
Q2T9L8	MARS	Methionine--tRNA ligase,	100.63	18.7	13	24	8.8	8	15
A6H7E1	MARS2	Methionine--tRNA ligase,	66.647	10.3	6	7	4.9	3	4
G5E5K2	MASTL	Serine/threonine-protein kinase	97.715	2.7	1	1	#N/A	#N/A	#N/A
A7E3T7	MAT2A	S-adenosylmethionine synthase	43.69	37.5	11	21	20.3	8	14
Q29RI9	MAT2B	Methionine adenosyltransferase 2 subunit beta	37.768	26.6	8	9	2.7	1	2
F1MXI4	MATR3	Uncharacterized protein	93.823	3.8	2	2	#N/A	#N/A	#N/A
F1MU42	MAVS	Uncharacterized protein	55.144	#N/A	#N/A	#N/A	2.3	1	1
E1B9A3	MB21D2	Uncharacterized protein	48.635	39	13	31	6.5	4	10
Q0VCY6	MBOAT7	Lysophospholipid acyltransferase 7	52.764	7.4	3	3	2.5	1	2
A5PJY3	MCART1	MCART1 protein	33.956	9.4	2	2	3	1	2
E1BPG0	MCAT	Uncharacterized protein	42.223	30.7	9	15	8.3	3	6
E1BGC1	MCCC1	Uncharacterized protein	80.482	#N/A	#N/A	#N/A	2.2	2	2
G3N1Q0	MCCC2	Uncharacterized protein	33.244	4.2	1	1	#N/A	#N/A	#N/A
F1N506	MCM2	Uncharacterized protein (Fragment)	100.65	26.9	19	40	8.2	7	9
G3X6V0	MCM3	DNA replication licensing factor	90.875	25.6	21	31	12	10	18
E1BLY2	MCM4	Uncharacterized protein	42.381	17.6	5	10	9.9	4	6
Q0V8B7	MCM5	DNA replication licensing factor	82.145	20.2	14	20	9.7	8	14
Q3ZBH9	MCM7	DNA replication licensing factor	81.314	39.9	22	38	12.8	9	14
E1BPX4	MCM8	DNA helicase MCM8	90.767	0.9	1	1	#N/A	#N/A	#N/A
F1MDL6	MCU	Uncharacterized protein (Fragment)	32.399	40.1	8	17	9	2	5
Q3T145	MDH1	Malate dehydrogenase, cytoplasmic	36.438	53.6	20	76	34.7	13	47
Q32LG3	MDH2	Malate dehydrogenase, mitochondrial	35.668	71.6	25	170	61.8	18	157
Q58DB8	MDS018	Phosphopantothenoylcysteine	25.18	43.9	9	22	9.1	3	6
F1N3V0	ME1	Malic enzyme	63.786	52.9	24	144	18.1	10	49
Q08DM3	ME2	Malic enzyme	65.412	62.2	27	89	27.2	15	54
F1MPH4	ME3	Malic enzyme	66.6	19.7	8	17	7.6	4	10

				Biological Replicate 1			Biological Replicate 2		
Accession number	Gene symbol	Discription	MW(kDa)	Sequence coverage(%)	Unique peptides	MS/MS count	Sequence coverage(%)	Unique peptides	MS/MS count
A9Q1J5	MELK	Maternal embryonic leucine zipper	74.251	2.8	2	2	#N/A	#N/A	#N/A
Q3T0U1	MESDC2	LDLR chaperone MESD	25.976	40.5	9	18	6.5	2	6
F1MZU5	MEST	Mesoderm-specific transcript homolog protein	38.793	16.1	4	7	#N/A	#N/A	#N/A
A6QLA4	METAP1	Methionine aminopeptidase 1	43.183	2.1	1	1	#N/A	#N/A	#N/A
Q3ZC89	METAP2	Methionine aminopeptidase 2	52.811	20.8	7	9	5.5	3	6
A5PK19	METTL13	Methyltransferase-like protein 13	78.75	1.3	1	2	#N/A	#N/A	#N/A
F1ML61	METTL22	Uncharacterized protein	39.046	5.1	1	2	#N/A	#N/A	#N/A
E1BLP4	MFF	Mitochondrial fission factor	38.53	27.2	1	7	#N/A	#N/A	#N/A
Q95114	MFGE8	Lactadherin	47.41	51.5	25	206	44	20	141
F1MPL1	MFN1	Uncharacterized protein (Fragment)	85.2	#N/A	#N/A	#N/A	1.2	1	1
E1BKT8	MFN2	Uncharacterized protein	87.628	11.4	8	13	11.2	9	17
Q1JQC1	MFSD1	Major facilitator superfamily domain-containing protein 1	51.537	#N/A	#N/A	#N/A	2.6	1	2
Q0P5M9	MFSD10	Major facilitator superfamily domain-containing protein 10	48.143	8.3	3	4	2	1	1
E1BPY5	MFSD8	Uncharacterized protein	57.792	11.6	4	7	2.3	1	2
E1BAZ5	MFSD9	Uncharacterized protein	50.51	#N/A	#N/A	#N/A	1.4	1	2
Q5E9I4	MGAT1	MGAT1 protein	51.702	8.1	3	7	4	2	2
O77836	MGAT4A	Alpha-1,3-mannosyl-glycoprotein 4-beta-N-	61.617	5.2	3	3	6.4	3	5
F1N6U2	MGAT4B	Uncharacterized protein (Fragment)	51.21	2	1	1	#N/A	#N/A	#N/A
E1BMM2	MGAT5	Uncharacterized protein	84.389	3.2	3	3	#N/A	#N/A	#N/A
Q3T0T9	MGC127133	20-beta-hydroxysteroid	31.707	17.5	1	5	#N/A	#N/A	#N/A
Q32P60	MGC133632	MGC133632 protein	8.4977	17.1	1	2	#N/A	#N/A	#N/A
Q2KIJ4	MGC137058	Hypothetical LOC540154	42.2	#N/A	#N/A	#N/A	2.8	1	1
Q05B90	MGC139698	Hypothetical LOC512024	18.325	5.2	1	2	5.2	1	3
Q08DN5	MGC142781	Carnitine acetyltransferase	71.016	37.1	22	44	21.7	13	33
A6QLS5	MGC143209	MGC143209 protein	41.837	29.1	10	21	5.4	2	6
F1MW73	MGC148692	Uncharacterized protein	138.44	6.4	7	9	1.3	1	2
A6QNZ5	MGC151537	MGC151537 protein	86.26	1.4	1	1	#N/A	#N/A	#N/A
A6QPV5	MGC157319	MGC157319 protein	62.761	1.4	1	1	#N/A	#N/A	#N/A

				Biological Replicate 1			Biological Replicate 2		
Accession number	Gene symbol	Discription	MW(kDa)	Sequence coverage(%)	Unique peptides	MS/MS count	Sequence coverage(%)	Unique peptides	MS/MS count
A5PK88	MGC159500	MGC159500 protein	24.116	71.1	13	33	30.8	8	19
A5PJL7	MGC160092	MGC160092 protein	45.999	5.3	2	2	#N/A	#N/A	#N/A
A5PK67	MGC165862	MGC165862 protein	9.6072	9.6	1	1	#N/A	#N/A	#N/A
Q58D67	MGC3248	Dynactin 4	17.797	#N/A	#N/A	#N/A	11	2	3
E1BQ16	MGEA5	Uncharacterized protein	102.88	9.1	9	14	2	2	2
F6Q192	MGME1	Mitochondrial genome maintenance exonuclease 1	39.224	25.1	7	11	11.1	4	7
I6L9J6	MGRN1	MGRN1 protein	43.4	#N/A	#N/A	#N/A	2.6	1	2
Q2KJG4	MGST2	Microsomal glutathione S-	16.483	9.6	1	4	9.6	1	1
Q3T100	MGST3	Microsomal glutathione S-	16.884	13.8	2	7	13.8	2	6
G5E5L5	MIA3	Melanoma inhibitory activity protein 3 (Fragment)	206.76	1.2	2	2	#N/A	#N/A	#N/A
F1MH07	MICAL1	Protein-methionine sulfoxide oxidase MICAL1	118.69	34.3	32	77	13	15	43
F1MF74	MICAL2	Protein-methionine sulfoxide oxidase MICAL2	124.01	8.1	8	11	2.7	3	6
E1BIT2	MIEF1	Uncharacterized protein	51.117	4.1	2	2	6.5	3	5
P80177	MIF	Macrophage migration inhibitory	12.343	23.5	3	22	15.7	2	2
Q5EA24	MINA	Bifunctional lysine-specific demethylase and histidyl-	52.454	13.4	6	8	#N/A	#N/A	#N/A
F6Q3P6	MINK1	Uncharacterized protein	147.35	1.1	2	2	#N/A	#N/A	#N/A
Q2TBT6	MINPP1	Multiple inositol polyphosphate histidine phosphatase, 1	41.434	4.9	2	2	2.7	1	1
E1B899	MIOS	Uncharacterized protein	98.514	8.5	7	10	9.5	8	12
F1MX73	MIPEP	Uncharacterized protein (Fragment)	81.094	2.2	1	1	5.5	4	4
F1N1A8	MKNK1	MAP kinase-interacting serine/threonine-protein kinase 1	36.421	3.7	1	1	#N/A	#N/A	#N/A
F6QQR5	MKRN2	Uncharacterized protein (Fragment)	34.215	#N/A	#N/A	#N/A	3.2	1	1
A6H797	MLEC	MLEC protein	31.566	44.5	11	34	40.6	11	69
F1N2X7	MLLT4	Uncharacterized protein (Fragment)	197.98	1	1	1	#N/A	#N/A	#N/A
Q17QU5	MLST8	Target of rapamycin complex subunit	35.92	5.5	1	1	#N/A	#N/A	#N/A
A5PJC5	MLYCD	MLYCD protein	55.415	12.4	4	7	7.8	3	5

				Biological Replicate 1			Biological Replicate 2		
Accession number	Gene symbol	Discription	MW(kDa)	Sequence coverage(%)	Unique peptides	MS/MS count	Sequence coverage(%)	Unique peptides	MS/MS count
A6QQK5	MMAA	MMAA protein	46.687	2.4	1	1	6.9	3	4
Q58D49	MMAB	Cob(I)yrinic acid a,c-diamide adenosyltransferase, mitochondrial	26.587	21.2	4	6	14.5	3	4
E1BP36	MMS19	MMS19 nucleotide excision repair protein homolog	113.11	16	15	27	7.7	8	13
Q58D63	MOB3A	MOB kinase activator 3A	25.41	4.1	1	1	#N/A	#N/A	#N/A
Q0VCJ5	MOBKL1B	MOB1, Mps One Binder kinase activator-like 1B (Yeast)	25.079	10.6	2	2	#N/A	#N/A	#N/A
A4FUE6	MOBKL3	MOBKL3 protein	26.032	#N/A	#N/A	#N/A	13.8	3	6
Q1JQD7	MOCS1	Molybdenum cofactor biosynthesis	69.658	6.8	4	8	4.4	3	5
Q70VZ7	MOGAT1	2-acylglycerol O-acyltransferase 1	39.109	26	7	13	3.3	1	2
E1B7U1	MOGS	Uncharacterized protein (Fragment)	79.861	11.1	7	11	11.2	8	15
E1BPI7	MON2	Uncharacterized protein	190.07	13	21	38	4.7	9	15
F1MFY1	MORC3	Uncharacterized protein (Fragment)	109.19	2.6	2	2	#N/A	#N/A	#N/A
A6QQK3	MOSPD2	MOSPD2 protein	56.376	16.7	10	15	#N/A	#N/A	#N/A
Q3ZCG2	MPC1	Mitochondrial pyruvate carrier 1	12.388	#N/A	#N/A	#N/A	6.4	1	1
Q148D6	MPDU1	Mannose-P-dolichol utilization	26.584	5.7	1	3	#N/A	#N/A	#N/A
F1MZT3	MPHOSPH9	Uncharacterized protein	115.93	10.9	8	10	2.2	3	5
Q3SZI0	MPI	Mannose-6-phosphate isomerase	46.368	8.3	3	3	#N/A	#N/A	#N/A
F1MYW5	MPND	Uncharacterized protein (Fragment)	53.725	19.8	7	10	7.5	4	7
E1BIQ8	MPP5	Uncharacterized protein	77.199	1.3	1	1	#N/A	#N/A	#N/A
F1MU05	MPP6	Uncharacterized protein (Fragment)	62.312	16	8	10	#N/A	#N/A	#N/A
Q3MHG3	MPST	Mercaptopyruvate sulfurtransferase	33.192	26.6	7	13	21.9	6	13
E1BIN9	MRE11A	Uncharacterized protein	80.534	1.8	1	1	#N/A	#N/A	#N/A
Q2NL31	MRI1	Methylthioribose-1-phosphate	37.826	4.5	1	1	#N/A	#N/A	#N/A
A7MBD2	MRM1	MRM1 protein	38.923	#N/A	#N/A	#N/A	3.1	1	2
A6QPQ5	MRPL1	39S ribosomal protein L1,	36.656	#N/A	#N/A	#N/A	5.2	2	2
F1MBS4	MRPL12	39S ribosomal protein L12, mitochondrial (Fragment)	32.175	50.8	14	36	34.7	10	51
Q3ZBR7	MRPL18	39S ribosomal protein L18,	20.396	5	1	1	5	1	1
Q2TBR2	MRPL20	39S ribosomal protein L20,	17.534	10.7	2	2	12.1	2	5
Q2TBS2	MRPL21	39S ribosomal protein L21,	23.183	4.8	1	1	8.1	2	5

				Biological Replicate 1			Biological Replicate 2		
Accession number	Gene symbol	Discription	MW(kDa)	Sequence coverage(%)	Unique peptides	MS/MS count	Sequence coverage(%)	Unique peptides	MS/MS count
Q3SZX5	MRPL22	39S ribosomal protein L22,	23.551	7.4	2	2	9.3	2	2
Q3ZBF3	MRPL38	39S ribosomal protein L38,	44.622	#N/A	#N/A	#N/A	5.5	2	5
Q05B74	MRPL39	Mitochondrial ribosomal protein L39	38.459	9.3	3	3	#N/A	#N/A	#N/A
P82927	MRPL42	39S ribosomal protein L42,	16.637	7	1	1	#N/A	#N/A	#N/A
Q2KIS2	MRPL44	39S ribosomal protein L44,	37.412	3.9	1	1	#N/A	#N/A	#N/A
P82670	MRPS10	28S ribosomal protein S10,	23.031	12.9	2	2	#N/A	#N/A	#N/A
Q6B860	MRPS14	28S ribosomal protein S14,	15.043	11.7	1	4	#N/A	#N/A	#N/A
P82915	MRPS16	28S ribosomal protein S16,	15.131	15.6	2	2	#N/A	#N/A	#N/A
P82916	MRPS17	28S ribosomal protein S17,	14.474	13.8	2	3	23.8	3	8
P82669	MRPS25	28S ribosomal protein S25,	20.055	27.7	3	4	5.2	1	2
Q3SZ86	MRPS26	28S ribosomal protein S26,	23.774	16.1	3	8	31.2	6	9
Q32PI8	MRPS27	28S ribosomal protein S27,	47.954	4.6	2	4	#N/A	#N/A	#N/A
P82924	MRPS30	28S ribosomal protein S30,	49.355	3.4	1	1	#N/A	#N/A	#N/A
P82908	MRPS36	28S ribosomal protein S36,	11.543	52.4	5	12	39.8	3	6
F1MNM3	MRS2	Uncharacterized protein	50.52	8.4	4	6	#N/A	#N/A	#N/A
Q3MHE4	MSH2	DNA mismatch repair protein Msh2	104.92	12.2	11	14	5.1	6	9
F1MLH1	MSH3	Uncharacterized protein	126.07	0.8	1	1	0.8	1	4
E1B9Q4	MSH6	Uncharacterized protein	152.52	2.2	3	3	#N/A	#N/A	#N/A
Q2HJ49	MSN	Moesin	67.974	67.8	34	91	39	23	58
F1MGJ1	MSRB2	Uncharacterized protein	19.785	3.3	1	1	#N/A	#N/A	#N/A
A5D9D4	MSTO1	Protein misato homolog 1	62.275	3.3	2	3	#N/A	#N/A	#N/A
P37359	MT3	Metallothionein-3	6.937	#N/A	#N/A	#N/A	33.8	3	5
Q3MHF7	MTAP	S-methyl-5-thioadenosine	31.256	57.6	12	22	19.8	5	14
A7YY65	MTCH1	MTCH1 protein	41.517	37.3	13	38	23.9	10	30
Q9N285	MTCH2	Mitochondrial carrier homolog 2	33.294	17.5	5	12	7.3	3	7
P00396	MTCO1	Cytochrome c oxidase subunit 1	57.032	1.6	1	4	#N/A	#N/A	#N/A
P68530	MTCO2	Cytochrome c oxidase subunit 2	26.021	26.4	5	22	6.2	2	4
P00157	MTCYB	Cytochrome b	42.59	4.2	3	4	#N/A	#N/A	#N/A
Q24JZ4	MTDH	Metadherin	64.054	2.7	1	1	3.3	1	1
Q3SZL7	MTFR1	Mitochondrial fission regulator 1	36.824	7.4	3	4	#N/A	#N/A	#N/A
Q3ZBW7	MTFR1L	Mitochondrial fission regulator 1-	31.712	12.5	3	7	6.6	2	3
A4FUD0	MTHFD1	MTHFD1 protein	101.18	67.2	57	260	41.1	41	197

				Biological Replicate 1			Biological Replicate 2		
Accession number	Gene symbol	Discription	MW(kDa)	Sequence coverage(%)	Unique peptides	MS/MS count	Sequence coverage(%)	Unique peptides	MS/MS count
E1B884	MTHFD1L	Monofunctional C1-tetrahydrofolate synthase, mitochondrial	102.75	#N/A	#N/A	#N/A	2.5	1	1
Q0P5C2	MTHFD2	Bifunctional methylenetetrahydrofolate dehydrogenase/cyclohydrolase,	37.766	#N/A	#N/A	#N/A	7.7	2	3
E1BNJ2	MTHFD2L	Uncharacterized protein	37.452	4	2	2	#N/A	#N/A	#N/A
Q5I598	MTHFR	Methylenetetrahydrofolate reductase	74.485	1.7	1	2	#N/A	#N/A	#N/A
A6QLT4	MTM1	Myotubularin	70.019	7.8	3	3	#N/A	#N/A	#N/A
E1BFE6	MTMR1	Uncharacterized protein	76.565	5.6	1	1	#N/A	#N/A	#N/A
E1BNH5	MTMR12	Uncharacterized protein	85.842	6.3	4	6	2.5	2	2
A6QLE9	MTMR14	MTMR14 protein	66.606	12.4	6	7	#N/A	#N/A	#N/A
F1MMI7	MTMR2	Myotubularin-related protein 2	70.715	17.2	7	10	3.6	2	3
F1MFT9	MTMR3	Uncharacterized protein	122.96	17.1	16	25	8.9	10	21
E1BJH8	MTMR6	Uncharacterized protein	70.116	#N/A	#N/A	#N/A	2	1	2
E1BEV2	MTMR7	Uncharacterized protein	75.906	4.5	3	4	3	2	3
A7MB43	MTMR9	MTMR9 protein	63.485	5.5	3	3	2.7	2	2
Q1JQB6	MTO1	Mitochondrial translation optimization 1 homolog (S.	77.272	2.7	2	2	5.5	4	5
F1MSM5	MTPAP	Uncharacterized protein	65.345	9.1	5	6	4.8	2	4
Q3T0F7	MTPN	Myotrophin	12.895	31.4	3	9	33.1	3	4
Q4JIJ3	MTR	Methionine synthase	140.48	10.6	10	13	3.6	5	10
E1BP52	MTSS1	Uncharacterized protein	82.343	#N/A	#N/A	#N/A	1.5	1	1
F1MGT3	MTSS1L	Uncharacterized protein (Fragment)	75.722	3.1	1	1	#N/A	#N/A	#N/A
Q2TBS1	MTX1	Metaxin-1	35.674	7.3	3	3	6.9	3	3
A6QLL2	MTX2	MTX2 protein	29.679	22.3	6	10	18.6	5	10
F1MRE1	MTX3	Uncharacterized protein	35.133	9.3	3	6	11.2	4	8
E1BGD5	MUL1	Uncharacterized protein	39.612	8.9	2	2	2.3	1	1
Q9GK13	MUT	Methylmalonyl-CoA mutase,	83.234	10.3	7	15	8	6	8
Q5E9T8	MVK	Mevalonate kinase	42.07	21	7	10	9.8	3	5
F1MNN6	MVP	Major vault protein	98.953	67.3	69	2241	65.4	65	1674
E1BKX3	MYBBP1A	Uncharacterized protein	152.04	1.3	2	3	#N/A	#N/A	#N/A
Q2TBP7	MYCBP	C-Myc-binding protein	11.997	#N/A	#N/A	#N/A	19.4	2	6

				Biological Replicate 1			Biological Replicate 2		
Accession number	Gene symbol	Discription	MW(kDa)	Sequence coverage(%)	Unique peptides	MS/MS count	Sequence coverage(%)	Unique peptides	MS/MS count
E1BH40	MYCBP2	Uncharacterized protein	510.3	0.3	2	2	0.5	3	4
Q27991	MYH10	Myosin-10	229.1	19.6	27	61	9.6	15	34
F1MYM9	MYH11	Uncharacterized protein	227.66	4.5	1	1	#N/A	#N/A	#N/A
F1MU39	MYH15	Uncharacterized protein (Fragment)	223.87	1.4	3	4	0.5	1	2
F1MQ37	MYH9	Uncharacterized protein	227.1	11.8	11	22	5.7	7	11
A0JNJ5	MYL1	Myosin light chain 1/3, skeletal muscle isoform	20.932	#N/A	#N/A	#N/A	8.3	1	1
A4IF97	MYL12B	Myosin regulatory light chain 12B	19.692	40.4	6	10	38	6	12
Q148H2	MYL6B	Myosin, light chain 6B, alkali, smooth muscle and non-muscle	23.403	#N/A	#N/A	#N/A	11.4	2	3
Q28824	MYLK	Myosin light chain kinase, smooth	128.82	0.7	1	2	#N/A	#N/A	#N/A
A6QLD6	MYO1B	MYO1B protein	131.83	14.4	15	21	10.7	13	23
F1MHT3	MYO5A	Uncharacterized protein (Fragment)	216.07	8.2	9	12	8.3	10	15
F1MMQ6	MYO5B	Uncharacterized protein (Fragment)	213.41	27.1	42	80	11.3	17	51
F1MX93	MYO5C	Uncharacterized protein	205.62	#N/A	#N/A	#N/A	2.1	2	2
E1BPK6	MYO6	Uncharacterized protein	149.61	27.5	33	60	12.1	16	31
G3N2Y8	MYPN	Uncharacterized protein (Fragment)	95.002	3	1	2	#N/A	#N/A	#N/A
A5PJV8	MZT2	Mitotic-spindle organizing protein	15.891	#N/A	#N/A	#N/A	8.9	1	1
A4FV35	N6AMT1	N6AMT1 protein	22.929	4.2	1	1	#N/A	#N/A	#N/A
Q2KI14	NAA10	N-alpha-acetyltransferase 10	26.52	12.3	4	4	#N/A	#N/A	#N/A
F1N4V5	NAA15	Uncharacterized protein	101.15	18.4	15	28	6.8	7	12
E1BDK3	NAA30	Uncharacterized protein	38.661	9.7	3	3	#N/A	#N/A	#N/A
F1MJT0	NAA35	Uncharacterized protein	86.544	5.4	5	6	#N/A	#N/A	#N/A
Q0IIJ0	NAA50	N-alpha-acetyltransferase 50	19.398	43.2	6	9	#N/A	#N/A	#N/A
A5D7P8	NABP1	SOSS complex subunit B2	22.449	4.4	1	1	#N/A	#N/A	#N/A
Q5E9A1	NACA	Nascent polypeptide-associated complex subunit alpha	23.37	18.6	3	7	12.6	2	2
F1MV80	NADK2	Uncharacterized protein	52.42	58.2	25	110	44	18	121
E1B8X4	NAE1	Uncharacterized protein	60.385	5.8	3	5	1.7	1	1
Q58DH9	NAGA	Alpha-N-acetylgalactosaminidase	46.532	10.7	4	8	9.7	4	8
Q3SZM9	NAGK	N-acetyl-D-glucosamine kinase	37.267	26.2	8	18	11	3	5
A6QM01	NAGLU	NAGLU protein	74.075	6.7	3	3	#N/A	#N/A	#N/A

				Biological Replicate 1			Biological Replicate 2		
Accession number	Gene symbol	Discription	MW(kDa)	Sequence coverage(%)	Unique peptides	MS/MS count	Sequence coverage(%)	Unique peptides	MS/MS count
G5E597	NAGPA	N-acetylglucosamine-1-phosphodiester alpha-N-	57.086	6.3	3	3	2.9	1	1
A6H767	NAP1L1	Nucleosome assembly protein 1-like	45.376	31.2	10	40	11.8	3	7
Q2TA40	NAP1L4	Nucleosome assembly protein 1-like	43.985	23.3	6	22	14.8	3	5
Q58CN9	NAPEPLD	N-acyl-phosphatidylethanolamine-hydrolyzing phospholipase D	45.341	8.9	3	6	#N/A	#N/A	#N/A
P81127	NAPG	Gamma-soluble NSF attachment	34.636	17.6	5	8	16.7	5	12
A4FV58	NARFL	Cytosolic Fe-S cluster assembly factor NARFL	52.605	#N/A	#N/A	#N/A	1.7	1	3
Q2KJG3	NARS	Asparagine--tRNA ligase,	64.398	37.9	18	29	3.4	2	2
F6QRZ3	NARS2	Uncharacterized protein	53.794	2.5	1	1	#N/A	#N/A	#N/A
Q2T9P4	NASP	Nuclear autoantigenic sperm protein	83.687	39.9	27	94	21	18	55
Q3MHZ1	NAT14	N-acetyltransferase 14	21.733	14.6	2	3	3.9	1	1
F1MPS0	NCAPD3	Uncharacterized protein	166.48	0.7	1	1	#N/A	#N/A	#N/A
F1MUI7	NCAPG	Uncharacterized protein	114.9	11.3	11	16	7.5	7	15
F1MH70	NCAPH	Condensin complex subunit 2	80.693	2.5	2	2	4.8	4	7
E1BMM0	NCBP1	Uncharacterized protein (Fragment)	91.852	15.6	9	9	#N/A	#N/A	#N/A
O77774	NCF1	Neutrophil cytosol factor 1	45.345	2	1	2	#N/A	#N/A	#N/A
Q1LZB2	NCK1	NCK adaptor protein 1	42.905	18	5	5	6.4	2	3
A6H720	NCK2	NCK2 protein	41.911	29	9	18	11.7	4	9
F1MPZ9	NCKAP1	Uncharacterized protein (Fragment)	124.89	13.7	13	15	9.5	12	16
E1B876	NCKAP1L	Uncharacterized protein	128.87	0.6	1	2	#N/A	#N/A	#N/A
E1BG80	NCKIPSD	Uncharacterized protein	79.585	5.1	4	4	#N/A	#N/A	#N/A
E1B8K6	NCL	Uncharacterized protein	77.852	30.6	22	45	25.1	21	55
Q1LZC3	NCLN	Nicalin homolog (Zebrafish)	63.108	38.8	22	52	26.3	15	40
F1MT12	NCSTN	Uncharacterized protein	79.086	11.6	8	16	4.6	4	12
Q6QTH1	ND1	NADH-ubiquinone oxidoreductase	35.656	11.6	3	4	#N/A	#N/A	#N/A
Q6QTH0	ND2	NADH-ubiquinone oxidoreductase	39.213	2.6	1	2	2	1	2
Q85BD6	ND5	NADH-ubiquinone oxidoreductase	68.256	4	2	4	4	2	2
E1BM06	NDC1	Uncharacterized protein	76.594	25.4	15	37	17.8	10	21
E1BF82	NDC80	Uncharacterized protein	73.767	10.7	7	8	11.2	8	12
F1ML71	NDFIP2	Uncharacterized protein	36.761	#N/A	#N/A	#N/A	4.2	1	1

				Biological Replicate 1			Biological Replicate 2		
Accession number	Gene symbol	Discription	MW(kDa)	Sequence coverage(%)	Unique peptides	MS/MS count	Sequence coverage(%)	Unique peptides	MS/MS count
F1MCV2	NDNL2	Uncharacterized protein	32.094	3.2	1	1	#N/A	#N/A	#N/A
Q1JPJ0	NDOR1	NADPH-dependent diflavin	66.567	5.2	3	3	#N/A	#N/A	#N/A
A7MB28	NDRG3	Protein NDRG3	41.251	10.9	3	6	2.7	1	2
P34942	NDUFA10	NADH dehydrogenase [ubiquinone] 1 alpha subcomplex subunit 10,	39.263	22.2	7	15	9.6	3	7
Q8HXG6	NDUFA11	NADH dehydrogenase [ubiquinone] 1 alpha subcomplex subunit 11	14.758	7.8	1	2	#N/A	#N/A	#N/A
O97725	NDUFA12	NADH dehydrogenase [ubiquinone] 1 alpha subcomplex subunit 12	17.089	40	6	14	42.1	6	18
Q95KV7	NDUFA13	NADH dehydrogenase [ubiquinone] 1 alpha subcomplex subunit 13	16.673	35.4	5	9	6.9	1	5
Q02371	NDUFA3	NADH dehydrogenase [ubiquinone] 1 alpha subcomplex subunit 3	9.3488	21.4	2	2	#N/A	#N/A	#N/A
Q01321	NDUFA4	NADH dehydrogenase [ubiquinone] 1 alpha subcomplex subunit 4	9.3246	34.1	3	5	28	3	5
P23935	NDUFA5	NADH dehydrogenase [ubiquinone] 1 alpha subcomplex subunit 5	13.315	49.1	4	7	22.4	3	7
Q02366	NDUFA6	NADH dehydrogenase [ubiquinone] 1 alpha subcomplex subunit 6	15.053	39.8	4	11	27.3	3	10
Q05752	NDUFA7	NADH dehydrogenase [ubiquinone] 1 alpha subcomplex subunit 7	12.676	60.2	9	11	38.1	5	12
P42029	NDUFA8	NADH dehydrogenase [ubiquinone] 1 alpha subcomplex subunit 8	20.091	25	3	6	29.1	4	9
P34943	NDUFA9	NADH dehydrogenase [ubiquinone] 1 alpha subcomplex subunit 9,	42.848	32.6	12	31	13.2	5	18
P52505	NDUFAB1	Acyl carrier protein, mitochondrial	17.402	#N/A	#N/A	#N/A	11.5	2	4
E1BAZ2	NDUFAF1	Uncharacterized protein	37.948	4.3	2	2	#N/A	#N/A	#N/A
Q2HJI2	NDUFAF3	NADH dehydrogenase [ubiquinone] 1 alpha subcomplex assembly factor 3	20.477	33.2	5	10	20.1	4	10
A4FUH5	NDUFAF4	NADH dehydrogenase [ubiquinone] 1 alpha subcomplex assembly factor 4	20.108	37.7	6	15	17.1	3	5

				Biological Replicate 1			Biological Replicate 2		
Accession number	Gene symbol	Discription	MW(kDa)	Sequence coverage(%)	Unique peptides	MS/MS count	Sequence coverage(%)	Unique peptides	MS/MS count
A7YVD7	NDUFAF6	NADH dehydrogenase (ubiquinone) complex I, assembly factor 6	38.102	12	3	3	#N/A	#N/A	#N/A
Q2KHV5	NDUFAF7	NADH dehydrogenase [ubiquinone] complex I, assembly factor 7	49.264	8.6	4	7	#N/A	#N/A	#N/A
Q02373	NDUFB10	NADH dehydrogenase [ubiquinone] 1 beta subcomplex subunit 10	20.965	33.5	6	16	20.5	3	8
Q8HXG5	NDUFB11	NADH dehydrogenase [ubiquinone] 1 beta subcomplex subunit 11,	17.573	18.2	2	4	7.1	1	4
Q02365	NDUFB3	NADH dehydrogenase [ubiquinone] 1 beta subcomplex subunit 3	11.141	12.2	1	2	12.2	1	5
P48305	NDUFB4	NADH dehydrogenase [ubiquinone] 1 beta subcomplex subunit 4	15.184	13.2	1	1	15.5	2	3
Q02380	NDUFB5	NADH dehydrogenase [ubiquinone] 1 beta subcomplex subunit 5,	21.589	10.1	2	3	16.9	3	3
Q02367	NDUFB6	NADH dehydrogenase [ubiquinone] 1 beta subcomplex subunit 6	15.524	21.1	2	2	14.8	2	6
Q02368	NDUFB7	NADH dehydrogenase [ubiquinone] 1 beta subcomplex subunit 7	16.398	19	2	3	9.5	1	1
Q02372	NDUFB8	NADH dehydrogenase [ubiquinone] 1 beta subcomplex subunit 8,	21.652	29	4	6	#N/A	#N/A	#N/A
Q02369	NDUFB9	NADH dehydrogenase [ubiquinone] 1 beta subcomplex subunit 9	21.789	29.6	6	11	7.8	2	9
Q02827	NDUFC2	NADH dehydrogenase [ubiquinone] 1 subunit C2	14.096	#N/A	#N/A	#N/A	15	2	3
P15690	NDUFS1	NADH-ubiquinone oxidoreductase 75 kDa subunit, mitochondrial	79.441	48.1	30	69	29.8	22	55
P17694	NDUFS2	NADH dehydrogenase [ubiquinone] iron-sulfur protein 2,	52.555	37.6	15	43	14.9	8	30
F1MS25	NDUFS3	NADH dehydrogenase [ubiquinone] iron-sulfur protein 3,	27.902	46.3	10	27	22.7	5	21
Q02375	NDUFS4	NADH dehydrogenase [ubiquinone] iron-sulfur protein 4,	19.811	23.4	4	5	20.6	3	7

				Biological Replicate 1			Biological Replicate 2		
Accession number	Gene symbol	Discription	MW(kDa)	Sequence coverage(%)	Unique peptides	MS/MS count	Sequence coverage(%)	Unique peptides	MS/MS count
P23934	NDUFS6	NADH dehydrogenase [ubiquinone] iron-sulfur protein 6,	13.413	12.1	1	3	21.8	2	7
A8WFQ0	NDUFS7	NADH dehydrogenase [ubiquinone] iron-sulfur protein 7,	20.121	32.4	6	13	24	4	12
P42028	NDUFS8	NADH dehydrogenase [ubiquinone] iron-sulfur protein 8,	23.896	27.8	5	12	17.5	3	6
P25708	NDUFV1	NADH dehydrogenase [ubiquinone] flavoprotein 1, mitochondrial	50.651	58.2	19	57	30.2	14	33
P04394	NDUFV2	NADH dehydrogenase [ubiquinone] flavoprotein 2, mitochondrial	27.307	34.5	6	12	12	3	9
E1BME9	NDUFV3	NADH dehydrogenase [ubiquinone] flavoprotein 3, mitochondrial	51.58	9.1	4	5	18.5	8	12
F1MQI3	NEB	Uncharacterized protein	801.88	#N/A	#N/A	#N/A	0.2	1	1
Q3T093	NECAP1	Adaptin ear-binding coat-associated protein 1	29.597	5.5	2	2	3.3	1	3
Q5E9Q4	NECAP2	Adaptin ear-binding coat-associated protein 2	28.551	39.8	8	19	18	5	12
Q3B7M6	NEDD1	Protein NEDD1	71.203	4.1	2	2	4.1	2	2
F1MJ10	NEDD4	Uncharacterized protein	148.56	2.4	1	2	#N/A	#N/A	#N/A
F1MJ09	NEDD4L	E3 ubiquitin-protein ligase	100.81	9.6	5	9	3.8	2	4
E1BBM3	NEK1	Uncharacterized protein	145.08	#N/A	#N/A	#N/A	0.6	1	2
A4IFF6	NEK3	NEK3 protein	56.245	2.2	1	1	#N/A	#N/A	#N/A
F1MM88	NEK9	Uncharacterized protein (Fragment)	108.34	18.7	13	21	7.9	8	19
G5E5S2	NELFB	Uncharacterized protein (Fragment)	65.622	9.5	5	6	2.2	1	1
F1MW69	NELFCD	Uncharacterized protein (Fragment)	67.484	11	5	7	#N/A	#N/A	#N/A
Q0V898	NELFE	Negative elongation factor E	42.33	4.8	1	1	#N/A	#N/A	#N/A
F1MKA1	NEMF	Uncharacterized protein	123.14	2	2	3	2.5	3	3
F1MQ21	NES	Uncharacterized protein (Fragment)	168.1	#N/A	#N/A	#N/A	0.6	1	2
A5PKG4	NFS1	NFS1 protein	50.343	7.9	4	6	14.9	7	14
Q2KJF3	NFU1	NFU1 iron-sulfur cluster scaffold homolog (S. cerevisiae)	28.145	11.1	2	3	9.9	2	4
F1MPV5	NFXL1	Uncharacterized protein	101.68	5.3	4	4	7.8	6	8

				Biological Replicate 1			Biological Replicate 2		
Accession number	Gene symbol	Discription	MW(kDa)	Sequence coverage(%)	Unique peptides	MS/MS count	Sequence coverage(%)	Unique peptides	MS/MS count
E1BKB2	NGLY1	Uncharacterized protein	74.58	29.1	23	47	19.9	15	29
Q1JQA6	NHEJ1	Nonhomologous end-joining factor 1	33.584	2.7	1	2	#N/A	#N/A	#N/A
Q3B8S0	NHP2L1	NHP2-like protein 1	14.173	9.4	1	1	#N/A	#N/A	#N/A
Q05B89	NIF3L1	Putative GTP cyclohydrolase 1 type 2 NIF3L1	41.905	46.9	14	46	19.1	7	22
E1BQ19	NIN	Uncharacterized protein (Fragment)	207.39	0.8	2	3	#N/A	#N/A	#N/A
Q5E9Q0	NIP30	NEFA-interacting nuclear protein	28.596	5.9	1	2	9.9	3	3
F1MSQ7	NIPSNAP1	Uncharacterized protein	33.125	14.4	4	7	16.5	5	7
G3X6L8	NIPSNAP3A	Uncharacterized protein	28.523	25.1	4	4	5.3	1	2
F1N322	NISCH	Uncharacterized protein (Fragment)	172.13	4.7	7	11	#N/A	#N/A	#N/A
Q32LH4	NIT1	Nitrilase homolog 1	36.239	5.5	1	1	#N/A	#N/A	#N/A
F1MJ59	NIT2	Omega-amidase NIT2	30.433	57.6	13	36	40.9	11	27
G3MYW2	NKRF	Uncharacterized protein	77.719	#N/A	#N/A	#N/A	2.2	1	1
A2VDQ5	NLN	Neurolysin, mitochondrial	80.727	#N/A	#N/A	#N/A	2.4	2	3
E1BNN6	NLRP1	Uncharacterized protein	165.51	4.4	6	6	#N/A	#N/A	#N/A
G3MYV6	NLRP13	Uncharacterized protein	115.51	50.4	48	108	25.5	25	65
E1BDZ4	NLRP14	Uncharacterized protein	125	49.6	48	176	22.8	23	93
G3N3W5	NLRP2	Uncharacterized protein (Fragment)	104.69	57.7	30	120	30.5	21	116
Q647I9	NLRP5	NACHT, LRR and PYD domains-containing protein 5	120.99	70.2	3	296	40.5	2	251
G3N2C1	NLRP7	Uncharacterized protein (Fragment)	101.34	59.8	37	261	30.3	23	177
E1BL33	NLRP8	Uncharacterized protein	115.5	53.7	52	271	27	29	157
F1MKL3	NLRP9	NACHT, LRR and PYD domains-containing protein 9	114.23	49.9	2	193	18.8	1	56
Q08DS5	NMD3	60S ribosomal export protein NMD3	57.748	16.5	8	8	#N/A	#N/A	#N/A
P52174	NME11	Nucleoside diphosphate kinase A 1	17.261	73.7	1	1	#N/A	#N/A	#N/A
P52175	NME12	Nucleoside diphosphate kinase A 2	17.298	84.2	3	132	55.3	5	37
Q3T0Q4	NME2	Nucleoside diphosphate kinase B	17.316	67.8	7	72	55.3	6	110
A5PK70	NME3	Nucleoside diphosphate kinase	19.079	13.6	3	3	7.1	1	2
G1K159	NME7	Nucleoside diphosphate kinase 7	22.368	5.6	1	2	#N/A	#N/A	#N/A
Q0VCN1	NMRAL1	NmrA-like family domain-containing protein 1	33.153	5.7	2	2	7.7	3	3

				Biological Replicate 1			Biological Replicate 2		
Accession number	Gene symbol	Discription	MW(kDa)	Sequence coverage(%)	Unique peptides	MS/MS count	Sequence coverage(%)	Unique peptides	MS/MS count
F1MZK0	NMT1	Glycylpeptide N-	56.805	6.2	3	3	#N/A	#N/A	#N/A
P11024	NNT	NAD(P) transhydrogenase,	113.85	24.7	26	56	17	19	51
F1MVS4	NOL3	Uncharacterized protein	22.596	20.4	4	8	14.6	2	2
F1MF76	NOMO1	Uncharacterized protein	134.07	36	31	63	13.8	16	51
Q2KJ42	NONO	Non-POU domain containing, octamer-	54.121	#N/A	#N/A	#N/A	6.4	1	1
Q3SZ63	NOP56	Nucleolar protein 56	66.33	3.9	2	2	#N/A	#N/A	#N/A
B0JYK2	NPC1	NPC1 protein	141.84	4.9	6	11	2.6	4	9
P79345	NPC2	Epididymal secretory protein E1	16.64	18.1	5	21	8.7	3	12
F1MPJ8	NPEPL1	Uncharacterized protein (Fragment)	50.295	41.1	16	33	15.6	6	20
E1BP91	NPEPPS	Uncharacterized protein	103.6	49.7	40	88	18.9	18	55
F1N7U2	NPLOC4	Uncharacterized protein	68.039	28.1	18	29	12.2	8	16
Q3T160	NPM1	Nucleophosmin	32.703	31.6	8	17	18	6	24
D2DSG3	NPM2	Nucleoplasmin 2	22.582	65.5	10	68	34	6	28
F6RM11	NPTN	Uncharacterized protein	30.975	#N/A	#N/A	#N/A	3.2	1	2
G3MYU9	NPTX2	Uncharacterized protein	46.887	21.7	11	23	15.2	9	24
Q3SZT2	NQO2	NAD(P)H dehydrogenase, quinone 2	25.916	7.4	1	1	#N/A	#N/A	#N/A
A5D7R9	NRAS	NRAS protein	21.229	#N/A	#N/A	#N/A	10.6	2	6
Q08DY5	NRBP1	Nuclear receptor binding protein 1	59.884	7.9	3	6	2.6	1	1
F1MXX5	NRD1	Uncharacterized protein (Fragment)	119.38	0.8	1	2	0.8	1	2
Q3ZBE9	NSDHL	Sterol-4-alpha-carboxylate 3-dehydrogenase, decarboxylating	39.902	42.7	12	36	20.8	8	35
F1MZU2	NSF	Uncharacterized protein	82.991	59	53	185	42.1	33	122
Q3SZC4	NSFL1C	NSFL1 cofactor p47	40.653	46.5	14	23	41.4	12	24
F1MK61	NSUN2	Uncharacterized protein	86.51	4	2	2	#N/A	#N/A	#N/A
O46411	NT5C2	Cytosolic purine 5-nucleotidase	64.84	18.6	8	14	1.8	1	3
F1MLB9	NT5C3L	Uncharacterized protein	33.89	8.9	3	3	#N/A	#N/A	#N/A
Q2TBU5	NT5DC1	5-nucleotidase domain-containing	51.365	6.9	3	6	2	1	1
E1BNI8	NT5DC2	Uncharacterized protein	64.314	19	9	14	10.6	7	9
Q05927	NT5E	5-nucleotidase	62.965	16.9	5	17	12.4	6	12
E1B891	NTAN1	Uncharacterized protein	34.639	29.4	7	9	#N/A	#N/A	#N/A
Q1LZ78	NTPCR	Cancer-related nucleoside-triphosphatase homolog	20.682	25.3	4	6	12.1	2	5

				Biological Replicate 1			Biological Replicate 2		
Accession number	Gene symbol	Discription	MW(kDa)	Sequence coverage(%)	Unique peptides	MS/MS count	Sequence coverage(%)	Unique peptides	MS/MS count
Q05B69	NUB1	NEDD8 ultimate buster 1	70.381	9.9	6	8	#N/A	#N/A	#N/A
Q24K00	NUBP1	Cytosolic Fe-S cluster assembly factor NUBP1	34.182	18.8	5	10	6.9	3	4
Q3MHY6	NUBP2	Cytosolic Fe-S cluster assembly factor NUBP2	28.789	33.6	6	8	5.9	1	1
E1BE32	NUBPL	Uncharacterized protein	35.733	6.3	2	2	6	2	3
Q0P569	NUCB1	Nucleobindin-1	54.982	12	5	6	9.3	5	7
Q0IIH5	NUCB2	Nucleobindin 2	49.188	5.5	2	3	12	5	11
Q17QG2	NUDC	Nuclear migration protein nudC	38.242	43.7	16	36	30.7	10	26
E1BLF7	NUDCD1	Uncharacterized protein	66.757	21.3	11	17	8.4	5	10
Q32KU8	NUDCD2	NudC domain containing 2	17.646	7	1	1	#N/A	#N/A	#N/A
Q24K01	NUDCD3	NudC domain containing 3	40.178	3.1	1	1	#N/A	#N/A	#N/A
F1MLL8	NUDT1	Uncharacterized protein (Fragment)	18.187	9.5	1	1	#N/A	#N/A	#N/A
A7MBH2	NUDT11	Diphosphoinositol polyphosphate phosphohydrolase 3-beta	18.518	11.6	3	3	#N/A	#N/A	#N/A
Q05B60	NUDT14	Uridine diphosphate glucose pyrophosphatase	23.901	27.5	5	7	6.8	2	2
E1B7T3	NUDT15	Uncharacterized protein	19.559	48	6	22	12.9	2	2
A1A4Q9	NUDT16	U8 snoRNA-decapping enzyme	21.398	47.2	7	11	5.6	1	2
A4FUG7	NUDT17	Nucleoside diphosphate-linked moiety X motif 17	32.389	6.3	1	2	#N/A	#N/A	#N/A
Q29RJ1	NUDT2	Bis(5-nucleosyl)-tetraphosphatase [asymmetrical]	16.775	46.3	5	15	#N/A	#N/A	#N/A
Q3ZCA2	NUDT21	Cleavage and polyadenylation specificity factor subunit 5	26.227	33.9	7	11	7.5	2	3
F1MMR8	NUDT5	Uncharacterized protein	18.648	18.6	3	7	9.3	1	2
A7MBI8	NUDT9	NUDT9 protein	38.612	14.6	5	6	#N/A	#N/A	#N/A
A6H7B3	NUF2	NUF2 protein	54.61	13.5	6	6	5.6	3	3
F1MXK2	NUFIP2	Uncharacterized protein (Fragment)	72.859	6.4	4	6	#N/A	#N/A	#N/A
E1BDU0	NUP107	Uncharacterized protein	107.16	10.2	8	10	1.7	2	2
F1MM55	NUP133	Uncharacterized protein	129.06	15.9	14	20	3.9	5	8
F1MNT1	NUP155	Uncharacterized protein	155.18	7.9	10	13	5.9	7	11

				Biological Replicate 1			Biological Replicate 2		
Accession number	Gene symbol	Discription	MW(kDa)	Sequence coverage(%)	Unique peptides	MS/MS count	Sequence coverage(%)	Unique peptides	MS/MS count
F1N6B6	NUP188	Uncharacterized protein	196.27	4.2	7	9	#N/A	#N/A	#N/A
A6QPZ3	NUP35	NUP35 protein	28.796	16.9	4	6	4.9	1	2
A3KMW1	NUP43	NUP43 protein	42.09	8.2	2	3	#N/A	#N/A	#N/A
F1N1Q5	NUP54	Uncharacterized protein	56.03	9	2	6	#N/A	#N/A	#N/A
Q3SZ56	NUP88	Nucleoporin 88kDa	83.771	11.2	6	6	3	2	4
A5PJZ5	NUP93	Nuclear pore complex protein Nup93	93.457	31	24	45	16.1	14	26
E1BCV4	NUP98	Uncharacterized protein	197.63	7.1	10	12	3.7	7	10
A5D7B3	NUPL1	NUPL1 protein	59.302	4.6	3	3	#N/A	#N/A	#N/A
Q2YDJ0	NUSAP1	Nucleolar and spindle-associated	52.327	8.2	4	4	5.8	3	5
Q32KP9	NUTF2	Nuclear transport factor 2	14.478	74.8	6	37	22.8	3	7
A6QLU8	NXN	Nucleoredoxin	48.331	21.8	7	16	15.2	5	10
Q1LZ74	OARD1	O-acetyl-ADP-ribose deacetylase 1	17.034	32.2	5	8	#N/A	#N/A	#N/A
Q3ZCF5	OAT	Ornithine aminotransferase,	48.075	19.4	7	12	#N/A	#N/A	#N/A
Q08DB2	OBFC1	CST complex subunit STN1	41.935	5.1	2	2	#N/A	#N/A	#N/A
Q5E948	Ociad1	OCIA domain-containing protein 1	27.829	17.8	4	6	3.2	1	2
Q148N0	OGDH	2-oxoglutarate dehydrogenase, mitochondrial	115.81	56.2	38	136	23.2	17	74
F1MWG1	OGDHL	Uncharacterized protein	114.42	42.6	25	65	17.9	12	35
F6RA54	OGFR	Uncharacterized protein	59.556	5.4	2	2	#N/A	#N/A	#N/A
A5D7G1	OGT	OGT protein	115.75	3	2	2	#N/A	#N/A	#N/A
Q2HJ33	OLA1	Obg-like ATPase 1	44.743	43.2	13	21	10.6	5	8
Q3SZN3	OMA1	Metalloendopeptidase OMA1,	59.895	2.3	1	1	#N/A	#N/A	#N/A
A0JNQ6	OOEP	Oocyte-expressed protein homolog	15.868	42.1	6	118	35.7	6	34
Q2Q0J1	OOSP1	Oocyte-secreted protein 1	18.506	6.7	1	2	6.7	1	3
E1BBC4	OPA1	Uncharacterized protein	115.59	56.7	63	221	43.4	45	205
D9IE06	OPA3	Mitochondrial optic atrophy 3 protein isoform b	20.655	19.4	4	7	4.8	1	5
Q3ZC32	OPTN	Optineurin	65.238	7	3	4	5.6	3	5
A5PKA7	ORAI1	ORAI1 protein	32.821	4.3	1	1	#N/A	#N/A	#N/A
Q58DC8	ORC1	Origin recognition complex subunit	97.901	1.6	1	1	#N/A	#N/A	#N/A
F1N230	ORC2	Origin recognition complex subunit	66.224	6.2	3	3	2.8	2	4
Q32PJ3	ORC3	Origin recognition complex subunit	82.23	1.1	1	2	2.1	2	4

				Biological Replicate 1			Biological Replicate 2		
Accession number	Gene symbol	Discription	MW(kDa)	Sequence coverage(%)	Unique peptides	MS/MS count	Sequence coverage(%)	Unique peptides	MS/MS count
A5PJS5	ORC5L	ORC5L protein	50.52	8.3	3	5	2.1	1	1
Q29RQ9	ORMDL1	ORM1-like protein 1	17.361	19	2	3	#N/A	#N/A	#N/A
Q5E972	ORMDL2	ORM1-like protein 2	17.403	#N/A	#N/A	#N/A	7.2	1	2
Q3MHX6	OS9	Protein OS-9	75.778	9.7	7	8	6	4	8
E1BPW1	OSBP	Oxysterol-binding protein	78.808	20.1	15	27	6.8	5	7
G3X8E8	OSBPL10	Oxysterol-binding protein	48.538	20.4	7	13	11	4	8
E1BLV1	OSBPL1A	Oxysterol-binding protein	108.44	24.3	21	42	10.4	11	15
E1BL67	OSBPL2	Oxysterol-binding protein	54.983	8.8	3	3	#N/A	#N/A	#N/A
F1MLJ5	OSBPL3	Oxysterol-binding protein	104.27	16.7	13	19	4.7	4	5
E1BA05	OSBPL8	Oxysterol-binding protein	100.09	10.5	8	13	2.5	2	2
F6Q615	OSBPL9	Oxysterol-binding protein	84.372	22.8	14	29	6.3	5	8
Q29S00	OSCP1	Protein OSCP1	43.016	5.3	1	1	#N/A	#N/A	#N/A
Q2KID7	OSTC	Oligosaccharyltransferase complex subunit OSTC	16.769	8.1	1	4	#N/A	#N/A	#N/A
Q3T0Y1	OTUB1	OTU domain, ubiquitin aldehyde	31.308	57.2	13	52	15.9	5	15
G3MZU7	OTUD3	Uncharacterized protein	46.489	5.3	1	3	#N/A	#N/A	#N/A
E1BN58	OTUD4	Uncharacterized protein	122.39	6	7	11	3.8	4	7
E1BFW9	OTUD5	Uncharacterized protein	60.321	3	1	1	#N/A	#N/A	#N/A
F1N0N7	OTUD6B	Uncharacterized protein	33.596	13.7	3	5	#N/A	#N/A	#N/A
E1B9S7	OTUD7B	Uncharacterized protein	91.973	1.7	1	1	#N/A	#N/A	#N/A
Q3SZ07	OVCA2	Ovarian cancer-associated gene 2 protein homolog	24.167	8.4	2	7	#N/A	#N/A	#N/A
F1N5F0	OVCH1	Uncharacterized protein (Fragment)	117.84	8.6	10	19	7.5	9	26
Q24JZ7	OXCT1	Succinyl-CoA:3-ketoacid-coenzyme A transferase	56.452	44.6	18	60	19.8	10	26
Q0VCA7	OXSM	3-oxoacyl-[acyl-carrier-protein] synthase, mitochondrial	48.616	8.3	3	4	#N/A	#N/A	#N/A
F1MYV9	OXSR1	Uncharacterized protein	57.926	27.7	13	26	12.5	7	14
Q3T044	P33MONOX	Putative monooxygenase p33MONOX	32.987	6.2	2	2	#N/A	#N/A	#N/A
F1MJQ4	P4HA2	Uncharacterized protein	60.727	32.8	16	28	22.5	11	25
A0JBZ9	p97bcnt2	Bucentaur-2	70.833	32.2	7	37	15.3	6	18
Q3ZBH5	PA2G4	Proliferation-associated 2G4, 38kDa	43.802	39.8	14	24	14.5	5	8

				Biological Replicate 1			Biological Replicate 2		
Accession number	Gene symbol	Discription	MW(kDa)	Sequence coverage(%)	Unique peptides	MS/MS count	Sequence coverage(%)	Unique peptides	MS/MS count
P61286	PABPC1	Polyadenylate-binding protein 1	70.67	37.6	9	24	28.3	9	41
E1BNB4	PABPC1L	Uncharacterized protein	68.685	47.4	26	150	34.5	18	110
A4IFC3	PABPC4	PABPC4 protein	70.837	42.9	14	55	22.3	6	23
E1BG62	PACS1	Uncharacterized protein (Fragment)	90.301	4.4	3	4	#N/A	#N/A	#N/A
Q1RMR9	PACSIN2	Protein kinase C and casein kinase substrate in neurons 2	55.89	38.9	17	31	23	13	26
E1BCN3	PADI4	Uncharacterized protein	74.158	1.1	1	6	1.1	1	3
F1N404	PADI6	Uncharacterized protein	76.993	64.2	46	862	42	34	435
P68401	PAFAH1B2	Platelet-activating factor acetylhydrolase IB subunit beta	25.569	30.6	6	10	7.9	2	8
Q29460	PAFAH1B3	Platelet-activating factor acetylhydrolase IB subunit gamma	25.865	57.8	11	34	22.4	6	10
Q2HJ26	PAICS	Phosphoribosylaminoimidazole carboxylase, phosphoribosylaminoimidazole	47.122	42.1	20	41	16.2	9	17
A5PKG2	PAIP1	PAIP1 protein	45.603	38.8	14	42	23	11	35
Q08E52	PAK1	Serine/threonine-protein kinase PAK	60.545	15.3	3	8	3.3	1	4
G3MZE2	PAK2	Uncharacterized protein	58.046	18.7	4	6	7.1	3	9
E1B7J7	PAK3	Uncharacterized protein	58.368	16.4	2	10	#N/A	#N/A	#N/A
F1MJW5	PALLD	Uncharacterized protein	125.18	3.2	3	4	0.8	1	1
F1N3Q4	PALM	Uncharacterized protein (Fragment)	42.37	7.5	2	2	#N/A	#N/A	#N/A
G3MWK1	PALM3	Uncharacterized protein	80.859	14.5	6	8	10.2	6	13
F2Z4I9	PAM16	Magmas-like protein	13.911	6.4	1	1	12	2	2
E1BML0	PAN2	Uncharacterized protein	135.13	0.9	1	1	#N/A	#N/A	#N/A
F1MLD0	PANK4	Uncharacterized protein	85.979	7	5	7	2.7	2	2
Q865R1	PAOX	Peroxisomal N(1)-acetyl-spermine/spermidine oxidase	56.324	24	11	16	14.1	7	14
A5D7N5	PAPOLG	PAPOLG protein	83.336	1.4	1	1	#N/A	#N/A	#N/A
Q2T9S9	PAQR7	Progestin and adipoQ receptor family member VII	39.983	2.9	1	1	#N/A	#N/A	#N/A
O02776	PARG	Poly(ADP-ribose) glycohydrolase	110.84	7.2	6	8	#N/A	#N/A	#N/A
Q5E946	PARK7	Protein DJ-1	20.035	85.2	19	117	69.8	15	95

				Biological Replicate 1			Biological Replicate 2		
**Accession number**	**Gene symbol**	**Discription**	**MW(kDa)**	**Sequence coverage(%)**	**Unique peptides**	**MS/MS count**	**Sequence coverage(%)**	**Unique peptides**	**MS/MS count**
Q2KHV4	PARL	Presenilins-associated rhomboid-like protein, mitochondrial	42.301	21.2	6	16	7.4	3	6
P18493	PARP1	Poly [ADP-ribose] polymerase 1	113.49	11.1	10	15	9.5	10	17
E1B7S1	PARP12	Uncharacterized protein	79.347	2	1	1	#N/A	#N/A	#N/A
A4FV46	PARP16	PARP16 protein	36.528	18.6	6	9	14.9	5	9
F1MHD4	PARS2	Uncharacterized protein	53.151	8.2	3	3	#N/A	#N/A	#N/A
F1N1E7	PATL1	Uncharacterized protein	86.886	2.2	2	2	1.3	1	1
F1N6C5	PATL2	Uncharacterized protein (Fragment)	58.17	29.7	1	46	17	6	19
F1MV85	PBDC1	Uncharacterized protein	24.93	28.8	6	9	#N/A	#N/A	#N/A
A6QQX0	PBK	PBK protein	36.441	34.9	11	24	18.8	7	16
Q2HJF4	PBLD	Phenazine biosynthesis-like domain-containing protein	31.883	16.3	4	4	#N/A	#N/A	#N/A
A6QLY7	PBXIP1	Pre-B-cell leukemia transcription factor-interacting protein 1	80.257	#N/A	#N/A	#N/A	1.5	1	1
Q29RK2	PC	Pyruvate carboxylase, mitochondrial	129.7	41.6	42	107	30.1	34	105
Q3ZBD3	PCBD1	Pterin-4-alpha-carbinolamine	11.985	64.4	7	22	49	5	9
Q5E9A3	PCBP1	Poly(rC)-binding protein 1	37.497	49.2	9	33	21.6	4	24
Q3SYT9	PCBP2	Poly(RC) binding protein 2	36.801	33.2	5	12	14.9	2	6
F6QNC4	PCCA	Uncharacterized protein	81.476	37.8	27	52	24.4	19	55
Q2TBR0	PCCB	Propionyl-CoA carboxylase beta chain, mitochondrial	58.31	54.9	22	40	35.1	15	51
F1MDS3	PCK2	Uncharacterized protein	70.649	16.2	9	14	6.1	4	7
P15246	PCMT1	Protein-L-isoaspartate(D-aspartate) O-methyltransferase	24.565	40.5	9	19	28.6	6	18
Q3ZBW4	PCNA	Proliferating cell nuclear antigen	28.748	64.4	16	59	35.2	10	37
Q32PF3	PCNP	PEST proteolytic signal-containing nuclear protein	18.988	5.1	1	1	#N/A	#N/A	#N/A
E1BKZ0	PCNT	Uncharacterized protein	327.24	0.5	2	2	#N/A	#N/A	#N/A
P02720	PCTP	Phosphatidylcholine transfer	24.643	28.6	5	9	#N/A	#N/A	#N/A
F1N2K1	PCYOX1	Uncharacterized protein	56.775	13.8	6	7	1.6	1	5
Q0P5H1	PCYOX1L	Prenylcysteine oxidase-like	54.437	17.9	9	13	11.4	6	25
Q0VCQ6	PDCD10	Programmed cell death 10	24.715	21.2	5	8	4.7	1	2

				Biological Replicate 1			Biological Replicate 2		
Accession number	Gene symbol	Discription	MW(kDa)	Sequence coverage(%)	Unique peptides	MS/MS count	Sequence coverage(%)	Unique peptides	MS/MS count
Q2HJH9	PDCD5	Programmed cell death protein 5	14.226	40	5	12	54.4	7	17
Q1LZ81	PDCD6	PDCD6 protein	21.737	39.7	6	10	7.4	2	2
E1BKM4	PDCD6IP	Uncharacterized protein (Fragment)	100.99	42	36	85	29.1	27	93
Q29RZ1	PDDC1	Parkinson disease 7 domain-containing protein 1	23.262	22.3	3	3	#N/A	#N/A	#N/A
Q08DF7	PDE12	2,5-phosphodiesterase 12	67.264	10.2	5	6	#N/A	#N/A	#N/A
A6QQQ0	PDE4A	PDE4A protein	70.684	1.6	1	1	#N/A	#N/A	#N/A
Q95142	PDE6D	Retinal rod rhodopsin-sensitive cGMP 3,5-cyclic phosphodiesterase	17.39	20.7	3	4	16	2	2
F1N5S7	PDF	Uncharacterized protein (Fragment)	25.763	5.2	1	1	#N/A	#N/A	#N/A
A7MB35	PDHA1	Pyruvate dehydrogenase E1 component subunit alpha, somatic form,	43.387	49	19	57	31	14	48
P11966	PDHB	Pyruvate dehydrogenase E1 component subunit beta, mitochondrial	39.126	49	14	36	21.2	7	33
P22439	PDHX	Pyruvate dehydrogenase protein X	53.885	24.6	12	22	17.4	9	34
A5D7E8	PDIA3	PDIA3 protein	56.929	77	49	418	58.4	35	324
F1MEN8	PDIA4	Protein disulfide-isomerase A4	72.497	61.1	52	238	50.9	40	229
Q2KIL5	PDIA5	Protein disulfide-isomerase A5	59.673	34.2	19	42	22.1	13	40
A6QNL5	PDIA6	PDIA6 protein (Fragment)	49.624	39.3	19	46	28.5	11	32
E1B8C5	PDK1	Uncharacterized protein	49.451	5	1	2	#N/A	#N/A	#N/A
A6QLG3	PDK3	PDK3 protein	47.913	47.2	17	37	23.9	11	35
A6H7E3	PDLIM1	PDZ and LIM domain 1	35.82	23.2	6	10	11	3	4
Q3T0C8	PDLIM2	PDZ and LIM domain protein 2	37.671	9.2	2	3	3.2	1	1
G3MY19	PDLIM5	Uncharacterized protein	58.837	15.6	5	12	7.4	3	5
P35816	PDP1	[Pyruvate dehydrogenase [acetyl-transferring]]-phosphatase 1, mitochondrial	61.184	18	8	9	3.2	2	2
G3N1T9	PDP2	Uncharacterized protein	59.579	6.8	3	4	4.1	2	5
F1MQJ7	PDPK1	Uncharacterized protein (Fragment)	63.295	19.4	11	13	12.6	6	12
O46504	PDPR	Pyruvate dehydrogenase phosphatase regulatory subunit, mitochondrial	98.856	14.2	12	19	8.7	8	14
A6QR05	PDSS2	PDSS2 protein	43.584	3	1	1	#N/A	#N/A	#N/A

				Biological Replicate 1			Biological Replicate 2		
Accession number	Gene symbol	Discription	MW(kDa)	Sequence coverage(%)	Unique peptides	MS/MS count	Sequence coverage(%)	Unique peptides	MS/MS count
A7MBC2	PDXDC1	Pyridoxal-dependent decarboxylase domain-containing protein 1	86.614	21.1	16	31	11.1	9	16
Q0II59	PDXK	Pyridoxal kinase	34.817	27.9	6	11	#N/A	#N/A	#N/A
Q3ZBF9	PDXP	Pyridoxal phosphate phosphatase	31.749	50	12	26	25	6	9
F1MT13	PDZD2	Uncharacterized protein (Fragment)	278.22	0.7	2	2	0.3	1	3
F1MWQ6	PDZD4	Uncharacterized protein (Fragment)	84.882	1.6	1	1	#N/A	#N/A	#N/A
E1BPC2	PDZD8	Uncharacterized protein	128.49	0.7	1	1	#N/A	#N/A	#N/A
Q3T0X8	PDZK1	Na(+)/H(+) exchange regulatory cofactor NHE-RF3	57.175	14.8	8	10	9	5	8
Q0VCY8	PEA15	Phosphoprotein enriched in	15.04	34.6	3	7	#N/A	#N/A	#N/A
P13696	PEBP1	Phosphatidylethanolamine-binding	20.985	83.4	15	84	64.2	10	99
A5D7S6	PEF1	PEF1 protein	30.178	10.1	3	5	#N/A	#N/A	#N/A
Q58DV0	PELO	Protein pelota homolog	43.421	22.1	10	13	7.5	3	4
E1BDV5	PELP1	Uncharacterized protein (Fragment)	122.12	4.1	3	4	2.7	3	4
F6Q234	PEPD	Uncharacterized protein	54.832	5.5	3	7	#N/A	#N/A	#N/A
E1BPU3	PEX1	Uncharacterized protein	142.15	#N/A	#N/A	#N/A	3.1	4	6
Q0VCP2	PEX11A	Peroxisomal membrane protein 11A	28.297	7.3	2	3	#N/A	#N/A	#N/A
Q148K5	PEX11B	Peroxisomal membrane protein 11B	28.278	22.1	6	9	#N/A	#N/A	#N/A
A1L567	PEX14	Peroxisomal biogenesis factor 14	32.546	16.6	6	9	11.5	4	11
Q2KII7	PEX16	Peroxisomal membrane protein PEX16	37.573	16.5	5	6	#N/A	#N/A	#N/A
Q3SZD1	PEX19	Peroxisomal biogenesis factor 19	32.667	#N/A	#N/A	#N/A	6	1	1
A6QPK4	PEX26	PEX26 protein	29.112	5.6	1	1	8.2	3	4
F1N5H3	PEX3	Peroxisomal biogenesis factor 3	42.102	16.6	6	10	7	3	7
Q1RMV0	PEX5	Peroxisomal targeting signal 1	70.877	25.9	11	16	6.9	4	10
E1B8F6	PEX6	Uncharacterized protein	103.78	5.1	4	7	5.9	6	8
F1N4K1	PFAS	Uncharacterized protein	144.17	10.5	9	16	#N/A	#N/A	#N/A
Q3SZE2	PFDN1	Prefoldin subunit 1	14.223	35.2	5	8	29.5	4	9
A1A4P5	PFDN2	Prefoldin subunit 2	16.648	44.8	6	8	28.6	4	13
F1MYY3	PFDN4	Prefoldin subunit 4 (Fragment)	15.303	9	1	3	#N/A	#N/A	#N/A
Q8HYI9	PFDN5	Prefoldin subunit 5	17.385	51.9	7	15	19.5	3	13
Q17Q89	PFDN6	Prefoldin subunit 6	14.554	14	2	3	22.5	3	6
A1A4J1	PFKL	6-phosphofructokinase, liver type	85.291	18.7	8	17	5.8	2	3

				Biological Replicate 1			Biological Replicate 2		
Accession number	Gene symbol	Discription	MW(kDa)	Sequence coverage(%)	Unique peptides	MS/MS count	Sequence coverage(%)	Unique peptides	MS/MS count
Q0IIG5	PFKM	6-phosphofructokinase, muscle type	85.293	12.3	7	11	5.8	3	7
Q09430	PFN2	Profilin-2	15.032	50.7	8	15	27.9	3	4
Q3SZ62	PGAM1	Phosphoglycerate mutase 1	28.852	59.4	13	48	36.2	8	20
E1BNQ0	PGAP1	Uncharacterized protein	104.84	3.5	4	7	5.3	6	8
Q3ZCI4	PGD	6-phosphogluconate dehydrogenase, decarboxylating	53.076	48.7	19	50	20.1	9	26
Q3T0P6	PGK1	Phosphoglycerate kinase 1	44.537	68.1	26	74	22.5	10	40
F1MM83	PGLS	6-phosphogluconolactonase	27.531	56.2	11	32	28.7	6	19
Q08DP0	PGM1	Phosphoglucomutase-1	61.589	31.5	16	25	15.8	9	14
A6QQ11	PGM2	PGM2 protein (Fragment)	69.581	7.5	5	7	2.6	2	3
A5PKH8	PGM2L1	PGM2L1 protein	70.419	29.6	17	23	13.3	9	16
F1MS56	PGM3	Uncharacterized protein	59.949	25.8	12	31	13.3	7	20
Q2T9S4	PGP	Phosphoglycolate phosphatase	34.32	34	10	28	19.3	5	13
Q17QC0	PGRMC1	Membrane-associated progesterone receptor component 1	21.622	21.1	5	8	28.4	5	14
A5PJQ6	PGRMC2	PGRMC2 protein	23.71	29.6	6	12	23.3	4	10
Q2KJ28	PGS1	CDP-diacylglycerol--glycerol-3-phosphate 3-	62.732	34.2	16	46	16.7	9	29
F1MVP7	PHACTR3	Phosphatase and actin regulator	59.743	14.7	6	12	10	5	10
Q3T165	PHB	Prohibitin	29.804	83.1	19	46	43.8	12	47
Q2HJ97	PHB2	Prohibitin-2	33.357	75.3	22	88	57.2	19	73
E1BKS5	PHEX	Uncharacterized protein	83.932	16.4	10	17	2.9	2	4
Q32PA4	PHPT1	14 kDa phosphohistidine phosphatase	13.93	65.6	10	20	42.4	5	11
Q0IIB1	PHYHD1	Phytanoyl-CoA dioxygenase domain-containing protein 1	32.539	27.5	5	17	12.4	3	8
Q32L96	PHYHIPL	Phytanoyl-CoA hydroxylase interacting protein-like	42.562	8.5	4	6	4.8	2	5
A6QLN5	PI4K2A	PI4K2A protein	54.084	36.3	15	23	16.3	8	21
F1N605	PICALM	Uncharacterized protein (Fragment)	66.95	10.1	3	5	#N/A	#N/A	#N/A
Q2T9M1	PICK1	PRKCA-binding protein	46.729	4.8	2	3	#N/A	#N/A	#N/A
Q3MHZ7	PIGK	GPI-anchor transamidase	45.172	8.9	3	5	2.5	1	2
E1BM45	PIGN	Uncharacterized protein	105.38	1.3	1	1	#N/A	#N/A	#N/A

				Biological Replicate 1			Biological Replicate 2		
Accession number	Gene symbol	Discription	MW(kDa)	Sequence coverage(%)	Unique peptides	MS/MS count	Sequence coverage(%)	Unique peptides	MS/MS count
Q32PC8	PIGP	Phosphatidylinositol glycan anchor biosynthesis, class P	14.717	#N/A	#N/A	#N/A	7.8	1	3
Q3SZL5	PIGS	GPI transamidase component PIG-S	61.785	14.2	7	13	5	3	6
F1N4W0	PIGT	Uncharacterized protein	65.337	10.9	6	7	8.3	5	11
F1N7F8	PIGU	Uncharacterized protein	50.011	2.5	1	2	4.8	2	4
Q1LZA4	PIGW	Phosphatidylinositol-glycan biosynthesis class W protein	56.298	#N/A	#N/A	#N/A	1.6	1	1
G3N1V8	PIGX	Uncharacterized protein (Fragment)	30.181	3.4	1	2	#N/A	#N/A	#N/A
Q0VCI6	PIH1D1	PIH1 domain-containing protein 1	32.415	13.4	3	6	6.2	2	3
F1MYM3	PIK3CB	Uncharacterized protein	122.94	2.3	2	2	1.4	2	2
P23727	PIK3R1	Phosphatidylinositol 3-kinase regulatory subunit alpha	83.496	4.4	2	2	3.2	1	1
Q5BIN5	PIN1	Peptidyl-prolyl cis-trans isomerase NIMA-interacting 1	18.273	26.4	3	4	4.9	1	3
G3X6D7	PIP4K2C	Phosphatidylinositol 5-phosphate 4-kinase type-2 gamma (Fragment)	41.206	20.9	8	10	6.3	3	5
A6QQH1	PIR	PIR protein	32.06	13.5	5	5	#N/A	#N/A	#N/A
Q2HJ54	PITPNA	Phosphatidylinositol transfer protein alpha isoform	31.849	60	16	38	28.5	8	14
Q9TR36	PITPNB	Phosphatidylinositol transfer protein beta isoform	31.539	13.3	2	2	#N/A	#N/A	#N/A
F1MFA3	PITRM1	Uncharacterized protein	116.38	7.6	6	7	4.6	5	7
E1B838	PIWIL3	Uncharacterized protein	101.05	37	31	61	17.9	18	37
A5D984	PKM2	Pyruvate kinase	57.948	81	45	234	51.2	26	113
Q8WMP9	PLA2G15	Group XV phospholipase A2	46.061	23.6	8	21	3.4	1	2
F1MNU5	PLA2G4F	Uncharacterized protein	94.347	22	14	28	9.5	8	18
A7Z055	PLAA	PLAA protein	87.12	60.6	42	183	36.1	25	90
F1MRH1	PLB1	Uncharacterized protein (Fragment)	160.79	4.1	5	5	#N/A	#N/A	#N/A
Q2KIY5	PLBD2	Putative phospholipase B-like 2	65.692	22.8	16	26	13.8	8	14
E1BHX7	PLCB3	Uncharacterized protein	139.27	3.2	3	3	#N/A	#N/A	#N/A
F1N4C7	PLCG2	Uncharacterized protein (Fragment)	140.24	16.9	17	29	2.9	3	9
F1MF11	PLCH1	Uncharacterized protein (Fragment)	186.92	1.1	2	6	0.5	1	5

				Biological Replicate 1			Biological Replicate 2		
Accession number	Gene symbol	Discription	MW(kDa)	Sequence coverage(%)	Unique peptides	MS/MS count	Sequence coverage(%)	Unique peptides	MS/MS count
Q2KJJ8	PLD3	Phospholipase D3	54.619	5.9	3	4	#N/A	#N/A	#N/A
E1BE10	PLD6	Mitochondrial cardiolipin hydrolase	25.15	15	3	5	7.3	2	2
F1MKZ7	PLEKHA7	Uncharacterized protein	132.09	4	4	4	0.9	1	2
A4FV32	PLEKHB2	PLEKHB2 protein	24.835	#N/A	#N/A	#N/A	3.2	1	2
F1MRP8	PLEKHG1	Uncharacterized protein	153.62	6.9	9	19	3.3	5	8
E1BEX3	PLEKHG4	Uncharacterized protein	132.21	1	1	2	#N/A	#N/A	#N/A
F1MQB0	PLIN2	Perilipin	45.106	60.1	21	50	25.3	12	38
Q2TA25	PLK1	Serine/threonine-protein kinase	68	13.5	7	11	9.8	6	12
F1MET0	PLOD3	Uncharacterized protein	85.895	3.6	2	3	#N/A	#N/A	#N/A
Q6Y1E2	PLP2	Proteolipid protein 2	16.645	#N/A	#N/A	#N/A	8.6	1	1
A6H742	PLS1	Plastin-1	70.531	32.7	15	26	9.2	5	17
A7E3Q8	PLS3	Plastin-3	70.797	6.2	0	1	4.1	1	1
Q3SZJ9	PMM2	Phosphomannomutase 2	27.997	28	7	8	8.5	2	3
Q0P5M8	PMPCA	Mitochondrial-processing peptidase subunit alpha	58.134	17.9	10	15	11	6	13
Q3SZ71	PMPCB	Mitochondrial-processing peptidase subunit beta	54.236	17.1	9	16	13.7	7	21
A7YY46	PNKD	Probable hydrolase PNKD	42.884	8.8	4	7	11.2	4	7
F1N3Q9	PNKP	Uncharacterized protein	57.361	4.2	2	2	2.9	1	1
F1MX01	PNLDC1	Uncharacterized protein	61.195	33.5	18	50	14.1	9	39
E1BMJ5	PNPLA6	Uncharacterized protein	145.63	1.9	2	2	4.8	5	5
E1BE78	PNPLA8	Uncharacterized protein	88.617	#N/A	#N/A	#N/A	2.4	2	2
Q5E9K3	PNPO	Pyridoxine-5-phosphate oxidase	30.365	6.9	1	1	3.1	1	1
E1BIQ0	PNPT1	Uncharacterized protein	85.901	17.1	14	21	11.1	10	19
F1MPI4	POFUT2	Uncharacterized protein	52.869	10	3	4	#N/A	#N/A	#N/A
F1MXS4	POLA2	DNA polymerase alpha subunit B	66.414	1.8	1	1	3	2	2
P28339	POLD1	DNA polymerase delta catalytic	123.71	9.5	9	13	6.6	8	9
P49004	POLD2	DNA polymerase delta subunit 2	50.997	25.2	9	15	3.8	2	2
G1K1U4	POLD3	DNA polymerase delta subunit 3	51.379	13.4	6	8	7.1	3	4
A5D9H9	POLDIP2	DNA polymerase delta interacting	41.942	25	6	8	#N/A	#N/A	#N/A
Q3SZN5	POLE3	DNA polymerase epsilon subunit 3	16.859	7.5	1	1	#N/A	#N/A	#N/A
A6QQ14	POLE4	DNA polymerase epsilon subunit 4	12.064	9.5	1	1	#N/A	#N/A	#N/A

				Biological Replicate 1			Biological Replicate 2		
Accession number	Gene symbol	Discription	MW(kDa)	Sequence coverage(%)	Unique peptides	MS/MS count	Sequence coverage(%)	Unique peptides	MS/MS count
E1BDI3	POLG	Uncharacterized protein	136.11	8	8	13	#N/A	#N/A	#N/A
Q0VC30	POLG2	DNA polymerase subunit gamma-2, mitochondrial	54.662	16.7	8	9	10.3	5	10
F1MHR0	POLI	Uncharacterized protein	78.592	3.7	3	3	1.3	1	2
F1MPJ5	POLM	Uncharacterized protein (Fragment)	52.876	4.7	2	2	#N/A	#N/A	#N/A
A5PJW8	POLR2B	DNA-directed RNA polymerase	133.87	2.6	3	6	0.8	1	2
Q3T0Q3	POLR2C	DNA-directed RNA polymerase II subunit RPB3	31.429	14.9	3	5	#N/A	#N/A	#N/A
Q2T9T3	POLR2E	DNA-directed RNA polymerases I, II, and III subunit RPABC1	24.481	4.3	1	1	4.3	1	1
Q5E9B8	POLR2G	DNA-directed RNA polymerase II subunit RPB7	19.294	4.7	1	1	#N/A	#N/A	#N/A
F2Z4H3	POLR2H	Uncharacterized protein	17.143	18	1	1	#N/A	#N/A	#N/A
Q5NDF2	POMGNT2	Protein O-linked-mannose beta-1,4-N-acetylglucosaminyltransferase 2	66.066	10.5	4	5	1.9	1	1
Q0MVC9	POMT1	POMT1 protein	82.745	1.8	1	1	#N/A	#N/A	#N/A
Q58DS7	PON2	Serum paraoxonase/arylesterase 2	39.421	5.1	2	4	7.9	2	2
Q3SYT8	POR	NADPH--cytochrome P450 reductase	77.02	44.4	25	63	25.4	20	50
P37980	PPA1	Inorganic pyrophosphatase	32.814	85.8	23	116	24.6	6	21
Q2KIV7	PPA2	Pyrophosphatase (Inorganic) 2	36.963	10.4	2	2	8.3	2	3
F1MV22	PPAT	Amidophosphoribosyltransferase	59.899	9.3	5	9	3.3	2	5
A6QPS1	PPCS	PPCS protein	22.591	21.1	6	11	17.8	5	13
F1MBQ1	PPEF1	Serine/threonine-protein phosphatase (Fragment)	74.829	1.5	1	4	1.5	1	2
E1BIA1	PPFIA3	Uncharacterized protein	133.74	0.8	1	3	#N/A	#N/A	#N/A
P62935	PPIA	Peptidyl-prolyl cis-trans isomerase	17.869	82.3	14	100	58.5	11	47
P80311	PPIB	Peptidyl-prolyl cis-trans isomerase	23.743	63.4	16	80	55.6	13	82
P26882	PPID	Peptidyl-prolyl cis-trans isomerase	40.62	51.1	19	57	30.3	12	35
P30404	PPIF	Peptidyl-prolyl cis-trans isomerase F, mitochondrial	22.019	38.9	6	14	23.1	5	13
Q0P5D0	PPIH	Peptidyl-prolyl cis-trans isomerase	19.208	28.2	5	13	#N/A	#N/A	#N/A
Q5E992	PPIL1	Peptidyl-prolyl cis-trans	18.237	#N/A	#N/A	#N/A	9	2	3

				Biological Replicate 1			Biological Replicate 2		
Accession number	Gene symbol	Discription	MW(kDa)	Sequence coverage(%)	Unique peptides	MS/MS count	Sequence coverage(%)	Unique peptides	MS/MS count
F1N6W2	PPIL4	Uncharacterized protein	57.121	3.7	2	2	2.2	1	1
F1MNG6	PPIP5K2	Uncharacterized protein	138.85	5.7	7	8	#N/A	#N/A	#N/A
F1N2K8	PPL	Uncharacterized protein (Fragment)	200.24	#N/A	#N/A	#N/A	1.1	2	2
O62829	PPM1A	Protein phosphatase 1A	42.529	18.1	3	4	#N/A	#N/A	#N/A
O62830	PPM1B	Protein phosphatase 1B	53.43	28.3	2	32	#N/A	#N/A	#N/A
F1N580	PPM1E	Uncharacterized protein (Fragment)	66.968	1.7	1	2	#N/A	#N/A	#N/A
E1BF95	PPM1F	Uncharacterized protein	45.897	#N/A	#N/A	#N/A	2.4	1	1
P79126	PPM1G	Protein phosphatase 1G	58.606	7.2	4	6	4.6	3	4
Q58DN4	PPME1	Protein phosphatase methylesterase	41.709	23.9	9	21	8.7	3	4
Q3T0E7	PPP1CA	Serine/threonine-protein phosphatase PP1-alpha catalytic	37.512	#N/A	#N/A	#N/A	9.4	2	4
F1MW57	PPP1CB	Serine/threonine-protein phosphatase (Fragment)	36.849	36.8	10	16	#N/A	#N/A	#N/A
P61287	PPP1CC	Serine/threonine-protein phosphatase PP1-gamma catalytic	36.983	#N/A	#N/A	#N/A	6.2	1	1
F1N3L1	PPP1R12A	Uncharacterized protein	79.342	#N/A	#N/A	#N/A	5.1	3	3
E1BHY2	PPP1R13B	Uncharacterized protein	118.57	0.7	1	2	#N/A	#N/A	#N/A
F1MNS9	PPP1R3D	Protein phosphatase 1 regulatory	32.325	8.7	2	2	#N/A	#N/A	#N/A
Q3T0W4	PPP1R7	Protein phosphatase 1 regulatory	41.388	26.1	8	12	7.2	3	3
P67774	PPP2CA	Serine/threonine-protein phosphatase 2A catalytic subunit	35.594	65.7	1	3	11.3	1	9
Q0P594	PPP2CB	Serine/threonine-protein phosphatase 2A catalytic subunit	35.561	68.3	2	47	11.3	1	2
Q32PI5	PPP2R1A	Serine/threonine-protein phosphatase 2A 65 kDa regulatory	65.29	54	21	85	22.8	17	36
F1MQN0	PPP2R1B	Uncharacterized protein	76.752	22	6	6	#N/A	#N/A	#N/A
F1MG56	PPP2R2A	Serine/threonine-protein phosphatase 2A 55 kDa regulatory	53.009	19.5	5	7	6.3	3	4
Q2KJ44	PPP2R4	Serine/threonine-protein phosphatase 2A activator	36.882	55.9	15	59	24.4	7	17
F1N1S8	PPP2R5D	Uncharacterized protein (Fragment)	72.695	46.3	27	57	19.8	11	36

				Biological Replicate 1			Biological Replicate 2		
Accession number	Gene symbol	Discription	MW(kDa)	Sequence coverage(%)	Unique peptides	MS/MS count	Sequence coverage(%)	Unique peptides	MS/MS count
A4FV68	PPP2R5E	Serine/threonine-protein phosphatase 2A 56 kDa regulatory	54.699	12.8	4	4	#N/A	#N/A	#N/A
A6H772	PPP4C	Serine/threonine-protein phosphatase 4 catalytic subunit	35.08	9.1	1	2	6.2	2	2
F1MRS1	PPP4R1	Uncharacterized protein (Fragment)	103.07	11.7	9	15	4.2	4	10
F1N719	PPP5C	Serine/threonine-protein	56.881	49.1	24	52	16	10	20
Q2KIC7	PPP6C	Serine/threonine-protein	35.144	12.1	3	5	#N/A	#N/A	#N/A
G3N214	PPP6R1	Uncharacterized protein	91.999	5	3	6	#N/A	#N/A	#N/A
P45478	PPT1	Palmitoyl-protein thioesterase 1	34.142	12.1	4	7	12.1	4	9
Q1JQA0	PPT2	Lysosomal thioesterase PPT2	34.626	7.5	2	3	#N/A	#N/A	#N/A
E1BEW5	PPTC7	Uncharacterized protein	32.816	11.7	3	3	8.8	2	3
Q2KHX3	PRAF2	PRA1 family protein 2	19.174	16.3	3	4	16.3	3	10
F1MRT3	PRC1	Uncharacterized protein	70.36	25	16	21	5.5	4	9
Q2TA14	PRCP	Lysosomal Pro-X carboxypeptidase	56.67	4.6	2	3	4.8	2	4
Q5E947	PRDX1	Peroxiredoxin-1	22.209	69.3	17	195	46.2	10	148
Q9BGI3	PRDX2	Peroxiredoxin-2	21.946	72.9	15	209	33.7	8	67
P35705	PRDX3	Thioredoxin-dependent peroxide reductase, mitochondrial	28.195	55.6	12	89	31.5	8	65
Q9BGI2	PRDX4	Peroxiredoxin-4	30.741	63.9	10	52	31.8	5	39
Q9BGI1	PRDX5	Peroxiredoxin-5, mitochondrial	23.253	#N/A	#N/A	#N/A	39.3	8	22
O77834	PRDX6	Peroxiredoxin-6	25.067	85.3	23	154	55.8	13	48
E1BL12	PREB	Uncharacterized protein	45.788	17.9	7	14	22.5	7	16
F1MQV7	PRKAA2	Uncharacterized protein (Fragment)	59.383	2.3	1	1	#N/A	#N/A	#N/A
P00517	PRKACA	cAMP-dependent protein kinase catalytic subunit alpha	40.619	5.4	2	2	#N/A	#N/A	#N/A
P05131	PRKACB	cAMP-dependent protein kinase catalytic subunit beta	40.593	#N/A	#N/A	#N/A	2.6	1	1
P58108	PRKAG1	5-AMP-activated protein kinase subunit gamma-1	37.497	14.8	5	5	13.6	5	7
P00514	PRKAR1A	cAMP-dependent protein kinase type I-alpha regulatory subunit	42.892	17.4	5	7	3.7	2	3

				Biological Replicate 1			Biological Replicate 2		
Accession number	Gene symbol	Discription	MW(kDa)	Sequence coverage(%)	Unique peptides	MS/MS count	Sequence coverage(%)	Unique peptides	MS/MS count
P00515	PRKAR2A	cAMP-dependent protein kinase type II-alpha regulatory subunit	45.093	16.5	4	6	2.2	1	3
P31322	PRKAR2B	cAMP-dependent protein kinase type II-beta regulatory subunit	46.335	8.1	2	2	8.6	3	3
F1MZX0	PRKCD	Protein kinase C delta type	77.327	2.7	1	1	1.5	1	3
Q28034	PRKCSH	Glucosidase 2 subunit beta	60.151	27.8	18	50	17.8	14	53
A0JNH7	PRKCZ	Protein kinase C, zeta	67.732	2.5	1	1	#N/A	#N/A	#N/A
Q2HJ92	PRKRA	Interferon-inducible double-stranded RNA-dependent protein	34.413	9.6	3	3	3.2	1	4
F1N443	PRMT5	Protein arginine N-	72.644	40.3	22	50	11.6	9	20
F1MQX7	PRMT7	Protein arginine N-	78.722	7.8	4	6	#N/A	#N/A	#N/A
G3N3Y2	PRODH	Proline dehydrogenase 1,	60.062	2.6	1	1	#N/A	#N/A	#N/A
F1MK52	PROM1	Uncharacterized protein	99.624	2.3	2	3	3	3	4
E1BM92	PROM2	Uncharacterized protein	91.52	2	1	1	3	3	3
Q3T0G5	PROSC	Proline synthase co-transcribed bacterial homolog protein	29.943	7.3	2	3	#N/A	#N/A	#N/A
A8E4M9	PRPF39	PRPF39 protein	64.282	4.9	3	3	#N/A	#N/A	#N/A
Q3MHE2	PRPF4	U4/U6 small nuclear	58.398	1.9	1	1	#N/A	#N/A	#N/A
Q2KJJ0	PRPF6	Pre-mRNA-processing factor 6	106.68	2.1	2	2	1.8	2	2
Q2HJ58	PRPS1	Ribose-phosphate pyrophosphokinase	34.834	25.5	6	11	23	6	20
G3MY14	PRPS2	Uncharacterized protein (Fragment)	23.556	15.3	1	1	10.6	1	3
Q08DW2	PRPSAP1	Phosphoribosyl pyrophosphate synthase-associated protein 1	39.423	26.4	6	12	14.9	5	9
A2VDS0	PRPSAP2	Phosphoribosyl pyrophosphate synthase-associated protein 2	40.714	7.6	1	1	#N/A	#N/A	#N/A
F1N116	PRSS53	Uncharacterized protein	59.242	6.5	2	5	2.9	1	2
E1BMA1	PRTG	Uncharacterized protein (Fragment)	114.33	0.9	1	2	#N/A	#N/A	#N/A
Q5E9Y6	PRUNE	Protein prune homolog	50.08	2	1	3	#N/A	#N/A	#N/A
Q9XT96	PSEN2	Presenilin-2	50.271	2	1	1	#N/A	#N/A	#N/A
Q3T0X5	PSMA1	Proteasome subunit alpha type-1	29.585	44.1	12	23	31.6	9	22
Q3T0Y5	PSMA2	Proteasome subunit alpha type-2	25.898	34.6	6	10	10.7	2	4
Q58DU5	PSMA3	Proteasome subunit alpha type-3	28.405	37.3	11	18	17.6	6	14

				Biological Replicate 1			Biological Replicate 2		
Accession number	Gene symbol	Discription	MW(kDa)	Sequence coverage(%)	Unique peptides	MS/MS count	Sequence coverage(%)	Unique peptides	MS/MS count
Q3ZCK9	PSMA4	Proteasome subunit alpha type-4	29.483	36.4	8	11	19.5	6	17
Q5E987	PSMA5	Proteasome subunit alpha type-5	26.411	45.6	9	22	20.3	4	7
G5E5C3	PSMA6	Proteasome subunit alpha type	27.284	34.3	9	19	27.8	7	24
Q3ZBG0	PSMA7	Proteasome subunit alpha type-7	27.869	54.4	11	21	27.4	6	20
G5E589	PSMB1	Proteasome subunit beta type	26.318	31.1	6	13	27.8	6	13
Q5E9K0	PSMB2	Proteasome subunit beta type-2	22.896	31.8	7	9	22.4	5	10
P33672	PSMB3	Proteasome subunit beta type-3	22.993	43.4	9	25	8.3	3	4
Q3T108	PSMB4	Proteasome subunit beta type-4	29.031	23.1	4	4	7.2	2	5
Q32KL2	PSMB5	Proteasome subunit beta type-5	28.609	35.4	9	21	20.2	5	13
Q3MHN0	PSMB6	Proteasome subunit beta type-6	25.542	21.3	6	11	17.6	4	9
F1MBI1	PSMB7	Proteasome subunit beta type	29.996	23.1	4	9	6.9	2	4
Q3T112	PSMB8	Proteasome subunit beta type-8	30.268	#N/A	#N/A	#N/A	2.9	1	1
Q3SZC2	PSMB9	Proteasome subunit beta type-9	23.404	4.6	1	2	8.7	2	3
A4FUZ3	PSMC1	Proteasome (Prosome, macropain) 26S subunit, ATPase, 1	49.184	31.1	11	21	20.2	8	22
Q5E9F9	PSMC2	26S protease regulatory subunit 7	48.633	64.9	26	50	36.5	17	30
F1MWE0	PSMC3	Uncharacterized protein	51.054	39.7	14	24	18.2	9	17
F1MG70	PSMC4	26S protease regulatory subunit 6B	47.269	24.2	9	16	18	7	19
P62194	PSMC5	26S protease regulatory subunit 8	45.626	33.7	11	34	19	6	25
F1MLV1	PSMC6	26S protease regulatory subunit 10B	44.172	44.5	15	28	29.3	12	26
A7MBA2	PSMD1	PSMD1 protein	105.89	22.1	17	31	13.7	13	33
Q2KI42	PSMD11	26S proteasome non-ATPase regulatory subunit 11	47.463	46.9	19	42	30.8	14	33
Q2KJ25	PSMD12	26S proteasome non-ATPase regulatory subunit 12	53.052	32.2	13	25	9.2	3	6
Q5E964	PSMD13	26S proteasome non-ATPase regulatory subunit 13	42.866	48.1	16	24	23.9	10	20
Q17QP9	PSMD14	Proteasome (Prosome, macropain) 26S subunit, non-ATPase, 14	34.577	22.3	5	12	8.7	3	11
P56701	PSMD2	26S proteasome non-ATPase regulatory subunit 2	100.26	42.5	30	65	19.3	16	28

				Biological Replicate 1			Biological Replicate 2		
Accession number	Gene symbol	Discription	MW(kDa)	Sequence coverage(%)	Unique peptides	MS/MS count	Sequence coverage(%)	Unique peptides	MS/MS count
Q2KJ46	PSMD3	26S proteasome non-ATPase regulatory subunit 3	60.956	45.5	23	58	23.6	14	37
F1MXE4	PSMD6	26S proteasome non-ATPase regulatory subunit 6	45.591	38	18	41	21.6	11	21
Q3ZBD0	PSMD7	26S proteasome non-ATPase regulatory subunit 7	36.754	29.2	8	21	9.9	4	14
Q3SZ19	PSMD9	26S proteasome non-ATPase regulatory subunit 9	24.266	48	8	17	24.4	5	10
Q4U5R3	PSME1	Proteasome activator complex	28.663	32.1	8	13	17.3	4	4
F1MU19	PSME2	Proteasome activator complex subunit 2 (Fragment)	28.42	24.4	5	6	#N/A	#N/A	#N/A
Q3SX30	PSMF1	Proteasome inhibitor PI31 subunit	29.704	13.7	3	7	#N/A	#N/A	#N/A
Q0P5F2	PSMG1	Proteasome assembly chaperone 1	33.015	9.7	3	3	6.9	2	3
Q2NL24	PSMG2	Proteasome assembly chaperone 2	29.224	20.1	6	9	#N/A	#N/A	#N/A
Q2NKS3	PSMG3	Proteasome assembly chaperone 3	12.901	10.7	1	2	#N/A	#N/A	#N/A
F1MDD0	PSMG4	Uncharacterized protein	13.747	27.6	2	2	#N/A	#N/A	#N/A
Q1LZD9	PSPC1	Paraspeckle component 1	58.274	11	3	6	6	3	4
Q2KHU0	PSPH	Phosphoserine phosphatase	24.85	34.7	7	23	13.8	3	5
A8YXY5	PTBP1	PTBP1 protein	47.027	12.6	4	6	#N/A	#N/A	#N/A
F6Q0P6	PTBP2	Uncharacterized protein	25.636	11	1	1	#N/A	#N/A	#N/A
Q2KI62	PTCD3	Pentatricopeptide repeat domain-containing protein 3, mitochondrial	77.83	1.9	1	1	#N/A	#N/A	#N/A
Q2KHY9	PTDSS1	Phosphatidylserine synthase 1	55.415	2.7	1	1	#N/A	#N/A	#N/A
F1N1J6	PTGES2	Prostaglandin E synthase 2	41.738	11.6	3	3	4.8	2	2
Q3ZBF7	PTGES3	Prostaglandin E synthase 3	18.697	47.5	8	29	34.4	6	26
F1MT41	PTGFRN	Uncharacterized protein (Fragment)	98.895	27.8	26	66	25.7	26	94
Q3SZJ4	PTGR1	Prostaglandin reductase 1	35.706	#N/A	#N/A	#N/A	4.3	1	1
Q32L99	PTGR2	Prostaglandin reductase 2	38.4	45	12	31	11.1	4	14
F1MS29	PTK2	Uncharacterized protein	119.8	5.1	4	5	#N/A	#N/A	#N/A
A7YY55	PTPLAD1	Very-long-chain (3R)-3-hydroxyacyl-[acyl-carrier protein] dehydratase	43.133	32	10	25	13.5	5	19

				Biological Replicate 1			Biological Replicate 2		
Accession number	Gene symbol	Discription	MW(kDa)	Sequence coverage(%)	Unique peptides	MS/MS count	Sequence coverage(%)	Unique peptides	MS/MS count
Q2KIP8	PTPLB	Very-long-chain (3R)-3-hydroxyacyl-[acyl-carrier protein] dehydratase	28.422	#N/A	#N/A	#N/A	8.3	2	2
Q2NKZ7	PTPMT1	Protein tyrosine phosphatase, mitochondrial 1	29.262	7.8	2	3	8.1	3	4
A6QQN2	PTPN1	Tyrosine-protein phosphatase non-receptor type	49.037	5.4	2	4	6.4	3	5
F1N339	PTPN11	Tyrosine-protein phosphatase non-receptor type	68.441	5	3	4	#N/A	#N/A	#N/A
F1N6W1	PTPN9	Uncharacterized protein (Fragment)	71.322	3.2	2	2	#N/A	#N/A	#N/A
F1MJR2	PTPRD	Uncharacterized protein (Fragment)	199.3	0.7	1	1	#N/A	#N/A	#N/A
Q3ZBL5	PTRH2	Peptidyl-tRNA hydrolase 2,	19.29	24	3	4	29.1	4	6
Q3SZ85	PTRHD1	Putative peptidyl-tRNA hydrolase	15.972	43.6	6	14	27.9	4	8
F1MX94	PTS	6-pyruvoyl tetrahydrobiopterin	16.363	28.3	4	4	6.2	1	2
Q3SZY3	PTTG1	Securin	22.131	7.4	3	4	#N/A	#N/A	#N/A
F1N238	PUF60	Poly(U)-binding-splicing factor	57.043	#N/A	#N/A	#N/A	2.8	2	2
A6QP68	PUSL1	tRNA pseudouridine synthase	34.022	14.7	5	10	9.6	3	3
Q2HJ56	PWP1	Periodic tryptophan protein 1	55.864	#N/A	#N/A	#N/A	3.8	2	2
A5PJQ7	PXK	PXK protein	65.075	2.8	1	1	#N/A	#N/A	#N/A
Q2KIY1	PXMP2	Peroxisomal membrane protein 2	22.258	21.4	6	14	17.3	5	11
F1MFD1	PXN	Uncharacterized protein (Fragment)	63.856	7	4	8	5.1	4	7
Q58DT4	PYCR1	Pyrroline-5-carboxylate reductase 1, mitochondrial	33.453	#N/A	#N/A	#N/A	3.4	1	2
Q58D08	PYCRL	Pyrroline-5-carboxylate reductase 3	32.035	40.3	11	17	14.9	4	8
Q3B7M9	PYGB	Glycogen phosphorylase, brain form	96.339	35.5	27	48	10.3	8	16
Q0VCM4	PYGL	Glycogen phosphorylase, liver form	97.455	#N/A	#N/A	#N/A	2.2	1	1
Q3T0Z7	QDPR	Dihydropteridine reductase	25.504	62.4	11	51	40.1	7	33
Q0V8G3	QPCTL	Glutaminyl-peptide cyclotransferase-like protein	42.897	7.8	4	6	8.1	3	6
P83862	QRFP	Orexigenic neuropeptide QRFP	14.566	9	2	2	#N/A	#N/A	#N/A
F1MM32	QSOX1	Sulfhydryl oxidase	62.886	3.9	2	3	1.6	1	1
E1BBS6	QSOX2	Sulfhydryl oxidase (Fragment)	75.364	13.7	8	10	3.7	3	3
A6H7F4	QTRTD1	QTRTD1 protein	37.511	4.7	1	2	#N/A	#N/A	#N/A

				Biological Replicate 1			Biological Replicate 2		
Accession number	Gene symbol	Discription	MW(kDa)	Sequence coverage(%)	Unique peptides	MS/MS count	Sequence coverage(%)	Unique peptides	MS/MS count
A6QLS9	RAB10	RAB10 protein	22.541	63	11	42	32	6	17
Q3MHP2	RAB11B	Ras-related protein Rab-11B	24.488	66.1	13	47	52.3	11	59
E1BNE5	RAB11FIP5	Uncharacterized protein	137.79	#N/A	#N/A	#N/A	1.3	2	3
Q3ZBG1	RAB14	RAB14 protein	23.897	73	12	25	15.8	4	9
Q1RMR4	RAB15	Ras-related protein Rab-15	24.424	20.8	3	5	17.5	3	5
Q0IIG8	RAB18	Ras-related protein Rab-18	22.991	48.5	9	14	41.7	8	30
A1L528	RAB1A	RAB1A, member RAS oncogene family	22.677	78.5	7	22	60	6	74
Q2HJH2	RAB1B	Ras-related protein Rab-1B	22.202	81.6	7	90	62.2	5	16
Q17R06	RAB21	Ras-related protein Rab-21	24.146	45.5	11	28	29.3	7	23
F1MHP4	RAB22A	Uncharacterized protein	21.798	60.3	10	15	30.4	5	10
E1B771	RAB24	Uncharacterized protein	23.383	12.7	1	2	4.4	1	3
Q58DW6	RAB25	Ras-related protein Rab-25	23.536	13.6	2	3	22.1	4	11
A6QLB1	RAB27A	RAB27A protein	24.978	32.6	7	16	31.7	7	34
Q3SWY9	RAB28	Ras-related protein Rab-28	24.743	5	1	1	#N/A	#N/A	#N/A
Q148J4	RAB2A	RAB2A, member RAS oncogene family	20.85	64.6	5	51	34.4	2	29
E1BC58	RAB2B	Uncharacterized protein	24.167	47.2	2	3	35.2	2	5
Q17QB7	RAB30	Ras-related protein Rab-30	23.058	20.7	5	7	7.9	1	2
E1BA60	RAB32	Uncharacterized protein	24.89	51.6	10	36	33.3	7	20
F1MSJ9	RAB35	Uncharacterized protein	23.011	26.4	5	14	21.9	5	16
E1BLD5	RAB37	Uncharacterized protein	24.348	6.5	1	1	#N/A	#N/A	#N/A
G3X7D3	RAB39	Uncharacterized protein	24.98	6	1	1	#N/A	#N/A	#N/A
Q17QU4	RAB39B	Ras-related protein Rab-39B	24.608	7.5	1	1	#N/A	#N/A	#N/A
P11023	RAB3A	Ras-related protein Rab-3A	24.954	25.5	1	3	14.1	1	7
P10948	RAB3B	Ras-related protein Rab-3B	24.766	17.4	2	2	#N/A	#N/A	#N/A
E1BF18	RAB3C	Ras-related protein Rab-3C	24.842	38.5	5	14	19.7	3	8
E1BNX1	RAB3D	Uncharacterized protein	24.387	33.3	3	14	28.8	4	18
A4FUE5	RAB3GAP1	RAB3GAP1 protein	110.71	16	14	23	8.8	9	11
G5E601	RAB3GAP2	Uncharacterized protein	155.62	12.9	17	27	7.3	10	16
Q2KI32	RAB3IP	RAB3A interacting protein (Rabin3)	51.026	32.8	14	29	12	6	13
Q2TBH7	RAB4A	Ras-related protein Rab-4A	24.392	24.8	4	7	9.6	2	4
Q0IIG7	RAB5A	Ras-related protein Rab-5A	23.688	57.2	5	14	30.2	2	2
F1MNI4	RAB5B	Uncharacterized protein (Fragment)	25.153	64.2	7	63	38.9	4	43

				Biological Replicate 1			Biological Replicate 2		
Accession number	Gene symbol	Discription	MW(kDa)	Sequence coverage(%)	Unique peptides	MS/MS count	Sequence coverage(%)	Unique peptides	MS/MS count
Q58DS9	RAB5C	Ras-related protein Rab-5C	23.466	30.1	2	3	26.9	2	5
F1MBF6	RAB6A	Uncharacterized protein	23.518	51	3	4	34.6	2	3
A6QR46	RAB6B	Ras-related protein Rab-6B	23.461	61.5	6	39	51.4	4	41
A4FV54	RAB8A	Ras-related protein Rab-8A	23.684	36.7	6	15	26.6	3	14
Q08DW1	RAB9A	RAB9A, member RAS oncogene family	22.909	77.8	12	30	23.2	5	18
Q0VCB4	RAB9B	RAB9B, member RAS oncogene family	22.705	31.8	3	4	#N/A	#N/A	#N/A
Q1RMH4	RABAC1	Prenylated Rab acceptor protein 1	20.626	#N/A	#N/A	#N/A	9.2	2	3
F1MQH8	RABEP1	Uncharacterized protein	99.516	#N/A	#N/A	#N/A	1.2	1	1
A4FUG8	RABEP2	Rab GTPase-binding effector protein	65.602	6.8	4	4	2.7	2	3
F1N746	RABGAP1	Uncharacterized protein	121	6.1	6	7	#N/A	#N/A	#N/A
A6QPS3	RABGAP1L	RABGAP1L protein	27.035	9	1	1	#N/A	#N/A	#N/A
Q2TBX8	RABGEF1	RABGEF1 protein	56.937	8.1	4	4	7.3	4	8
Q5EA80	RABGGTA	Geranylgeranyl transferase type-2 subunit alpha	64.945	5.3	3	3	#N/A	#N/A	#N/A
Q5E9B3	RABGGTB	Geranylgeranyl transferase type-2 subunit beta	36.972	#N/A	#N/A	#N/A	3.3	1	1
P62998	RAC1	Ras-related C3 botulinum toxin	21.45	34.9	2	12	19.8	3	16
F1MCR0	RAC3	Uncharacterized protein (Fragment)	20.177	22.8	1	3	#N/A	#N/A	#N/A
E1BJP2	RACGAP1	Uncharacterized protein	71.087	17.2	10	12	9.8	7	11
Q58DB5	RAD18	Postreplication repair protein	37.715	2.7	1	1	#N/A	#N/A	#N/A
Q29RK4	RAD23B	UV excision repair protein RAD23	43.117	26	12	29	20.8	11	30
Q5E9A4	RAE1	mRNA export factor	40.967	22.8	9	11	14.9	5	12
A7E3S4	RAF1	RAF proto-oncogene serine/threonine-protein kinase	72.874	1.7	1	1	#N/A	#N/A	#N/A
Q3ZCK2	RALA	Uncharacterized protein	23.54	47.1	2	3	27.7	3	14
A5D977	RALB	RALB protein	23.438	56.3	7	45	31.1	5	63
F1MCG4	RALGAPB	Uncharacterized protein	168.85	2.1	3	3	1.1	2	3
Q5E952	RALY	RNA binding protein (Autoantigenic, hnRNP-associated with lethal yellow) short isoform	30.142	#N/A	#N/A	#N/A	6.7	2	2
Q3T054	RAN	GTP-binding nuclear protein Ran	24.423	45.8	10	37	30.6	7	46
Q3T0M7	RANBP1	Ran-specific GTPase-activating	23.712	18.4	4	9	#N/A	#N/A	#N/A

				Biological Replicate 1			Biological Replicate 2		
Accession number	Gene symbol	Discription	MW(kDa)	Sequence coverage(%)	Unique peptides	MS/MS count	Sequence coverage(%)	Unique peptides	MS/MS count
F1MFD5	RANGAP1	Uncharacterized protein	62.941	12.8	8	12	9.6	6	9
F1MW14	RANGRF	Ran guanine nucleotide release	20.714	25.8	3	4	#N/A	#N/A	#N/A
P62833	RAP1A	Ras-related protein Rap-1A	20.987	#N/A	#N/A	#N/A	30.4	1	1
P61223	RAP1B	Ras-related protein Rap-1b	20.825	47.3	9	25	37.5	2	27
F6RPT3	RAP1GDS1	Rap1 GTPase-GDP dissociation stimulator 1 (Fragment)	60.889	34.3	16	25	14.5	7	20
A4IFU2	RAP2B	RAP2B protein	20.488	12.6	1	1	#N/A	#N/A	#N/A
Q08DI5	RAP2C	Ras-related protein Rap-2c	20.745	10.9	1	5	6	1	3
F1MJY2	RAPGEF1	Uncharacterized protein (Fragment)	137.63	2.9	3	3	1.7	2	3
E1BPM7	RAPH1	Uncharacterized protein	124.13	1.6	2	2	1.6	2	2
Q0P5D6	RARRES1	Retinoic acid receptor responder (Tazarotene induced) 1	32.771	21.1	6	38	5.9	3	5
A7YW98	RARS	Arginine--tRNA ligase, cytoplasmic	75.56	59.2	38	124	33.5	24	78
Q0P5H7	RARS2	Probable arginine--tRNA ligase, mitochondrial	65.63	7.6	4	5	4.2	3	3
F1MC29	RASSF1	Uncharacterized protein	31.209	3	1	5	#N/A	#N/A	#N/A
Q3MHL3	RBBP4	Histone-binding protein RBBP4	47.655	19.5	2	4	#N/A	#N/A	#N/A
Q3SWX8	RBBP7	Histone-binding protein RBBP7	47.844	40	8	28	20.2	3	22
F6RBQ9	RBM3	Uncharacterized protein	17.496	19.4	2	4	#N/A	#N/A	#N/A
Q3MHX3	RBM4	RNA-binding protein 4	40.077	25.7	8	14	13.3	5	14
Q0P5L0	RBM42	RNA-binding protein 42	47.187	3.8	1	1	#N/A	#N/A	#N/A
P02694	RBP1	Retinol-binding protein 1	15.823	76.3	11	24	31.9	5	9
F1N2U8	RBPMS2	Uncharacterized protein (Fragment)	22.414	37.9	6	9	7.4	2	5
Q2HJI9	RBX1	Ring-box 1	12.274	13	3	8	13	2	7
E1BKF7	RCBTB1	Uncharacterized protein	58.18	3	1	1	2.1	1	2
A6QL85	RCC2	RCC2 protein	55.942	11.3	5	6	4.2	2	2
A4IFE2	RCHY1	RCHY1 protein	30.168	5.4	1	4	#N/A	#N/A	#N/A
G3N2L2	RCN1	Uncharacterized protein (Fragment)	29.312	17.9	4	5	8.1	2	4
Q0VCQ9	RCN2	Reticulocalbin 2, EF-hand calcium binding domain	36.909	16.4	4	6	5	1	1
E1BM93	RDH11	Uncharacterized protein	35.487	14.7	4	6	6.3	2	4
Q17QC2	RDH13	Retinol dehydrogenase 13 (All-	36.272	#N/A	#N/A	#N/A	3.9	1	3

				Biological Replicate 1			Biological Replicate 2		
Accession number	Gene symbol	Discription	MW(kDa)	Sequence coverage(%)	Unique peptides	MS/MS count	Sequence coverage(%)	Unique peptides	MS/MS count
Q17QW3	RDH14	Retinol dehydrogenase 14 (All-trans/9-cis/11-cis)	36.625	12.2	4	6	8.3	3	5
Q32LP2	RDX	Radixin	68.567	36	6	6	22.1	6	9
Q2KI30	REEP2	Receptor expression-enhancing	28.439	5.9	2	2	#N/A	#N/A	#N/A
Q3ZCI8	REEP4	Receptor expression-enhancing	29.597	5.8	2	3	9.7	3	8
F1MUS6	RELN	Reelin (Fragment)	368.91	1	2	2	0.5	2	3
F6Q4T4	REPS1	Uncharacterized protein	86.77	8.7	7	8	2.5	2	2
A5PJ65	RER1	Protein RER1	22.863	28.1	5	7	9.2	2	8
A2VE52	REXO2	Oligoribonuclease, mitochondrial	26.885	46.4	10	32	27.8	6	15
F1N4T1	RFC1	Uncharacterized protein	48.717	5.3	2	3	3	1	1
Q05B83	RFC2	Replication factor C subunit 2	38.729	9.9	4	5	#N/A	#N/A	#N/A
Q2TBV1	RFC3	Replication factor C subunit 3	40.465	14.3	4	4	5.3	2	3
Q29RS9	RFC4	Replication factor C (Activator 1) 4, 37kDa	37.097	13.6	5	7	11.9	4	6
Q32PI3	RFC5	Replication factor C (Activator 1) 5, 36.5kDa	35.675	17.1	5	7	5.4	2	4
E1BNC6	RFT1	Uncharacterized protein	60.521	1.7	1	2	#N/A	#N/A	#N/A
E1BGG6	RFX5	Uncharacterized protein	65.286	1.8	1	1	#N/A	#N/A	#N/A
G3N0I0	RGAG4	Uncharacterized protein	65.571	1.2	1	1	#N/A	#N/A	#N/A
Q9TTJ5	RGN	Regucalcin	33.307	17.1	5	8	8.7	2	3
Q2T9P3	RGP1	Retrograde Golgi transport protein RGP1 homolog	43.097	8.6	4	5	6.3	3	4
O46471	RGS16	Regulator of G-protein signaling 16	22.635	#N/A	#N/A	#N/A	11.9	2	2
Q0P5H5	RGS2	Regulator of G-protein signaling 2	24.209	5.7	1	1	#N/A	#N/A	#N/A
P61585	RHOA	Transforming protein RhoA	21.768	44	9	30	21.8	5	16
Q1RMI2	RHOG	Ras homolog gene family, member G	21.28	11	1	1	11	2	2
Q2HJF8	RHOT1	Mitochondrial Rho GTPase 1	72.077	21.4	11	18	7.9	3	7
Q5E9M9	RHOT2	Mitochondrial Rho GTPase 2	68.151	19.3	8	11	12.1	6	15
Q08DT8	RINT1	RAD50 interactor 1	90.879	3.8	4	5	#N/A	#N/A	#N/A
A5D7A4	RIT1	RIT1 protein	25.055	5.5	1	1	#N/A	#N/A	#N/A
Q32KL4	RMDN1	Regulator of microtubule dynamics protein 1	35.923	8.8	1	1	6	2	4

				Biological Replicate 1			Biological Replicate 2		
Accession number	Gene symbol	Discription	MW(kDa)	Sequence coverage(%)	Unique peptides	MS/MS count	Sequence coverage(%)	Unique peptides	MS/MS count
Q1JQC5	RMDN3	Regulator of microtubule dynamics protein 3	51.905	44.2	17	53	24.4	11	41
F1MS81	RMND1	Uncharacterized protein	51.811	12	6	7	12	5	12
F1N2X2	RNASEH1	Uncharacterized protein	31.168	14.3	3	3	3.6	1	2
Q0III8	RNASET2	RNASET2 protein (Fragment)	32.829	9.3	2	2	#N/A	#N/A	#N/A
Q4U5R4	RNF114	RING finger protein 114	25.669	51.7	12	58	48.7	11	44
F1MM46	RNF121	Uncharacterized protein	34.554	3.1	1	3	5.4	2	5
Q0VD51	RNF13	E3 ubiquitin-protein ligase RNF13	42.688	7.9	2	2	#N/A	#N/A	#N/A
E1B7X3	RNF146A	E3 ubiquitin-protein ligase RNF146-	37.542	3.5	1	1	#N/A	#N/A	#N/A
F1MK05	RNF170	E3 ubiquitin-protein ligase RNF170	29.689	23.6	5	10	8.9	2	4
A9Q1J6	RNF18	Ring finger protein 18	51.584	6	3	3	#N/A	#N/A	#N/A
A4IFC2	RNF185	RNF185 protein	20.428	7.3	2	3	#N/A	#N/A	#N/A
A2VDP1	RNF20	E3 ubiquitin-protein ligase BRE1A	113.66	2.2	2	2	#N/A	#N/A	#N/A
F1MLH7	RNF214	Uncharacterized protein	77.691	#N/A	#N/A	#N/A	1.4	1	1
A6QP97	RNF220	E3 ubiquitin-protein ligase RNF220	27.528	4.1	1	1	#N/A	#N/A	#N/A
Q5E9J6	RNF34	E3 ubiquitin-protein ligase RNF34	41.787	10.9	3	5	#N/A	#N/A	#N/A
Q0P5F4	RNF7	Ring finger protein 7	12.679	18.6	2	2	#N/A	#N/A	#N/A
H9GW43	RNH1	Uncharacterized protein	25.835	71.4	11	29	29.9	5	7
E1BBM0	RNMTL1	Uncharacterized protein	46.299	2.6	1	1	#N/A	#N/A	#N/A
G3X743	RNPEP	Uncharacterized protein (Fragment)	56.56	2	1	1	#N/A	#N/A	#N/A
F1MF50	ROCK1	Rho-associated protein kinase 1	159.76	2.9	2	2	#N/A	#N/A	#N/A
F1N319	ROCK2	Rho-associated protein kinase 2	155.89	3.3	2	5	0.6	1	2
Q0VCV0	RPA1	Replication protein A1, 70kDa	68.213	44.2	26	67	24.8	15	40
Q2KI86	RPA2	Replication protein A2, 32kDa	29.163	39.3	7	21	15.9	4	12
Q3SZ11	RPA3	Replication protein A3, 14kDa	13.508	51.2	4	9	21.5	2	3
A0JN53	RPAP1	RNA polymerase II-associated	152.86	8.7	12	20	1.2	2	4
F6RRD7	RPAP2	Putative RNA polymerase II subunit B1 CTD phosphatase RPAP2	68.697	#N/A	#N/A	#N/A	3.5	2	2
F1N0V7	RPAP3	Uncharacterized protein	75.594	13.2	8	16	7.2	5	7
G5E6S3	RPE	Uncharacterized protein (Fragment)	20.706	#N/A	#N/A	#N/A	4.8	1	2
G3X872	RPH3AL	Rab effector Noc2	31.915	14.4	4	8	2.7	1	2
Q3T186	RPIA	Ribose-5-phosphate isomerase	28.753	33	8	16	6.8	2	3

				Biological Replicate 1			Biological Replicate 2		
Accession number	Gene symbol	Discription	MW(kDa)	Sequence coverage(%)	Unique peptides	MS/MS count	Sequence coverage(%)	Unique peptides	MS/MS count
Q9XSI3	RPL10	60S ribosomal protein L10	24.603	36	8	14	9.8	2	5
Q5E9E6	RPL10A	60S ribosomal protein L10a	24.831	52.5	13	20	27.2	6	15
Q3T087	RPL11	60S ribosomal protein L11	20.252	25.3	5	6	21.3	4	11
P61284	RPL12	60S ribosomal protein L12	17.818	67.3	9	21	67.3	10	31
Q56JZ1	RPL13	60S ribosomal protein L13	24.291	30.3	8	19	27	6	14
Q3SZ90	RPL13A	60S ribosomal protein L13a	23.523	31	8	15	29.1	7	22
G8JKV5	RPL14	60S ribosomal protein L14	23.365	24.4	5	9	21.6	1	12
Q5EAD6	RPL15	60S ribosomal protein L15	24.146	34.8	7	11	11.3	3	7
Q3T025	RPL17	60S ribosomal protein L17	21.397	27.2	6	10	10.3	2	2
Q5E973	RPL18	60S ribosomal protein L18	21.535	38.3	6	11	23.9	4	9
Q3T003	RPL18A	60S ribosomal protein L18a	20.762	26.1	5	11	22.2	4	10
Q3T0W9	RPL19	60S ribosomal protein L19	23.466	20.9	4	7	8.2	2	4
Q861S4	RPL21	Similar to ribosomal protein L21 (Fragment)	18.607	18.8	3	4	#N/A	#N/A	#N/A
F1N301	RPL22	Uncharacterized protein	14.759	23.4	3	7	25	3	13
Q3T057	RPL23	60S ribosomal protein L23	14.865	50	7	11	12.9	2	9
Q24JY1	RPL23A	60S ribosomal protein L23a	17.695	26.9	4	5	21.2	4	6
Q862I1	RPL24	60S ribosomal protein L24	17.779	23.6	4	6	10.8	2	3
P61257	RPL26	60S ribosomal protein L26	17.258	21.4	4	4	17.2	3	7
P61356	RPL27	60S ribosomal protein L27	15.798	40.4	5	6	25	4	7
G1K1B4	RPL27A	60S ribosomal protein L27a	16.446	14.3	2	4	#N/A	#N/A	#N/A
Q3T0L7	RPL28	60S ribosomal protein L28	15.692	41.6	8	14	43.8	7	16
P39872	RPL3	60S ribosomal protein L3	46.023	30	14	21	19.6	11	28
Q3T0D5	RPL30	60S ribosomal protein L30	12.784	33.9	5	6	45.2	4	7
Q56JX3	RPL31	60S ribosomal protein L31	14.463	24.8	3	4	28	4	11
Q3SZQ6	RPL32	60S ribosomal protein L32	15.874	20	3	3	15.6	3	4
F1ML72	RPL34	Uncharacterized protein (Fragment)	14.617	32.8	5	10	13.3	2	7
Q3MHM7	RPL35	60S ribosomal protein L35	14.565	18.7	2	2	14.6	2	8
Q56JY1	RPL35A	60S ribosomal protein L35a	12.552	45.5	7	11	31.8	4	11
Q3T171	RPL36	60S ribosomal protein L36	12.224	9.5	1	2	9.5	1	1
G8JL02	RPL36AHNRNPH2	Uncharacterized protein	12.144	#N/A	#N/A	#N/A	9.6	1	2
Q3MIC0	RPL37A	60S ribosomal protein L37a	10.275	28.3	2	2	18.5	2	2

				Biological Replicate 1			Biological Replicate 2		
Accession number	Gene symbol	Discription	MW(kDa)	Sequence coverage(%)	Unique peptides	MS/MS count	Sequence coverage(%)	Unique peptides	MS/MS count
Q3T051	RPL39	60S ribosomal protein L39	6.4066	19.6	1	3	#N/A	#N/A	#N/A
Q58DW0	RPL4	60S ribosomal protein L4	47.411	42.4	7	30	13.7	6	11
Q58DW5	RPL5	60S ribosomal protein L5	34.344	21.9	6	10	10.4	3	5
Q58DQ3	RPL6	60S ribosomal protein L6	32.689	25.8	7	15	27.9	9	31
Q58DT1	RPL7	60S ribosomal protein L7	29.168	37.5	11	19	19.4	6	17
Q2TBQ5	RPL7A	60S ribosomal protein L7a	30.025	32.3	10	17	22.6	7	21
Q3T0S6	RPL8	60S ribosomal protein L8	28.024	30	9	14	24.1	8	15
Q3SYR7	RPL9	60S ribosomal protein L9	21.876	39.6	7	10	16.1	3	9
Q95140	RPLP0	60S acidic ribosomal protein P0	34.37	53.8	14	37	38.7	9	40
Q56K14	RPLP1	60S acidic ribosomal protein P1	11.514	14	1	6	#N/A	#N/A	#N/A
P42899	RPLP2	60S acidic ribosomal protein P2	11.702	16.5	1	1	31.3	3	7
A3KN04	RPN1	RPN1 protein	68.351	56	28	67	29.1	18	71
Q3SZI6	RPN2	Dolichyl-diphosphooligosaccharide--protein glycosyltransferase subunit	69.213	33	17	50	20.9	11	38
A6QLW3	RPRD1B	RPRD1B protein	36.899	7.1	2	2	#N/A	#N/A	#N/A
Q3T0F4	RPS10	40S ribosomal protein S10	18.898	60.6	11	21	33.3	8	16
Q3T0V4	RPS11	40S ribosomal protein S11	18.431	50.6	8	12	27.8	6	17
Q76I81	RPS12	40S ribosomal protein S12	14.515	40.2	5	14	40.2	6	16
Q56JX8	RPS13	40S ribosomal protein S13	17.222	59.6	11	21	41.7	8	16
Q3T076	RPS14	Ribosomal protein S14	16.776	28	4	6	14.6	3	5
Q56K10	RPS15	40S ribosomal protein S15	17.026	17.9	2	4	#N/A	#N/A	#N/A
Q76I82	RPS15A	40S ribosomal protein S15a	14.839	48.5	7	11	38.5	6	11
Q3T0X6	RPS16	40S ribosomal protein S16	16.445	58.2	11	21	50.7	9	23
A5PK63	RPS17	40S ribosomal protein S17	15.582	48.9	4	12	16.3	3	6
Q3T0R1	RPS18	40S ribosomal protein S18	17.718	52.6	11	19	25	5	12
Q32PD5	RPS19	40S ribosomal protein S19	16.06	54.5	11	26	47.6	11	23
O18789	RPS2	40S ribosomal protein S2	31.235	51.2	17	28	25.3	9	25
Q3ZBH8	RPS20	40S ribosomal protein S20	13.373	32.8	4	13	28.6	4	10
Q56JX5	RPS25	40S ribosomal protein S25	13.742	24	3	7	29.6	5	12
Q56JV1	RPS26	40S ribosomal protein S26	13.015	20.9	2	3	20.9	2	3
P62992	RPS27A	Ubiquitin-40S ribosomal protein	17.965	50	1	89	#N/A	#N/A	#N/A
Q3T0B7	RPS27L	40S ribosomal protein S27-like	9.4631	22.6	2	2	9.5	1	2

				Biological Replicate 1			Biological Replicate 2		
Accession number	Gene symbol	Discription	MW(kDa)	Sequence coverage(%)	Unique peptides	MS/MS count	Sequence coverage(%)	Unique peptides	MS/MS count
Q56JX6	RPS28	40S ribosomal protein S28	7.8409	17.4	1	2	15.9	1	1
P62276	RPS29	40S ribosomal protein S29	6.6767	19.6	1	2	#N/A	#N/A	#N/A
Q3T169	RPS3	40S ribosomal protein S3	26.688	67.5	16	39	62.6	17	53
Q56JV9	RPS3A	40S ribosomal protein S3a	29.945	39	13	25	38.6	10	19
P79103	RPS4	40S ribosomal protein S4	29.597	50.6	13	32	#N/A	#N/A	#N/A
Q5E988	RPS5	40S ribosomal protein S5	22.876	31.9	9	15	26.5	8	21
Q5E995	RPS6	40S ribosomal protein S6	28.666	34.5	8	15	13.7	3	6
A4IFF4	RPS6KA1	Ribosomal protein S6 kinase	82.724	31.8	12	33	15.5	5	23
F1MZW8	RPS6KA3	Ribosomal protein S6 kinase	83.666	29.1	12	19	10.7	2	6
F1MNP4	RPS6KB1	Ribosomal protein S6 kinase	59.109	16	9	19	6.9	3	8
A6H769	RPS7	40S ribosomal protein S7	22.127	22.7	5	6	12.9	3	7
G8JKY0	RPS8	40S ribosomal protein S8 (Fragment)	24.074	51.2	9	17	31.4	6	17
A6QLG5	RPS9	40S ribosomal protein S9	22.591	46.9	14	28	34.5	9	25
P26452	RPSA	40S ribosomal protein SA	32.884	44.4	10	30	20.3	6	29
F1MG06	RPTOR	Uncharacterized protein	149.48	0.7	1	1	#N/A	#N/A	#N/A
A7MB47	RQCD1	Cell differentiation protein RCD1	33.601	23.1	6	9	3.7	1	2
Q3SX43	RRAGA	Ras-related GTP-binding protein A	36.566	25.9	8	13	13.1	4	7
Q0VD29	RRAGC	Ras-related GTP binding C	44.292	28.8	5	29	22.8	2	17
E1BK97	RRAGD	Uncharacterized protein	45.158	11.8	1	1	18.6	1	1
A5PKL2	RRAS2	RRAS2 protein	23.399	23	4	6	22.1	5	11
F1MDC1	RRBP1	Uncharacterized protein	166.91	3.9	3	4	5	8	13
F1MSF1	RRM1	Ribonucleoside-diphosphate	89.99	24.3	18	38	10.6	10	26
E1BFQ8	RRM2B	Uncharacterized protein	40.859	11.4	3	3	#N/A	#N/A	#N/A
E1BNA1	RRP12	Uncharacterized protein	137.23	#N/A	#N/A	#N/A	0.9	1	2
Q5E9C0	RSU1	Ras suppressor protein 1	31.537	33.2	7	16	6.1	2	2
Q2HJ88	RTCA	RNA 3-terminal phosphate cyclase	39.251	44	13	35	17.8	6	13
Q5E9T9	RTCB	tRNA-splicing ligase RtcB homolog	55.199	40.2	19	38	19.4	10	16
F1N405	RTN4	Reticulon	130.41	7.2	7	14	3.6	4	8
E1BNY5	RUFY3	Uncharacterized protein	55.581	3.4	2	2	5.2	2	3
A7MBG8	RUVBL1	RUVBL1 protein	50.153	38.4	13	29	12.1	5	17
Q2TBU9	RUVBL2	RuvB-like 2	51.169	43.8	18	32	23.5	11	35
Q32L76	SAA4	Serum amyloid A-4 protein	14.687	8.5	1	1	13.2	2	3

				Biological Replicate 1			Biological Replicate 2		
Accession number	Gene symbol	Discription	MW(kDa)	Sequence coverage(%)	Unique peptides	MS/MS count	Sequence coverage(%)	Unique peptides	MS/MS count
A6QL88	SACM1L	Phosphatidylinositide phosphatase	67.092	36.8	20	45	16	10	33
A2VE14	SAE1	SUMO-activating enzyme subunit 1	38.305	44.2	14	25	23.1	9	18
E1BDE7	SAFB2	Uncharacterized protein	108.52	1.4	2	2	#N/A	#N/A	#N/A
E1BJZ5	SALL4	Uncharacterized protein	110.79	3.5	4	4	2	2	3
F1N763	SAMD12	Uncharacterized protein	18.204	14.3	2	2	#N/A	#N/A	#N/A
Q2HJ55	SAMM50	Sorting and assembly machinery component 50 homolog	52.042	52.7	21	56	35	15	38
A7Z051	SAPS3	SAPS3 protein	97.612	2.5	2	2	#N/A	#N/A	#N/A
Q3T0D7	SAR1A	GTP-binding protein SAR1a	22.409	21.2	5	15	20.7	4	21
Q08E24	SARAF	Store-operated calcium entry-associated regulatory factor	36.314	2.4	1	1	#N/A	#N/A	#N/A
Q9GMB8	SARS	Serine--tRNA ligase, cytoplasmic	58.604	32.9	16	38	20	10	20
F1N0I9	SART3	Uncharacterized protein (Fragment)	74.322	6.1	4	7	#N/A	#N/A	#N/A
Q3SWZ6	SBDS	Ribosome maturation protein SBDS	28.718	33.6	8	16	21.6	6	10
E1BMP8	SBNO1	Uncharacterized protein	154.51	2.7	2	2	#N/A	#N/A	#N/A
A6H7G3	SC4MOL	SC4MOL protein	35.06	7.2	2	2	#N/A	#N/A	#N/A
F1MYK6	SCAF8	Uncharacterized protein (Fragment)	140.15	1.1	1	1	#N/A	#N/A	#N/A
Q3T0D2	SCAMP1	Secretory carrier membrane protein	39.986	5.4	2	2	8.8	2	3
A6QR35	SCAMP2	SCAMP2 protein	36.703	15.9	4	12	16.5	5	15
Q58DR5	SCAMP3	Secretory carrier-associated membrane protein 3	38.277	15.6	5	13	17	5	11
Q58DF6	SCAMP4	Secretory carrier-associated membrane protein 4	25.595	24.8	4	8	8.7	2	7
Q17QF8	SCAMP5	Secretory carrier-associated membrane protein 5	26.116	8.1	2	3	#N/A	#N/A	#N/A
A6QQP4	SCARB2	SCARB2 protein	53.984	24.1	10	22	12.1	6	15
Q3T067	SCCPDH	Saccharopine dehydrogenase-like oxidoreductase	47.319	34.3	10	25	13.3	5	14
G3N2Z2	SCD	Acyl-CoA desaturase (Fragment)	29.863	#N/A	#N/A	#N/A	5.1	1	1
E1BG76	SCFD1	Uncharacterized protein	72.406	49	26	85	14.8	9	19
A0JN38	SCFD2	Sec1 family domain containing 2	75.07	7.7	5	6	3.1	2	4
A1A4J8	SCO1	Protein SCO1 homolog, mitochondrial	33.657	#N/A	#N/A	#N/A	5.2	2	2

				Biological Replicate 1			Biological Replicate 2		
Accession number	Gene symbol	Discription	MW(kDa)	Sequence coverage(%)	Unique peptides	MS/MS count	Sequence coverage(%)	Unique peptides	MS/MS count
A6H784	SCO2	Protein SCO2 homolog, mitochondrial	29.789	#N/A	#N/A	#N/A	5.6	1	2
P07857	SCP2	Non-specific lipid-transfer protein	58.577	14.5	8	9	5.9	3	5
Q2NKZ9	SCPEP1	Serine carboxypeptidase 1	50.84	2.9	1	2	#N/A	#N/A	#N/A
P83939	SCRN1	Secernin-1	46.201	10.1	3	5	#N/A	#N/A	#N/A
Q17QS0	SCRN3	Secernin-3	48.233	#N/A	#N/A	#N/A	2.6	1	5
Q3ZBX5	SCYE1	Small inducible cytokine subfamily E, member 1 (Endothelial monocyte-	35.412	32.3	9	13	22.4	7	16
F1MVK2	SCYL1	N-terminal kinase-like protein	89.234	26	18	36	7.3	6	13
F1MID0	SCYL2	Uncharacterized protein	103.16	1.3	1	1	1.7	2	2
Q3SZA6	SDCBP	Syndecan binding protein (Syntenin)	32.389	45.6	9	27	22.8	5	5
Q3T083	SDF2L1	Stromal cell-derived factor 2-like protein 1	23.557	62.9	8	18	3.6	1	5
G3MY67	SDHA	Succinate dehydrogenase [ubiquinone] flavoprotein subunit, mitochondrial (Fragment)	74.28	48.6	23	57	27.9	18	54
Q3T189	SDHB	Succinate dehydrogenase [ubiquinone] iron-sulfur subunit,	31.518	27.9	8	14	16.8	6	12
P35720	SDHC	Succinate dehydrogenase cytochrome b560 subunit, mitochondrial	18.389	12.4	2	3	#N/A	#N/A	#N/A
Q95123	SDHD	Succinate dehydrogenase [ubiquinone] cytochrome b small	17.124	4.4	1	1	#N/A	#N/A	#N/A
Q17QH8	SDR39U1	Epimerase family protein SDR39U1	31.334	#N/A	#N/A	#N/A	3.1	1	1
P67810	SEC11A	Signal peptidase complex catalytic subunit SEC11A	20.625	15.1	3	4	9.5	2	6
Q2KI36	SEC11C	SEC11 homolog C (S. cerevisiae)	21.63	27.1	6	13	8.3	2	6
Q3ZCC9	SEC13	Protein SEC13 homolog	35.471	26.1	8	15	20.5	5	9
P58875	SEC14L2	SEC14-like protein 2	46.2	6.5	2	2	#N/A	#N/A	#N/A
F1N6K7	SEC16A	Uncharacterized protein	246.28	0.9	3	4	1.5	4	5
F1N2T5	SEC22A	Uncharacterized protein	35.053	6.2	1	1	#N/A	#N/A	#N/A
F1MLE8	SEC22B	Uncharacterized protein	28.709	47.4	12	49	29.1	9	21
F1MVW5	SEC23A	Protein transport protein Sec23A	86.545	35.1	15	45	10.7	7	16
Q3SZN2	SEC23B	Protein transport protein Sec23B	86.461	19.2	6	13	#N/A	#N/A	#N/A

				Biological Replicate 1			Biological Replicate 2		
Accession number	Gene symbol	Discription	MW(kDa)	Sequence coverage(%)	Unique peptides	MS/MS count	Sequence coverage(%)	Unique peptides	MS/MS count
E1BKW5	SEC23IP	Uncharacterized protein	111.63	6.2	6	8	2.4	3	7
A6QNT8	SEC24A	Protein transport protein Sec24A	120.1	4.5	4	5	#N/A	#N/A	#N/A
E1BPR4	SEC24B	Uncharacterized protein	135.4	1.7	2	3	1.6	2	3
E1BIU0	SEC24C	Uncharacterized protein	117.99	18.4	16	27	7.5	8	11
F1MV07	SEC24D	Uncharacterized protein	112.46	9.6	8	10	0.9	1	2
E1BMP2	SEC31A	Uncharacterized protein	126.34	17.2	20	34	7.5	9	15
E1BLS3	SEC31B	Uncharacterized protein	128.72	21.8	24	45	7.7	9	16
Q5EA68	SEC61A1	Protein transport protein Sec61 subunit alpha isoform 1	52.224	9.5	2	3	11.6	2	16
Q2KHX4	SEC61A2	Protein transport protein Sec61 subunit alpha isoform 2	52.247	11.3	3	12	11.6	2	5
Q2NKT5	SEC61B	Sec61 beta subunit	9.9443	10.4	1	1	#N/A	#N/A	#N/A
F1MUP3	SEC62	Uncharacterized protein	45.708	9	4	5	9	5	8
E1B7B1	SEC63	Uncharacterized protein	87.882	7.9	5	10	3.3	2	2
A7YY75	SEH1L	Nucleoporin SEH1	39.64	23.6	6	7	8.6	3	5
F1MGJ5	SEL1L	Uncharacterized protein (Fragment)	94.29	13.8	8	18	6.5	4	9
Q2KJ32	SELENBP1	Selenium-binding protein 1	52.555	33.5	12	23	4.4	2	3
E1BKK7	SENP8	Uncharacterized protein (Fragment)	25.045	28.2	4	10	6.4	2	11
Q0VC82	SEPHS1	Selenide, water dikinase 1	42.88	2.6	1	1	#N/A	#N/A	#N/A
E1BHE5	SEPHS2	Uncharacterized protein	43.006	4.4	1	3	#N/A	#N/A	#N/A
A5PJU9	SEPT1	Septin-1	41.982	18.5	6	14	5.4	2	4
A2VE99	SEPT11	Septin-11	48.991	24	8	15	17.2	6	9
A8YXY3	SEPT15	15 kDa selenoprotein	17.974	23.5	3	11	9.9	2	2
Q2NKY7	SEPT2	Septin-2	41.571	25.2	6	8	5.8	2	2
Q08DM7	SEPT3	Neuronal-specific septin-3	40.537	15.4	4	4	2.5	1	1
F1MBT8	SEPT7	Septin-7	48.901	25.5	8	18	9.3	4	7
A1L5B7	SERBP1	SERPINE1 mRNA binding protein 1	42.329	8.8	2	3	10.4	3	4
F1MBF9	SERINC1	Serine incorporator 1	49.61	3.8	1	2	#N/A	#N/A	#N/A
F1N5T9	SERINC5	Uncharacterized protein (Fragment)	51.697	6.5	3	5	3.9	2	5
A6QPQ2	SERPINA38	Serpin A3-8	46.963	7.9	1	2	#N/A	#N/A	#N/A
O02739	SERPINB6	Serpin B6	42.56	34.4	6	28	8.5	2	12
P13909	SERPINE1	Plasminogen activator inhibitor 1	45.371	19.2	5	13	#N/A	#N/A	#N/A

				Biological Replicate 1			Biological Replicate 2		
Accession number	Gene symbol	Discription	MW(kDa)	Sequence coverage(%)	Unique peptides	MS/MS count	Sequence coverage(%)	Unique peptides	MS/MS count
F1MZX2	SERPINE2	Uncharacterized protein	44.3	21.4	9	24	14.9	7	16
Q2KJH6	SERPINH1	Serpin H1	46.506	#N/A	#N/A	#N/A	1.9	1	1
F1MCF1	SET	Uncharacterized protein (Fragment)	29.718	29.1	6	13	3.9	1	2
E1BAS6	SETX	Uncharacterized protein	301.19	0.3	1	2	#N/A	#N/A	#N/A
A2VDM7	SF1	Splicing factor 1	68.329	1.6	1	1	#N/A	#N/A	#N/A
G3MW14	SF3A2	Splicing factor 3A subunit 2	43.974	#N/A	#N/A	#N/A	5.8	2	2
Q3SWY7	SF3A3	Splicing factor 3a, subunit 3	58.905	5.4	2	2	#N/A	#N/A	#N/A
F1MX61	SF3B1	Uncharacterized protein	145.86	1.2	2	2	1.6	2	2
Q2KIQ7	SF3B14	Splicing factor 3B, 14 kDa subunit	14.585	9.6	1	1	#N/A	#N/A	#N/A
F1MC31	SF3B2	Uncharacterized protein	100.31	4.4	3	5	#N/A	#N/A	#N/A
A0JN52	SF3B3	Splicing factor 3B subunit 3	135.58	5.4	6	11	1.9	3	3
Q0VC36	SFN	14-3-3 protein sigma	27.849	19.8	2	7	14.9	2	7
E1BQ37	SFPQ	Uncharacterized protein	76.248	10.6	6	12	7.8	4	7
A4FUC6	SFRS9	SFRS9 protein	25.478	9	2	2	#N/A	#N/A	#N/A
F1N0V9	SFT2D3	Uncharacterized protein (Fragment)	22.494	#N/A	#N/A	#N/A	4.1	1	2
Q5E9M8	SFXN1	Sideroflexin-1	35.689	32.6	9	17	19.3	5	13
Q5EA43	SFXN2	Sideroflexin-2	36.301	6.5	2	4	6.2	2	2
A6QP55	SFXN3	Sideroflexin-3	35.707	8.7	2	3	11.8	3	4
Q3T0M2	SFXN4	Sideroflexin-4	35.426	21.7	7	20	11.2	3	5
E1BNX6	SGMS2	Uncharacterized protein	42.44	2.7	1	1	2.7	1	2
A5D788	SGPL1	SGPL1 protein	63.566	12.7	7	7	5.8	4	9
A6QPY4	SGSH	SGSH protein	35.044	9.2	4	4	3.9	1	3
Q2KI13	SGSM3	Small G protein signaling modulator	85.009	7.8	5	5	#N/A	#N/A	#N/A
Q32LM2	SGTA	Small glutamine-rich tetratricopeptide repeat-containing	34.213	38.7	12	21	20.4	6	15
A4IFC4	SH3BGRL2	SH3 domain-binding glutamic acid-rich-like protein 2	12.292	62.6	5	8	#N/A	#N/A	#N/A
Q3ZCL8	SH3BGRL3	SH3 domain-binding glutamic acid-rich-like protein 3	10.438	38.7	3	5	#N/A	#N/A	#N/A
Q2KJA1	SH3GL1	Endophilin-A2	41.454	23.1	6	10	12	3	9
Q08DX1	SH3GL2	SH3-domain GRB2-like 2	39.886	25	4	5	9.1	1	1
Q08DK5	SH3GLB2	Endophilin-B2	44.05	29.4	9	12	18.5	6	10

				Biological Replicate 1			Biological Replicate 2		
Accession number	Gene symbol	Discription	MW(kDa)	Sequence coverage(%)	Unique peptides	MS/MS count	Sequence coverage(%)	Unique peptides	MS/MS count
E1BDF2	SHARPIN	Sharpin	43.393	#N/A	#N/A	#N/A	2.9	1	1
Q0IIE2	SHC1	SHC-transforming protein 1	51.631	14.6	6	8	11.4	5	11
E1B738	SHCBP1	Uncharacterized protein	76.003	9.9	7	10	4.9	4	5
Q3SZ20	SHMT2	Serine hydroxymethyltransferase, mitochondrial	55.605	51.8	20	39	21.2	11	36
A6QLV3	SHOC2	Leucine-rich repeat protein SHOC-2	64.859	48.3	22	72	19.2	11	25
F1N0F2	SIAE	Uncharacterized protein	59.672	1.9	1	1	#N/A	#N/A	#N/A
F1MVZ5	SIDT2	Uncharacterized protein	51.291	3.7	2	2	3.3	1	3
Q58DH7	SIGMAR1	Sigma non-opioid intracellular	24.914	16.1	3	4	11.2	2	5
Q32KV6	SIL1	Nucleotide exchange factor SIL1	52.581	30.3	13	64	14.3	7	25
F1MTR3	SIN3A	Uncharacterized protein	145.19	1.6	2	2	1.4	2	2
F6QK60	SIRT2	Uncharacterized protein	43.276	4.4	1	1	#N/A	#N/A	#N/A
G5E521	SIRT3	Uncharacterized protein	36.991	#N/A	#N/A	#N/A	6.3	2	3
Q3ZBQ0	SIRT5	NAD-dependent protein deacylase sirtuin-5, mitochondrial	33.988	50.3	12	34	36.1	10	33
F1MUQ7	SKA2	Spindle and kinetochore-associated protein 2	14.111	11.6	1	1	#N/A	#N/A	#N/A
E1BMS0	SKIV2L	Uncharacterized protein	137.65	11.5	10	10	0.8	1	2
Q3ZCF3	SKP1	S-phase kinase-associated protein 1	18.658	50.3	6	16	#N/A	#N/A	#N/A
A7MB09	SKP2	SKP2 protein	47.819	6.1	2	2	#N/A	#N/A	#N/A
F1MQ42	SLBP	Uncharacterized protein	31.509	13.1	4	5	3.3	2	2
F1MUK9	SLC10A5	Uncharacterized protein	30.361	4.5	1	1	#N/A	#N/A	#N/A
F1N2X1	SLC12A6	Uncharacterized protein	127.47	8.4	11	21	7	9	20
F1N140	SLC12A7	Uncharacterized protein (Fragment)	118.94	#N/A	#N/A	#N/A	1.1	1	1
E1BLP2	SLC12A9	Uncharacterized protein	96.183	15.1	10	15	3.1	3	4
A6QQL0	SLC15A4	Solute carrier family 15 member 4	61.379	9	5	6	4.8	3	6
Q3MHW6	SLC16A1	Monocarboxylate transporter 1	54.297	6.8	3	4	4.8	3	4
Q17QR6	SLC16A13	Monocarboxylate transporter 13	44.713	3.8	1	2	3.8	1	1
F1MXZ2	SLC16A3	Uncharacterized protein	49.493	6.1	3	4	4.3	2	3
A2VDL4	SLC1A4	Neutral amino acid transporter A	56.007	13.6	5	10	4	2	2
Q95JC7	SLC1A5	Neutral amino acid transporter B(0)	56.447	4.5	2	4	#N/A	#N/A	#N/A

				Biological Replicate 1			Biological Replicate 2		
Accession number	Gene symbol	Discription	MW(kDa)	Sequence coverage(%)	Unique peptides	MS/MS count	Sequence coverage(%)	Unique peptides	MS/MS count
F1MXY8	SLC25A1	Tricarboxylate transport protein, mitochondrial (Fragment)	34.017	41.3	12	40	30	10	38
P22292	SLC25A11	Mitochondrial 2-oxoglutarate/malate carrier protein	34.171	42.7	12	32	26.4	8	33
A6QNM9	SLC25A12	SLC25A12 protein	74.529	45.9	19	39	27.4	13	32
F1MX88	SLC25A13	Uncharacterized protein	75.041	43.2	20	61	29.3	15	67
Q2KHW4	SLC25A15	Solute carrier family 25 (Mitochondrial carrier; ornithine	32.746	70.4	16	130	24.3	8	59
Q2KJJ1	SLC25A17	Solute carrier family 25 (Mitochondrial carrier; peroxisomal membrane protein, 34kDa), member 17	34.553	10.4	3	6	#N/A	#N/A	#N/A
Q29RM1	SLC25A19	Mitochondrial thiamine pyrophosphate carrier	35.111	#N/A	#N/A	#N/A	14.2	5	6
Q3SZA4	SLC25A20	Solute carrier family 25 (Carnitine/acylcarnitine translocase), member 20	32.927	54.5	14	39	25.2	9	37
Q08DK4	SLC25A22	Mitochondrial glutamate carrier 1	34.531	54	12	34	38.2	10	51
A5PJZ1	SLC25A24	Calcium-binding mitochondrial carrier protein SCaMC-1	53.284	63.7	30	112	38.2	23	105
Q0V7M4	SLC25A25	Calcium-binding mitochondrial carrier protein SCaMC-2	52.769	#N/A	#N/A	#N/A	4.5	2	2
E1BME0	SLC25A27	Uncharacterized protein	35.782	6.2	2	2	4.6	2	4
F1N2U7	SLC25A30	Uncharacterized protein	32.425	6.5	2	3	7.9	3	7
Q2YDD9	SLC25A31	ADP/ATP translocase 4	35.694	19.8	4	4	15.8	4	6
G3X7X2	SLC25A32	Uncharacterized protein	35.618	#N/A	#N/A	#N/A	2.5	1	1
Q58DS3	SLC25A35	Solute carrier family 25 member 35	27.109	4.4	1	1	#N/A	#N/A	#N/A
F1MU04	SLC25A36	Uncharacterized protein	34.251	6.8	2	4	6.8	2	3
P02722	SLC25A4	ADP/ATP translocase 1	32.967	51.7	5	13	40.9	5	18
G5E5Z7	SLC25A40	Uncharacterized protein (Fragment)	28.281	7.2	2	3	#N/A	#N/A	#N/A
E1B8B9	SLC25A42	Uncharacterized protein	35.423	2.8	1	1	#N/A	#N/A	#N/A
G3N0T6	SLC25A43	Uncharacterized protein	37.602	2.3	1	2	2.3	1	3
Q8SQH5	SLC25A5	ADP/ATP translocase 2	32.955	52.3	2	44	40.9	2	31

				Biological Replicate 1			Biological Replicate 2		
Accession number	Gene symbol	Discription	MW(kDa)	Sequence coverage(%)	Unique peptides	MS/MS count	Sequence coverage(%)	Unique peptides	MS/MS count
F1MBR0	SLC25A53	Uncharacterized protein	34.621	#N/A	#N/A	#N/A	2.9	1	1
P32007	SLC25A6	ADP/ATP translocase 3	32.877	60.4	6	138	40.9	5	101
Q58DD2	SLC26A11	Sodium-independent sulfate anion transporter	65.349	2.5	1	2	#N/A	#N/A	#N/A
E1BFJ3	SLC26A6	Uncharacterized protein	83.345	#N/A	#N/A	#N/A	2.2	2	2
Q0VCQ2	SLC27A4	Solute carrier family 27 (Fatty acid transporter), member 4	72.198	6.8	4	4	3.3	2	3
Q3ZC83	SLC29A1	Solute carrier family 29 (Nucleoside transporters), member 1	49.881	6.4	2	4	2.2	1	1
P58352	SLC2A3	Solute carrier family 2, facilitated glucose transporter	54.019	3	2	3	#N/A	#N/A	#N/A
P58354	SLC2A8	Solute carrier family 2, facilitated glucose transporter	51.416	4	2	2	#N/A	#N/A	#N/A
F1MDV2	SLC30A9	Uncharacterized protein	63.164	6.7	3	3	1.9	1	1
Q58DA6	SLC35A2	UDP-galactose translocator	41.18	2.3	1	1	#N/A	#N/A	#N/A
Q3ZBB0	SLC35B2	Solute carrier family 35, member B2	47.69	6.9	3	4	4.4	2	4
E1BEH8	SLC35B4	Uncharacterized protein	37.358	3	1	1	#N/A	#N/A	#N/A
F1ML50	SLC35E1	Uncharacterized protein	44.604	2.2	1	1	#N/A	#N/A	#N/A
E1B9A0	SLC36A1	Uncharacterized protein	52.953	3.2	1	2	#N/A	#N/A	#N/A
A7E3U5	SLC38A7	Putative sodium-coupled neutral amino acid transporter 7	49.852	1.9	1	2	3	1	1
A5D7L5	SLC39A14	Zinc transporter ZIP14	53.933	2.4	1	2	2.4	1	1
Q0VCM6	SLC43A2	Large neutral amino acids transporter small subunit 4	62.655	#N/A	#N/A	#N/A	1.8	1	1
E1BIP2	SLC44A1	Uncharacterized protein	73.87	2	1	2	#N/A	#N/A	#N/A
A5D7H3	SLC44A2	Choline transporter-like protein 2	80.026	4.4	3	5	2.1	2	4
F1MR50	SLC4A1AP	Uncharacterized protein (Fragment)	83.194	9.4	6	6	7.8	6	8
F1MDA3	SLC7A6	Uncharacterized protein	56.848	4.3	2	5	#N/A	#N/A	#N/A
F1MSI3	SLC8B1	Uncharacterized protein	63.993	3.6	2	6	#N/A	#N/A	#N/A
Q3SZK8	SLC9A3R1	Na(+)/H(+) exchange regulatory cofactor NHE-RF1	39.603	16	6	11	11.4	4	9
A7MB44	SLC9A6	Sodium/hydrogen exchanger	77.87	2.6	2	2	#N/A	#N/A	#N/A

				Biological Replicate 1			Biological Replicate 2		
Accession number	Gene symbol	Discription	MW(kDa)	Sequence coverage(%)	Unique peptides	MS/MS count	Sequence coverage(%)	Unique peptides	MS/MS count
Q32P59	SLIRP	SRA stem-loop-interacting RNA-binding protein, mitochondrial	12.493	9.9	1	3	#N/A	#N/A	#N/A
E1BMC6	SLMAP	Uncharacterized protein (Fragment)	89.544	3.5	3	3	#N/A	#N/A	#N/A
Q08DE0	SMAD2	Mothers against decapentaplegic	48.955	4.8	2	3	#N/A	#N/A	#N/A
F1N052	SMARCA5	Uncharacterized protein	121.96	4.9	5	5	#N/A	#N/A	#N/A
E1B7X9	SMARCAD1	SWI/SNF-related matrix-associated actin-dependent regulator of chromatin subfamily A containing	117.57	2.4	2	2	#N/A	#N/A	#N/A
F1MY41	SMC2	Structural maintenance of chromosomes protein	135.64	15.7	19	30	12.3	16	24
E1BMZ9	SMC4	Structural maintenance of chromosomes protein	147.27	11.2	15	21	10	14	24
A4IFN9	SMG5	SMG5 protein	113.76	1.9	1	2	#N/A	#N/A	#N/A
Q2YDD2	SMG9	Protein SMG9	57.637	18.5	7	9	2.9	1	2
F1N588	SMPD2	Uncharacterized protein	47.655	8	3	5	#N/A	#N/A	#N/A
Q2TBS9	SMU1	WD40 repeat-containing protein SMU1	57.543	1.8	1	1	1.8	1	1
Q0P585	SMYD2	N-lysine methyltransferase SMYD2	49.86	12.2	5	7	#N/A	#N/A	#N/A
E1BEP0	SMYD4	Uncharacterized protein	89.28	5.2	4	4	#N/A	#N/A	#N/A
F1MGV1	SMYD5	Uncharacterized protein	47.254	4.5	2	2	#N/A	#N/A	#N/A
Q0II86	SNAP29	Synaptosomal-associated protein 29	28.489	3.9	1	1	#N/A	#N/A	#N/A
F1MGT4	SNAP91	Uncharacterized protein (Fragment)	89.568	13.3	8	16	4.6	4	4
A4FUG0	SNAPIN	SNAPIN protein	14.85	19.1	2	3	#N/A	#N/A	#N/A
Q3T0G8	SNCA	Alpha-synuclein	14.508	31.4	4	4	22.1	3	4
Q863B3	SND1	Staphylococcal nuclease domain-containing protein 1	101.99	34.2	26	52	15.1	13	22
Q08DR7	SNF8	SNF8, ESCRT-II complex subunit, homolog (S. cerevisiae)	28.836	8.1	2	2	#N/A	#N/A	#N/A
Q1RMR2	SNRNP70	U1 small nuclear ribonucleoprotein	51.425	3.9	2	2	#N/A	#N/A	#N/A
Q2KIR1	SNRPA	U1 small nuclear ribonucleoprotein	31.263	18.1	5	7	8.2	1	7
A6H788	SNRPA1	SNRPA1 protein	28.369	27.5	6	10	7.5	2	3
Q32PA0	SNRPC	U1 small nuclear ribonucleoprotein	17.394	#N/A	#N/A	#N/A	5.7	1	2

				Biological Replicate 1			Biological Replicate 2		
Accession number	Gene symbol	Discription	MW(kDa)	Sequence coverage(%)	Unique peptides	MS/MS count	Sequence coverage(%)	Unique peptides	MS/MS count
G3X6Z3	SNRPD2	Small nuclear ribonucleoprotein Sm D2 (Fragment)	13.396	8.5	1	1	8.5	1	2
F1MZ00	SNRPD3	Small nuclear ribonucleoprotein D3 polypeptide	13.916	7.1	1	2	7.9	1	1
F1ME11	SNRPE	Small nuclear ribonucleoprotein E (Fragment)	10.708	12.1	1	2	#N/A	#N/A	#N/A
A7YWQ4	SNTB2	SNTB2 protein	57.956	1.7	1	1	#N/A	#N/A	#N/A
F1MYH6	SNX1	Sorting nexin-1	54.637	31.6	13	24	11.6	4	11
Q08DD7	SNX11	Sorting nexin-11	30.205	15.9	4	5	3	1	2
A6QR61	SNX12	SNX12 protein	18.884	67.3	8	29	27.2	4	14
Q5EA77	SNX17	Sorting nexin-17	52.825	3	2	2	#N/A	#N/A	#N/A
Q17QS1	SNX24	Sorting nexin-24	19.908	6.5	1	3	#N/A	#N/A	#N/A
A5PKA5	SNX27	Sorting nexin-27	61.126	8.1	5	6	2.8	2	4
Q1RMH8	SNX3	Sorting nexin-3	18.762	43.8	7	7	26.5	3	6
E1BMD8	SNX30	Uncharacterized protein (Fragment)	44.974	19.3	9	9	3.6	2	5
A1A4L0	SNX4	Sorting nexin-4	52.194	25.3	11	18	13.8	7	16
Q3ZBM5	SNX5	Sorting nexin-5	46.819	23	8	11	13.9	5	8
F1MHJ5	SNX6	Uncharacterized protein (Fragment)	44.639	17	7	8	2.1	1	2
Q2KHV6	SNX8	Sorting nexin-8	51.619	17.6	6	9	2.8	1	1
F1N6W6	SNX9	Sorting nexin (Fragment)	66.114	15	8	17	3.4	2	3
Q3T0N9	SOAT1	O-acyltransferase	64.831	8.5	5	6	1.6	1	1
P00442	SOD1	Superoxide dismutase [Cu-Zn]	15.682	32.2	4	9	23	3	14
P41976	SOD2	Superoxide dismutase [Mn],	24.638	26.1	5	55	19.8	4	41
Q0VCF1	SORBS1	Sorbin and SH3 domain containing 1	46.461	5.8	2	2	2.1	1	4
Q58D31	SORD	Sorbitol dehydrogenase	38.099	14.6	4	5	5.1	2	5
E1BKZ9	SORT1	Uncharacterized protein	91.816	1.1	1	1	#N/A	#N/A	#N/A
E1BL97	SOX13	Uncharacterized protein	69.008	5.8	3	5	5.3	3	8
A4FUB3	SOX5	SOX5 protein	80.126	1.5	1	1	1.5	1	2
F1MZ69	SPAG9	Uncharacterized protein	128.57	2.5	2	3	0.9	1	1
A2VDN5	SPAST	Spastin	67.224	13.5	7	10	5.5	4	5
E1BLR1	SPATA2	Uncharacterized protein	57.382	3.7	1	1	#N/A	#N/A	#N/A
F1MFP1	SPATA5	Uncharacterized protein (Fragment)	83.328	3.9	2	2	#N/A	#N/A	#N/A

				Biological Replicate 1			Biological Replicate 2		
Accession number	Gene symbol	Discription	MW(kDa)	Sequence coverage(%)	Unique peptides	MS/MS count	Sequence coverage(%)	Unique peptides	MS/MS count
A7YSY2	SPATA5L1	Spermatogenesis-associated protein 5-like protein 1	82.553	2.1	1	1	#N/A	#N/A	#N/A
F1MK70	SPATS2	Uncharacterized protein	59.285	#N/A	#N/A	#N/A	4.9	2	2
Q24JY3	SPC24	Kinetochore protein Spc24	22.755	12.7	2	3	14.7	3	5
Q3ZBK3	SPC25	Kinetochore protein Spc25	26.607	11.1	3	4	13.3	3	4
Q3T134	SPCS1	Signal peptidase complex subunit 1	11.805	11.8	1	1	#N/A	#N/A	#N/A
E1BEI2	SPCS2	Uncharacterized protein	25.348	29.6	7	10	18.7	6	11
Q3SZU5	SPCS3	Signal peptidase complex subunit 3	20.313	15.6	3	6	15.6	3	9
Q08DR9	SPDL1	Protein Spindly	69.62	11.6	6	8	#N/A	#N/A	#N/A
E1BJ04	SPECC1L	Uncharacterized protein	124.03	1.4	1	1	#N/A	#N/A	#N/A
G3N022	SPEG	Uncharacterized protein	355.05	#N/A	#N/A	#N/A	0.3	1	1
Q8MJJ1	SPG21	Maspardin	35.006	33.1	9	11	16.2	5	10
A7E2Z6	SPG7	SPG7 protein	86.193	9	6	8	7.7	6	9
Q2KJ54	SPINT2	Serine peptidase inhibitor, Kunitz	28.002	4	1	1	5.6	1	2
Q08DX7	SPNS1	Protein spinster homolog 1	56.519	15.2	5	6	#N/A	#N/A	#N/A
Q27967	SPP2	Secreted phosphoprotein 24	23.134	16.3	3	4	10.3	2	3
F1MG08	SPPL2A	Uncharacterized protein (Fragment)	57.074	1.8	1	1	#N/A	#N/A	#N/A
E1BKF8	SPPL2B	Uncharacterized protein	63.707	4.8	3	4	5	4	6
Q17QK8	SPR	Sepiapterin reductase	28.939	44.2	9	19	19.5	5	10
Q0P5G9	SPRYD4	SPRY domain containing 4	22.985	9.7	2	2	#N/A	#N/A	#N/A
Q2T9X3	SPRYD7	SPRY domain-containing protein 7	21.637	5.1	1	1	13.8	2	4
E1BMN5	SPTBN2	Uncharacterized protein	271.99	0.3	1	1	#N/A	#N/A	#N/A
Q3MHG1	SPTLC1	Serine palmitoyltransferase 1	52.787	#N/A	#N/A	#N/A	3.4	2	2
A5D9A8	SQLE	Squalene epoxidase	63.56	19.4	8	12	3	2	2
F1MKF8	SQRDL	Uncharacterized protein	39.163	26.2	9	16	17.2	7	25
F1MIE2	SQSTM1	Uncharacterized protein	47.897	16.4	5	6	11.6	3	6
Q2HJF6	SRA1	Steroid receptor RNA activator 1	24.943	6.6	1	3	#N/A	#N/A	#N/A
Q0IIA3	SRI	Sorcin	20.331	53	9	16	32.2	6	19
E1BM12	SRM	Uncharacterized protein	33.904	17.9	4	4	#N/A	#N/A	#N/A
Q2T9U1	SRP54	Signal recognition particle 54 kDa	55.704	2.4	1	1	7.1	4	6
A6QQW3	SRP68	Signal recognition particle subunit	70.507	7.2	4	5	4.2	3	3
E1BB38	SRP72	Signal recognition particle subunit	74.727	1.6	1	2	2.2	2	2

				Biological Replicate 1			Biological Replicate 2		
Accession number	Gene symbol	Discription	MW(kDa)	Sequence coverage(%)	Unique peptides	MS/MS count	Sequence coverage(%)	Unique peptides	MS/MS count
E1BNV4	SRPK1	Uncharacterized protein	72.504	4.1	2	2	#N/A	#N/A	#N/A
F1MLG1	SRPRB	Uncharacterized protein	29.716	38.7	9	9	37.6	8	18
F1MQX1	SRPX	Uncharacterized protein	50.944	7.4	3	11	#N/A	#N/A	#N/A
A0JNI4	SRR	Serine racemase	36.18	3.6	1	1	#N/A	#N/A	#N/A
F1MIT4	SRRT	Serrate RNA effector molecule	100.62	1.6	2	3	#N/A	#N/A	#N/A
Q0VCY7	SRSF1	Serine/arginine-rich splicing	27.744	18.1	3	5	#N/A	#N/A	#N/A
Q3MHR5	SRSF2	Serine/arginine-rich splicing	25.476	17.2	4	4	#N/A	#N/A	#N/A
Q3SZR8	SRSF3	Serine/arginine-rich splicing	19.329	18.3	2	3	17.7	3	3
Q3B7L6	SRSF6	Serine/arginine-rich splicing	39.646	2.6	1	1	#N/A	#N/A	#N/A
Q3T106	SRSF7	Serine/arginine-rich splicing	26.941	17.9	3	7	#N/A	#N/A	#N/A
P10881	SSB	Lupus La protein homolog	46.533	37.1	14	24	4.7	3	4
E1BIZ2	SSH1	Uncharacterized protein	111.12	2.2	1	2	#N/A	#N/A	#N/A
F1MI92	SSH3	Uncharacterized protein	71.187	25	14	37	12.8	8	15
A6QLP7	SSR1	Translocon-associated protein subunit alpha	32.054	11.9	3	6	6.6	2	3
Q3SZ87	SSR3	Translocon-associated protein subunit gamma	21.074	7.6	1	4	#N/A	#N/A	#N/A
Q2TBX5	SSR4	Translocon-associated protein subunit delta	18.814	19.8	3	5	24.4	3	3
F6QYV9	SSRP1	Uncharacterized protein	81.071	13.7	8	9	3.8	3	6
F1MVW1	SSSCA1	Uncharacterized protein	21.554	21.1	3	7	11.1	3	3
Q17QI2	SSU72	RNA polymerase II subunit A C-terminal domain phosphatase SSU72	22.588	5.7	1	1	#N/A	#N/A	#N/A
A7E3S8	ST13	Heat shock 70kD protein binding	41.444	28.2	7	19	18.4	5	21
Q17QR2	STAMBP	STAM binding protein	48.067	7.3	3	3	9.5	4	6
F1MXU5	STARD10	Uncharacterized protein	33.005	24.1	6	8	6.9	2	6
B0JYL6	STAT1	Signal transducer and activator of transcription	86.929	18.3	11	16	#N/A	#N/A	#N/A
Q9TUM3	STAT5B	Signal transducer and activator of transcription 5B	89.99	1.1	1	1	#N/A	#N/A	#N/A
F1MH34	STAU1	Uncharacterized protein (Fragment)	76.89	15.6	11	18	13.9	9	17
F1MYP9	STEAP3	Uncharacterized protein (Fragment)	54.45	3.1	1	1	#N/A	#N/A	#N/A

				Biological Replicate 1			Biological Replicate 2		
Accession number	Gene symbol	Discription	MW(kDa)	Sequence coverage(%)	Unique peptides	MS/MS count	Sequence coverage(%)	Unique peptides	MS/MS count
Q3ZBZ8	STIP1	Stress-induced-phosphoprotein 1	62.481	60	43	124	47.1	32	107
F1ML95	STK11IP	Uncharacterized protein (Fragment)	118.25	8.5	6	8	4.3	4	7
Q2KI50	STK16	Serine/threonine kinase 16	34.788	10.5	3	4	7.2	2	2
E1BBF0	STK31	Uncharacterized protein	115.22	13.6	11	14	3.7	4	5
F1MUU2	STK35	Uncharacterized protein	44.646	23.1	9	11	12.9	6	8
F1MBL1	STK38	Serine/threonine-protein kinase 38	54.168	3.7	1	1	#N/A	#N/A	#N/A
Q32LA8	STK39	Serine threonine kinase 39 (STE20/SPS1 homolog, yeast)	24.892	28.7	3	3	#N/A	#N/A	#N/A
Q3T0C7	STMN1	Stathmin	17.302	30.9	3	11	25.5	2	11
Q3MHJ8	STMN2	Stathmin	20.828	15.1	1	2	15.1	1	4
A5PJA6	STOML2	Stomatin (EPB72)-like 2	38.733	66	18	36	23.6	9	35
Q0V8E7	STRA6	Stimulated by retinoic acid gene 6 protein homolog	73.761	15.6	8	19	12	6	11
Q5E959	STRAP	Serine-threonine kinase receptor-associated protein	38.485	50.9	14	26	16.3	5	10
Q0P5J8	STRIP1	Striatin-interacting protein 1	95.686	11.8	8	10	3.9	3	7
E1BFB3	STRN	Uncharacterized protein	86.209	17.7	9	12	7.4	6	10
Q2KJI2	STT3A	Dolichyl-diphosphooligosaccharide--protein glycosyltransferase subunit	80.655	15.2	10	24	16.9	12	39
A5D7G6	STT3B	STT3B protein	93.216	7.8	5	8	8.2	6	8
F1MUH4	STUB1	Uncharacterized protein	34.833	68.5	20	59	40.7	14	35
E1BQ02	STX10	Uncharacterized protein	28.316	4.4	1	3	#N/A	#N/A	#N/A
A7MAZ2	STX12	STX12 protein	31.355	46.7	10	21	26.3	6	15
E1BIQ3	STX16	Uncharacterized protein	37.277	21.2	7	12	2.5	1	2
Q5E9Y2	STX17	Syntaxin-17	33.559	24.2	6	20	7.6	2	5
F1N1W6	STX18	Uncharacterized protein	38.673	2.4	1	2	10.1	4	5
F1MDL3	STX3	Uncharacterized protein (Fragment)	31.997	26.9	9	14	14.3	4	5
Q3SWZ3	STX4	Syntaxin-4	34.4	7.4	2	5	3.7	1	1
Q08DB5	STX5	Syntaxin-5	39.546	14.4	4	8	12.1	5	6
A2VE18	STX6	STX6 protein	29.073	17.3	5	8	4.3	1	5
Q3ZBT5	STX7	Syntaxin-7	29.656	19.2	5	9	6.5	2	4
Q3T075	STX8	Syntaxin-8	26.759	9.7	2	3	6.4	1	1

				Biological Replicate 1			Biological Replicate 2		
Accession number	Gene symbol	Discription	MW(kDa)	Sequence coverage(%)	Unique peptides	MS/MS count	Sequence coverage(%)	Unique peptides	MS/MS count
P61763	STXBP1	Syntaxin-binding protein 1	67.568	20.4	9	15	9.9	6	9
F1MHC2	STXBP2	Uncharacterized protein	66.356	40	20	36	16.9	10	17
F1MXB4	STXBP3	Uncharacterized protein	67.624	6.6	4	4	7.1	4	7
E1BNK4	STXBP5	Uncharacterized protein	127.54	2.3	2	2	#N/A	#N/A	#N/A
Q2YDL1	STXBP6	Syntaxin-binding protein 6	23.667	8.6	2	2	4.3	1	1
A7YWC6	SUB1	SUB1 protein	14.369	18.9	2	4	#N/A	#N/A	#N/A
Q58DR8	SUCLG1	Succinyl-CoA ligase [ADP/GDP-forming] subunit alpha,	36.166	21.7	7	22	22.5	7	24
Q3MHX5	SUCLG2	Succinyl-CoA ligase [GDP-forming] subunit beta, mitochondrial	46.69	47.7	18	33	18.1	12	28
E1BE55	SUGP2	Uncharacterized protein	121.57	1	1	1	1.3	1	1
Q2KIK0	SUGT1	Suppressor of G2 allele of SKP1	38.074	74.3	24	95	49.1	14	65
Q17QV7	SULT4A1	Sulfotransferase family 4A, member	33.081	9.5	3	3	#N/A	#N/A	#N/A
Q0P5L5	SUMF1	Sulfatase-modifying factor 1	40.342	4	1	1	4	1	1
Q58CP2	SUMF2	Sulfatase-modifying factor 2	33.829	19.3	4	7	#N/A	#N/A	#N/A
P61955	SUMO2	Small ubiquitin-related modifier 2	10.871	23.2	2	4	23.2	2	7
F6RUR3	SUOX	Uncharacterized protein	60.482	6.4	3	3	4.6	3	5
E1BNP8	SUPT16H	Uncharacterized protein	119.93	8.1	8	12	4.1	5	6
A6QP28	SUPV3L1	SUPV3L1 protein	58.835	#N/A	#N/A	#N/A	6	3	4
F1N6P2	SURF1	Uncharacterized protein	82.121	12.1	8	30	7.9	6	11
F1MSE7	SUSD2	Uncharacterized protein	90.052	#N/A	#N/A	#N/A	2.7	3	3
Q3T161	SYAP1	Synapse associated protein 1, SAP47 homolog (Drosophila)	40.787	27.1	8	12	13.7	3	5
E1BJF6	SYMPK	Uncharacterized protein	140.97	#N/A	#N/A	#N/A	1.3	2	2
F1MCT8	SYNCRIP	Uncharacterized protein (Fragment)	71.174	21.9	10	21	8.8	7	14
A7E3W5	SYNGR2	Synaptogyrin-2	25.139	#N/A	#N/A	#N/A	3.6	1	2
E1BD68	SYNJ1	Synaptojanin-1	134.83	4.1	4	4	1.2	1	2
Q3T0C9	SYNJ2BP	Synaptojanin-2-binding protein	15.81	33.8	3	4	29.7	4	11
F1MI41	SYNRG	Uncharacterized protein	147.41	4	6	9	1.3	2	2
A8PVV5	SYPL1	SYPL1 protein	19.476	10.3	2	2	#N/A	#N/A	#N/A
Q1RMQ3	SYS1	Protein SYS1 homolog	17.444	5.8	1	1	#N/A	#N/A	#N/A
F1MY23	SYTL2	Synaptotagmin-like protein 2	105.79	4.7	4	6	0.8	1	2

				Biological Replicate 1			Biological Replicate 2		
Accession number	Gene symbol	Discription	MW(kDa)	Sequence coverage(%)	Unique peptides	MS/MS count	Sequence coverage(%)	Unique peptides	MS/MS count
E1BPE2	SYTL4	Uncharacterized protein	76.202	1.3	1	1	#N/A	#N/A	#N/A
E1BAK9	SYVN1	Uncharacterized protein	67.098	18	10	20	5.7	4	10
F1MSQ8	TACC1	Uncharacterized protein (Fragment)	78.462	#N/A	#N/A	#N/A	2.2	2	3
A6QL93	TACC3	TACC3 protein	86.367	32.6	28	69	24.4	24	72
E1BJK7	TACO1	Uncharacterized protein	32.244	#N/A	#N/A	#N/A	4	1	1
F1MSN2	TACSTD2	Uncharacterized protein	35.441	7.8	2	3	#N/A	#N/A	#N/A
Q3SZP8	TADA2A	Transcriptional adapter 2-alpha	51.468	3.6	1	1	#N/A	#N/A	#N/A
E1BGX1	TAF15	Uncharacterized protein	50.926	4.9	1	1	3.2	2	4
F1MDZ8	TAF6L	Uncharacterized protein	72.727	1.7	1	2	#N/A	#N/A	#N/A
Q9TS87	TAGLN	Transgelin	22.599	7	1	1	11.4	2	3
Q5E9F5	TAGLN2	Transgelin-2	22.426	29.6	4	5	#N/A	#N/A	#N/A
Q3ZBY2	TAGLN3	Transgelin-3	22.484	4	1	1	#N/A	#N/A	#N/A
G5E5C8	TALDO1	Transaldolase	37.681	50.4	20	56	34.7	14	51
Q32L81	TAMM41	Mitochondrial translocator assembly and maintenance protein 41 homolog	37.42	6	1	1	4.8	2	2
Q29RZ5	TANGO2	Transport and Golgi organization protein 2 homolog	30.829	11.6	2	2	6.2	2	2
Q3ZBV8	TARS	Threonine--tRNA ligase, cytoplasmic	83.491	36.4	24	53	20.6	16	23
A6QNM8	TARSL2	Probable threonine--tRNA ligase 2, cytoplasmic	83.075	2.8	2	2	#N/A	#N/A	#N/A
Q148G4	TATDN1	Putative deoxyribonuclease TATDN1	33.886	14.8	5	8	9.8	3	3
Q2KJE0	TAX1BP1	Tax1-binding protein 1 homolog	94.058	13.6	11	12	9.2	9	12
O97790	TBC1D1	TBC1 domain family member 1	132.1	1	1	1	#N/A	#N/A	#N/A
F1MB46	TBC1D10A	Uncharacterized protein (Fragment)	49.716	14	7	9	4.6	2	4
F1MJZ7	TBC1D10B	Uncharacterized protein	87.074	1.9	1	1	2	2	2
F1MXD4	TBC1D13	Uncharacterized protein	46.475	15.3	5	7	#N/A	#N/A	#N/A
F1N090	TBC1D15	Uncharacterized protein (Fragment)	77.578	31.7	20	36	14.2	9	18
F1MU30	TBC1D23	Uncharacterized protein	78.471	4	2	2	#N/A	#N/A	#N/A
Q29RJ2	TBC1D24	TBC1 domain family member 24	58.425	13.2	7	8	2.1	1	2
Q5E9C4	TBC1D7	TBC1 domain family member 7	33.904	14.7	4	6	#N/A	#N/A	#N/A
F1MS54	TBC1D9B	Uncharacterized protein	141.22	4	4	5	#N/A	#N/A	#N/A
P48427	TBCA	Tubulin-specific chaperone A	12.707	23.1	4	6	24.1	3	7

				Biological Replicate 1			Biological Replicate 2		
**Accession number**	**Gene symbol**	**Discription**	**MW(kDa)**	**Sequence coverage(%)**	**Unique peptides**	**MS/MS count**	**Sequence coverage(%)**	**Unique peptides**	**MS/MS count**
Q5E951	TBCB	Tubulin-folding cofactor B	27.517	36.5	8	17	23.4	6	11
Q3SZE9	TBCC	Tubulin-specific chaperone C	38.902	21.7	6	12	#N/A	#N/A	#N/A
A7MB50	TBCD	TBCD protein	129.96	34.1	36	80	12.4	16	32
Q32KS0	TBCE	Tubulin-specific chaperone E	59.326	23.5	13	26	18	11	23
F1MCE2	TBCEL	Uncharacterized protein	48.114	26.2	10	12	#N/A	#N/A	#N/A
F1MSR5	TBCK	Uncharacterized protein	100.79	1.8	2	2	#N/A	#N/A	#N/A
Q0P5H6	TBL2	Transducin (Beta)-like 2	49.911	35.4	14	22	26.9	11	29
F1MP97	TBRG4	Protein TBRG4 (Fragment)	62.362	5.5	3	4	#N/A	#N/A	#N/A
Q29RL9	TCEA1	Transcription elongation factor A protein 1	33.89	24.3	8	9	25.2	6	13
Q2KII4	TCEB1	Transcription elongation factor B polypeptide 1	12.473	45.5	4	11	27.7	3	9
Q3SZ32	TCEB2	Elongin B	13.17	60.2	7	11	39	6	15
E1BB92	TCL1A	Uncharacterized protein	13.351	33	2	4	7	1	4
G3N1Y7	TCL1B	Uncharacterized protein (Fragment)	12.439	25.7	4	6	#N/A	#N/A	#N/A
Q9XSC9	TCN2	Transcobalamin-2	47.958	22.7	10	16	9	4	8
E1BHU1	TCOF1	Uncharacterized protein	151.29	1.3	2	2	#N/A	#N/A	#N/A
G5E531	TCP1	T-complex protein 1 subunit alpha	60.222	51.6	25	87	29.9	16	79
F1MQ24	TCP11L1	Uncharacterized protein	56.617	12.6	5	9	4.5	2	3
F1MST1	TDP1	Uncharacterized protein	68.381	#N/A	#N/A	#N/A	2.6	2	2
A7YWI9	TDP2	Tyrosyl-DNA phosphodiesterase 2	40.826	5.5	1	1	#N/A	#N/A	#N/A
A6QLE1	TDRD7	Tudor domain-containing protein 7	123.9	10.4	12	14	5.6	8	16
F1MUI1	TDRKH	Uncharacterized protein	62.649	47.7	24	77	22.3	12	63
Q3ZCD7	TECR	Very-long-chain enoyl-CoA reductase	36.072	24.7	10	28	14.3	5	13
Q0VCT3	TERF2IP	Telomeric repeat-binding factor 2-interacting protein 1	44.221	3.5	1	1	#N/A	#N/A	#N/A
Q2YDE9	TES	Testin	48.049	34.9	12	21	22.8	8	16
F1MDB6	TESC	Uncharacterized protein (Fragment)	19.894	25.1	4	6	9.9	2	3
F1MND3	TEX10	Uncharacterized protein	105.78	1.7	2	2	3.8	4	5
F1MJA6	TEX2	Uncharacterized protein	116.29	2.4	3	4	4.2	5	7
Q32L30	TEX264	Testis expressed 264	33.812	6.4	1	1	3.2	1	3
Q3ZC52	TEX30	Testis-expressed sequence 30	25.181	12.5	2	2	#N/A	#N/A	#N/A
Q0II87	TFAM	Transcription factor A,	28.906	16.7	4	4	13	4	6

				Biological Replicate 1			Biological Replicate 2		
Accession number	Gene symbol	Discription	MW(kDa)	Sequence coverage(%)	Unique peptides	MS/MS count	Sequence coverage(%)	Unique peptides	MS/MS count
Q2TBQ0	TFB1M	Mitochondrial dimethyladenosine transferase 1	38.883	2.6	1	1	#N/A	#N/A	#N/A
F6PW02	TFG	Uncharacterized protein	43.204	36.2	11	42	20.2	6	16
E1BIG6	TFRC	Uncharacterized protein	83.166	4.5	4	4	7	6	10
F1MBS3	TGFBI	Transforming growth factor-beta-induced protein ig-h3 (Fragment)	72.251	32.7	18	43	14.5	9	17
A6QQ20	TGOLN2	TGOLN2 protein (Fragment)	33.444	6.8	4	7	8.7	3	4
Q3SWW8	THBS4	Thrombospondin-4	105.97	4	3	3	3.4	3	6
A6QQ83	THEM2	THEM2 protein	16.857	8.4	1	5	14.8	2	7
A1A4L1	THEM4	Acyl-coenzyme A thioesterase THEM4	26.812	16.9	4	6	#N/A	#N/A	#N/A
A6H707	THEM6	Protein THEM6	23.749	66.3	16	31	42.8	12	30
Q05B50	THG1L	Probable tRNA(His)	34.739	10.1	3	3	3.7	1	1
Q29RH4	THOC3	THO complex subunit 3	38.872	4.8	2	2	8	3	4
Q1JPJ8	THOP1	Thimet oligopeptidase	78.138	59.8	41	127	30.9	25	99
Q8MKF1	THTPA	Thiamine-triphosphatase	23.983	17.8	4	6	8.7	2	2
Q24K03	THUMPD1	THUMP domain-containing protein 1	39.753	14.3	5	9	6.7	3	6
Q2T9W2	THUMPD3	THUMP domain-containing protein 3	56.674	2.2	1	1	4.3	2	2
F2Z4H8	TIAL1	Uncharacterized protein	42.253	12.6	4	5	7.4	3	6
Q1JQA7	TIGAR	Fructose-2,6-bisphosphatase TIGAR	29.466	31.9	7	12	17.4	4	10
F6RJY8	TIMM13	Uncharacterized protein	16.172	#N/A	#N/A	#N/A	9.3	1	1
Q2HJE9	TIMM17B	Mitochondrial import inner membrane translocase subunit Tim17-B	18.321	25	4	5	#N/A	#N/A	#N/A
Q3SZV6	TIMM21	Mitochondrial import inner membrane translocase subunit Tim21	27.938	9.8	2	2	18.9	4	10
A4IFL0	TIMM23	Mitochondrial import inner membrane translocase subunit Tim23	21.827	12.9	2	2	#N/A	#N/A	#N/A
E1B844	TIMM44	Uncharacterized protein	51.445	49.1	25	71	35.9	18	60
Q3SZB3	TIMM50	Mitochondrial import inner membrane translocase subunit TIM50	40.108	27.3	8	21	13.8	6	9
A3KN17	TINF2	TINF2 protein	49.795	2.2	1	1	4	2	2
Q29RT7	TIPRL	TIP41, TOR signaling pathway regulator-like (S. cerevisiae)	28.161	31	5	9	13.2	3	9

				Biological Replicate 1			Biological Replicate 2		
Accession number	Gene symbol	Discription	MW(kDa)	Sequence coverage(%)	Unique peptides	MS/MS count	Sequence coverage(%)	Unique peptides	MS/MS count
F1N2D3	TJP1	Uncharacterized protein (Fragment)	190.4	3.5	5	5	1.9	3	5
F1MTP9	TJP2	Uncharacterized protein	92.733	1.5	1	1	#N/A	#N/A	#N/A
F1MNI9	TK2	Uncharacterized protein (Fragment)	34.814	9.8	2	3	5.9	2	2
A7E3W4	TKT	Transketolase	64.875	27.9	18	33	12.4	9	19
Q2NL26	TKTL1	Transketolase-like protein 1	64.966	3.9	2	3	1.5	1	2
F1MTK2	TLDC1	Uncharacterized protein	50.25	25.4	11	15	11.8	6	11
F1MIL2	TLE6	Uncharacterized protein (Fragment)	50.5	40.2	19	105	31.9	15	95
F1MF35	TLL2	Uncharacterized protein (Fragment)	100.69	22.2	19	37	16.1	11	32
Q8WMV1	TM7SF2	Delta(14)-sterol reductase	46.75	#N/A	#N/A	#N/A	3.1	1	1
Q08DX3	TM7SF3	Transmembrane 7 superfamily member	64.454	11.4	6	15	5.3	3	9
F1MCZ2	TM9SF1	Transmembrane 9 superfamily member	69.191	2	1	1	#N/A	#N/A	#N/A
F1N672	TM9SF2	Uncharacterized protein (Fragment)	76.269	9.8	6	15	4.4	3	10
E1BMF1	TM9SF3	Uncharacterized protein	67.528	12.4	10	26	12.1	9	36
A5D7E2	TM9SF4	Transmembrane 9 superfamily member	74.366	11.7	7	14	8.1	6	14
Q0V882	TMBIM6	Bax inhibitor 1	26.365	3.8	2	3	#N/A	#N/A	#N/A
F1N546	TMCC3	Uncharacterized protein	53.561	1.9	1	1	#N/A	#N/A	#N/A
Q3T0N3	TMCO1	Transmembrane and coiled-coil domain-containing protein 1	21.175	4.3	1	1	#N/A	#N/A	#N/A
Q2TBK5	TMED1	Transmembrane emp24 domain-containing protein 1	25.137	4	1	1	4	1	3
Q5E971	TMED10	Transmembrane emp24 domain-containing protein 10	24.827	34.7	6	23	28.8	6	35
C3V9V7	TMED2	RNP24	22.733	32.8	6	13	15.9	4	9
A2VDW4	TMED3	Transmembrane emp24 protein transport domain containing 3	24.8	37.2	9	19	27.1	6	12
F1N6C2	TMED4	Uncharacterized protein (Fragment)	27.506	17.8	4	12	15.4	5	10
Q2KJ84	TMED5	Transmembrane emp24 domain-containing protein 5	25.874	5.3	1	3	#N/A	#N/A	#N/A
A4IFT6	TMED7	TMED7 protein	25.335	14.7	3	7	14.2	3	8
E1BNI6	TMED8	Uncharacterized protein	35.87	#N/A	#N/A	#N/A	4	1	2
Q3T133	TMED9	Transmembrane emp24 domain-containing protein 9	27.298	29.4	7	21	12.8	4	19

				Biological Replicate 1			Biological Replicate 2		
Accession number	Gene symbol	Discription	MW(kDa)	Sequence coverage(%)	Unique peptides	MS/MS count	Sequence coverage(%)	Unique peptides	MS/MS count
Q5EA90	TMEM106A	Transmembrane protein 106A	28.828	12.3	2	10	12.3	2	15
Q29RH9	TMEM109	Transmembrane protein 109	26.225	6.2	1	2	3.7	1	2
A5D7N3	TMEM11	Transmembrane protein 11,	21.478	22.9	4	13	22.4	5	7
F1N070	TMEM119	Uncharacterized protein	29.562	3.8	1	3	#N/A	#N/A	#N/A
Q05B45	TMEM120A	Transmembrane protein 120A	40.73	10.5	3	4	5.5	2	4
A6QPF8	TMEM120B	Transmembrane protein 120B	40.372	10.6	2	3	5.6	2	2
Q32L86	TMEM126A	Transmembrane protein 126A	21.536	13.7	2	5	9.1	2	3
Q3SZR6	TMEM147	Transmembrane protein 147	25.321	11.6	2	4	#N/A	#N/A	#N/A
Q3ZCI1	TMEM14C	Transmembrane protein 14C	11.735	9.6	1	4	9.6	1	9
Q24JY6	TMEM160	Transmembrane protein 160	19.465	38.3	7	25	16	4	14
A6QQX9	TMEM163	Transmembrane protein 163	31.195	16.4	4	4	12.9	3	4
A0JNG0	TMEM168	Transmembrane protein 168	79.784	16.4	11	18	5.6	5	6
F1N3P0	TMEM181	Uncharacterized protein (Fragment)	70.346	1.8	1	1	1.8	1	2
A2VDL9	TMEM184B	Transmembrane protein 184B	45.489	#N/A	#N/A	#N/A	1.7	1	2
A7E3R1	TMEM19	Transmembrane protein 19	36.44	2.1	1	1	#N/A	#N/A	#N/A
E1BL89	TMEM192	Uncharacterized protein (Fragment)	31.079	8.8	2	3	#N/A	#N/A	#N/A
G5E6J0	TMEM200B	Uncharacterized protein	32.668	3.3	1	23	3.3	1	22
Q32PF0	TMEM201	Transmembrane protein 201	43.242	6.6	2	2	#N/A	#N/A	#N/A
Q32L10	TMEM205	Transmembrane protein 205	20.97	22.2	3	3	15.3	2	6
A4FV45	TMEM214	Transmembrane protein 214	77.015	#N/A	#N/A	#N/A	3.8	3	4
A7MB75	TMEM231	Transmembrane protein 231	36.003	3.8	1	1	#N/A	#N/A	#N/A
E1BD52	TMEM245	Transmembrane protein 245	96.121	#N/A	#N/A	#N/A	3.3	3	5
Q3SYV1	TMEM263	UPF0444 transmembrane protein C12orf23 homolog	11.721	8.6	1	1	8.6	1	4
Q17QL5	TMEM30A	Transmembrane protein 30A	40.683	11.6	5	10	20.8	7	14
E1BNJ0	TMEM33	Uncharacterized protein	27.913	27.2	6	19	7.7	2	6
Q5E9T5	TMEM35	Transmembrane protein 35	19.307	5.6	2	2	18.1	2	2
Q0VC58	TMEM38B	Trimeric intracellular cation channel type B	32.451	10.3	2	6	#N/A	#N/A	#N/A
Q08D99	TMEM41A	Transmembrane protein 41A	29.577	#N/A	#N/A	#N/A	4.9	1	2
A6QQR5	TMEM43	TMEM43 protein	44.791	55	19	57	20.2	10	25
Q17QI9	TMEM55B	Transmembrane protein 55B	29.437	17	4	6	11.9	3	8

				Biological Replicate 1			Biological Replicate 2		
Accession number	Gene symbol	Discription	MW(kDa)	Sequence coverage(%)	Unique peptides	MS/MS count	Sequence coverage(%)	Unique peptides	MS/MS count
F1MY22	TMEM63B	Uncharacterized protein	94.759	3.1	3	4	8.8	7	14
Q0VCH8	TMEM65	Transmembrane protein 65	25.041	30.9	7	15	28	6	20
Q3MHQ7	TMEM86A	Lysoplasmalogenase-like protein	26.426	#N/A	#N/A	#N/A	3.3	1	1
A4FV22	TMEM87A	TMEM87A protein	63.373	10.5	5	9	4.5	3	7
Q3ZCD6	TMEM9B	TMEM9 domain family, member B	22.519	10.1	3	4	#N/A	#N/A	#N/A
E1BAI7	TMOD2	Uncharacterized protein	39.528	8.8	3	4	#N/A	#N/A	#N/A
F1MSQ9	TMPO	Uncharacterized protein	42.894	15.7	5	7	10.7	3	7
Q0Z7W6	TMX1	Thioredoxin-related transmembrane protein 1	31.581	32.4	15	44	22.7	10	28
Q2TBU2	TMX2	Thioredoxin-related transmembrane protein 2	34.028	26	8	13	16.2	5	18
A6QL97	TMX3	TMX3 protein	51.729	8.1	4	7	5.7	3	4
A6H732	TMX4	TMX4 protein	38.883	3.4	1	1	3.4	1	1
A4IF78	TNFAIP8	Tumor necrosis factor alpha-induced protein 8	22.995	7.1	2	2	4	1	1
F1MBJ7	TNPO2	Uncharacterized protein (Fragment)	116.09	9.1	4	7	4.9	2	3
A5D7C4	TNPO3	TNPO3 protein	104.11	#N/A	#N/A	#N/A	1.1	1	2
Q2LGB5	TOLLIP	Toll-interacting protein	30.081	21.2	4	7	13.2	3	6
Q5BIP4	TOM1	Target of myb1	54.211	17.7	7	9	11.8	5	7
A6QPI6	TOMM22	Mitochondrial import receptor subunit TOM22 homolog	15.292	16.4	2	5	8.6	1	4
E1BGD1	TOMM34	Uncharacterized protein	34.699	57	19	47	39.2	12	35
Q1LZB5	TOMM40	Mitochondrial import receptor subunit TOM40 homolog	37.725	9.4	3	7	5.5	2	4
A6QR22	TOMM40L	Mitochondrial import receptor subunit TOM40B	33.81	19.8	6	13	12.7	3	10
Q08E34	TOMM70A	Translocase of outer mitochondrial membrane 70	67.568	31.5	18	38	25.6	17	53
A5PK75	TOR1A	TOR1A protein	37.81	3	1	1	3.3	1	2
A1A4M8	TOR3A	Torsin family 3, member A	45.717	2.2	1	1	#N/A	#N/A	#N/A
F1N6L2	TPBGL	Uncharacterized protein (Fragment)	24.808	4.9	1	1	#N/A	#N/A	#N/A
F1MBV6	TPD52	Uncharacterized protein	19.875	6.5	1	2	14.1	2	6

				Biological Replicate 1			Biological Replicate 2		
Accession number	Gene symbol	Discription	MW(kDa)	Sequence coverage(%)	Unique peptides	MS/MS count	Sequence coverage(%)	Unique peptides	MS/MS count
G3MWH1	TPD52L2	Uncharacterized protein	24.912	26.6	8	17	22.3	5	9
Q5E956	TPI1	Triosephosphate isomerase	26.689	76.3	18	60	66.3	14	56
Q5E9T4	TPK1	Thiamin pyrophosphokinase 1	27.028	7	1	1	#N/A	#N/A	#N/A
F1MV90	TPM1	Uncharacterized protein	28.984	#N/A	#N/A	#N/A	25.8	2	4
Q0V8B6	TPP1	Tripeptidyl-peptidase 1	61.351	5.7	3	8	3.7	2	3
F1N0M0	TPP2	Tripeptidyl-peptidase 2	138.34	25	26	51	17.5	22	41
Q32LJ4	TPRG1L	Tumor protein p63-regulated gene 1-like protein	25.53	7.4	2	3	8.3	2	6
E1BN75	TPRKB	Uncharacterized protein	19.551	10.3	1	1	#N/A	#N/A	#N/A
Q3SYY2	TPST2	Protein-tyrosine sulfotransferase 2	41.966	6.4	3	6	#N/A	#N/A	#N/A
Q5E984	TPT1	Translationally-controlled tumor	19.581	44.2	8	43	33.1	6	22
Q2KJ71	TRA2A	Transformer 2 alpha homolog	32.631	3.2	1	2	#N/A	#N/A	#N/A
E1B9D9	TRAF2	Uncharacterized protein (Fragment)	58.274	6.7	4	7	#N/A	#N/A	#N/A
E1BBH2	TRAF3	Uncharacterized protein	64.2	5.6	3	3	#N/A	#N/A	#N/A
Q3ZCC3	TRAF6	TNF receptor-associated factor 6	61.646	2.6	1	1	#N/A	#N/A	#N/A
Q58D05	TRAFD1	TRAF-type zinc finger domain-containing protein 1	64.846	5.2	2	4	6.4	4	5
G3N1I4	TRAK2	Uncharacterized protein (Fragment)	84.156	1.1	1	3	#N/A	#N/A	#N/A
Q9GKZ4	TRAM1	Translocating chain-associated membrane protein 1	43.201	3.7	1	1	#N/A	#N/A	#N/A
E1BCM2	TRAM2	Uncharacterized protein	43.07	#N/A	#N/A	#N/A	2.4	1	1
Q2TBI4	TRAP1	Heat shock protein 75 kDa,	79.38	53.1	36	113	31.3	24	94
Q17QI1	TRAPPC1	Trafficking protein particle complex subunit 1	16.831	43.4	6	9	6.9	1	3
A7MB76	TRAPPC13	Trafficking protein particle complex subunit 13	46.546	25.9	10	17	12.2	7	15
Q3T0F2	TRAPPC2	Trafficking protein particle complex subunit 2	16.415	23.6	3	4	#N/A	#N/A	#N/A
F1MIB4	TRAPPC2L	Trafficking protein particle complex subunit 2-like protein	16.099	25.9	3	7	22.3	3	4
F1N5V9	TRAPPC3	Uncharacterized protein	16.987	15.9	3	3	10.6	2	3

				Biological Replicate 1			Biological Replicate 2		
Accession number	Gene symbol	Discription	MW(kDa)	Sequence coverage(%)	Unique peptides	MS/MS count	Sequence coverage(%)	Unique peptides	MS/MS count
Q2TBL9	TRAPPC4	Trafficking protein particle complex subunit 4	24.264	29.2	6	12	7.8	2	5
F1MSD6	TRAPPC5	Trafficking protein particle complex subunit 5	20.767	24.5	5	5	13.3	3	10
Q3T086	TRAPPC6A	Trafficking protein particle complex subunit 6A	17.639	5.7	1	2	19.5	3	7
Q32L78	TRAPPC6B	Trafficking protein particle complex subunit 6B	17.983	17.1	3	3	16.5	3	4
F1MLG3	TRAPPC8	Uncharacterized protein (Fragment)	115.37	21.2	18	34	11.4	13	29
Q32PH0	TRAPPC9	Trafficking protein particle complex subunit 9	127	#N/A	#N/A	#N/A	0.9	1	4
F2Z4H6	TRIM23	Uncharacterized protein	64.707	1.7	1	8	#N/A	#N/A	#N/A
G3X823	TRIM26	Uncharacterized protein	62.572	6	2	2	4	2	3
E1BJG5	TRIM28	Uncharacterized protein	86.733	18.3	13	16	5.8	5	8
A7MB36	TRIM3	TRIM3 protein	80.845	6.6	4	4	1.5	1	1
Q0VCP5	TRIM32	Tripartite motif-containing 32	71.87	11.5	7	11	9.6	6	10
F1MNE1	TRIM33	Uncharacterized protein	122.55	4.4	4	4	1.7	2	4
E1BAW5	TRIM36	Uncharacterized protein	82.94	1.2	1	2	#N/A	#N/A	#N/A
E1BJS7	TRIM71	E3 ubiquitin-protein ligase TRIM71	93.191	4.8	4	5	5	4	4
E1B7Q7	TRIP12	E3 ubiquitin-protein ligase TRIP12	220.39	5.2	9	14	0.8	2	2
F1N3B9	TRIP13	Uncharacterized protein	48.649	2.8	1	1	2.8	1	1
Q2KIA2	TRMT112	tRNA methyltransferase 112 homolog	14.319	52.8	6	13	#N/A	#N/A	#N/A
Q1RML6	TRNT1	Uncharacterized protein	49.779	41.9	17	32	15.7	8	18
F1MM99	TSC1	Uncharacterized protein	128.59	7.5	7	8	6.7	8	14
E1BNT2	TSC2	Uncharacterized protein (Fragment)	201.37	2.4	4	5	4.6	8	9
E1BCC2	TSC22D2	Uncharacterized protein	77.707	#N/A	#N/A	#N/A	1.2	1	2
P43896	TSFM	Elongation factor Ts, mitochondrial	36.602	43.2	12	24	29.6	10	27
A3KN51	TSG101	TSG101 protein	44.055	32.7	12	27	18.9	8	22
Q08DM8	TSN	Translin	26.169	54.8	13	42	26.8	7	18
G3MY03	TSNAX	Uncharacterized protein	33.049	49.7	10	22	8.6	3	6
Q2KJ41	TSPAN14	Tetraspanin 14	30.723	3.7	1	2	#N/A	#N/A	#N/A
Q3SYV5	TSPAN33	Tetraspanin-33	31.574	#N/A	#N/A	#N/A	3.2	1	1

				Biological Replicate 1			Biological Replicate 2		
Accession number	Gene symbol	Discription	MW(kDa)	Sequence coverage(%)	Unique peptides	MS/MS count	Sequence coverage(%)	Unique peptides	MS/MS count
Q32KU6	TSPAN6	Tetraspanin-6	27.524	9.4	2	4	4.5	2	3
Q148J1	TSPAN7	Tetraspanin 7	27.554	9.6	2	5	9.6	2	5
Q3T090	TSR2	Pre-rRNA-processing protein TSR2	20.789	12.1	2	2	8.9	2	5
G3N094	TSSC1	Uncharacterized protein	43.217	26.1	7	11	10.1	4	9
P00586	TST	Thiosulfate sulfurtransferase	33.295	8.1	2	2	9.8	3	3
Q2KIT8	TSTA3	Tissue specific transplantation antigen P35B	36.026	36.4	13	38	16.5	5	9
F1MW03	TSTD1	Uncharacterized protein	12.562	31.6	4	10	26.3	4	9
Q3ZBR5	TTC1	Tetratricopeptide repeat protein 1	33.596	7.2	2	2	12.7	4	5
F1MZZ3	TTC15	Uncharacterized protein	85.014	24.8	15	24	13.9	11	23
F1MEM9	TTC19	Uncharacterized protein	40.417	14	4	7	9.6	4	6
E1B885	TTC37	Uncharacterized protein	174.66	7.5	10	13	3.1	6	8
F1MNU0	TTC39A	Uncharacterized protein	65.577	#N/A	#N/A	#N/A	1.6	1	1
Q5EA11	TTC4	Tetratricopeptide repeat protein 4	44.372	12.1	5	10	4.4	2	5
E1BPP1	TTK	Uncharacterized protein (Fragment)	89.274	2.5	2	2	#N/A	#N/A	#N/A
P38584	TTL	Tubulin--tyrosine ligase	43.27	2.4	1	1	#N/A	#N/A	#N/A
F1N5K2	TTLL12	Uncharacterized protein	75.492	44.1	21	55	11	6	10
F1N757	TTN	Uncharacterized protein	3813.9	0.1	2	2	0.1	4	6
O46375	TTR	Transthyretin	15.727	51	4	11	19.7	2	9
F1N1D4	TTYH3	Protein tweety homolog	57.753	2.1	1	1	#N/A	#N/A	#N/A
F2Z4C1	TUBA1A	Uncharacterized protein	50.135	63	0	5	#N/A	#N/A	#N/A
P81947	TUBA1B	Tubulin alpha-1B chain	50.151	63	1	8	38.1	1	8
Q3ZCJ7	TUBA1C	Tubulin alpha-1C chain	49.857	70.6	1	10	#N/A	#N/A	#N/A
Q2HJ86	TUBA1D	Tubulin alpha-1D chain	50.282	57.7	1	5	40	1	75
E1BJB1	TUBB2B	Tubulin beta-2B chain	49.906	70.3	6	216	36.2	2	24
Q2T9S0	TUBB3	Tubulin beta-3 chain	50.432	56.7	7	29	39.6	4	22
Q3MHM5	TUBB4B	Tubulin beta-4B chain	49.83	74.2	4	61	40	3	107
Q2KJD0	TUBB5	Tubulin beta-5 chain	49.67	70.5	4	24	36.3	2	10
A6QNT3	TUBE1	TUBE1 protein	40.753	3.3	1	1	#N/A	#N/A	#N/A
Q32KM1	TUBG2	Tubulin gamma-2 chain	51.064	39.5	16	27	11.8	6	15
A5D7P5	TUBGCP2	TUBGCP2 protein	102.75	18.3	14	28	7.6	8	15
E1BNG7	TUBGCP3	Uncharacterized protein	103.12	17.1	13	19	5.4	5	7

				Biological Replicate 1			Biological Replicate 2		
Accession number	Gene symbol	Discription	MW(kDa)	Sequence coverage(%)	Unique peptides	MS/MS count	Sequence coverage(%)	Unique peptides	MS/MS count
A0JNH5	TUBGCP4	Tubulin, gamma complex associated protein 4	75.932	9.6	5	6	5	4	5
P49410	TUFM	Elongation factor Tu, mitochondrial	49.398	59.3	23	88	29.2	12	58
F1N7S0	TULP3	Uncharacterized protein	52.905	2.9	2	3	#N/A	#N/A	#N/A
E1BD72	TUSC2	Uncharacterized protein	12.132	51.8	4	17	#N/A	#N/A	#N/A
Q29S14	TVP23B	Golgi apparatus membrane protein TVP23 homolog B	23.667	9.1	3	4	#N/A	#N/A	#N/A
Q56JV6	TWF1	Twinfilin-1	40.248	43.4	11	26	19.1	8	11
A2VDX0	TWF2	TWF2 protein	39.564	10	1	1	#N/A	#N/A	#N/A
E1BGS4	TXLNA	Uncharacterized protein	63.288	6.1	3	3	#N/A	#N/A	#N/A
G3X6A9	TXLNG	Uncharacterized protein	58.605	3.5	1	1	1.8	1	1
G8JKZ8	TXN	Thioredoxin (Fragment)	10.868	55.7	6	13	32	3	6
Q5E936	TXNDC12	Thioredoxin domain-containing	19.222	23.3	4	6	27.3	5	7
F1MHF7	TXNDC17	Uncharacterized protein	13.941	15.4	2	5	#N/A	#N/A	#N/A
F1MKS3	TXNDC5	Uncharacterized protein (Fragment)	28.673	37.4	8	20	13.6	5	12
F1MYQ1	TXNDC9	Thioredoxin domain-containing	26.488	15.5	4	4	11.1	3	5
F1MLV3	TXNL1	Uncharacterized protein	32.281	51.2	10	19	20.4	4	7
G1K1Q2	TXNRD1	Thioredoxin reductase 1,	54.563	2.4	1	1	#N/A	#N/A	#N/A
Q9N2I8	TXNRD2	Thioredoxin reductase 2,	54.669	15.5	6	13	11.4	5	9
F1MBL2	TXNRD3	Uncharacterized protein (Fragment)	62.65	8.3	5	6	4.9	3	5
Q2TA32	TYMS	Thymidylate synthase	39.785	9.6	3	3	3.1	1	1
A1A4K8	U2AF1	Splicing factor U2AF 35 kDa subunit	27.701	3.4	1	1	#N/A	#N/A	#N/A
Q24JZ8	U2AF2	U2 small nuclear RNA auxiliary	53.486	1.7	1	1	#N/A	#N/A	#N/A
G3N337	UAP1L1	Uncharacterized protein (Fragment)	56.711	14.5	6	11	4.7	2	7
A3KMV5	UBA1	Ubiquitin-like modifier-activating enzyme 1	117.83	56.4	49	167	31.7	29	112
A4FV12	UBA2	UBA2 protein	71.031	1.4	1	1	1.4	1	2
Q0P5I7	UBA3	Ubiquitin-like modifier activating enzyme 3	51.804	5.8	3	3	5.6	3	3
A7MAZ3	UBA5	Ubiquitin-like modifier-activating	44.696	24.3	6	12	2.7	1	1
P63048	UBA52	Ubiquitin-60S ribosomal protein L40	14.728	#N/A	#N/A	#N/A	36.7	5	76
F1ME38	UBA6	Uncharacterized protein	118.79	40.8	37	75	14.5	16	40
Q0VBZ2	UBAC1	UBA domain containing 1	45.088	5.2	2	2	#N/A	#N/A	#N/A
A6QR26	UBAP1	UBAP1 protein	54.99	7.4	2	2	#N/A	#N/A	#N/A

				Biological Replicate 1			Biological Replicate 2		
Accession number	Gene symbol	Discription	MW(kDa)	Sequence coverage(%)	Unique peptides	MS/MS count	Sequence coverage(%)	Unique peptides	MS/MS count
E1BA82	UBAP2	Uncharacterized protein	119.55	4.4	4	6	2.1	2	4
A6QLT5	UBAP2L	UBAP2L protein	103.95	12.7	9	16	4.2	4	6
F1MYY9	UBASH3A	Uncharacterized protein	70.154	9.1	6	9	7.7	5	6
F1MTZ3	UBASH3B	Uncharacterized protein (Fragment)	66.702	2	1	2	#N/A	#N/A	#N/A
P0CG53	UBB	Polyubiquitin-B	34.308	84.3	1	1	#N/A	#N/A	#N/A
Q32PA5	UBE2C	Ubiquitin-conjugating enzyme E2 C	19.608	30.7	4	8	#N/A	#N/A	#N/A
Q1RMX2	UBE2D2	Ubiquitin-conjugating enzyme E2 D2	16.735	44.9	1	17	32.7	1	2
Q3ZCF7	UBE2D3	Ubiquitin-conjugating enzyme E2 D3	16.745	34.7	1	3	34.7	2	18
G3N2X5	UBE2E2	Uncharacterized protein (Fragment)	14.145	6.3	1	2	15.1	2	3
Q1RMW1	UBE2F	NEDD8-conjugating enzyme UBE2F	21.133	7	1	2	#N/A	#N/A	#N/A
A2VE20	UBE2G1	UBE2G1 protein	19.509	7.1	1	2	#N/A	#N/A	#N/A
Q32LN1	UBE2H	Ubiquitin-conjugating enzyme E2 H	20.655	13.1	2	2	#N/A	#N/A	#N/A
A6H744	UBE2I	SUMO-conjugating enzyme UBC9	18.007	22.8	4	6	#N/A	#N/A	#N/A
P61085	UBE2K	Ubiquitin-conjugating enzyme E2 K	22.406	49	8	13	17.5	3	8
F1MC72	UBE2L3	Ubiquitin-conjugating enzyme E2 L3 (Fragment)	16.944	67.1	6	23	30.1	5	12
A3KN22	UBE2M	NEDD8-conjugating enzyme Ubc12	20.9	45.4	9	10	22.4	5	5
Q0P5K3	UBE2N	Ubiquitin-conjugating enzyme E2 N	17.138	59.2	8	22	30.3	5	19
F1N3I3	UBE2O	Uncharacterized protein	140.39	10.3	12	18	2.6	4	5
E1BIE5	UBE2Q1	Uncharacterized protein	46.126	29.4	10	15	9.7	5	9
E1BAB9	UBE2R2	Uncharacterized protein	25.732	14.2	3	5	#N/A	#N/A	#N/A
Q3SZ52	UBE2V1	Ubiquitin-conjugating enzyme E2	16.495	49.7	2	18	15.6	1	10
Q3SZ43	UBE2V2	Ubiquitin-conjugating enzyme E2	16.363	53.8	2	3	#N/A	#N/A	#N/A
F1N1X8	UBE2Z	Uncharacterized protein	38.412	19.7	7	16	9.3	4	9
A4IFN7	UBE3A	UBE3A protein	100.36	9.8	9	13	7.9	7	14
E1BCU1	UBE3B	Uncharacterized protein	122.96	10.8	12	16	5.1	6	6
F1N703	UBE3C	Uncharacterized protein	123.38	3.3	3	4	#N/A	#N/A	#N/A
G3MZF7	UBFD1	Uncharacterized protein	33.038	20.9	4	24	13.1	3	6
Q3SZ34	UBL4A	Ubiquitin-like 4A	21.699	27.7	5	8	24.6	5	9
Q3T0Z3	UBL5	Ubiquitin-like protein 5	8.5468	12.3	1	1	#N/A	#N/A	#N/A
Q2KJD7	UBLCP1	Ubiquitin-like domain-containing CTD phosphatase 1	36.804	7.5	2	2	#N/A	#N/A	#N/A

				Biological Replicate 1			Biological Replicate 2		
Accession number	Gene symbol	Discription	MW(kDa)	Sequence coverage(%)	Unique peptides	MS/MS count	Sequence coverage(%)	Unique peptides	MS/MS count
F1N1A3	UBQLN1	Uncharacterized protein	62.412	15.6	6	10	4.9	2	3
E1BIW0	UBR1	Uncharacterized protein	199.46	2.8	4	6	#N/A	#N/A	#N/A
E1BCW1	UBR2	Uncharacterized protein	200.52	3.6	7	9	1.9	4	4
F1MQK6	UBR3	Uncharacterized protein (Fragment)	213.48	0.5	1	1	#N/A	#N/A	#N/A
E1BHT5	UBR4	Uncharacterized protein	572.59	0.2	1	1	#N/A	#N/A	#N/A
A3KMX6	UBR7	UBR7 protein	48.222	16.9	5	6	#N/A	#N/A	#N/A
Q3ZBU9	UBXN4	UBX domain-containing protein 4	56.784	3.3	1	2	3.1	2	2
Q2KIJ6	UBXN6	UBX domain-containing protein 6	49.732	14.5	6	8	7.3	4	7
F1MUA8	UBXN7	Uncharacterized protein (Fragment)	53.668	4.2	2	3	#N/A	#N/A	#N/A
P23356	UCHL1	Ubiquitin carboxyl-terminal hydrolase isozyme L1	28.335	67.5	18	229	54	15	120
Q2TBG8	UCHL3	Ubiquitin carboxyl-terminal hydrolase isozyme L3	26.181	60	12	37	43.9	8	18
Q9XSJ0	UCHL5	Ubiquitin carboxyl-terminal hydrolase isozyme L5	37.431	5.2	1	2	#N/A	#N/A	#N/A
Q0P5A4	UCK1	Uridine-cytidine kinase 1	31.195	6.9	2	3	#N/A	#N/A	#N/A
E1BP85	UCKL1	Uridine-cytidine kinase	60.866	7.3	4	5	#N/A	#N/A	#N/A
A5PJF9	UEVLD	UEVLD protein	52.237	3.2	2	2	#N/A	#N/A	#N/A
Q5E953	UFC1	Ubiquitin-fold modifier-conjugating enzyme 1	19.431	32.3	6	8	#N/A	#N/A	#N/A
Q0P568	UFD1L	Ubiquitin fusion degradation 1 like	26.071	23.8	4	10	10	2	5
A1A4I9	UFL1	E3 UFM1-protein ligase 1	89.382	15.8	11	14	8.6	6	12
Q2KJG2	UFM1	Ubiquitin-fold modifier 1	9.1675	35.3	3	6	#N/A	#N/A	#N/A
E1BK69	UFSP2	Uncharacterized protein	53.136	9.8	4	5	#N/A	#N/A	#N/A
E1B9R3	UGGT1	Uncharacterized protein	177.69	15	19	23	6.2	9	16
F1MSL9	UGGT2	Uncharacterized protein (Fragment)	174.79	29.2	43	93	15.7	25	75
Q07130	UGP2	UTP--glucose-1-phosphate uridylyltransferase	56.903	60	30	96	39.4	21	84
Q1LZI1	UGT3A1	UDP-glucuronosyltransferase 3A1	59.681	1.9	1	1	#N/A	#N/A	#N/A
A7E320	UHRF1	E3 ubiquitin-protein ligase UHRF1	88.338	26.2	17	32	14.8	9	12
F1MFX7	UHRF2	Uncharacterized protein (Fragment)	83.881	54	31	161	41.2	26	122
F1N332	ULK3	Uncharacterized protein	53.351	7.6	3	3	2.1	1	2

				Biological Replicate 1			Biological Replicate 2		
Accession number	Gene symbol	Discription	MW(kDa)	Sequence coverage(%)	Unique peptides	MS/MS count	Sequence coverage(%)	Unique peptides	MS/MS count
P31754	UMPS	Uridine 5-monophosphate synthase	52.229	9.2	4	4	2.5	1	1
Q3SYR2	UNC119	Protein unc-119 homolog A	26.987	3.8	1	1	#N/A	#N/A	#N/A
F1MP30	UNC13B	Uncharacterized protein	180.73	2.4	3	4	1.2	2	2
A5PKJ5	UNC45A	UNC45A protein	101.87	9.4	8	9	1.9	2	2
E1BEK9	UPF1	Uncharacterized protein	124.28	14.6	14	18	3.1	5	7
Q2T9M5	UPF3B	UPF3 regulator of nonsense transcripts homolog B (Yeast)	36.349	#N/A	#N/A	#N/A	2.9	1	1
Q32LA4	UPRT	Uracil phosphoribosyltransferase	33.354	8.8	3	3	#N/A	#N/A	#N/A
F6RX10	UQCC	Uncharacterized protein	34.031	11.8	4	6	#N/A	#N/A	#N/A
Q3SZ13	UQCC2	Ubiquinol-cytochrome-c reductase complex assembly factor 2	15.975	20.6	3	3	#N/A	#N/A	#N/A
P00129	UQCRB	Cytochrome b-c1 complex subunit 7	13.476	29.7	4	6	30.6	4	11
P31800	UQCRC1	Cytochrome b-c1 complex subunit 1, mitochondrial	52.735	46	18	65	34.2	14	39
P23004	UQCRC2	Cytochrome b-c1 complex subunit 2, mitochondrial	48.148	57.4	20	62	26	12	46
G3MXD1	UQCRFS1	Cytochrome b-c1 complex subunit Rieske, mitochondrial	29.92	27.7	7	28	16.6	5	12
P13271	UQCRQ	Cytochrome b-c1 complex subunit 8	9.7201	42.7	4	9	42.7	4	11
E1BEX4	UROD	Uroporphyrinogen decarboxylase	40.811	17.2	6	9	5.7	2	2
Q148D2	USE1	Uncharacterized protein	29.463	11.9	3	4	#N/A	#N/A	#N/A
P41541	USO1	General vesicular transport factor	107.51	27.5	25	59	15.3	15	29
A1L506	USP11	Ubiquitin carboxyl-terminal	86.426	2.8	1	1	#N/A	#N/A	#N/A
Q0IIF7	USP14	Ubiquitin carboxyl-terminal	56.012	13.6	6	8	#N/A	#N/A	#N/A
Q2HJE4	USP15	Ubiquitin carboxyl-terminal	109.44	17.5	14	26	10	9	17
F1MUD4	USP19	Ubiquitin carboxyl-terminal hydrolase (Fragment)	157.69	3.1	4	6	0.8	1	1
E1BDG7	USP28	Ubiquitin carboxyl-terminal	122.73	2.2	2	2	#N/A	#N/A	#N/A
A6QNM7	USP33	Ubiquitin carboxyl-terminal	103.32	3.1	2	5	#N/A	#N/A	#N/A
F1N0A8	USP34	Ubiquitin carboxyl-terminal hydrolase (Fragment)	404.3	#N/A	#N/A	#N/A	0.2	1	1
E1BG52	USP35	Ubiquitin carboxyl-terminal	113.13	2.1	2	2	#N/A	#N/A	#N/A

				Biological Replicate 1			Biological Replicate 2		
Accession number	Gene symbol	Discription	MW(kDa)	Sequence coverage(%)	Unique peptides	MS/MS count	Sequence coverage(%)	Unique peptides	MS/MS count
A6QR55	USP4	Ubiquitin carboxyl-terminal	108.51	8.8	5	7	#N/A	#N/A	#N/A
F1N1Z2	USP47	Ubiquitin carboxyl-terminal hydrolase (Fragment)	157.58	6.8	10	16	2.2	3	5
F1N3P2	USP5	Ubiquitin carboxyl-terminal	95.814	37.9	25	73	15.9	12	36
F1N556	USP7	Ubiquitin carboxyl-terminal hydrolase (Fragment)	126.54	21	20	29	7.7	9	16
Q0IIM6	USP8	Ubiquitin carboxyl-terminal	124.24	11.4	12	17	3.8	5	8
G5E630	USP9X	Ubiquitin carboxyl-terminal	290.3	1.5	4	4	2.6	7	9
E1BMI4	UXS1	Uncharacterized protein	47.747	8.1	3	3	#N/A	#N/A	#N/A
Q32P97	UXT	Protein UXT	18.083	#N/A	#N/A	#N/A	5.1	1	2
F1MJZ1	VAC14	Protein VAC14 homolog	87.828	#N/A	#N/A	#N/A	1.3	1	2
G3X752	VAMP3	Vesicle-associated membrane protein 3 (Fragment)	11.404	38.8	4	8	23.3	2	4
Q17QI5	VAMP7	Vesicle-associated membrane protein	24.962	50.5	11	27	25.5	6	15
Q0VCY1	VAPA	Vesicle-associated membrane protein-associated protein A	27.856	20.9	7	13	21.3	4	9
A2VDZ9	VAPB	Vesicle-associated membrane protein-associated protein B	27.158	53.5	13	53	36.6	9	57
E1BLV6	VARS	Uncharacterized protein (Fragment)	112	22.7	22	60	16.9	16	36
F1N6L1	VARS2	Uncharacterized protein (Fragment)	121.76	3.4	2	5	#N/A	#N/A	#N/A
Q2TA49	VASP	Vasodilator-stimulated	40.463	12.8	4	6	9.7	3	3
F1MUP9	VAT1	Uncharacterized protein	42.84	38.1	13	48	17.4	7	21
Q2TBX2	VBP1	Prefoldin subunit 3	22.482	51.3	11	33	50.8	11	27
F1N789	VCL	Uncharacterized protein	123.86	37.7	37	66	21.4	26	39
Q3ZBT1	VCP	Transitional endoplasmic reticulum	89.329	58.8	43	166	36.7	28	121
E1BCH9	VCPIP1	Uncharacterized protein	134.61	13.3	12	18	8.4	9	12
P45879	VDAC1	Voltage-dependent anion-selective channel protein 1	30.74	72.1	16	55	39.6	10	79
P68002	VDAC2	Voltage-dependent anion-selective channel protein 2	31.619	62.6	14	50	26.9	8	51
Q9MZ13	VDAC3	Voltage-dependent anion-selective channel protein 3	30.738	60.8	15	84	43.1	11	75

				Biological Replicate 1			Biological Replicate 2		
Accession number	Gene symbol	Discription	MW(kDa)	Sequence coverage(%)	Unique peptides	MS/MS count	Sequence coverage(%)	Unique peptides	MS/MS count
P48616	VIM	Vimentin	53.727	54.1	27	63	45.1	20	50
Q6B4J2	VKORC1	Vitamin K epoxide reductase complex subunit 1	17.967	16	3	10	19.6	3	8
G3MZM8	VKORC1L1	Uncharacterized protein (Fragment)	12.528	18	2	5	8.1	1	2
F1MIQ2	VLDLR	Uncharacterized protein	93.121	#N/A	#N/A	#N/A	1.3	1	1
Q0VCK9	VMP1	Vacuole membrane protein 1	46.182	#N/A	#N/A	#N/A	2.2	1	1
F1N5Q5	VPS11	Uncharacterized protein	107.6	2	2	2	4	4	5
Q5E9L7	VPS16	Vacuolar protein sorting-associated protein 16 homolog	94.701	9.1	6	6	1.8	1	1
A6QR33	VPS18	Uncharacterized protein	110.07	6.5	7	7	0.8	1	2
Q5E9A6	VPS25	Vacuolar protein-sorting-associated protein 25	20.746	27.8	4	7	11.4	2	2
Q0VD53	VPS26A	Vacuolar protein sorting-associated protein 26A	38.155	36.1	8	16	17.4	4	12
E1BMM4	VPS26B	Uncharacterized protein (Fragment)	38.834	23.5	6	6	5.4	1	1
Q3T178	VPS28	Vacuolar protein sorting-associated protein 28 homolog	25.484	44.8	8	11	17.6	3	6
G3X6P5	VPS29	Vacuolar protein sorting-associated protein 29 (Fragment)	20.786	32.4	6	15	21.6	4	22
F1MT37	VPS33A	Uncharacterized protein	67.506	13.1	7	7	3.2	2	2
Q2HJG5	VPS35	Vacuolar protein sorting-associated protein 35	91.75	41.5	30	80	22.2	17	45
F1MT69	VPS36	Vacuolar protein-sorting-associated protein 36	43.758	11.9	4	4	#N/A	#N/A	#N/A
Q29RR7	VPS37A	Uncharacterized protein	44.249	14.9	4	6	10.6	3	3
E1BC70	VPS37C	Uncharacterized protein	38.07	4.3	1	1	4.3	1	2
E1BM41	VPS39	Uncharacterized protein	100.62	3.4	3	3	2.2	2	2
A3KN15	VPS41	Uncharacterized protein	98.583	1.2	1	1	#N/A	#N/A	#N/A
F1MDA6	VPS45	Uncharacterized protein	65.104	16.5	10	17	5.4	3	7
F1MJ84	VPS51	Uncharacterized protein	77.832	13.5	3	17	5.4	4	5
E1BDC9	VPS52	Uncharacterized protein	82.323	11.9	8	10	4.6	3	4
E1BJW7	VPS53	Uncharacterized protein	94.446	9.1	8	13	7.6	7	12

				Biological Replicate 1			Biological Replicate 2		
Accession number	Gene symbol	Discription	MW(kDa)	Sequence coverage(%)	Unique peptides	MS/MS count	Sequence coverage(%)	Unique peptides	MS/MS count
Q32PI1	VRK1	Serine/threonine-protein kinase	45.357	28	10	16	7.1	3	6
Q32L63	VTA1	Vacuolar protein sorting-associated protein VTA1 homolog	34.016	39.4	12	26	20.2	6	23
Q2KIU0	VTI1B	Vesicle transport through interaction with t-SNAREs homolog	26.708	21.1	4	6	17.2	4	10
E1BGC3	WAPAL	Uncharacterized protein	133.27	1.4	1	1	#N/A	#N/A	#N/A
P17248	WARS	Tryptophan--tRNA ligase,	53.811	47.5	20	43	18.7	9	25
Q3T099	WARS2	Tryptophan--tRNA ligase,	40.204	13.1	3	3	3.1	1	2
A2VDK6	WASF2	Wiskott-Aldrich syndrome protein family member 2	54.03	4.9	2	2	1.6	1	2
A7Z063	WASH1	WAS protein family homolog 1	50.772	16.8	8	17	8.7	5	10
Q95107	WASL	Neural Wiskott-Aldrich syndrome	54.671	13.3	6	10	5.1	4	4
E1BNM6	WBP11	Uncharacterized protein	70.128	2.2	1	3	#N/A	#N/A	#N/A
E1BGX4	WBP2	Uncharacterized protein	37.241	21.7	9	32	12	6	22
A7MBG9	WBSCR1	Eukaryotic translation initiation factor 4H	24.472	25.2	4	10	3.2	1	2
E1BIS5	WBSCR16	Uncharacterized protein	50.006	1.7	1	2	#N/A	#N/A	#N/A
Q2KIY3	WDFY1	WD repeat and FYVE domain-containing protein 1	46.28	5.1	2	2	#N/A	#N/A	#N/A
Q2KJH4	WDR1	WD repeat-containing protein 1	66.257	68.2	35	150	38	24	93
Q08D81	WDR13	Uncharacterized protein	53.684	2.3	1	1	#N/A	#N/A	#N/A
Q3SZD4	WDR18	WD repeat-containing protein 18	47.611	13.7	5	5	12.5	5	6
E1B7Q4	WDR19	Uncharacterized protein	151.77	1.3	2	4	#N/A	#N/A	#N/A
E1BDQ9	WDR20	Uncharacterized protein	62.435	3.7	2	2	#N/A	#N/A	#N/A
F1MJS7	WDR24	Uncharacterized protein	88.263	1.6	1	1	3.9	3	3
Q2TBM8	WDR26	Uncharacterized protein	58.577	14.2	5	5	1.9	1	2
E1BCT7	WDR33	Uncharacterized protein	145.86	#N/A	#N/A	#N/A	1.3	2	3
F1MTS5	WDR34	Uncharacterized protein	57.658	3	2	2	#N/A	#N/A	#N/A
Q9XSC3	WDR44	WD repeat-containing protein 44	101.29	5.9	5	8	2.4	2	3
E1BP79	WDR47	Uncharacterized protein	102.34	12.3	9	17	4.7	4	4
Q32PG3	WDR48	WD repeat-containing protein 48	76.192	6.1	5	6	7.1	4	4
G3N1S7	WDR52	Uncharacterized protein (Fragment)	115.08	0.6	1	1	#N/A	#N/A	#N/A

				Biological Replicate 1			Biological Replicate 2		
Accession number	Gene symbol	Discription	MW(kDa)	Sequence coverage(%)	Unique peptides	MS/MS count	Sequence coverage(%)	Unique peptides	MS/MS count
A7MBJ6	WDR59	Uncharacterized protein	109.76	1.4	1	1	#N/A	#N/A	#N/A
Q32LN7	WDR61	WD repeat-containing protein 61	33.568	30.2	5	11	12.5	4	7
Q32LB0	WDR70	WD repeat-containing protein 70	72.64	2.9	2	2	#N/A	#N/A	#N/A
G3MZF6	WDR76	Uncharacterized protein	70.41	1.9	1	1	#N/A	#N/A	#N/A
Q5E9I7	WDR77	Methylosome protein 50	36.789	29.2	7	18	11.4	3	9
A7Z049	WDR81	Uncharacterized protein	96.132	#N/A	#N/A	#N/A	1.2	1	2
Q2HJE1	WDR91	WD repeat-containing protein 91	83.156	9.8	7	15	1.6	1	2
Q29RZ9	WDR92	WD repeat-containing protein 92	39.817	7.3	1	1	#N/A	#N/A	#N/A
F1MRF3	WDSUB1	Uncharacterized protein	52.95	9.2	3	3	#N/A	#N/A	#N/A
F1MZD1	WEE2	Uncharacterized protein	62.464	19.6	11	42	14.3	7	22
G3N348	WFS1	Uncharacterized protein	99.741	11.8	11	18	1.9	2	2
F1MW50	WHSC2	Uncharacterized protein	45.968	8.1	3	5	#N/A	#N/A	#N/A
A6QPH1	WIBG	Partner of Y14 and mago	22.662	14.8	2	2	23.6	3	3
E1B777	WIPF2	Uncharacterized protein	46.141	14.8	5	12	7.5	3	5
Q2KJ60	WIPI2	Uncharacterized protein	47.622	5.3	3	3	4.4	2	4
E1BDN1	WLS	Uncharacterized protein	62.268	3	2	3	7.4	4	6
E1BN63	WNK1	Uncharacterized protein	247.46	1.6	5	6	0.7	2	2
F1MGL0	WRNIP1	Uncharacterized protein (Fragment)	53.401	6.1	3	3	#N/A	#N/A	#N/A
Q0P5N4	WWOX	Uncharacterized protein	46.191	13.8	5	5	2.4	1	1
A3KMV3	WWP2	E3 ubiquitin-protein ligase	98.695	3.4	1	2	1	1	1
G3MWG5	XIAP	Uncharacterized protein	56.533	1.8	1	1	#N/A	#N/A	#N/A
E1BL04	XIRP2	Uncharacterized protein	428.73	0.3	1	5	#N/A	#N/A	#N/A
Q1JPJ2	XPNPEP1	Xaa-Pro aminopeptidase 1	69.789	51.8	23	45	12.8	8	17
E1B735	XPNPEP2	Uncharacterized protein	75.635	#N/A	#N/A	#N/A	1.3	1	6
E1BE98	XPO1	Uncharacterized protein	123.14	36.4	31	74	7.3	8	16
F1MUF1	XPO5	Uncharacterized protein (Fragment)	132.42	7.5	8	11	2.5	4	6
F1MI29	XPO6	Uncharacterized protein (Fragment)	128.77	5.3	5	9	1.2	2	4
A1A4I8	XPO7	Uncharacterized protein	123.89	22.1	22	36	14	17	27
E1B8Q8	XPOT	Uncharacterized protein	110.28	16	13	22	#N/A	#N/A	#N/A
F1MHL9	XPR1	Uncharacterized protein	81.57	4	3	3	1.1	1	1
F1N2F9	XRCC1	Uncharacterized protein (Fragment)	70.482	3.9	3	3	4	3	5
G5E5D1	XRCC5	Uncharacterized protein	83.006	9.1	6	14	3.8	3	3

				Biological Replicate 1			Biological Replicate 2		
Accession number	Gene symbol	Discription	MW(kDa)	Sequence coverage(%)	Unique peptides	MS/MS count	Sequence coverage(%)	Unique peptides	MS/MS count
F1MMD5	XRCC6	Uncharacterized protein	70.476	11.1	5	6	3.8	2	4
Q3SYZ6	XYLB	Xylulose kinase	53.304	2.2	1	3	#N/A	#N/A	#N/A
F1MHM5	YARS	Tyrosine--tRNA ligase, cytoplasmic	59.16	24.8	12	19	11.9	7	11
A5D7G0	YARS2	Uncharacterized protein	52.991	21.3	7	11	7.5	3	8
P67808	YBX1	Nuclease-sensitive element-binding protein 1	35.924	18.2	1	1	18.2	1	2
A5D7M4	YBX2	Uncharacterized protein	38.268	53.1	13	130	49.4	9	57
F1MC73	YDJC	Uncharacterized protein	34.294	6.2	2	3	5.3	2	3
Q32LE1	YEATS4	Uncharacterized protein	26.543	6.6	1	2	#N/A	#N/A	#N/A
A7MB57	YES1	Uncharacterized protein	60.596	4.8	3	7	4.6	2	2
E1BGG4	YIF1B	Uncharacterized protein	34.142	4.2	1	1	#N/A	#N/A	#N/A
A7MB97	YIPF2	Uncharacterized protein	34.899	2.6	1	1	#N/A	#N/A	#N/A
F1MHD7	YIPF4	Uncharacterized protein	26.693	3.8	1	1	#N/A	#N/A	#N/A
Q5E9E8	YIPF5	Protein YIPF5	28.016	3.5	1	1	3.5	1	7
A6QLC6	YIPF6	Protein YIPF6	26.184	13.6	3	6	#N/A	#N/A	#N/A
Q3T000	YKT6	Synaptobrevin homolog YKT6	22.466	57.6	11	31	22.2	5	14
A6QR12	YME1L1	Uncharacterized protein	80.059	7	4	4	4.5	3	3
Q0VC80	YRDC	YrdC domain-containing protein, mitochondrial	28.893	4.3	1	2	#N/A	#N/A	#N/A
E1BAQ2	YTHDF1	Uncharacterized protein	60.717	3.7	2	2	#N/A	#N/A	#N/A
Q0VCZ3	YTHDF2	YTH domain family protein 2	62.449	#N/A	#N/A	#N/A	3.6	1	1
E1BH80	YTHDF3	Uncharacterized protein	63.74	#N/A	#N/A	#N/A	6.5	2	5
P62261	YWHAE	14-3-3 protein epsilon	29.174	71	19	144	51	12	72
P68509	YWHAH	14-3-3 protein eta	28.211	61.4	11	32	25.6	4	11
Q3SZI4	YWHAQ	14-3-3 protein theta	27.764	66.1	11	28	36.7	7	17
P63103	YWHAZ	14-3-3 protein zeta/delta	27.745	74.7	14	108	47.8	9	62
Q1PSA0	ZAR1	Uncharacterized protein	42.009	23.4	9	22	21.9	9	36
Q1XFL1	ZAR1L	ZAR1-like protein	36.053	34.6	9	17	19.8	6	18
Q1RMM1	ZC3H15	Zinc finger CCCH domain-containing protein 15	48.527	12	5	6	6.1	3	5
F1MQG4	ZDBF2	Uncharacterized protein	252.21	0.6	1	2	#N/A	#N/A	#N/A
F1MCX6	ZDHHC13	Palmitoyltransferase	71.065	15.6	8	12	14.5	8	22

				Biological Replicate 1			Biological Replicate 2		
Accession number	Gene symbol	Discription	MW(kDa)	Sequence coverage(%)	Unique peptides	MS/MS count	Sequence coverage(%)	Unique peptides	MS/MS count
A5PJK9	ZFP3	Uncharacterized protein	58.337	#N/A	#N/A	#N/A	2	1	1
Q2YDD3	ZFPL1	Zinc finger protein-like 1	34.401	13.1	4	7	7.1	2	2
G3N025	ZFYVE1	Uncharacterized protein	86.954	10.3	6	7	3.6	3	6
Q2HJ64	ZFYVE19	Uncharacterized protein	44.232	7.8	2	3	#N/A	#N/A	#N/A
F1MKY4	ZFYVE9	Uncharacterized protein	156.22	#N/A	#N/A	#N/A	0.6	1	1
G3MWN0	ZGLP1	Uncharacterized protein (Fragment)	27.123	#N/A	#N/A	#N/A	3.6	1	1
A8KC64	ZHX1	Uncharacterized protein	98.124	#N/A	#N/A	#N/A	2.3	1	1
F1MVH9	ZNF207	Uncharacterized protein	45.283	5.7	2	3	#N/A	#N/A	#N/A
Q2TBX0	ZNF259	Zinc finger protein ZPR1	51.015	13.5	6	12	#N/A	#N/A	#N/A
G3N065	ZNF385C	Uncharacterized protein	53.189	2	1	1	#N/A	#N/A	#N/A
E1BF46	ZNF407	Uncharacterized protein	239.98	0.7	2	9	0.7	2	11
E1BMZ4	ZNF436	Uncharacterized protein	54.549	2.8	1	1	4.7	2	2
G3X749	ZNF503	Uncharacterized protein	51.193	#N/A	#N/A	#N/A	1.9	1	2
F1MEB7	ZNF645	Uncharacterized protein	54.505	16.5	6	11	3.5	2	3
Q9BH10	ZP2	Zona pellucida sperm-binding	79.547	38.3	22	496	20.3	12	465
P48830	ZP3	Zona pellucida sperm-binding	46.545	26.4	13	345	28	14	272
Q9BH11	ZP4	Zona pellucida sperm-binding	59.2	25.5	13	366	22.1	10	122
A7YWH2	ZRANB2	Uncharacterized protein	36.291	7.2	2	2	#N/A	#N/A	#N/A
Q0P5L6	ZW10	Uncharacterized protein	88.416	21.7	14	22	6.2	5	14
A6QM04	ZWILCH	Protein zwilch homolog	66.384	22.6	12	19	6.3	4	6
A4FUE7; G1K1W5	ZC2HC1A	Zinc finger C2HC domain-containing protein 1A	35.113	23.5	9	14	10	3	4
A4IFM2; Q3ZKN0	SLC27A1	Long-chain fatty acid transport protein 1	71.023	12.7	8	12	8.2	4	4
A5D7P3; P43033	PAFAH1B1	Platelet-activating factor acetylhydrolase IB subunit alpha	46.67	30.5	12	23	27.3	10	19
A5D7S0; P81125	NAPA	Alpha-soluble NSF attachment protein	33.204	59	14	30	24.1	6	17
A5PK73; Q3T0S5	ALDOB	Fructose-bisphosphate aldolase	39.527	29.9	9	12	27.7	8	13
A6H7J6; P05307	P4HB	P4HB protein	57.203	72	43	205	50.2	29	219

				Biological Replicate 1			Biological Replicate 2		
Accession number	Gene symbol	Discription	MW(kDa)	Sequence coverage(%)	Unique peptides	MS/MS count	Sequence coverage(%)	Unique peptides	MS/MS count
A6QLC7; F1MXL8	TRAPPC11	Trafficking protein particle complex subunit 11	128.27	26.6	24	37	9	10	22
A6QLW5; Q58DN0	DSCR3	DSCR3 protein	32.57	16.8	4	10	4.6	1	1
A6QR09; E1BJ79	SLC25A26	S-adenosylmethionine mitochondrial carrier protein	29.409	20.8	4	7	6.2	2	4
A7Z057; P68252	YWHAG	14-3-3 protein gamma	28.302	66	9	30	30.4	6	21
A8E4Q4; Q58DH2	PISD	PISD protein	31.769	13.9	4	8	7.5	4	6
A8QQF2; E1BHQ9	MCAM	MCAM protein	49.002	2	1	2	3.3	2	3
C7FFR6; Q1JQ93	DPM1	Dolichol-phosphate mannosyltransferase	29.606	40	11	21	20.8	6	11
E1BB06; G3N135	FMNL2	Uncharacterized protein	98.583	4.6	1	5	1.9	2	2
E1BB79; G3N0M0	TNN	Uncharacterized protein	98.583	2	2	3	1.9	2	3
E1BCE7; F1MID5	CNOT2	Uncharacterized protein (Fragment)	16.346	10.4	5	6	4.2	2	6
E1BHW7; G3N2P9	C18orf8	Uncharacterized protein	98.583	4.4	3	3	2.2	1	2
E1BL56; E1BH11	SH3BP1	Uncharacterized protein	98.583	20.6	13	24	8.5	4	8
E1BL66; G3MX31	LOC101902617	Uncharacterized protein	98.583	27.8	5	9	23	1	1
E1BP30; Q1LZ76	RTN1	Reticulon (Fragment)	75.828	4.7	3	4	7.7	2	8
F1MD39; P59837	RDH12	Retinol dehydrogenase 12	35.084	35.1	10	19	18.7	5	9
F1ME95; Q148L1	GPR108	Protein GPR108	42.253	4.5	2	2	5.1	3	8

				Biological Replicate 1			Biological Replicate 2		
Accession number	Gene symbol	Discription	MW(kDa)	Sequence coverage(%)	Unique peptides	MS/MS count	Sequence coverage(%)	Unique peptides	MS/MS count
F1MGC0; Q148D5	SUCLA2	Succinyl-CoA ligase [ADP-forming] subunit beta, mitochondrial	50.146	43.2	18	51	26.1	15	49
F1MLB8; P19483	ATP5A1	ATP synthase subunit alpha	59.689	68.9	1	144	39.8	23	126
F1MMI4; Q32KV2	MAEL	Protein maelstrom homolog	47.375	34.5	11	22	7.6	3	5
F1MMY6; Q3SYU7	TNPO1	Transportin-1 (Fragment)	97.325	20.5	10	32	9	5	22
F1MNT3; P16386	LIPE	Hormone-sensitive lipase	82.638	8.7	6	10	3.2	3	4
F1MVI1; Q8HXM1	CSTF2	Cleavage stimulation factor subunit 2	62.436	9	4	9	4.9	3	4
F1MVW4; A5PJX2	XPO4	Uncharacterized protein (Fragment)	16.346	5.7	5	6	1.5	1	1
F1MW91; A6H7I5	DNM2	Dynamin-2 (Fragment)	92.283	29.3	17	46	16.6	16	36
F1MX38; P61635	STAT3	Signal transducer and activator of transcription	86.929	29.7	18	27	7.3	6	9
F1MYH8; Q3SYT7	PSMD8	26S proteasome non-ATPase regulatory subunit 8	32.523	29.3	9	15	16.7	6	15
F1MZD5; Q8SPU8	DHRS4	Dehydrogenase/reductase SDR family member 4	29.454	15.1	4	5	20.8	6	15
F1MZF0; A6QPB2	GRSF1	Uncharacterized protein (Fragment)	16.346	26.6	7	14	2.8	1	3
F1MZN7; Q2TBW7	SNX2	Sorting nexin-2	58.434	18.9	7	10	9.1	3	3
F1N0E5; Q2T9X2	CCT4	T-complex protein 1 subunit delta	58.161	66.8	32	108	43.4	25	95
F1N0N6; Q32P66	C3H1orf123	Uncharacterized protein	98.583	29.4	5	9	34.4	5	11
F1N103; Q5EA64	COCH	Cochlin	59.595	37.5	20	79	22.2	13	54

				Biological Replicate 1			Biological Replicate 2		
Accession number	Gene symbol	Discription	MW(kDa)	Sequence coverage(%)	Unique peptides	MS/MS count	Sequence coverage(%)	Unique peptides	MS/MS count
F1N1S0; Q32PB0	SSBP1	Single-stranded DNA-binding protein	17.191	37.8	7	22	33.8	6	13
F1N2B5; Q08DL0	SLC3A2	Uncharacterized protein	98.583	30	11	22	11.9	6	13
F1N2L9; Q2KJH9	ALDH9A1	4-trimethylaminobutyraldehyde dehydrogenase	53.991	25.5	12	29	24.9	11	36
F1N3H1; Q3T0K1	CALU	Calumenin	37.086	32.7	10	21	30.5	11	26
F1N6Z0; Q0P5A6	PSMD5	26S proteasome non-ATPase regulatory subunit 5	56.17	53.6	21	58	19.7	9	23
F1N7N8; P09851	RASA1	Ras GTPase-activating protein 1	115.86	41.4	37	80	10.7	13	27
F2Z4I1; Q56JV3	RHEB	GTP-binding protein Rheb	20.479	56.5	11	23	27.7	7	19
F2Z4J5; Q3ZC12	EIF3G	Eukaryotic translation initiation factor 3 subunit G	35.611	31.9	8	16	19.1	6	8
F2Z4J6; Q2KIN8	GTF2B	Transcription initiation factor IIB	34.833	25	6	8	14.2	4	5
F6Q200; Q1JPJ6	PDK2	Uncharacterized protein	98.583	14.5	4	6	9.3	2	2
G3MX91; Q2KJ45	TARDBP	Uncharacterized protein	98.583	12.3	4	5	5.7	2	2
G3X780; Q32LJ6	RABL3	Rab-like protein 3 (Fragment)	21.562	10	2	2	12.7	3	7
G3X8E2; Q2HJB1	VPS4A	Uncharacterized protein (Fragment)	16.346	13	5	9	9.1	4	5
G8JKV4; Q9N0F3	SARS2	Serine--tRNA ligase, mitochondrial	51.722	36.6	13	29	9.7	4	6
O02751; F1MS40	CFDP2	Craniofacial development protein 2	66.324	29.9	7	9	16.6	7	30
O46414; G1K1K0	FTH1	Ferritin heavy chain	21.051	27.6	4	8	32.8	6	21

				Biological Replicate 1			Biological Replicate 2		
**Accession number**	**Gene symbol**	**Discription**	**MW(kDa)**	**Sequence coverage(%)**	**Unique peptides**	**MS/MS count**	**Sequence coverage(%)**	**Unique peptides**	**MS/MS count**
P19803; Q58DT6	ARHGDIA	Rho GDP-dissociation inhibitor 1	23.421	65.7	13	54	32	4	13
P82931; G3X705	MRPS6	28S ribosomal protein S6, mitochondrial	14.147	21.8	3	7	28.9	4	10
Q07536; F1N7K8	ALDH6A1	Methylmalonate-semialdehyde dehydrogenase [acylating], mitochondrial	58.062	21.4	11	16	8.8	5	11
Q0VCQ4; G3N1Z2	MRRF	Ribosome-recycling factor, mitochondrial	28.974	16.8	3	3	11.1	1	3
Q1RMU3; A6QL77	P4HA1	Prolyl 4-hydroxylase subunit alpha-1	61.009	53.7	24	58	28.7	2	42
Q2KIM7; F1N540	ALG5	Asparagine-linked glycosylation 5, dolichyl-phosphate beta-glucosyltransferase homolog (S. cerevisiae)	32.857	5.9	1	1	5.9	2	2
Q2KIZ8; E1BH89	MCM6	DNA replication licensing factor MCM6	83.104	29.8	2	41	12.5	9	13
Q2TBT7; A6QLG6	FXR1	Fragile X mental retardation syndrome-related protein 1	69.75	16.3	8	13	16	6	8
Q32KX0; F1N6A0	ISOC2	Isochorismatase domain-containing protein 2, mitochondrial	22.42	15.7	3	7	6.9	2	3
Q32LK1; E1BP20	AARSD1	Alanyl-tRNA editing protein Aarsd1	48.983	6.5	3	4	3.9	3	4
Q32LM0; F1N7B9	SH3GLB1	Endophilin-B1	40.777	46	16	30	20.7	7	20
Q3MHE8; F1MNJ2	SRPR	Signal recognition particle receptor subunit alpha	69.895	19.7	10	15	13.7	9	17
Q3MHH4; F1MTC2	QARS	Glutamine--tRNA ligase	87.642	26.5	16	28	5.6	4	10
Q3SYV4; A6QLB7	CAP1	Adenylyl cyclase-associated protein 1	51.272	67.2	1	71	30.8	17	60

				Biological Replicate 1			Biological Replicate 2		
Accession number	Gene symbol	Discription	MW(kDa)	Sequence coverage(%)	Unique peptides	MS/MS count	Sequence coverage(%)	Unique peptides	MS/MS count
Q3SZ76; F1MUK6	COMMD3	COMM domain-containing protein 3	22.31	45.6	6	10	12.6	2	5
Q3SZA5; F1ME02	SMS	Spermine synthase	41.224	62.2	24	69	27.8	12	33
Q3T0F5; F1MJQ1	RAB7A	Ras-related protein Rab-7a	23.544	78.3	16	54	53.6	11	60
Q3ZBL4; F1MNG7	LZTFL1	Leucine zipper transcription factor-like protein 1	34.577	27.4	8	10	7	2	2
Q58D01; Q58DA0	PSMD4	26S proteasome non-ATPase regulatory subunit 4	33.96	29.4	6	7	13.4	5	11
Q58D97; O77689	CCNB2	Cyclin B2	28.792	6.4	2	2	2.8	1	1
Q58DK4; A0JN77	DAK	Bifunctional ATP-dependent dihydroxyacetone kinase/FAD-AMP lyase (cyclizing)	59.012	34.6	15	30	15.1	8	17
Q5E9V6; Q58DF4	COMMD4	COMM domain-containing protein 4	21.648	28.1	6	15	23.5	5	12
Q7YS70; F1MEY2	MECR	Trans-2-enoyl-CoA reductase, mitochondrial	40.274	11	4	5	4	1	1
A1A4J7-2		Isoform 2 of Protein SMG8	105.02	5.9	5	7	#N/A	#N/A	#N/A
A4FV58-2		Isoform 2 of Cytosolic Fe-S cluster assembly factor NARFL	39.1	5.8	2	2	#N/A	#N/A	#N/A
A5PK51-2		Isoform 2 of Nicotinate phosphoribosyltransferase	57.387	2.8	1	1	#N/A	#N/A	#N/A
A6H754-2		Isoform 2 of Hsp90 co-chaperone Cdc37-like 1	35.282	8.2	3	4	8.2	3	5
A6QNS3-2		Isoform 2 of Active breakpoint cluster region-related protein	31.599	3.6	1	2	#N/A	#N/A	#N/A
A6QQF5		Quinone oxidoreductase-like protein	37.668	5.4	1	1	#N/A	#N/A	#N/A
E1B9D4		Uncharacterized protein	20.482	5.5	1	1	5	1	1
E1BAY3		Uncharacterized protein	54.378	#N/A	#N/A	#N/A	1.8	1	2

				Biological Replicate 1			Biological Replicate 2		
Accession number	Gene symbol	Discription	MW(kDa)	Sequence coverage(%)	Unique peptides	MS/MS count	Sequence coverage(%)	Unique peptides	MS/MS count
E1BBV5		Uncharacterized protein (Fragment)	18.39	#N/A	#N/A	#N/A	6.6	1	1
E1BBW6		Uncharacterized protein	13.116	#N/A	#N/A	#N/A	9.2	1	4
E1BC32		Uncharacterized protein	38.38	#N/A	#N/A	#N/A	3.3	1	2
E1BCT3		Uncharacterized protein	170.05	1	1	1	#N/A	#N/A	#N/A
E1BEF1		Uncharacterized protein	18.903	11.9	2	2	#N/A	#N/A	#N/A
E1BF78		Uncharacterized protein	18.548	28	4	6	11.3	2	4
E1BFC9		Uncharacterized protein	16.695	42.7	2	87	44.1	2	73
E1BFZ9		Uncharacterized protein	94.099	6.6	6	10	4.4	3	6
E1BH99		Uncharacterized protein	17.757	#N/A	#N/A	#N/A	7.3	1	2
E1BHJ0		Profilin	15.057	79.3	1	1	47.9	1	1
E1BIU3		Uncharacterized protein	21.086	14.5	2	2	#N/A	#N/A	#N/A
E1BJN0		Uncharacterized protein	19.936	28.1	5	9	11.1	2	6
E1BKY8		Uncharacterized protein	57.015	2.3	1	1	#N/A	#N/A	#N/A
E1BL61		Uncharacterized protein	12.562	#N/A	#N/A	#N/A	11.8	1	2
E1BMW2		Uncharacterized protein (Fragment)	106.91	23.8	10	13	14.5	4	4
E1BN74		Uncharacterized protein	33.439	12.9	4	6	#N/A	#N/A	#N/A
F1MB80		NADH dehydrogenase [ubiquinone] 1 alpha subcomplex subunit 2	10.903	22.4	3	5	10.2	2	7
F1MCK4		Uncharacterized protein	24.064	13.4	3	3	#N/A	#N/A	#N/A
F1MCL7		Uncharacterized protein	146.25	5.8	6	8	1.4	2	2
F1ME51		Uncharacterized protein	65.536	4.1	1	1	#N/A	#N/A	#N/A
F1ME91		Uncharacterized protein	38.878	13.1	1	3	8.1	1	3
F1MFW0		Uncharacterized protein (Fragment)	23.855	19.6	1	4	#N/A	#N/A	#N/A
F1MJU4		Uncharacterized protein (Fragment)	140.77	9.9	3	10	#N/A	#N/A	#N/A
F1MKM4		Uncharacterized protein	75.429	1.8	1	1	#N/A	#N/A	#N/A
F1MLM3		Uncharacterized protein (Fragment)	112.3	15.6	13	21	5	5	10
F1MLW8		Uncharacterized protein	24.623	13.7	1	4	6.9	1	37
F1MNP3		Uncharacterized protein	120.61	#N/A	#N/A	#N/A	1.2	1	2
F1MU60		Uncharacterized protein	18.389	14.5	2	2	11.5	1	1
F1MV39		Uncharacterized protein (Fragment)	21.498	30.3	6	17	20	3	7
F1MWK9		Uncharacterized protein (Fragment)	25.517	#N/A	#N/A	#N/A	12.8	3	4
F1N741		Uncharacterized protein (Fragment)	55.79	6.3	1	1	#N/A	#N/A	#N/A

				Biological Replicate 1			Biological Replicate 2		
Accession number	Gene symbol	Discription	MW(kDa)	Sequence coverage(%)	Unique peptides	MS/MS count	Sequence coverage(%)	Unique peptides	MS/MS count
F1N7U3		Uncharacterized protein	18.522	#N/A	#N/A	#N/A	19.4	4	6
F6R9F1		Uncharacterized protein	55.414	#N/A	#N/A	#N/A	2.4	1	2
F6RCB9		Uncharacterized protein	31.939	18.1	4	9	13.5	3	7
F6RP72		Uncharacterized protein (Fragment)	49.828	56.1	3	9	36.3	1	3
G3MWS9		Uncharacterized protein	34.928	6.9	2	2	#N/A	#N/A	#N/A
G3MXK7		Uncharacterized protein (Fragment)	22.314	26.7	1	7	27.7	1	6
G3MXP4		Uncharacterized protein (Fragment)	69.325	31.1	19	48	29.3	17	71
G3MZT9		Uncharacterized protein (Fragment)	37.832	20.9	1	1	#N/A	#N/A	#N/A
G3MZV0		Uncharacterized protein	18.344	#N/A	#N/A	#N/A	18.1	3	12
G3N058		Uncharacterized protein (Fragment)	16.346	9.1	1	1	9.1	1	3
G3N0K3		Uncharacterized protein	29.478	#N/A	#N/A	#N/A	23.3	6	13
G3N0Q3		Uncharacterized protein	89.129	43.7	28	96	27.4	16	74
G3N0V0		Uncharacterized protein (Fragment)	35.951	20.2	3	6	18.1	3	13
G3N220		Uncharacterized protein	31.674	#N/A	#N/A	#N/A	7.4	2	2
G3N2F0		Elongation factor 1-alpha	49.954	26.1	1	2	#N/A	#N/A	#N/A
G3N2X8		Uncharacterized protein (Fragment)	14.641	31	3	4	#N/A	#N/A	#N/A
G3X7J3		Uncharacterized protein	23.244	#N/A	#N/A	#N/A	7.2	2	4
G3X807		Histone H4 (Fragment)	10.949	54.1	6	14	#N/A	#N/A	#N/A
G5E582		Uncharacterized protein (Fragment)	11.65	#N/A	#N/A	#N/A	15.5	2	6
G5E5V7		Uncharacterized protein (Fragment)	34.008	#N/A	#N/A	#N/A	5.6	2	4
G5E604		Uncharacterized protein (Fragment)	11.058	28	3	4	28	2	6
G5E6Q3		Uncharacterized protein (Fragment)	37.121	2	1	1	#N/A	#N/A	#N/A
O02810-2		Isoform 2 of Phosphatidylinositol 4-kinase beta	89.802	3.9	3	3	1.5	1	2
O46385-2		Isoform 2 of Supervillin	200.62	11.3	17	25	6.2	12	22
O46563-2		Isoform Beta of V-type proton ATPase subunit H	54.117	11	4	4	3.7	2	6
O46606-2		Isoform 2 of Phospholipase DDHD1	92.968	1.6	2	2	3.6	3	4
O62830-2		Isoform Beta-2 of Protein phosphatase 1B	42.834	35.9	1	1	23.3	5	11

				Biological Replicate 1			Biological Replicate 2		
Accession number	Gene symbol	Discription	MW(kDa)	Sequence coverage(%)	Unique peptides	MS/MS count	Sequence coverage(%)	Unique peptides	MS/MS count
O77784-2		Isoform A of Isocitrate dehydrogenase [NAD] subunit beta, mitochondrial	42.134	#N/A	#N/A	#N/A	8.6	4	18
P00727-2		Isoform 2 of Cytosol aminopeptidase	54.036	4	2	3	#N/A	#N/A	#N/A
P04896-2		Isoform Gnas-2 of Guanine nucleotide-binding protein G(s)	44.28	5.3	1	1	5.5	1	3
P04973-2		Isoform Non-brain of Clathrin light chain A	23.308	32.4	8	18	26.3	8	25
P04975-2		Isoform Non-brain of Clathrin light chain B	23.073	#N/A	#N/A	#N/A	3.8	1	2
P07106-2		Isoform 2 of Acyl-CoA-binding domain-containing protein 5	58.352	2.5	1	1	2.1	1	1
P08037-2		Isoform Short of Beta-1,4-galactosyltransferase 1	43.483	#N/A	#N/A	#N/A	2.1	1	1
P08166-2		Isoform AK2B of Adenylate kinase 2, mitochondrial	25.633	47	10	19	22.2	5	21
P12234-2		Isoform B of Phosphate carrier protein, mitochondrial	40.003	39.9	13	36	14.1	6	18
P36225-3		Isoform 3 of Microtubule-associated protein 4	114.64	19.2	20	42	21.5	22	48
P48452-2		Isoform 2 of Serine/threonine-protein phosphatase 2B catalytic subunit alpha isoform	57.642	5.5	3	3	#N/A	#N/A	#N/A
P60661-2		Isoform Smooth muscle of Myosin light polypeptide 6	16.961	41.7	6	8	41.7	6	18
P68250-2		Isoform Short of 14-3-3 protein beta/alpha	27.849	60.2	6	18	25.4	2	6
P79136-2		Isoform Beta-2 of F-actin-capping protein subunit beta	30.628	56.2	19	44	40.4	13	33
P81282-4		Isoform V3 of Versican core protein	74.793	3	2	2	7.9	4	7
P98072-2		Isoform Short of Enteropeptidase	112.13	1.3	1	1	#N/A	#N/A	#N/A

				Biological Replicate 1			Biological Replicate 2		
Accession number	Gene symbol	Discription	MW(kDa)	Sequence coverage(%)	Unique peptides	MS/MS count	Sequence coverage(%)	Unique peptides	MS/MS count
Q05B54-2		Isoform 2 of Transmembrane protein 134	20.12	#N/A	#N/A	#N/A	8.3	1	1
Q08D83-2		Isoform 2 of Reticulon-3	25.496	15.2	4	10	8	2	13
Q0V7M7-2		Isoform 2 of Spindle and kinetochore-associated protein 1	26.213	#N/A	#N/A	#N/A	8.8	2	2
Q27966-2		Isoform 2 of Unconventional myosin-Ic	118.15	8.9	7	7	#N/A	#N/A	#N/A
Q29455-2		Isoform CSP2 of DnaJ homolog subfamily C member 5	18.815	#N/A	#N/A	#N/A	14.4	3	7
Q29463		Anionic trypsin	26.289	17	3	7	7.3	2	6
Q29466-2		Isoform 2 of V-type proton ATPase 116 kDa subunit a isoform 1	95.63	22.7	16	31	16.1	12	27
Q2KII7-2		Isoform 2 of Peroxisomal membrane protein PEX16	34.609	#N/A	#N/A	#N/A	7.9	2	5
Q32PC9-2		Isoform 2 of ADP-ribosylation factor-like protein 2-binding	17.589	5.9	1	2	5.9	1	2
Q3B7M2-2		Isoform 2 of Hydroxyacylglutathione hydrolase, mitochondrial	29.156	47.7	9	26	21.5	5	17
Q58DG1		UPF0160 protein MYG1, mitochondrial	42.967	36.2	10	28	6.6	2	3
Q5KR47-2		Isoform 2 of Tropomyosin alpha-3 chain	29.032	23	1	12	29	2	20
Q8HXY9-2		Isoform 2 of Craniofacial development protein 1	23.822	3.7	1	3	#N/A	#N/A	#N/A
Q9BGI1-2		Isoform Cytoplasmic+peroxisomal of Peroxiredoxin-5, mitochondrial	17.362	49.4	8	21	#N/A	#N/A	#N/A
Q9GKI7-2		Isoform 2 of Collagen type IV alpha-3-binding protein	68.076	11.2	6	8	4	3	5
Q9N1F0-2		Isoform 2 of Protein MRVI1	93.135	3.1	2	2	3.1	3	4
Q9N2J2-2		Isoform Cytoplasmic of Phospholipid hydroperoxide glutathione peroxidase, mitochondrial	19.436	52.9	9	23	35.9	6	16
Q9TU23-2		Isoform 2 of Centrosomal protein of 290 kDa	156.78	0.7	1	1	#N/A	#N/A	#N/A

Table S2. GO analysis of identified buffalo oocyte proteins.

Category	Term	Count	%	PValue	Genes	List Total	Pop Hits	Pop Total	Fold Enrichment	Bonferroni	Benjamini	FDR
GOTERM_BP_FAT	GO:0015031~protein transport	179	8.352776482	2.35E-40	P81623, Q0VCL3, Q71SS4, Q02399, Q3ZBM5, Q3SZN2, Q3T087, Q3ZBV2, Q08E32, Q3SZF2, Q0IIG8, P53620, A1A4I8, Q0IIG7, Q5E9Q4, Q2TBX8, P61763, Q24K11, Q32KR9, Q3T000, Q3T178, A6QR22, A5D7D1, Q1JQ98, Q2KIU0, Q2TBH9, Q28104, A5PKA5, P62261, P45478, Q17QF8, Q2TBH7, Q3ZBW4, A7YWD2, Q2T9P4, Q5E9Y2, P10948, Q3T093, Q3T0F5, Q17R06, Q3ZCH0, Q1RMR4, Q9GKZ4, Q5E9I6, Q3ZBT5, A1L567, Q2KJ37, A5PJF9, Q5E994, Q27954, Q3SX42, Q5E9A6, Q3SZD1, P35605, Q0P5N5, P35604, Q3ZBT1, Q5E9A1, Q58DF6, P63103, A6QR46, Q3ZBG1, A8E4N0, Q0VCB4, Q32KP9, P81127, P53619, Q0VCK5, Q3ZBN8, Q58DW6, P41541, Q08DS5, A3KN15, P50397, A6QR35, Q3MHP2, Q32PG1, A7YY75, Q58CS7, Q3MHG0, Q0G846, Q5EA68, Q2YDM7, A7MAZ2, Q29RT6, Q5E9L7, Q0II86, Q9XT96, Q2T9M1, Q08DD7, Q17QU4, Q3SWZ3, Q17QI5, P84081, A7Z057, Q32L81, A6QR54, Q5E971, P49951, Q3ZC32, Q2YDH6, A4FUG8, Q2KHV6, Q2HJE9, A6QLS9, Q3SZC1, A4IFL0, Q5EA77, Q58DR5, Q2T9U1, P11023, Q865S1, A6QLB1, A6QPK4, A6QPI6, Q148L0, P63009, Q3MHE8, Q3ZCC9, Q1LZB5, A2VDR8, Q5BIP4, Q6EWQ7, A1A4L0, Q3T054, Q2KII7, Q2KJ32, A5D7S0, Q5E9E8, A6QQL0, Q3SZB3, Q17QB7, Q08DB5, Q1RMV0, Q17QS1, A5PJZ5, A6H704, Q2KHX3, Q32KL9, Q3SZV6, Q2KHX4, Q3ZC13, Q32L63, Q1RMH8, Q3SYW1, Q0V8C2, Q2HJG5, P52193, C1JZ67, Q1RMQ3, Q5E9F1, Q0VD53, A6QLD1, Q17QC5, Q0V8S0, Q3T0U5, A3KN51, Q3SZ87, A4FV54, Q0P5F3, A6QNT8, Q3SZI7, Q148J4, Q3SZI4, Q2KJ81, A6QLL2, A0JN39, A2VE18, Q2TBS1, Q2HJH2, Q3T0D2, Q32KV6, Q3T0D7, P21856, P60519, Q58DS9, Q0V8B7, A2VE21, Q3SYV6, Q08DW1	1472	403	8785	2.650830389	7.21316E-37	7.21316E-37	4.24289E-37
GOTERM_BP_FAT	GO:0045184~establishment of protein localization	179	8.352776482	3.553E-40	P81623, Q0VCL3, Q71SS4, Q02399, Q3ZBM5, Q3SZN2, Q3T087, Q3ZBV2, Q08E32, Q3SZF2, Q0IIG8, P53620, A1A4I8, Q0IIG7, Q5E9Q4, Q2TBX8, P61763, Q24K11, Q32KR9, Q3T000, Q3T178, A6QR22, A5D7D1, Q1JQ98, Q2KIU0, Q2TBH9, Q28104, A5PKA5, P62261, P45478, Q17QF8, Q2TBH7, Q3ZBW4, A7YWD2, Q2T9P4, Q5E9Y2, P10948, Q3T093, Q3T0F5, Q17R06, Q3ZCH0, Q1RMR4, Q9GKZ4, Q5E9I6, Q3ZBT5, A1L567, Q2KJ37, A5PJF9, Q5E994, Q27954, Q3SX42, Q5E9A6, Q3SZD1, P35605, Q0P5N5, P35604, Q3ZBT1, Q5E9A1, Q58DF6, P63103, A6QR46, Q3ZBG1, A8E4N0, Q0VCB4, Q32KP9, P81127, P53619, Q0VCK5, Q3ZBN8, Q58DW6, P41541, Q08DS5, A3KN15, P50397, A6QR35, Q3MHP2, Q32PG1, A7YY75, Q58CS7, Q3MHG0, Q0G846, Q5EA68, Q2YDM7, A7MAZ2, Q29RT6, Q5E9L7, Q0II86, Q9XT96, Q2T9M1, Q08DD7, Q17QU4, Q3SWZ3, Q17QI5, P84081, A7Z057, Q32L81, A6QR54, Q5E971, P49951, Q3ZC32, Q2YDH6, A4FUG8, Q2KHV6, Q2HJE9, A6QLS9, Q3SZC1, A4IFL0, Q5EA77, Q58DR5, Q2T9U1, P11023, Q865S1, A6QLB1, A6QPK4, A6QPI6, Q148L0, P63009, Q3MHE8, Q3ZCC9, Q1LZB5, A2VDR8, Q5BIP4, Q6EWQ7, A1A4L0, Q3T054, Q2KII7, Q2KJ32, A5D7S0, Q5E9E8, A6QQL0, Q3SZB3, Q17QB7, Q08DB5, Q1RMV0, Q17QS1, A5PJZ5, A6H704, Q2KHX3, Q32KL9, Q3SZV6, Q2KHX4, Q3ZC13, Q32L63, Q1RMH8, Q3SYW1, Q0V8C2, Q2HJG5, P52193, C1JZ67, Q1RMQ3, Q5E9F1, Q0VD53, A6QLD1, Q17QC5, Q0V8S0, Q3T0U5, A3KN51, Q3SZ87, A4FV54, Q0P5F3, A6QNT8, Q3SZI7, Q148J4, Q3SZI4, Q2KJ81, A6QLL2, A0JN39, A2VE18, Q2TBS1, Q2HJH2, Q3T0D2, Q32KV6, Q3T0D7, P21856, P60519, Q58DS9, Q0V8B7, A2VE21, Q3SYV6, Q08DW1	1472	404	8785	2.644268928	1.09053E-36	5.45263E-37	6.41464E-37
GOTERM_BP_FAT	GO:0008104~protein localization	189	8.819412039	3.749E-39	P81623, Q0VCL3, Q71SS4, Q02399, Q3ZBM5, Q3SZN2, Q3T087, Q3ZBV2, Q08E32, Q3SZF2, Q0IIG8, P53620, A1A4I8, Q0IIG7, Q5E9Q4, Q2TBX8, Q08DR9, P61763, Q24K11, Q32KR9, Q3T000, Q3T178, A6QR22, A5D7D1, Q1JQ98, Q2KIU0, Q2TBH9, Q28104, A5PKA5, P62261, Q32LP2, P45478, Q17QF8, Q2TBH7, Q3ZBW4, A7YWD2, Q2T9P4, Q5E9Y2, P10948, Q3T093, Q3T0F5, Q17R06, A6QR14, Q3ZCH0, Q1RMR4, Q3MHI2, Q9GKZ4, Q5E9I6, Q3ZBT5, A1L567, Q3MHX6, Q2KJ37, A5PJF9, Q5E994, Q27954, Q3SX42, Q5E9A6, Q3SZD1, P35605, Q0P5N5, P35604, Q3ZBT1, Q5E9A1, Q58DF6, P63103, A6QR46, Q3ZBG1, A8E4N0, Q0VCB4, Q32KP9, P81127, Q0VCX4, P53619, Q0VCK5, Q3ZBN8, Q58DW6, P41541, Q08DS5, A3KN15, P50397, A6QR35, Q3MHP2, Q32PG1, A7YY75, Q58CS7, Q3MHG0, Q0G846, Q5EA68, Q2YDM7, A7MAZ2, Q29RT6, Q5E9L7, Q0II86, Q9XT96, Q2T9M1, Q08DD7, Q17QU4, Q3SWZ3, Q17QI5, P84081, A7Z057, Q32L81, A6QR54, Q5E971, P49951, Q3ZC32, Q2YDH6, Q2KHV6, A4FUG8, Q2HJE9, A6QLS9, Q3SZC1, A4IFL0, Q5EA77, Q58DR5, Q2T9U1, P11023, Q865S1, A6QLB1, A6QPK4, A6QPI6, Q148L0, P63009, Q3MHE8, Q3ZCC9, Q1LZB5, A2VDR8, Q5BIP4, Q6EWQ7, A1A4L0, Q3T054, Q2KII7, Q2KJ32, A5D7S0, Q5E9E8, A6QQL0, Q3SZB3, Q17QB7, Q08DB5, Q1RMV0, Q17QS1, A5PJZ5, A6H704, Q32KL9, Q2KHX3, Q3SZV6, Q32LM0, Q2KHX4, Q3ZC13, Q32L63, Q1RMH8, Q3SYW1, Q0V8C2, Q2HJG5, C1JZ67, P52193, Q1RMQ3, Q5E9F1, Q0VD53, Q0VCT3, A6QLD1, Q17QC5, Q0V8S0, Q3T0U5, A3KN51, Q3SZ87, A4FV54, Q0P5F3, A6QNT8, Q3SZI7, Q148J4, Q3SZI4, Q2KJ81, A6QLL2, A0JN39, A2VE18, Q2TBS1, Q2HJH2, Q3T0D2, Q32KV6, P79132, Q3T0D7, P21856, P60519, Q58DS9, Q0V8B7, A2VE21, Q3SYV6, O02703, Q08DW1	1472	446	8785	2.529070299	1.15057E-35	3.83525E-36	6.76786E-36
GOTERM_BP_FAT	GO:0006412~translation	140	6.532897807	9.107E-38	P82670, Q95140, Q3ZBV8, Q58DV0, Q3MIC0, A7YW98, Q3T087, Q2KJG3, Q3SZX5, P39872, Q2KHZ2, Q56JV9, Q3MHH4, A7MBC5, Q9XSI3, Q3SZ65, Q56JV1, Q861S4, Q3SYU2, A8E4P2, P68103, P68102, P63048, P49410, Q3T003, Q3T0X6, Q3ZBL5, Q5E9B4, Q5EAD6, Q3T102, Q3T099, Q0VC30, A6QLG5, Q3SZ54, Q0IIF2, P82931, P61257, Q3T0W9, P82924, Q5E995, Q3T116, Q3T058, Q3T057, Q3T122, Q9TU47, A6QPQ5, A7MB16, Q1RMU2, Q95L46, Q0VCX5, P17248, P82915, Q58DW0, Q58DW5, P82916, A5PK63, Q5E983, O18789, A6H7E1, Q5E988, Q3T076, Q3T0R1, Q3SYR7, A7MBD4, Q6B860, Q76I82, Q76I81, Q9N0T5, P61356, Q32PD5, P62276, Q56JZ1, Q32LC3, A4FUC8, Q5E973, Q2KHU8, Q56JZ5, P62992, A6H769, Q5E9D0, P43896, P61284, Q0P5H7, Q32LJ9, Q56K10, Q5E966, Q3T0S6, Q3T148, Q56K14, Q3MHM7, Q3ZBH8, P26452, Q6EWQ7, Q862I1, Q56JY1, Q58DQ3, Q32LK1, Q3SZ90, Q3T0B7, A3KN48, A6QLR2, A5D7G0, P79103, Q3T051, Q0VCQ4, Q5E9E6, Q2T9L8, A6QNM8, Q3SZV3, Q3ZCK1, A6QNM2, Q58DT1, A5D7A2, Q2TBR2, Q2KIF8, Q0II26, Q0II31, Q56JX6, Q3SZ85, Q56JX3, A6QR01, Q3SYW6, P42899, Q56JX8, Q9GMB8, Q3T169, Q3B7M5, Q3T025, Q3SZQ6, Q3T0D5, Q2KI84, Q2TBS2, Q0VCU8, A6QLT9, Q3T0L7, Q3T0V4, Q3T171, Q24JY1, Q3T0V3	1472	283	8785	2.952402443	2.79507E-34	6.98768E-35	1.6441E-34
GOTERM_BP_FAT	GO:0006091~generation of precursor metabolites and energy	110	5.132991134	3.2E-32	Q95KV7, Q5W5U3, Q32LG3, P35720, Q6QTG5, A7MB35, Q3T0G8, P00171, Q0IIG5, Q3B7M9, Q05752, P32876, Q3SZ62, P52505, P23935, P23934, Q0VCM4, Q5E9B1, P10096, A5PJN2, P00157, P33097, Q05B87, Q9XSJ4, Q3T189, A5PJF9, Q3MHX5, Q3ZC09, O77784, Q05B51, Q3T0P6, P11019, Q9XSG3, A2VE47, P31408, Q2KJE5, Q148D3, Q2TBV3, P31404, P13271, P34943, Q85BD6, P34942, P31800, Q6QTH0, O97725, P10575, Q148N0, P00829, P11966, Q01321, P61420, P04394, Q29RK1, O97681, Q9MYM4, Q58DR8, Q02365, P23004, P61287, Q3T145, Q0Z7W6, P40682, P05631, P05630, Q8HXG5, Q02827, P19858, Q8HXG6, P21282, P17694, P11179, A5D9A7, P20004, P00125, Q95123, P42029, P42028, P13619, A5D984, P15690, Q2KJB6, P00129, Q5E956, Q3SZP5, Q00361, Q3T0E7, Q58D96, Q02380, P13620, P13621, A1A4J1, P00432, P68530, Q02367, Q02366, Q02369, Q02368, A6QLL8, Q02373, P48305, Q02375, Q2KIG0, Q02371, Q0VCU1, Q02372, Q3ZBY4, A7MB78, Q28852, P25708	1472	211	8785	3.111316454	9.82124E-29	1.96425E-29	5.77701E-29
GOTERM_BP_FAT	GO:0046907~intracellular transport	111	5.17965469	4.737E-20	Q02399, Q71SS4, Q3SZN2, Q3T087, Q0P5L6, A1A4I8, P53620, Q08DH4, Q24K11, Q3T000, Q2KIU0, Q1JQ98, Q28104, Q2TBP1, P62261, A2VDN5, A7E2Z6, P79345, Q3ZBW4, A7YWD2, Q5E9Y2, Q08DQ1, Q5E9R3, Q3T0F2, Q3ZCH0, Q3ZBT5, A1L567, Q27954, Q0P5N5, P35605, A2VDK6, Q3SZD1, P35604, Q3ZBT1, P63103, A8E4N0, P81127, Q32KP9, P53619, Q0VCK5, Q3ZBU5, P41541, A5PJ65, A3KN15, Q32PG1, Q0G846, Q5EA68, Q2YDM7, A7MAZ2, Q17QI1, Q5E9L7, Q2T9M1, Q3SWZ3, Q17QI5, A7Z057, Q32L81, Q5E971, P49951, Q3ZC32, Q2YDH6, Q2HJE9, Q3SZC1, A4IFL0, A6H7H2, Q2T9U1, P11023, P23356, A6QLB1, Q865S1, P57709, A6QPK4, Q56K10, P63009, Q3MHE8, Q5E9M9, Q1LZB5, A2VDR8, Q5BIP4, Q2TBL9, Q6EWQ7, Q3T054, Q2HJF8, Q2KII7, A5D7S0, Q3SX02, Q1RMV0, Q08DB5, Q32KL9, Q3ZC13, Q3SYW1, P52193, C1JZ67, Q5E9F1, Q0VD53, Q17QC5, Q0V8S0, Q3SZ87, Q3T0M7, A4FV54, A6QNT8, Q2KJ81, Q3SZI4, A6QLL2, A0JN39, A2VE18, Q2TBS1, Q3T0D7, P60519, Q0V8B7, A2VE21, Q3SYV6, O02703	1472	281	8785	2.357494101	1.45368E-16	2.4228E-17	8.55076E-17

Category	Term	Count	%	PValue	Genes	List Total	Pop Hits	Pop Total	Fold Enrichment	Bonferroni	Benjamini	FDR
GOTERM_BP_FAT	GO:0055114~oxidation reduction	167	7.792813812	1.498E-19	Q32LG3, Q95KV7, Q3ZBV9, Q2KHZ9, P00366, Q2NL01, Q0P5L5, Q2KJH7, P35720, Q6QTG5, A7MB35, Q3T0G8, P00171, Q2T9Y6, Q2KIL4, Q05752, Q3ZBE9, P52505, P23935, P23934, Q3ZCI4, Q5E9H7, Q3ZBW6, Q5E9B1, P10096, A5PJN2, Q8WMV1, P00157, Q5EAD4, A5D7K0, Q9BGI2, Q3SYT8, P16116, Q9BGI3, Q9BGI1, A5PJF6, Q1JPJ0, Q3T189, P41976, Q5E936, A5PJF9, Q3SZD7, Q5I598, P35705, P48818, O77784, Q0P5N4, Q05B51, A7YWE4, Q3T067, Q9XSG3, Q7YS70, Q3MHF7, Q17QW3, Q2KJE5, Q2TBV3, Q1RMU3, P13271, Q1JQD0, Q5E9J5, Q85BD6, P34943, Q59A28, P34942, Q0IIL6, P31800, Q3T0Z7, Q6QTH1, P52556, Q6QTH0, P31228, O97725, P10575, Q148N0, A6QQR7, O77834, Q0P5C2, Q0VC97, P11966, Q01321, Q2HJD7, Q5E9K3, Q0P5H1, Q3T0R4, Q3SZJ4, Q58D31, Q17QU7, A6QQT4, P04394, A5D9A8, Q07536, Q02365, A6H7H3, P23004, Q3SWY3, P11024, Q3T145, Q0Z7W6, Q3SZ00, Q148L6, A6H7G3, Q8HXG5, P19858, Q02827, Q3SZU4, Q8HXG6, Q32L99, Q3ZC42, P17694, P11178, A5D9A7, A4FUF0, O46414, P00125, O46415, P21839, P42029, Q95123, Q6B4J2, P42028, P20000, P15690, Q3SZB4, Q08DM3, Q3ZCD7, P00129, P07514, Q17QK8, Q0IIB1, P00442, Q3T0T9, O02691, Q3SZP5, A6QLU8, Q58DT4, Q0VCN1, Q58D96, Q0NXR6, P48644, O97764, Q02380, A0JNA3, Q9N2I8, Q17QC2, P00376, Q0VC53, P00435, P00432, Q3ZCJ2, Q5E947, P68530, Q02367, Q1JPD3, Q02366, Q02369, Q02368, Q3SZI8, Q02373, P48305, Q02375, Q58CW1, Q2KIG0, Q58D08, Q02371, Q02372, Q865R1, Q3ZBY7, P25708	1472	513	8785	1.94282222	4.59712E-16	6.56732E-17	2.7041E-16
GOTERM_BP_FAT	GO:0022900~electron transport chain	51	2.379841344	5.383E-18	Q95KV7, P35720, P04394, Q6QTG5, Q3T0G8, P00171, Q02365, Q05752, P52505, P23004, P23935, P23934, Q0Z7W6, A5PJN2, Q8HXG5, Q02827, P00157, Q8HXG6, P17694, A5D9A7, P00125, P42029, Q95123, P42028, P15690, Q3T189, P00129, Q05B51, Q2TBV3, Q02380, P13271, Q85BD6, P34943, P34942, P31800, P68530, Q6QTH0, Q02367, Q02366, Q02369, Q02368, O97725, P10575, Q02373, P48305, Q02375, Q2KIG0, Q02371, Q02372, Q01321, P25708	1472	87	8785	3.498524175	1.6519E-14	2.06488E-15	9.71674E-15
GOTERM_BP_FAT	GO:0016192~vesicle-mediated transport	97	4.526364909	2.917E-16	Q2YDD3, Q3SZN2, Q3T086, Q0P5L6, P83939, Q3SZF2, Q5E9Q4, Q0IIG7, P53620, Q3T0G8, Q08DZ3, Q5EAE0, Q2TBX8, Q24K11, P61763, Q3T000, Q1JQ98, Q28104, Q2YDL1, A2VDN5, P45478, Q17QF8, Q5E9Y2, Q3T093, Q5E9R3, Q3T0F2, Q27991, Q5E9I6, Q2KJ37, Q3ZBT5, Q27954, P35605, A2VDK6, Q0IIE6, P35604, A6QR46, A8E4N0, P81127, Q0VCX4, P53619, Q0VCK5, P41541, A5PJ65, Q3T0Q4, A3KN15, Q32PG1, A7MAZ2, Q17QI1, Q3SWZ3, Q17QI5, P84081, Q5E971, P49951, Q3ZC32, Q2YDH6, A4FUG8, P80746, Q3T0C9, Q95114, Q5EA77, P11023, A6QLB1, Q865S1, P62998, P63009, A2VDR8, Q2TBL9, A5D7S0, Q08E63, Q5E9E8, A6H7G2, A7Z035, Q08DB5, A5D7A2, Q32KL9, Q3ZC13, Q0V8C2, Q3SYW1, Q0VD53, A6QLD1, Q17QC5, Q2KJA1, A4FV54, Q0P5F3, A6QNT8, A5D7R9, Q2KJ81, A0JN38, A5D794, A0JN39, P52174, Q32L78, P52175, A2VE18, Q3T0D2, Q3T0D7, P60519, A2VE21, Q3SYV4	1472	254	8785	2.279145092	1.02218E-12	1.13576E-13	5.9952E-13
GOTERM_BP_FAT	GO:0070727~cellular macromolecule localization	86	4.013065796	6.788E-16	Q5E9L7, Q2T9M1, Q02399, Q71SS4, Q3SWZ3, A7Z057, Q3SZN2, Q32L81, Q3T087, P49951, Q3ZC32, Q2YDH6, A5D7H5, A1A4I8, Q2HJE9, P53620, Q3SZC1, Q08DR9, A4IFL0, Q24K11, Q2T9U1, Q2KIU0, Q1JQ98, P11023, A6QLB1, Q865S1, P62261, A6QPK4, P63009, Q3MHE8, Q1LZB5, Q3ZBW4, A2VDR8, A7YWD2, Q5BIP4, Q5E9Y2, Q6EWQ7, Q3T054, Q2KII7, A5D7S0, Q3ZCH0, Q3ZBT5, A1L567, Q08DB5, Q1RMV0, Q27954, P35605, Q0P5N5, Q3SZD1, P35604, Q3ZBT1, Q32KL9, Q32LM0, Q3ZC13, P63103, Q3SYW1, C1JZ67, P52193, Q5E9F1, Q0VCT3, Q17QC5, A8E4N0, Q0V8S0, P81127, Q3SZ87, Q32KP9, A6QNT8, Q0VCX4, P53619, Q0VCK5, Q2KJ81, Q3SZI4, A6QLL2, P41541, A0JN39, A2VE18, Q2TBS1, A3KN15, Q32PG1, Q3T0D7, Q0V8B7, A2VE21, Q3SYV6, Q0G846, O02703, Q5EA68, A7MAZ2	1472	215	8785	2.387228261	2.04436E-12	2.04392E-13	1.19904E-12
GOTERM_BP_FAT	GO:0034613~cellular protein localization	85	3.96640224	1.56E-15	Q5E9L7, Q2T9M1, Q02399, Q71SS4, Q3SWZ3, A7Z057, Q3SZN2, Q32L81, Q3T087, P49951, Q3ZC32, Q2YDH6, A1A4I8, Q2HJE9, P53620, Q3SZC1, Q08DR9, A4IFL0, Q24K11, Q2T9U1, Q2KIU0, Q1JQ98, P11023, A6QLB1, Q865S1, P62261, A6QPK4, P63009, Q3MHE8, Q1LZB5, Q3ZBW4, A2VDR8, A7YWD2, Q5BIP4, Q5E9Y2, Q6EWQ7, Q3T054, Q2KII7, A5D7S0, Q3ZCH0, Q3ZBT5, A1L567, Q08DB5, Q1RMV0, Q27954, P35605, Q0P5N5, Q3SZD1, P35604, Q3ZBT1, Q32KL9, Q32LM0, Q3ZC13, P63103, Q3SYW1, C1JZ67, P52193, Q5E9F1, Q0VCT3, Q17QC5, A8E4N0, Q0V8S0, P81127, Q3SZ87, Q32KP9, A6QNT8, Q0VCX4, P53619, Q0VCK5, Q2KJ81, Q3SZI4, A6QLL2, P41541, A0JN39, A2VE18, Q2TBS1, A3KN15, Q32PG1, Q3T0D7, Q0V8B7, A2VE21, Q3SYV6, Q0G846, O02703, Q5EA68, A7MAZ2	1472	214	8785	2.370495352	4.77018E-12	4.33653E-13	2.80886E-12
GOTERM_BP_FAT	GO:0006886~intracellular protein transport	80	3.733084461	7.523E-15	Q5E9L7, Q2T9M1, Q02399, Q71SS4, Q3SWZ3, A7Z057, Q3SZN2, Q32L81, Q3T087, P49951, Q3ZC32, Q2YDH6, A1A4I8, Q2HJE9, P53620, Q3SZC1, A4IFL0, Q24K11, Q2T9U1, Q2KIU0, Q1JQ98, P11023, A6QLB1, Q865S1, P62261, A6QPK4, P63009, Q3MHE8, Q1LZB5, Q3ZBW4, A2VDR8, A7YWD2, Q5BIP4, Q5E9Y2, Q6EWQ7, Q3T054, Q2KII7, A5D7S0, Q3ZCH0, Q3ZBT5, A1L567, Q08DB5, Q1RMV0, Q27954, Q0P5N5, P35605, Q3SZD1, P35604, Q3ZBT1, Q32KL9, Q3ZC13, Q3SYW1, P63103, C1JZ67, P52193, Q5E9F1, Q17QC5, A8E4N0, Q0V8S0, P81127, Q3SZ87, Q32KP9, A6QNT8, P53619, Q0VCK5, Q2KJ81, Q3SZI4, A6QLL2, P41541, A0JN39, A2VE18, Q2TBS1, A3KN15, Q32PG1, Q3T0D7, Q0V8B7, A2VE21, Q3SYV6, Q0G846, Q5EA68, A7MAZ2	1472	200	8785	2.387228261	2.31695E-11	1.93079E-12	1.36335E-11
GOTERM_BP_FAT	GO:0051186~cofactor metabolic process	57	2.659822678	1.567E-14	Q17QE6, Q32LG3, Q3T186, P35720, Q29RK1, Q58DR8, Q2T9Y6, Q2KIL4, Q4JIJ3, Q3ZCI4, Q3SZG8, Q3T145, P45478, Q3ZBS0, Q2KJF3, Q05B87, A5D7K0, P11179, A4FUF0, P20004, Q95123, Q24K00, Q6B4J2, Q0P5A2, Q5BIP7, Q3T189, Q3SZD7, Q3MHX5, Q2TBG7, P00442, O77784, Q5E956, Q0VCA7, Q2KIN5, Q32PJ6, Q58DK5, Q1JQD7, Q9XSG3, Q58D96, Q0II59, Q148D3, A4FV58, A6QR05, Q2NL34, P00435, Q5EAC2, P22600, Q32S38, Q3T131, Q58D49, A5PJL7, Q58CW1, A4FUD0, Q5E9T4, Q0P5C2, Q0VCU1, Q2HJD7	1472	120	8785	2.83483356	4.80426E-11	3.6956E-12	2.82552E-11
GOTERM_BP_FAT	GO:0045333~cellular respiration	32	1.493233784	1.486E-13	Q32LG3, Q3MHX5, O77784, P35720, Q6QTG5, Q29RK1, Q3T0G8, Q9XSG3, Q58D96, Q58DR8, Q148D3, Q05752, P52505, P23935, Q3T145, Q85BD6, P31800, P00432, P00157, Q02827, P68530, Q6QTH0, Q05B87, P11179, Q02375, P20004, Q95123, Q0VCU1, Q02372, P15690, Q3T189, P00129	1472	48	8785	3.978713768	4.55893E-10	3.25638E-11	2.68163E-10
GOTERM_BP_FAT	GO:0015980~energy derivation by oxidation of organic compounds	39	1.819878675	3.908E-13	Q32LG3, Q3MHX5, O77784, P35720, Q6QTG5, Q29RK1, Q9XSG3, Q3T0G8, Q3T0E7, Q9MYM4, Q58D96, Q58DR8, Q148D3, Q3B7M9, Q05752, P52505, P61287, P23935, Q3T145, Q85BD6, Q0VCM4, P31800, P00432, P00157, Q02827, P68530, Q6QTH0, P33097, Q05B87, P11179, Q02375, P20004, Q95123, Q0VCU1, Q02372, P15690, A7MB78, Q3T189, P00129	1472	69	8785	3.373257325	1.19936E-09	7.99574E-11	7.0548E-10
GOTERM_BP_FAT	GO:0006457~protein folding	54	2.519832011	8.285E-13	Q2TBI4, Q32KS0, A1A4M8, Q3MHL7, P30404, P61603, Q3SZC1, Q3ZBA6, Q3T0K2, Q3ZCI9, Q2TBX2, P19120, Q0P5D0, Q8HYI9, Q2KJC8, A7Z066, Q2HJ94, A5PJN2, Q3ZBH0, Q3SYT6, P48427, Q3ZCH0, A1A4P5, Q3SZ99, Q5E992, A5PK75, P80311, Q5E954, Q0P5N5, Q5E951, Q32PA9, Q0IIM3, Q32LM0, Q3MI00, Q29455, Q17Q89, P52193, P26884, Q0III6, Q32P97, Q95M18, P26882, Q9TRY0, Q76LV1, Q3SZE9, Q3SZE2, Q76LV2, P62935, Q5BIN5, A6QLT9, Q3T0L2, Q0G846, P18203, A1A4J9	1472	119	8785	2.708200128	2.54251E-09	1.58907E-10	1.49555E-09
GOTERM_BP_FAT	GO:0044265~cellular macromolecule catabolic process	95	4.433037797	2.149E-11	Q9XT96, Q1RMX2, Q0VCL3, Q71SS4, A6QR55, Q2YDD2, Q3SX24, A1L506, Q3ZBV3, Q0VCP5, A7MBJ5, Q2KJG2, Q29RK4, A5D7H5, Q58DH8, Q2TBX8, Q0VD31, A3KMV5, Q2HJE4, P56541, O41515, A3KMV3, Q3SZC2, P23356, Q58CZ0, Q3T0X5, P45478, P33672, Q3T102, Q0IIF7, Q3T108, Q0P594, Q9XSJ0, Q2KII4, Q0VC37, Q29463, Q2TBG8, Q3SZ52, Q2NL22, Q08DY9, Q3MHN0, Q58DU5, Q5E9K0, Q3MHX6, Q0IIM6, A3KN22, A6QNM7, Q32KL2, A1A4K3, P00442, Q3T0Y5, Q32PA5, Q5E953, Q0P568, Q2T9S7, Q3ZBT1, Q3ZCK9, P62194, Q3T112, Q58DG6, Q3SZ43, Q3ZBG0, P38447, Q32PF3, Q5E9J6, P61955, Q1RMU2, A6H744, Q2KIJ6, Q5E9F9, Q10741, Q95M18, Q5E987, A6QPH1, A4IFN7, Q8MJJ7, A4FV12, Q3T168, Q0P5K3, Q1RMW1, A2VE14, Q3T0Z3, A2VDP1, Q32LN1, P61085, Q2KIK0, Q3ZCF7, A2VE20, Q32PG3, Q3ZCF3, Q59HJ6, O02703, Q08DE9, Q1LZE1, A2VEA3	1472	290	8785	1.955057627	6.59604E-08	3.88002E-09	3.87989E-08

Category	Term	Count	%	PValue	Genes	List Total	Pop Hits	Pop Total	Fold Enrichment	Bonferroni	Benjamini	FDR
GOTERM_BP_FAT	GO:0009057~macromolecule catabolic process	103	4.806346244	3.785E-11	Q0VCL3, Q02399, Q71SS4, Q2YDD2, Q3ZBV3, A7MBJ5, Q2KJG2, Q2TBX8, O41515, P56541, A3KMV5, A3KMV3, Q3T0X5, P45478, A7E2Z6, Q3T102, Q2KJI7, Q0IIF7, Q3T108, Q0P594, Q9XSJ0, Q0VC37, A6QR14, Q2TBG8, A6QR12, Q2NL22, Q3SZ52, Q08DY9, Q58DU5, Q5E9K0, Q3MHX6, Q0IIM6, A3KN22, Q3T0Y5, Q3ZBT1, Q2T9S7, P62194, Q3T112, Q58DG6, Q3SZ43, Q3ZBG0, Q32PF3, Q5E9J6, P61955, Q1RMU2, A6H744, Q2KIJ6, Q10741, Q95M18, A4IFN7, A6QPH1, Q5E987, A4FV12, Q3T0Z3, P61085, Q2KIK0, Q3ZCF7, Q32PG3, Q3ZCF3, Q08DE9, Q1LZE1, Q9XT96, Q1RMX2, A6QR55, Q3SX24, A1L506, Q0VCP5, Q29RK4, A5D7H5, Q58DH8, Q9MYM4, Q0VD31, Q2HJE4, P23356, Q3SZC2, Q58CZ0, P33672, Q2KII4, Q08E63, Q29463, Q3MHN0, A6QNM7, Q32KL2, P00442, A1A4K3, Q32PA5, Q5E953, Q0P568, Q3ZCK9, Q5EAB4, P38447, Q5E9F9, Q8MJJ7, Q3T168, Q0P5K3, Q1RMW1, A2VE14, A2VDP1, Q32LN1, A2VE20, Q59HJ6, O02703, A2VEA3	1472	327	8785	1.879851001	1.16151E-07	6.45285E-09	6.8322E-08
GOTERM_BP_FAT	GO:0007005~mitochondrion organization	35	1.633224452	3.829E-10	Q0II87, Q0VCA3, Q1RMV0, P35705, Q32L81, A6QNM2, Q0P5N5, A0JNK3, Q32LM0, Q32LE4, Q3T0G8, Q2HJI2, Q3SZC1, Q3MHF7, A5D9D4, P11023, Q5E9H5, P82922, Q0P5F4, A6QPI6, P00435, A7E2Z6, Q8WMV3, A6QLL2, Q1LZB5, Q0VC36, Q2TBS1, Q76LV2, Q05B87, Q02375, P42028, Q0G846, O02703, Q08DY9, A4FUH5, Q3SZB3	1472	69	8785	3.027282215	1.17504E-06	6.18445E-08	6.9118E-07
GOTERM_BP_FAT	GO:0048193~Golgi vesicle transport	22	1.026598227	2.019E-09	A2VDN5, Q17QI1, P53619, Q17QI5, Q27954, Q3SZN2, A5PJ65, Q5E971, A2VDR8, P35605, P35604, Q0P5L6, Q3ZC32, A2VE18, Q2TBL9, P53620, P60519, Q3T0F2, Q3T000, A4FV54, Q28104, A6QNT8	1472	33	8785	3.978713768	6.19713E-06	3.09857E-07	3.64526E-06
GOTERM_BP_FAT	GO:0044257~cellular protein catabolic process	80	3.733084461	2.23E-09	Q9XT96, Q1RMX2, Q0VCL3, Q71SS4, A6QR55, Q3SX24, A1L506, Q0VCP5, A7MBJ5, Q2KJG2, Q29RK4, Q2TBX8, Q0VD31, A3KMV5, Q2HJE4, O41515, A3KMV3, P23356, Q3SZC2, Q3T0X5, P45478, P33672, Q0IIF7, Q3T108, Q0P594, Q9XSJ0, Q2KII4, Q29463, Q2TBG8, Q3SZ52, Q58DU5, Q3MHN0, Q5E9K0, Q3MHX6, Q0IIM6, A3KN22, A6QNM7, Q32KL2, A1A4K3, Q3T0Y5, Q32PA5, Q5E953, Q0P568, Q3ZBT1, Q2T9S7, P62194, Q3ZCK9, Q3T112, Q58DG6, Q3SZ43, Q3ZBG0, Q32PF3, Q5E9J6, P61955, Q1RMU2, A6H744, Q2KIJ6, Q5E9F9, Q10741, Q95M18, Q5E987, A4IFN7, A4FV12, Q3T168, Q0P5K3, Q1RMW1, A2VE14, Q3T0Z3, A2VDP1, Q32LN1, P61085, Q2KIK0, Q3ZCF7, A2VE20, Q32PG3, Q3ZCF3, Q59HJ6, Q08DE9, Q1LZE1, A2VEA3	1472	248	8785	1.925184081	6.84486E-06	3.25947E-07	4.02627E-06
GOTERM_BP_FAT	GO:0006732~coenzyme metabolic process	41	1.913205786	2.794E-09	Q32LG3, Q3MHX5, Q3T186, P00442, O77784, Q5E956, Q0VCA7, P35720, Q29RK1, Q9XSG3, Q1JQD7, Q58D96, Q0II59, Q58DR8, Q2T9Y6, Q148D3, Q2KIL4, Q4JIJ3, A6QR05, Q2NL34, Q3ZCI4, Q3T145, P00435, Q3ZBS0, Q5EAC2, P11179, Q32S38, Q3T131, A4FUF0, A5PJL7, P20004, Q58CW1, Q95123, A4FUD0, Q5E9T4, Q0P5C2, Q0VCU1, Q5BIP7, Q0P5A2, Q3T189, Q2HJD7	1472	94	8785	2.603094646	8.57466E-06	3.89759E-07	5.04377E-06
GOTERM_BP_FAT	GO:0030163~protein catabolic process	84	3.919738684	3.906E-09	Q9XT96, Q1RMX2, Q0VCL3, Q71SS4, A6QR55, Q3SX24, A1L506, Q0VCP5, A7MBJ5, Q2KJG2, Q29RK4, Q2TBX8, Q0VD31, A3KMV5, Q2HJE4, O41515, A3KMV3, P23356, Q3SZC2, Q3T0X5, P45478, P33672, A7E2Z6, Q2KJI7, Q0IIF7, Q3T108, Q0P594, Q9XSJ0, Q2KII4, Q08E63, Q29463, Q2TBG8, A6QR12, Q3SZ52, Q3MHN0, Q58DU5, Q5E9K0, Q3MHX6, Q0IIM6, A3KN22, A6QNM7, Q32KL2, A1A4K3, Q3T0Y5, Q32PA5, Q5E953, Q0P568, Q3ZBT1, Q2T9S7, Q3ZCK9, P62194, Q3T112, Q58DG6, Q3SZ43, Q3ZBG0, Q32PF3, Q5E9J6, P61955, Q1RMU2, A6H744, Q2KIJ6, Q5E9F9, Q10741, Q95M18, Q5E987, A4IFN7, A4FV12, Q3T168, Q0P5K3, Q1RMW1, A2VE14, Q3T0Z3, A2VDP1, Q32LN1, P61085, Q2KIK0, Q3ZCF7, A2VE20, Q32PG3, Q3ZCF3, Q59HJ6, Q08DE9, Q1LZE1, A2VEA3	1472	268	8785	1.870589309	1.19865E-05	5.21157E-07	7.05071E-06
GOTERM_BP_FAT	GO:0051603~proteolysis involved in cellular protein catabolic process	79	3.686420905	4.394E-09	Q9XT96, Q1RMX2, Q0VCL3, Q71SS4, A6QR55, Q3SX24, A1L506, Q0VCP5, A7MBJ5, Q2KJG2, Q29RK4, Q2TBX8, Q0VD31, A3KMV5, Q2HJE4, O41515, A3KMV3, P23356, Q3SZC2, Q3T0X5, P33672, Q0IIF7, Q3T108, Q0P594, Q9XSJ0, Q2KII4, Q29463, Q2TBG8, Q3SZ52, Q58DU5, Q3MHN0, Q5E9K0, Q3MHX6, Q0IIM6, A3KN22, A6QNM7, Q32KL2, A1A4K3, Q3T0Y5, Q32PA5, Q5E953, Q0P568, Q3ZBT1, Q2T9S7, P62194, Q3ZCK9, Q3T112, Q58DG6, Q3SZ43, Q3ZBG0, Q32PF3, Q5E9J6, P61955, Q1RMU2, A6H744, Q2KIJ6, Q5E9F9, Q10741, Q95M18, Q5E987, A4IFN7, A4FV12, Q3T168, Q0P5K3, Q1RMW1, A2VE14, Q3T0Z3, A2VDP1, Q32LN1, P61085, Q2KIK0, Q3ZCF7, A2VE20, Q32PG3, Q3ZCF3, Q59HJ6, Q08DE9, Q1LZE1, A2VEA3	1472	247	8785	1.90881612	1.34843E-05	5.6185E-07	7.93173E-06
GOTERM_BP_FAT	GO:0006007~glucose catabolic process	26	1.21325245	6.184E-09	A5PJF9, Q32LG3, Q5W5U3, Q3T186, Q3ZC09, Q5E956, Q3T0P6, A7MB35, A2VE47, Q0IIG5, Q2KJE5, Q3SZ62, Q3ZCI4, Q3T145, P10096, Q5E9B1, A1A4J1, P19858, A6QLL8, Q148N0, A4FUF0, Q9XSJ4, Q3ZBY4, A5D984, P11966, Q2HJD7	1472	46	8785	3.373257325	1.89782E-05	7.59135E-07	1.11634E-05
GOTERM_BP_FAT	GO:0019320~hexose catabolic process	26	1.21325245	1.112E-08	A5PJF9, Q32LG3, Q5W5U3, Q3T186, Q3ZC09, Q5E956, Q3T0P6, A7MB35, A2VE47, Q0IIG5, Q2KJE5, Q3SZ62, Q3ZCI4, Q3T145, P10096, Q5E9B1, A1A4J1, P19858, A6QLL8, Q148N0, A4FUF0, Q9XSJ4, Q3ZBY4, A5D984, P11966, Q2HJD7	1472	47	8785	3.301485893	3.41376E-05	1.31301E-06	2.00806E-05
GOTERM_BP_FAT	GO:0046365~monosaccharide catabolic process	26	1.21325245	1.955E-08	A5PJF9, Q32LG3, Q5W5U3, Q3T186, Q3ZC09, Q5E956, Q3T0P6, A7MB35, A2VE47, Q0IIG5, Q2KJE5, Q3SZ62, Q3ZCI4, Q3T145, P10096, Q5E9B1, A1A4J1, P19858, A6QLL8, Q148N0, A4FUF0, Q9XSJ4, Q3ZBY4, A5D984, P11966, Q2HJD7	1472	48	8785	3.232704937	5.99888E-05	2.22187E-06	3.52874E-05
GOTERM_BP_FAT	GO:0043039~tRNA aminoacylation	23	1.073261783	2.031E-08	A6QNM8, Q3ZBV8, A7YW98, Q3T099, A6H7E1, Q2KJG3, Q0VC30, A5D7A2, Q2KI84, A4FUC8, Q2KIF8, Q3MHH4, A7MBC5, A6QLT9, A7MBD4, Q32LK1, A6QLR2, A5D7G0, Q2T9L8, A8E4P2, Q0P5H7, Q9GMB8, P17248	1472	39	8785	3.51963141	6.23312E-05	2.22618E-06	3.66653E-05
GOTERM_BP_FAT	GO:0006418~tRNA aminoacylation for protein translation	23	1.073261783	2.031E-08	A6QNM8, Q3ZBV8, A7YW98, Q3T099, A6H7E1, Q2KJG3, Q0VC30, A5D7A2, Q2KI84, A4FUC8, Q2KIF8, Q3MHH4, A7MBC5, A6QLT9, A7MBD4, Q32LK1, A6QLR2, A5D7G0, Q2T9L8, A8E4P2, Q0P5H7, Q9GMB8, P17248	1472	39	8785	3.51963141	6.23312E-05	2.22618E-06	3.66653E-05
GOTERM_BP_FAT	GO:0043038~amino acid activation	23	1.073261783	2.031E-08	A6QNM8, Q3ZBV8, A7YW98, Q3T099, A6H7E1, Q2KJG3, Q0VC30, A5D7A2, Q2KI84, A4FUC8, Q2KIF8, Q3MHH4, A7MBC5, A6QLT9, A7MBD4, Q32LK1, A6QLR2, A5D7G0, Q2T9L8, A8E4P2, Q0P5H7, Q9GMB8, P17248	1472	39	8785	3.51963141	6.23312E-05	2.22618E-06	3.66653E-05
GOTERM_BP_FAT	GO:0007264~small GTPase mediated signal transduction	59	2.75314979	2.055E-08	Q17QU4, P84081, Q5E9C0, Q2KI13, Q3SZF2, Q0IIG8, P19803, Q32LJ2, Q0IIG7, A6QLS9, A4IFU2, Q3SWY9, P11023, A6QLB1, P62998, Q5E9M9, Q2TBH7, A4IFP7, P10948, A7YY55, Q3T0F5, Q1RMI2, Q2HJF8, Q17R06, P61585, A6H7G2, Q1RMR4, Q17QB7, Q5E9I6, Q0IIM6, Q2KJ93, Q3ZCK2, A2VDK6, Q0IIM2, A5D7A4, A5D977, A6QR46, Q3ZBG1, Q0VCB4, A4FV54, A5D7R9, P61223, P62833, Q3SZA6, Q148J4, Q3SZI4, Q2KI07, Q58DW6, Q2HJH2, P50397, Q3MHP2, A5PKL2, P21856, Q58DS9, P19035, Q2YDM1, Q2TA37, Q08DI5, Q08DW1	1472	170	8785	2.071271579	6.30574E-05	2.17446E-06	3.70925E-05
GOTERM_BP_FAT	GO:0044275~cellular carbohydrate catabolic process	28	1.306579561	2.752E-08	A5PJF9, Q32LG3, Q5W5U3, Q3T186, Q3ZC09, Q5E956, Q3T0P6, A7MB35, A2VE47, Q9MYM4, Q0IIG5, Q2KJE5, Q3SZ62, Q3ZCI4, Q3T145, P10096, Q5E9B1, A1A4J1, P19858, A6QLL8, Q148N0, A4FUF0, A6QQR7, Q9XSJ4, Q3ZBY4, A5D984, P11966, Q2HJD7	1472	55	8785	3.038290514	8.44538E-05	2.81524E-06	4.96791E-05
GOTERM_BP_FAT	GO:0044271~nitrogen compound biosynthetic process	75	3.499766682	6.04E-08	Q5EA40, Q2HJ58, A7E3T7, Q2KJH7, A4IFQ7, O97681, Q3T0G8, Q3T0C6, Q59A32, P32876, Q4JIJ3, Q3SWY3, Q3SZJ0, Q1RMS2, P57709, P40682, P05631, Q05927, P05630, Q0VD27, Q2KHU0, P33097, P21282, A2VDS0, P13619, Q2KJB6, Q3ZBD3, A0JNE9, Q17QK8, A5PK70, Q00361, A6H7F4, Q2KIN5, P11019, A7YWE4, P12344, Q58DK5, Q1LZA3, Q1JQD7, Q58DT4, P31408, P31404, Q2KIW9, A0JNA3, P31754, Q3SZ18, Q3SZA5, P00376, P13620, P13621, Q2NL31, Q58DW2, Q08DA1, Q58DL1, P22027, Q2TA32, Q0VCK0, Q3T0Z7, Q2HJ26, P52174, P52175, Q3T0Q4, Q8MKF1, P22600, Q0VC13, Q56JW4, Q58D49, A0JNI4, Q0VCY0, Q5E9T4, P56658, Q58D08, Q28851, P00829, P61420, Q28852, Q08DW2	1472	243	8785	1.841997115	0.000185349	5.97955E-06	0.000109035
GOTERM_BP_FAT	GO:0006119~oxidative phosphorylation	29	1.353243117	6.144E-08	Q00361, Q6QTG5, P11019, O97681, Q3T0G8, P31408, P32876, Q05752, P31404, P52505, P13620, P13621, Q85BD6, P40682, P05631, P31800, P05630, Q02827, Q6QTH0, Q05B87, P21282, P13619, Q02372, P15690, Q2KJB6, P00829, Q28852, P61420, P00129	1472	60	8785	2.884567482	0.000188545	5.89256E-06	0.000110915
GOTERM_BP_FAT	GO:0006096~glycolysis	22	1.026598227	6.964E-08	Q3T145, A5PJF9, Q32LG3, Q5W5U3, P10096, Q5E9B1, A1A4J1, Q3ZC09, Q5E956, P19858, Q3T0P6, A6QLL8, A7MB35, Q148N0, A2VE47, Q0IIG5, Q2KJE5, Q9XSJ4, Q3ZBY4, A5D984, Q3SZ62, P11966	1472	38	8785	3.455198799	0.000213713	6.47684E-06	0.000125723
GOTERM_BP_FAT	GO:0016052~carbohydrate catabolic process	30	1.399906673	1.221E-07	A5PJF9, Q32LG3, Q5W5U3, Q3T186, Q3ZC09, Q5E956, Q3T0P6, A7MB35, A2VE47, Q9MYM4, Q0IIG5, Q5EAB4, Q2KJE5, Q3SZ62, Q3ZCI4, Q3T145, Q5E9B1, P10096, A1A4J1, Q3ZCJ2, P19858, A6QLL8, Q148N0, A4FUF0, A6QQR7, Q9XSJ4, Q3ZBY4, A5D984, P11966, Q2HJD7	1472	65	8785	2.754494147	0.000374784	1.10251E-05	0.000220495

Category	Term	Count	%	PValue	Genes	List Total	Pop Hits	Pop Total	Fold Enrichment	Bonferroni	Benjamini	FDR
GOTERM_BP_FAT	GO:0043632~modification-dependent macromolecule catabolic process	70	3.266448903	1.519E-07	Q1RMX2, Q0VCL3, Q71SS4, A6QR55, Q3SX24, A1L506, Q0VCP5, A7MBJ5, Q2KJG2, Q29RK4, Q2TBX8, Q0VD31, A3KMV5, O41515, Q2HJE4, A3KMV3, P23356, Q3T0X5, Q0IIF7, Q0P594, Q9XSJ0, Q2KII4, Q2TBG8, Q3SZ52, Q58DU5, Q3MHX6, Q0IIM6, A3KN22, A6QNM7, Q32KL2, A1A4K3, Q3T0Y5, Q32PA5, Q5E953, Q3ZBT1, Q2T9S7, Q0P568, P62194, Q3ZCK9, Q58DG6, Q3SZ43, Q3ZBG0, Q32PF3, Q5E9J6, P61955, Q1RMU2, A6H744, Q2KIJ6, Q5E9F9, Q95M18, Q5E987, A4IFN7, A4FV12, Q3T168, Q0P5K3, Q1RMW1, A2VE14, Q3T0Z3, A2VDP1, Q32LN1, P61085, Q2KIK0, Q3ZCF7, A2VE20, Q32PG3, Q3ZCF3, Q59HJ6, Q08DE9, Q1LZE1, A2VEA3	1472	226	8785	1.848517459	0.000465959	1.33161E-05	0.000274148
GOTERM_BP_FAT	GO:0019941~modification-dependent protein catabolic process	70	3.266448903	1.519E-07	Q1RMX2, Q0VCL3, Q71SS4, A6QR55, Q3SX24, A1L506, Q0VCP5, A7MBJ5, Q2KJG2, Q29RK4, Q2TBX8, Q0VD31, A3KMV5, O41515, Q2HJE4, A3KMV3, P23356, Q3T0X5, Q0IIF7, Q0P594, Q9XSJ0, Q2KII4, Q2TBG8, Q3SZ52, Q58DU5, Q3MHX6, Q0IIM6, A3KN22, A6QNM7, Q32KL2, A1A4K3, Q3T0Y5, Q32PA5, Q5E953, Q3ZBT1, Q2T9S7, Q0P568, P62194, Q3ZCK9, Q58DG6, Q3SZ43, Q3ZBG0, Q32PF3, Q5E9J6, P61955, Q1RMU2, A6H744, Q2KIJ6, Q5E9F9, Q95M18, Q5E987, A4IFN7, A4FV12, Q3T168, Q0P5K3, Q1RMW1, A2VE14, Q3T0Z3, A2VDP1, Q32LN1, P61085, Q2KIK0, Q3ZCF7, A2VE20, Q32PG3, Q3ZCF3, Q59HJ6, Q08DE9, Q1LZE1, A2VEA3	1472	226	8785	1.848517459	0.000465959	1.33161E-05	0.000274148
GOTERM_BP_FAT	GO:0009060~aerobic respiration	17	0.793280448	1.587E-07	Q3T145, Q3MHX5, Q32LG3, P00432, O77784, P35720, Q29RK1, Q05B87, Q9XSG3, P11179, Q58D96, Q58DR8, P20004, Q148D3, Q95123, Q0VCU1, Q3T189	1472	25	8785	4.058288043	0.000486881	1.35277E-05	0.00028646
GOTERM_BP_FAT	GO:0006099~tricarboxylic acid cycle	15	0.699953336	1.828E-07	Q3T145, Q3MHX5, Q32LG3, O77784, P35720, Q29RK1, P11179, Q9XSG3, Q58D96, Q58DR8, P20004, Q148D3, Q95123, Q0VCU1, Q3T189	1472	20	8785	4.476052989	0.000560914	1.5164E-05	0.00033003
GOTERM_BP_FAT	GO:0046356~acetyl-CoA catabolic process	15	0.699953336	1.828E-07	Q3T145, Q3MHX5, Q32LG3, O77784, P35720, Q29RK1, P11179, Q9XSG3, Q58D96, Q58DR8, P20004, Q148D3, Q95123, Q0VCU1, Q3T189	1472	20	8785	4.476052989	0.000560914	1.5164E-05	0.00033003
GOTERM_BP_FAT	GO:0051188~cofactor biosynthetic process	29	1.353243117	2.224E-07	Q17QE6, Q2TBG7, Q2KIN5, Q32PJ6, Q58DK5, Q1JQD7, Q0II59, Q2T9Y6, Q2KIL4, Q4JIJ3, A4FV58, A6QR05, Q2NL34, Q3SZG8, Q3ZBS0, Q2KJF3, Q5EAC2, P22600, Q05B87, Q3T131, Q32S38, Q58D49, Q58CW1, A4FUD0, Q5E9T4, Q0P5C2, Q24K00, Q0P5A2, Q5BIP7	1472	63	8785	2.747207126	0.000682205	1.79587E-05	0.000401419
GOTERM_BP_FAT	GO:0006084~acetyl-CoA metabolic process	15	0.699953336	4.638E-07	Q3T145, Q3MHX5, Q32LG3, O77784, P35720, Q29RK1, P11179, Q9XSG3, Q58D96, Q58DR8, P20004, Q148D3, Q95123, Q0VCU1, Q3T189	1472	21	8785	4.262907609	0.00142242	3.64976E-05	0.000837281
GOTERM_BP_FAT	GO:0046164~alcohol catabolic process	27	1.259916006	4.887E-07	A5PJF9, Q32LG3, Q5W5U3, Q3T186, Q3ZC09, Q5E956, Q3T0P6, A7MB35, A2VE47, Q0IIG5, Q2KJE5, Q3SZ62, Q3ZCI4, Q3T145, P10096, Q5E9B1, A1A4J1, P19858, A6QLL8, Q148N0, A4FUF0, A6QQR7, Q9XSJ4, Q3ZBY4, A5D984, P11966, Q2HJD7	1472	58	8785	2.778239786	0.001498589	3.74921E-05	0.00088215
GOTERM_BP_FAT	GO:0006511~ubiquitin-dependent protein catabolic process	37	1.726551563	7.713E-07	Q3MHX6, Q1RMX2, Q0IIM6, Q71SS4, A6QNM7, Q32KL2, A6QR55, A1A4K3, Q3SX24, Q3T0Y5, A1L506, Q0VCP5, Q0P568, P62194, Q3ZCK9, Q29RK4, Q3ZBG0, Q2HJE4, Q32PF3, Q1RMU2, P23356, Q5E9F9, Q95M18, Q3T0X5, Q5E987, Q0P5K3, A2VDP1, Q0IIF7, Q0P594, Q9XSJ0, Q3ZCF7, Q2KII4, Q3ZCF3, Q2TBG8, Q08DE9, A2VEA3, Q58DU5	1472	96	8785	2.300193897	0.002364454	5.77362E-05	0.001392446
GOTERM_BP_FAT	GO:0005996~monosaccharide metabolic process	44	2.053196454	8.346E-07	A5PJF9, Q5W5U3, Q08DP0, Q32LG3, Q3T186, Q3ZC09, Q5E956, Q3SZJ9, Q3T0P6, Q29RK2, A7MB35, Q9MYM4, A7MBC0, A2VE47, Q3T0E7, Q0IIG5, Q2KJE5, Q3B7M9, Q29451, Q58CX1, Q17QL1, Q3SZ62, P61287, Q3ZCI4, Q3T145, Q0VCM4, Q5E9B1, P10096, A1A4J1, Q3ZCJ2, P19858, Q3SYZ6, A6QLL8, Q148N0, A4FUF0, Q2KIM0, Q9XSJ4, Q3ZBY4, A5D984, A7MB78, Q3SZM9, Q3SZB7, P11966, Q2HJD7	1472	124	8785	2.117702489	0.002558017	6.09813E-05	0.001506581
GOTERM_BP_FAT	GO:0043623~cellular protein complex assembly	34	1.586560896	8.867E-07	Q02399, Q0II87, A0JNQ6, Q2T9S0, Q08E52, A1A4I8, Q2HJI2, Q17Q89, P52193, A6H784, A5D9D4, A6QNT3, A1L5A6, Q32KM1, Q5E9H5, Q0P5F2, A6QPI6, Q9TRY0, Q3MHM5, A7YWD2, Q2KJD0, Q3ZCJ7, Q76LV2, P81947, Q02375, Q08DE0, P42028, Q2HJ86, P48427, A4FUH5, A1A4J8, Q2NL24, Q148J6, Q647I9	1472	85	8785	2.387228261	0.002717686	6.3286E-05	0.001600748
GOTERM_BP_FAT	GO:0009109~coenzyme catabolic process	15	0.699953336	1.079E-06	Q3T145, Q3MHX5, Q32LG3, O77784, P35720, Q29RK1, P11179, Q9XSG3, Q58D96, Q58DR8, P20004, Q148D3, Q95123, Q0VCU1, Q3T189	1472	22	8785	4.069139081	0.003305319	7.52425E-05	0.001947441
GOTERM_BP_FAT	GO:0070271~protein complex biogenesis	57	2.659822678	1.159E-06	Q02399, Q0II87, Q58D72, Q0P5L6, A1A4I8, Q2HJI2, Q0IIG5, P18246, Q5E9H5, Q32L01, A6QPI6, P80177, A2VDN5, Q3MHM5, A7YWD2, P42028, Q2KJF9, Q2HJ86, P48427, Q647I9, Q3ZBD3, A1L567, Q3ZC55, A0JNQ6, Q5EA65, Q2T9S0, Q1RMV0, Q08E52, Q17Q89, P52193, A6H784, A5D9D4, A6QR05, A6QNT3, A1L5A6, Q32KM1, Q3SZ18, Q0P5F2, P01096, Q9TRY0, P00432, Q2KJD0, Q3ZCJ7, Q76LV2, P81947, P79132, A6QLI8, Q02375, A0JNI4, Q08DE0, Q59HJ6, O02703, A1A4J8, A4FUH5, Q148J6, Q2NL24, Q95107	1472	180	8785	1.88988904	0.003550995	7.90483E-05	0.002092446
GOTERM_BP_FAT	GO:0006461~protein complex assembly	57	2.659822678	1.159E-06	Q02399, Q0II87, Q58D72, Q0P5L6, A1A4I8, Q2HJI2, Q0IIG5, P18246, Q5E9H5, Q32L01, A6QPI6, P80177, A2VDN5, Q3MHM5, A7YWD2, P42028, Q2KJF9, Q2HJ86, P48427, Q647I9, Q3ZBD3, A1L567, Q3ZC55, A0JNQ6, Q5EA65, Q2T9S0, Q1RMV0, Q08E52, Q17Q89, P52193, A6H784, A5D9D4, A6QR05, A6QNT3, A1L5A6, Q32KM1, Q3SZ18, Q0P5F2, P01096, Q9TRY0, P00432, Q2KJD0, Q3ZCJ7, Q76LV2, P81947, P79132, A6QLI8, Q02375, A0JNI4, Q08DE0, Q59HJ6, O02703, A1A4J8, A4FUH5, Q148J6, Q2NL24, Q95107	1472	180	8785	1.88988904	0.003550995	7.90483E-05	0.002092446
GOTERM_BP_FAT	GO:0051187~cofactor catabolic process	16	0.746616892	1.301E-06	Q3T145, Q3MHX5, Q32LG3, O77784, P35720, Q29RK1, A5D7K0, P11179, Q9XSG3, Q58D96, Q58DR8, P20004, Q148D3, Q95123, Q0VCU1, Q3T189	1472	25	8785	3.819565217	0.003983695	8.67713E-05	0.002347923
GOTERM_BP_FAT	GO:0006006~glucose metabolic process	35	1.633224452	2.29E-06	A5PJF9, Q08DP0, Q32LG3, Q5W5U3, Q3T186, Q3ZC09, Q5E956, Q3T0P6, Q29RK2, A7MB35, A2VE47, Q3T0E7, Q9MYM4, Q0IIG5, Q2KJE5, Q3B7M9, Q3SZ62, P61287, Q3ZCI4, Q3T145, Q0VCM4, Q5E9B1, P10096, A1A4J1, P19858, A6QLL8, Q148N0, A4FUF0, Q9XSJ4, Q3ZBY4, A5D984, A7MB78, Q3SZB7, P11966, Q2HJD7	1472	92	8785	2.270461661	0.007002317	0.000149498	0.004133274
GOTERM_BP_FAT	GO:0006605~protein targeting	33	1.53989734	2.846E-06	A1L567, Q2T9M1, Q1RMV0, A7Z057, Q32L81, Q3T087, Q3SZD1, Q0P5N5, Q3ZC32, A1A4I8, P63103, Q3SZC1, C1JZ67, P52193, Q3SZ87, Q32KP9, Q2T9U1, A6QLB1, P62261, A6QPK4, Q3MHE8, Q3SZI4, A6QLL2, Q1LZB5, A7YWD2, Q2TBS1, Q6EWQ7, Q32PG1, Q2KII7, Q3SYV6, Q3ZCH0, Q0G846, Q5EA68	1472	85	8785	2.317015665	0.008695278	0.000181927	0.005136931
GOTERM_BP_FAT	GO:0006897~endocytosis	31	1.446570229	3.468E-06	A7Z035, Q17QI5, Q0IIE6, A2VDK6, P80746, A4FUG8, Q3T0G8, Q0IIG7, Q5E9Q4, Q08DZ3, Q3T0C9, Q2TBX8, Q95114, Q5EA77, P61763, Q2KJA1, Q1JQ98, P11023, A5D7R9, P62998, P63009, Q0VCK5, P45478, A5D794, P52174, P52175, Q3T0Q4, Q3T093, Q5E9R3, Q32PG1, Q3SYV4, Q08E63, A6H7G2	1472	78	8785	2.371925516	0.010587035	0.00021719	0.006260461
GOTERM_BP_FAT	GO:0010324~membrane invagination	31	1.446570229	3.468E-06	A7Z035, Q17QI5, Q0IIE6, A2VDK6, P80746, A4FUG8, Q3T0G8, Q0IIG7, Q5E9Q4, Q08DZ3, Q3T0C9, Q2TBX8, Q95114, Q5EA77, P61763, Q2KJA1, Q1JQ98, P11023, A5D7R9, P62998, P63009, Q0VCK5, P45478, A5D794, P52174, P52175, Q3T0Q4, Q3T093, Q5E9R3, Q32PG1, Q3SYV4, Q08E63, A6H7G2	1472	78	8785	2.371925516	0.010587035	0.00021719	0.006260461
GOTERM_BP_FAT	GO:0022904~respiratory electron transport chain	14	0.653289781	4.16E-06	Q85BD6, P31800, Q02827, P00157, P68530, Q6QTH0, Q6QTG5, Q3T0G8, Q05752, Q02372, P15690, P52505, P23935, P00129	1472	21	8785	3.978713768	0.012687163	0.000255334	0.007510249
GOTERM_BP_FAT	GO:0016044~membrane organization	44	2.053196454	4.361E-06	A7Z035, Q0VCA3, Q17QI5, P00442, Q1RMV0, A4IF87, Q0IIE6, A2VDK6, Q32LM0, P80746, A4FUG8, Q5E9Q4, Q0IIG7, Q3T0G8, Q08DZ3, Q3T0C9, Q2TBX8, Q95114, Q5EA77, P61763, Q2KJA1, Q1JQ98, A4FV54, P11023, A5D7R9, P62998, A6QPI6, P63009, Q0VCK5, P45478, P41541, A5D794, P52174, P52175, Q3T0Q4, Q76LV2, Q3T093, Q5E9R3, Q32PG1, A6QR14, Q3SYV4, Q08E63, A6H7G2, O02703, Q27991, Q3SZB3	1472	131	8785	2.004542814	0.013294613	0.000262393	0.007872235
GOTERM_BP_FAT	GO:0008652~cellular amino acid biosynthetic process	18	0.839944004	4.578E-06	Q2NL31, Q58DW2, Q5EA40, Q2KJH7, A7E3T7, A4IFQ7, Q0VD27, Q2KHU0, A7YWE4, P33097, Q1LZA3, P12344, Q58DT4, A0JNI4, Q58D08, Q4JIJ3, P00376, Q3SZJ0	1472	33	8785	3.255311265	0.013950847	0.000270138	0.008263541

Category	Term	Count	%	PValue	Genes	List Total	Pop Hits	Pop Total	Fold Enrichment	Bonferroni	Benjamini	FDR
GOTERM_BP_FAT	GO:0007049~cell cycle	73	3.406439571	5.566E-06	P00570, Q2KIZ8, Q02399, Q2TA25, A6QLA6, Q3MHL3, A6QM04, Q3ZBV1, P48734, Q0P5L6, Q2KI13, Q2NKY7, Q08DR9, Q3B7M6, Q0VCV0, Q1JQB2, Q0P5H5, P61287, A2VDN5, Q3ZBH9, Q3SZY3, Q2T9P4, Q2KJI1, Q05B84, Q2YDG3, Q862I1, Q5E964, P62285, Q3T054, Q0P5A1, Q08DQ9, Q08DM7, Q3ZBD9, Q27991, Q1LZG6, Q0P5I7, Q3MHE4, P62157, Q2YDJ0, Q5E994, Q2TA06, Q32PA5, Q3ZBK3, Q17QG2, Q0II23, Q3SZP2, Q2HJG1, Q3T0E7, Q58D97, P52193, A5D7V1, A6H7B3, Q1RMH9, Q32PF3, Q3T0M7, P53712, Q2NL37, Q0VCX4, Q2TBN3, Q3HNG7, Q24JY3, Q2KJD0, Q3SWZ6, P46196, A7YY75, Q5BIN5, P79126, P46193, Q08DE0, A2VE99, A5PJU9, Q3ZCF0, Q2TA37	1472	261	8785	1.669230489	0.016938102	0.000322272	0.010048093
GOTERM_BP_FAT	GO:0019318~hexose metabolic process	39	1.819878675	6.491E-06	A5PJF9, Q5W5U3, Q08DP0, Q32LG3, Q3T186, Q3ZC09, Q5E956, Q3SZJ9, Q3T0P6, Q29RK2, A7MB35, A2VE47, Q3T0E7, Q9MYM4, Q0IIG5, Q2KJE5, Q3B7M9, Q29451, Q58CX1, Q3SZ62, P61287, Q3ZCI4, Q3T145, Q0VCM4, Q5E9B1, P10096, A1A4J1, P19858, A6QLL8, Q148N0, A4FUF0, Q2KIM0, Q9XSJ4, Q3ZBY4, A5D984, A7MB78, Q3SZB7, P11966, Q2HJD7	1472	112	8785	2.078167459	0.019724206	0.000368845	0.011717344
GOTERM_BP_FAT	GO:0006979~response to oxidative stress	26	1.21325245	8.433E-06	Q32KL2, P00442, P35705, Q2NL01, Q58DT4, Q9XSG3, Q3T0G8, Q3MHF7, Q2T9Y6, Q9N2I8, P00435, P00432, Q5E946, Q5E947, Q02366, O97725, Q0P594, P37359, P17694, P48305, Q32S38, Q9BGI3, Q9BGI1, O77834, P42028, Q59HJ6, P41976	1472	62	8785	2.502739306	0.025549145	0.000470456	0.015222558
GOTERM_BP_FAT	GO:0009259~ribonucleotide metabolic process	40	1.866542231	1.002E-05	P00570, A5PK70, Q2HJ58, Q00361, P11019, O97681, Q2HJ33, P31408, Q3T0C6, Q59A32, P31404, P32876, A0JNA3, P31754, Q3SWY3, P13620, P57709, P13621, P40682, Q08DA1, P05631, P22027, Q05927, P05630, Q0VCK0, Q2HJ26, A7MBI8, P52174, P52175, Q3T0Q4, Q0VC13, P21282, Q0VCY0, P56658, P13619, Q59HJ6, P15690, Q2KJB6, Q28851, P00829, Q28852, P61420	1472	118	8785	2.023074797	0.030275186	0.00054883	0.01808184
GOTERM_BP_FAT	GO:0006413~translational initiation	17	0.793280448	1.436E-05	Q5E966, Q9N0T5, Q3ZCK1, Q5E9B4, Q3T102, Q32LC3, Q3T058, Q0II31, Q2KHU8, Q56JZ5, Q3T122, Q5E9D0, Q3SYW6, Q0IIF2, A7MB16, Q3T0V3, Q32LJ9	1472	32	8785	3.170537534	0.043110876	0.00077282	0.025917973
GOTERM_BP_FAT	GO:0045454~cell redox homeostasis	23	1.073261783	1.686E-05	Q0Z7W6, P35705, P23196, Q3ZCL8, Q58DA7, Q5E947, A6QLZ6, Q2TBU2, Q9BGI2, A6QLU8, P10575, A5D7E8, Q9BGI3, Q32S38, Q3MHF7, Q9BGI1, Q3T0L2, O77834, A6H784, Q2KIL5, A6H732, Q9N2I8, A6QL97, Q5E936	1472	53	8785	2.589917453	0.050426901	0.000891719	0.030431262
GOTERM_BP_FAT	GO:0009150~purine ribonucleotide metabolic process	38	1.773215119	2.126E-05	P00570, A5PK70, Q00361, P11019, O97681, Q2HJ33, P31408, Q3T0C6, Q59A32, P31404, P32876, A0JNA3, Q3SWY3, P13620, P57709, P13621, P40682, Q08DA1, P05631, P22027, Q05927, P05630, Q0VCK0, Q2HJ26, A7MBI8, P52174, P52175, Q3T0Q4, Q0VC13, P21282, Q0VCY0, P56658, P13619, Q59HJ6, P15690, Q2KJB6, Q28851, P00829, Q28852, P61420	1472	113	8785	2.006961812	0.063170157	0.001105382	0.038375816
GOTERM_BP_FAT	GO:0031163~metallo-sulfur cluster assembly	8	0.373308446	2.492E-05	Q3SZG8, Q17QE6, Q2TBG7, Q24K00, A4FV58, Q2KJF3, Q32PJ6, Q05B87	1472	8	8785	5.968070652	0.073622353	0.001273743	0.044972661
GOTERM_BP_FAT	GO:0016226~iron-sulfur cluster assembly	8	0.373308446	2.492E-05	Q3SZG8, Q17QE6, Q2TBG7, Q24K00, A4FV58, Q2KJF3, Q32PJ6, Q05B87	1472	8	8785	5.968070652	0.073622353	0.001273743	0.044972661
GOTERM_BP_FAT	GO:0007010~cytoskeleton organization	50	2.333177788	3.101E-05	Q32KS0, Q3B7N2, P38584, A6QNZ5, Q08DR9, P63258, A6H720, A5D7D1, A5D7P5, P57709, P62998, A2VDN5, Q32L92, Q3ZCL5, P61585, Q27991, Q2YDJ0, Q5E997, A4FUA8, P00442, Q3ZCK2, Q2TA45, A2VDK3, Q3ZBK3, A2VDK6, Q0II23, Q2HJG1, Q1LZB2, P52193, Q2TA49, Q0VCF1, Q3T0M7, Q08DP6, P53712, Q0VCX4, A5D7R9, Q2TBN3, Q32P97, A7E3S4, P31976, A0JNH5, Q2KJD0, A0JNH7, P81947, Q09430, A7YY75, Q3SYV4, Q148J6, Q95107, Q1RMT6	1472	167	8785	1.786847501	0.090773484	0.001558801	0.055959521
GOTERM_BP_FAT	GO:0006163~purine nucleotide metabolic process	42	1.959869342	3.901E-05	P00570, A5PK70, Q2HJ58, Q00361, P11019, O97681, Q2HJ33, P31408, Q3T0C6, Q59A32, P31404, P32876, A0JNA3, Q3SZ18, Q3SWY3, P13620, P57709, P13621, P40682, Q08DA1, P05631, P22027, Q05927, Q0VCK0, P05630, Q2HJ26, A7MBI8, P52174, P52175, Q3T0Q4, Q8MKF1, Q0VC13, P21282, Q0VCY0, P56658, P46195, P13619, Q59HJ6, P15690, Q2KJB6, Q28851, P00829, Q28852, P61420	1472	133	8785	1.88465389	0.112840374	0.001929271	0.070402447
GOTERM_BP_FAT	GO:0007017~microtubule-based process	42	1.959869342	3.901E-05	Q2YDJ0, Q32KS0, Q2NL05, Q2T9S0, Q3ZBK3, P38584, Q0II23, Q2HJG1, A6QNZ5, Q08DH4, Q08DR9, A5D7N6, A6H7H2, A6QNT3, Q3T0M7, A5D7P5, Q32KM1, P23356, Q0VCX4, Q2TBN3, P61285, Q32P97, A7MBA1, A2VDN5, Q3MHM5, A7E2Z6, Q5E9M9, A0JNH5, Q2KJD0, Q3ZCJ7, P81947, A0JNH7, Q2HJF8, A7YY75, Q0P5A1, Q3MHR3, Q2HJ86, Q3ZCF0, Q3MHI2, A6QPE8, Q3ZBD2, Q29RT6	1472	133	8785	1.88465389	0.112840374	0.001929271	0.070402447
GOTERM_BP_FAT	GO:0010035~response to inorganic substance	20	0.933271115	4.748E-05	P00435, P35705, P00442, P00432, Q5E946, Q5E947, Q8WN95, Q2KI72, A4FV00, Q5EAC2, Q05B87, P37359, Q0P594, Q9BGI3, Q32S38, Q3MHF7, Q9BGI1, O77834, A5PJI5, Q9N2I8, P41976	1472	45	8785	2.652475845	0.135591124	0.002310175	0.085671792
GOTERM_BP_FAT	GO:0016053~organic acid biosynthetic process	33	1.53989734	5.081E-05	Q5EA40, Q5E956, Q0VCA7, Q95JH2, Q2KJH7, A7E3T7, Q2KJG4, A4IFQ7, A7YWE4, Q1LZA3, Q32LM0, P12344, Q7YS70, Q58DT4, Q4JIJ3, P58108, P52505, A5PJC5, P00376, Q3SZJ0, Q2NL31, P80177, Q58DW2, Q3ZCJ2, A6H7G3, Q0VD27, Q2KHU0, P33097, A0JNI4, Q58D08, Q5BIP7, Q3ZBF7, Q3ZBY7	1472	96	8785	2.051524287	0.144397078	0.002433735	0.091689483
GOTERM_BP_FAT	GO:0046394~carboxylic acid biosynthetic process	33	1.53989734	5.081E-05	Q5EA40, Q5E956, Q0VCA7, Q95JH2, Q2KJH7, A7E3T7, Q2KJG4, A4IFQ7, A7YWE4, Q1LZA3, Q32LM0, P12344, Q7YS70, Q58DT4, Q4JIJ3, P58108, P52505, A5PJC5, P00376, Q3SZJ0, Q2NL31, P80177, Q58DW2, Q3ZCJ2, A6H7G3, Q0VD27, Q2KHU0, P33097, A0JNI4, Q58D08, Q5BIP7, Q3ZBF7, Q3ZBY7	1472	96	8785	2.051524287	0.144397078	0.002433735	0.091689483
GOTERM_BP_FAT	GO:0000279~M phase	36	1.679888007	8.458E-05	Q2YDJ0, Q2TA25, Q32PA5, A6QM04, Q3ZBK3, Q17QG2, Q0P5L6, P48734, Q0II23, Q3SZP2, Q2NKY7, Q2HJG1, Q58D97, Q08DR9, Q3B7M6, A6H7B3, Q0VCV0, Q1JQB2, Q3T0M7, Q2TBN3, Q3HNG7, Q24JY3, Q3SZY3, Q2KJD0, Q05B84, Q2KJI1, Q862I1, Q5E964, P62285, Q3T054, A7YY75, Q0P5A1, Q08DQ9, Q3ZBD9, Q3ZCF0, Q1LZG6	1472	111	8785	1.935590482	0.228627274	0.00398563	0.152574563
GOTERM_BP_FAT	GO:0018130~heterocycle biosynthetic process	17	0.793280448	8.926E-05	Q17QK8, Q3T0Z7, Q2KJH7, Q2KIN5, A7YWE4, P22600, Q58DK5, Q1JQD7, Q58DT4, Q56JW4, Q58D49, Q5E9T4, P56658, Q58D08, Q5BIP7, Q3SZ18, Q3ZBD3	1472	36	8785	2.818255586	0.239631057	0.004142178	0.161012745
GOTERM_BP_FAT	GO:0006890~retrograde vesicle-mediated transport, Golgi to ER	8	0.373308446	9.581E-05	P53620, P53619, Q27954, A5PJ65, P35605, A2VDR8, P35604, Q28104	1472	9	8785	5.304951691	0.254761975	0.004379213	0.172816202
GOTERM_BP_FAT	GO:0016071~mRNA metabolic process	46	2.146523565	0.0001007	Q17QI2, Q5E959, Q3MHX3, Q2YDD2, Q24JZ8, Q2KJJ0, Q5E992, Q3ZBV3, Q5E9Z8, Q3SZR8, Q3ZBK6, A5D7H5, P09867, Q3ZBA6, Q3ZCE0, Q0P5D0, Q3ZCE2, Q0P5D2, Q2HJ60, P61286, A1A4K8, Q32PE9, Q29RH4, A6QPH1, Q3ZCA2, Q3T147, Q8MJJ7, Q5E9J1, Q3T0D0, Q3T102, P67808, A5D9H5, A0JN52, Q3T106, Q3B8S0, Q3SYR3, Q3MHR5, P79101, Q2KIR1, Q3B7L6, Q3MHE2, Q2KJ71, Q2NL22, Q10568, Q1RMR2, Q0VCY7	1472	156	8785	1.759815705	0.265965603	0.004536728	0.181710561
GOTERM_BP_FAT	GO:0000302~response to reactive oxygen species	13	0.606626225	0.0001022	P00435, P00442, P35705, P00432, Q5E946, Q5E947, P37359, Q0P594, Q9BGI3, Q3MHF7, Q9BGI1, O77834, Q9N2I8, P41976	1472	23	8785	3.373257325	0.269294279	0.004536699	0.184379156
GOTERM_BP_FAT	GO:0042773~ATP synthesis coupled electron transport	11	0.513299113	0.0001047	Q3T0G8, Q85BD6, P31800, Q02372, Q05752, P15690, Q02827, Q6QTG5, Q6QTH0, P52505, P00129	1472	17	8785	3.861692775	0.27480588	0.004579708	0.188824481
GOTERM_BP_FAT	GO:0034404~nucleobase, nucleoside and nucleotide biosynthetic process	46	2.146523565	0.0001188	A0JNE9, A5PK70, Q2HJ58, Q00361, A6H7F4, P11019, O97681, P31408, Q3T0C6, Q59A32, P31404, P32876, Q2KIW9, A0JNA3, P31754, Q3SZ18, Q3SWY3, P00376, P13620, P57709, P13621, Q1RMS2, P40682, Q08DA1, P05631, P22027, Q2TA32, Q05927, Q0VCK0, P05630, Q2HJ26, P52174, P52175, Q3T0Q4, Q8MKF1, Q0VC13, P21282, Q56JW4, A2VDS0, Q0VCY0, P56658, P13619, Q2KJB6, Q28851, P00829, Q28852, P61420, Q08DW2	1472	157	8785	1.748606688	0.305532447	0.005122186	0.214239316
GOTERM_BP_FAT	GO:0034654~nucleobase, nucleoside, nucleotide and nucleic acid biosynthetic process	46	2.146523565	0.0001188	A0JNE9, A5PK70, Q2HJ58, Q00361, A6H7F4, P11019, O97681, P31408, Q3T0C6, Q59A32, P31404, P32876, Q2KIW9, A0JNA3, P31754, Q3SZ18, Q3SWY3, P00376, P13620, P57709, P13621, Q1RMS2, P40682, Q08DA1, P05631, P22027, Q2TA32, Q05927, Q0VCK0, P05630, Q2HJ26, P52174, P52175, Q3T0Q4, Q8MKF1, Q0VC13, P21282, Q56JW4, A2VDS0, Q0VCY0, P56658, P13619, Q2KJB6, Q28851, P00829, Q28852, P61420, Q08DW2	1472	157	8785	1.748606688	0.305532447	0.005122186	0.214239316
GOTERM_BP_FAT	GO:0015992~proton transport	20	0.933271115	0.0001354	P40682, P22027, P05631, P05630, Q00361, P11019, O97681, P21282, P31408, P13619, P32876, P31404, Q2KJB6, Q28851, P00829, Q28852, P61420, P13620, P11024, P13621	1472	48	8785	2.486696105	0.340120967	0.005756959	0.244221714

Category	Term	Count	%	PValue	Genes	List Total	Pop Hits	Pop Total	Fold Enrichment	Bonferroni	Benjamini	FDR
GOTERM_BP_FAT	GO:0006790~sulfur metabolic process	21	0.979934671	0.0001453	Q2NL31, Q58DW2, P00435, Q5I598, P00442, Q3MHL4, A7E3T7, Q3SZU4, Q0VD27, Q9TUM3, Q5EAC2, Q8MKF1, Q9XSG3, A5PKG4, Q32S38, Q2T9Y6, Q5E9T4, Q4JIJ3, Q5BIP7, Q17QV7, O18756	1472	52	8785	2.410182379	0.359874879	0.006092211	0.262054017
GOTERM_BP_FAT	GO:0048285~organelle fission	29	1.353243117	0.0001471	Q2YDJ0, Q148K5, Q2TA25, Q32PA5, A6QM04, Q3ZBK3, Q17QG2, Q0P5L6, P48734, Q3SZP2, Q2NKY7, Q58D97, Q08DR9, Q0VCP2, Q3B7M6, A6H7B3, Q1JQB2, Q2TBN3, Q24JY3, Q3SZY3, Q05B84, P62285, Q3T054, A7YY75, Q0P5A1, Q3ZBD9, Q3ZCF0, O02703, Q1LZG6	1472	84	8785	2.060405344	0.363254473	0.006081234	0.265159569
GOTERM_BP_FAT	GO:0051301~cell division	36	1.679888007	0.0001858	Q2YDJ0, Q02399, Q5E994, Q2TA25, Q32PA5, A6QM04, Q3ZBK3, Q3ZBV1, Q17QG2, P48734, Q3ZBK8, Q3SZP2, Q2NKY7, Q58D97, Q3T0E7, Q3B7M6, Q5E9D5, P61287, Q2TBN3, A2VDN5, Q24JY3, Q3SZY3, Q05B84, Q2YDG3, Q2KJI1, P62285, Q3T054, A7YY75, Q0P5A1, Q08DM7, A2VE99, Q3ZBD9, A5PJU9, Q27991, Q1LZG6, P27214	1472	115	8785	1.868265595	0.434623192	0.007574671	0.334875454
GOTERM_BP_FAT	GO:0008610~lipid biosynthetic process	48	2.239850677	0.0001866	P07514, A0JNC1, P10790, Q8WMY2, Q5EA65, Q3MHZ7, Q5E956, Q0VCA7, Q95JH2, Q1LZ95, Q2KJG4, Q29RK2, Q32LM0, Q1LZA4, Q7YS70, Q32PF2, Q70VZ7, P58108, A6QR05, Q58CR4, Q1JQD0, Q3ZBE9, P52505, A5PJC5, Q3SZL5, Q3SZL3, Q5E9H7, Q5E9J5, P80177, Q2KHY9, Q8WMV1, A6H7G3, A4FUD2, Q3T103, Q2KJ28, P84466, P56966, Q5E9T8, Q58DH7, A6QR14, Q5E9R6, P20456, Q3ZBF7, Q3ZBY7, Q2NL29, Q3ZCD7, Q0VCY6, Q3ZBF8	1472	169	8785	1.695073321	0.436098762	0.007509506	0.336407478
GOTERM_BP_FAT	GO:0006818~hydrogen transport	20	0.933271115	0.0001869	P40682, P22027, P05631, P05630, Q00361, P11019, O97681, P21282, P31408, P13619, P32876, P31404, Q2KJB6, Q28851, P00829, Q28852, P61420, P13620, P11024, P13621	1472	49	8785	2.435947205	0.436550397	0.007422671	0.336877189
GOTERM_BP_FAT	GO:0000096~sulfur amino acid metabolic process	10	0.466635558	0.0001924	A5PKG4, Q2NL31, Q58DW2, Q32S38, Q5I598, Q3MHL4, Q4JIJ3, A7E3T7, Q3SZU4, Q0VD27	1472	15	8785	3.978713768	0.445887523	0.007540498	0.346672793
GOTERM_BP_FAT	GO:0009069~serine family amino acid metabolic process	11	0.513299113	0.0002003	A5PKG4, Q58DW2, Q32S38, P25285, A0JNI4, Q3SZU4, Q2KHU0, O46504, P20821, P00376, Q3SZ20	1472	18	8785	3.647154287	0.4592032	0.007750966	0.360929974
GOTERM_BP_FAT	GO:0009260~ribonucleotide biosynthetic process	34	1.586560896	0.0002132	A5PK70, Q2HJ58, Q00361, P11019, O97681, P31408, Q3T0C6, Q59A32, P32876, P31404, A0JNA3, P31754, Q3SWY3, P13620, P57709, P13621, P40682, Q08DA1, P05631, P22027, P05630, Q0VCK0, P52174, Q2HJ26, P52175, Q3T0Q4, Q0VC13, P21282, Q0VCY0, P56658, P13619, Q2KJB6, Q28851, P00829, Q28852, P61420	1472	107	8785	1.896396282	0.480251023	0.00814675	0.384193748
GOTERM_BP_FAT	GO:0006891~intra-Golgi vesicle-mediated transport	8	0.373308446	0.000273	P53620, P53619, P60519, Q27954, P35605, P35604, Q28104, Q3ZC32	1472	10	8785	4.774456522	0.567410357	0.010291928	0.491691589
GOTERM_BP_FAT	GO:0000087~M phase of mitotic cell cycle	28	1.306579561	0.0003016	Q2YDJ0, Q2TA25, Q32PA5, A6QM04, Q3ZBK3, Q17QG2, Q0P5L6, P48734, Q3SZP2, Q2NKY7, Q58D97, Q08DR9, Q3B7M6, A6H7B3, Q1JQB2, Q2TBN3, Q24JY3, Q3SZY3, Q05B84, P62285, Q862I1, Q3T054, A7YY75, Q0P5A1, Q08DQ9, Q3ZBD9, Q3ZCF0, Q1LZG6	1472	83	8785	2.013325039	0.603728697	0.011225014	0.543005633
GOTERM_BP_FAT	GO:0043933~macromolecular complex subunit organization	76	3.546430238	0.0003285	Q02399, Q3T0N9, Q0II87, Q58D72, Q0P5L6, A1A4I8, Q2HJI2, Q0IIG5, A6H767, P84227, P18246, Q32S29, Q32L01, Q5E9H5, A6QPI6, A2VDN5, P80177, Q17QJ0, Q3MHM5, A7YWD2, Q2T9P4, Q862I1, Q0VCI6, P42028, Q2KJF9, Q2HJ86, P48427, Q647I9, Q3ZBD3, A1L567, Q3ZC55, Q2TA40, A0JNQ6, Q17QG8, Q5EA65, Q2T9S0, Q1RMV0, A6QNM2, Q08E52, Q3T116, Q9TU47, Q3T122, Q17Q89, P52193, A6H784, A5D9D4, A6QR05, A1L5A6, A6QNT3, Q3SZ18, Q32KM1, Q0VCX5, Q0P5F2, Q5E9F8, Q3HNG7, P01096, Q9TRY0, P00432, Q2KJD0, Q3ZCJ7, Q76LV2, P81947, P79132, A6QLI8, Q02375, A7YY75, A0JNI4, Q08DE0, Q59HJ6, P19035, O02703, A4FUH5, A1A4J8, Q148J6, Q2NL24, Q95107	1472	308	8785	1.47264081	0.635223301	0.012076724	0.591441624
GOTERM_BP_FAT	GO:0009165~nucleotide biosynthetic process	43	2.006532898	0.0003435	A0JNE9, A5PK70, Q2HJ58, Q00361, P11019, O97681, P31408, Q3T0C6, Q59A32, P31404, P32876, Q2KIW9, A0JNA3, P31754, Q3SZ18, Q3SWY3, P00376, P13620, P57709, P13621, Q1RMS2, P40682, Q08DA1, P05631, P22027, Q2TA32, Q0VCK0, P05630, Q2HJ26, P52174, P52175, Q3T0Q4, Q8MKF1, Q0VC13, P21282, A2VDS0, Q0VCY0, P56658, P13619, Q2KJB6, Q28851, P00829, Q28852, P61420, Q08DW2	1472	150	8785	1.71084692	0.651558219	0.012472566	0.618227262
GOTERM_BP_FAT	GO:0012501~programmed cell death	52	2.4265049	0.000347	Q95KV7, Q5E9L4, Q5E9C2, A0JNK3, P56541, Q3T0K8, Q58CZ0, P45478, Q5E9M9, Q645M6, Q0VC36, Q29RZ9, P43481, Q2HJF8, Q0VC37, Q0II48, Q2HJF6, Q0P5M9, P15690, Q08DY9, Q0V882, Q3SX43, P00442, Q2KJE0, Q3T0P5, Q3ZBT1, Q32KL9, Q32LM0, Q0II26, Q32LE4, Q3T0I5, P38447, Q5E9J6, Q3ZBG5, Q2NL36, Q0VCX4, P82922, P45879, Q0P5F4, A6QQW8, P00435, A7E3S4, P32007, Q3T169, Q1JQC5, A4IF78, Q08DE0, Q2HJH9, Q5EAC7, O02703, Q2NL24, Q0IIH8	1472	192	8785	1.616352468	0.655264343	0.012450983	0.624478114
GOTERM_BP_FAT	GO:0006399~tRNA metabolic process	28	1.306579561	0.0003748	A6QNM8, Q3ZBV8, Q05B50, A7YW98, O02691, A6H7F4, Q2KJG3, A5D7A2, A4FUC8, Q2KIF8, Q3MHH4, A7MBC5, Q1JQB6, Q1RML6, A8E4P2, Q0P5H7, P17248, Q9GMB8, Q3T099, A6H7E1, Q0VC30, Q2KI84, A6QLT9, A7MBD4, Q32LK1, A6QLR2, A5D7G0, Q2T9L8	1472	84	8785	1.989356884	0.683469749	0.013286944	0.674361579
GOTERM_BP_FAT	GO:0006915~apoptosis	51	2.379841344	0.0003825	Q95KV7, Q5E9L4, Q5E9C2, A0JNK3, P56541, Q3T0K8, Q58CZ0, P45478, Q5E9M9, Q645M6, Q0VC36, Q29RZ9, Q2HJF8, Q0II48, Q0VC37, Q2HJF6, Q0P5M9, P15690, Q08DY9, Q0V882, Q3SX43, P00442, Q2KJE0, Q3T0P5, Q3ZBT1, Q32KL9, Q32LM0, Q0II26, Q32LE4, Q3T0I5, P38447, Q5E9J6, Q3ZBG5, Q2NL36, Q0VCX4, P82922, P45879, Q0P5F4, A6QQW8, P00435, A7E3S4, P32007, Q3T169, Q1JQC5, A4IF78, Q08DE0, Q2HJH9, Q5EAC7, O02703, Q2NL24, Q0IIH8	1472	188	8785	1.61899789	0.690918819	0.01340533	0.688274403
GOTERM_BP_FAT	GO:0008380~RNA splicing	32	1.493233784	0.0004357	Q5E959, Q3MHX3, Q2KJJ0, Q5E992, Q3ZBV3, Q5E9Z8, Q3SZR8, Q3ZBK6, A5D7H5, P09867, Q3ZCE0, Q0P5D0, Q2HJ60, P61286, A1A4K8, Q32PE9, Q29RH4, Q3T147, Q5E9J1, Q3T0D0, P67808, A0JN52, Q3T106, Q3B8S0, Q3MHR5, Q2KIR1, Q3B7L6, Q3MHE2, Q2KJ71, Q2NL22, Q1RMR2, Q0VCY7	1472	102	8785	1.872335891	0.737474051	0.015082885	0.783596233
GOTERM_BP_FAT	GO:0009152~purine ribonucleotide biosynthetic process	32	1.493233784	0.0004357	A5PK70, Q00361, P11019, O97681, P31408, Q3T0C6, Q59A32, P32876, P31404, A0JNA3, Q3SWY3, P13620, P13621, P57709, P40682, Q08DA1, P05631, P22027, P05630, Q0VCK0, P52174, Q2HJ26, P52175, Q3T0Q4, Q0VC13, P21282, Q0VCY0, P56658, P13619, Q2KJB6, Q28851, P00829, Q28852, P61420	1472	102	8785	1.872335891	0.737474051	0.015082885	0.783596233
GOTERM_BP_FAT	GO:0006518~peptide metabolic process	13	0.606626225	0.0004461	Q1JPJ8, Q3T134, P67810, P00435, P00442, Q2KI36, Q3SZU5, Q5EAC2, Q9XSG3, Q32S38, Q2T9Y6, Q24K02, Q0V8B6	1472	26	8785	2.984035326	0.74576492	0.015269811	0.802322726
GOTERM_BP_FAT	GO:0070647~protein modification by small protein conjugation or removal	23	1.073261783	0.0004529	Q3MHX6, Q0VCL3, Q1RMX2, P63048, A3KN22, A1A4K3, Q1RMW1, Q32PA5, Q0VCP5, A7MBJ5, A2VDP1, Q32LN1, Q08E57, Q2HJI9, Q32PG3, P62992, Q08E63, Q32PF3, P61955, Q0P5I7, P23356, Q3ZCC3, Q0P5F4	1472	64	8785	2.144775391	0.751001039	0.015329145	0.814464901
GOTERM_BP_FAT	GO:0019725~cellular homeostasis	56	2.613159123	0.0005714	Q02399, Q1LZ90, P23196, Q3T0G8, Q9MYM4, Q0IIG5, A5PJI5, Q2KIL5, A6QL97, Q9TTJ5, P57709, Q0Z7W6, P45478, Q3SZK8, Q5E9M9, Q3ZCL8, Q58DA7, Q2TBU2, Q5E9B4, Q2KJI7, Q05B87, P37359, Q2HJF8, Q9BGI2, Q9BGI3, O46414, Q9BGI1, O46415, P15690, A6H732, Q5E936, Q5EA61, Q0V8R6, P35705, P00442, Q3T0E0, A6QLU8, Q3T058, Q3MHF7, A6H784, Q9N2I8, Q5E946, A6QLZ6, Q5E947, P79132, P10575, A5D7E8, Q32S38, Q3T0L2, O77834, Q0VCU1, P00829, O02703, A1A4J8, P20072, P18203	1472	215	8785	1.554474216	0.826923588	0.019090394	1.026438675
GOTERM_BP_FAT	GO:0006164~purine nucleotide biosynthetic process	35	1.633224452	0.0005896	A5PK70, Q2HJ58, Q00361, P11019, O97681, P31408, Q3T0C6, Q59A32, P31404, P32876, A0JNA3, Q3SZ18, Q3SWY3, P13620, P57709, P13621, P40682, Q08DA1, P05631, P22027, P05630, Q0VCK0, Q2HJ26, P52174, P52175, Q3T0Q4, Q8MKF1, Q0VC13, P21282, Q0VCY0, P56658, P13619, Q2KJB6, Q28851, P00829, Q28852, P61420	1472	117	8785	1.785320281	0.836343108	0.019481465	1.059012604
GOTERM_BP_FAT	GO:0010498~proteasomal protein catabolic process	11	0.513299113	0.0006118	Q3MHX6, Q95M18, Q1RMX2, Q3ZCF7, Q71SS4, Q32KL2, Q3SX24, Q32PF3, P62194, Q29RK4, Q0P594	1472	20	8785	3.282438859	0.847149157	0.019994109	1.098759833
GOTERM_BP_FAT	GO:0034599~cellular response to oxidative stress	11	0.513299113	0.0006118	Q3T0G8, Q58DT4, Q9BGI3, Q3MHF7, P00435, Q9BGI1, P00442, P35705, P00432, Q59HJ6, Q5E947, P37359	1472	20	8785	3.282438859	0.847149157	0.019994109	1.098759833
GOTERM_BP_FAT	GO:0016126~sterol biosynthetic process	11	0.513299113	0.0006118	P07514, Q5E9T8, Q58DH7, Q8WMY2, A6QR14, Q8WMV1, Q3ZBE9, Q1LZ95, A4FUD2, P84466, Q5E9J5	1472	20	8785	3.282438859	0.847149157	0.019994109	1.098759833
GOTERM_BP_FAT	GO:0043161~proteasomal ubiquitin-dependent protein catabolic process	11	0.513299113	0.0006118	Q3MHX6, Q95M18, Q1RMX2, Q3ZCF7, Q71SS4, Q32KL2, Q3SX24, Q32PF3, P62194, Q29RK4, Q0P594	1472	20	8785	3.282438859	0.847149157	0.019994109	1.098759833

Category	Term	Count	%	PValue	Genes	List Total	Pop Hits	Pop Total	Fold Enrichment	Bonferroni	Benjamini	FDR
GOTERM_BP_FAT	GO:0030262~apoptotic nuclear changes	8	0.373308446	0.0006421	Q95KV7, P45478, Q0VC37, P00442, P38447, O02703, Q08DY9, Q58CZ0	1472	11	8785	4.34041502	0.860710004	0.020751838	1.15279248
GOTERM_BP_FAT	GO:0008064~regulation of actin polymerization or depolymerization	13	0.606626225	0.0006818	Q32LP2, Q5E997, A4FUA8, Q5E963, Q3T035, Q1LZB2, Q3MHR7, Q5E9D5, Q58CQ2, Q1JP79, P61157, Q3SYX9, P62998	1472	27	8785	2.873515499	0.876684866	0.021790781	1.223594512
GOTERM_BP_FAT	GO:0030832~regulation of actin filament length	13	0.606626225	0.0006818	Q32LP2, Q5E997, A4FUA8, Q5E963, Q3T035, Q1LZB2, Q3MHR7, Q5E9D5, Q58CQ2, Q1JP79, P61157, Q3SYX9, P62998	1472	27	8785	2.873515499	0.876684866	0.021790781	1.223594512
GOTERM_BP_FAT	GO:0006414~translational elongation	9	0.419972002	0.0007018	P68103, Q3SZV3, Q95140, P49410, Q56K14, A3KN48, P43896, Q5E983, P42899	1472	14	8785	3.836616848	0.884040358	0.022192877	1.259321202
GOTERM_BP_FAT	GO:0042775~mitochondrial ATP synthesis coupled electron transport	9	0.419972002	0.0007018	Q3T0G8, P31800, Q02372, Q05752, Q02827, Q6QTG5, Q6QTH0, P52505, P00129	1472	14	8785	3.836616848	0.884040358	0.022192877	1.259321202
GOTERM_BP_FAT	GO:0015985~energy coupled proton transport, down electrochemical gradient	17	0.793280448	0.000747	P40682, P05631, P05630, Q00361, P11019, O97681, P21282, P31408, P13619, P32876, P31404, Q2KJB6, P00829, Q28852, P61420, P13620, P13621	1472	42	8785	2.415647645	0.899082123	0.023366471	1.339982857
GOTERM_BP_FAT	GO:0015986~ATP synthesis coupled proton transport	17	0.793280448	0.000747	P40682, P05631, P05630, Q00361, P11019, O97681, P21282, P31408, P13619, P32876, P31404, Q2KJB6, P00829, Q28852, P61420, P13620, P13621	1472	42	8785	2.415647645	0.899082123	0.023366471	1.339982857
GOTERM_BP_FAT	GO:0034621~cellular macromolecular complex subunit organization	53	2.473168455	0.0007715	Q02399, Q0II87, A1A4I8, Q2HJI2, A6H767, P84227, Q32S29, Q5E9H5, Q32L01, A6QPI6, Q17QJ0, Q3MHM5, A7YWD2, Q2T9P4, Q862I1, Q0VCI6, P42028, Q2HJ86, P48427, Q647I9, Q2TA40, A0JNQ6, Q17QG8, Q2T9S0, A6QNM2, Q08E52, Q3T116, Q3T122, Q9TU47, Q17Q89, P52193, A6H784, A5D9D4, A6QNT3, A1L5A6, Q32KM1, Q0VCX5, Q0P5F2, Q3HNG7, Q5E9F8, Q9TRY0, Q2KJD0, Q3ZCJ7, Q76LV2, P81947, Q02375, A7YY75, Q08DE0, A1A4J8, A4FUH5, Q148J6, Q2NL24, Q95107	1472	203	8785	1.558166229	0.906406597	0.023881593	1.383699665
GOTERM_BP_FAT	GO:0000280~nuclear division	26	1.21325245	0.0007886	Q2YDJ0, Q2TA25, Q32PA5, A6QM04, Q3ZBK3, Q17QG2, Q0P5L6, P48734, Q3SZP2, Q2NKY7, Q58D97, Q08DR9, A6H7B3, Q3B7M6, Q1JQB2, Q2TBN3, Q24JY3, Q3SZY3, Q05B84, P62285, Q3T054, A7YY75, Q0P5A1, Q3ZBD9, Q3ZCF0, Q1LZG6	1472	79	8785	1.964175151	0.911179242	0.024159285	1.414055902
GOTERM_BP_FAT	GO:0007067~mitosis	26	1.21325245	0.0007886	Q2YDJ0, Q2TA25, Q32PA5, A6QM04, Q3ZBK3, Q17QG2, Q0P5L6, P48734, Q3SZP2, Q2NKY7, Q58D97, Q08DR9, A6H7B3, Q3B7M6, Q1JQB2, Q2TBN3, Q24JY3, Q3SZY3, Q05B84, P62285, Q3T054, A7YY75, Q0P5A1, Q3ZBD9, Q3ZCF0, Q1LZG6	1472	79	8785	1.964175151	0.911179242	0.024159285	1.414055902
GOTERM_BP_FAT	GO:0032956~regulation of actin cytoskeleton organization	14	0.653289781	0.0008063	Q32LP2, Q5E997, A4FUA8, Q5E963, Q3T035, Q1LZB2, Q3MHR7, P61585, Q5E9D5, Q58CQ2, Q1JP79, P61157, Q3SYX9, P62998	1472	31	8785	2.695257714	0.915892853	0.024452705	1.445671985
GOTERM_BP_FAT	GO:0006397~mRNA processing	39	1.819878675	0.0009125	Q17QI2, Q5E959, Q3MHX3, Q24JZ8, Q2KJJ0, Q5E992, Q3ZBV3, Q5E9Z8, Q3SZR8, Q3ZBK6, A5D7H5, P09867, Q3ZCE0, Q0P5D0, Q0P5D2, Q2HJ60, P61286, A1A4K8, Q32PE9, Q29RH4, Q3ZCA2, Q3T147, Q5E9J1, Q3T0D0, P67808, A0JN52, Q3T106, Q3B8S0, Q3SYR3, Q3MHR5, P79101, Q2KIR1, Q3B7L6, Q3MHE2, Q2KJ71, Q2NL22, Q10568, Q1RMR2, Q0VCY7	1472	138	8785	1.686628663	0.939293969	0.027358512	1.634505005
GOTERM_BP_FAT	GO:0009309~amine biosynthetic process	20	0.933271115	0.0010147	Q2NL31, Q58DW2, Q5EA40, Q2KJH7, A7E3T7, A4IFQ7, Q0VD27, Q2KHU0, A7YWE4, P33097, Q1LZA3, P12344, Q3T0G8, Q58DT4, A0JNI4, Q58D08, Q4JIJ3, Q3SZA5, P00376, Q3SZJ0	1472	55	8785	2.17020751	0.955650124	0.030083742	1.815979438
GOTERM_BP_FAT	GO:0032446~protein modification by small protein conjugation	20	0.933271115	0.0010147	Q3MHX6, Q0VCL3, Q1RMX2, P63048, A3KN22, A1A4K3, Q1RMW1, Q32PA5, Q0VCP5, A7MBJ5, A2VDP1, Q32LN1, Q2HJI9, P62992, Q08E63, Q32PF3, P61955, Q0P5I7, Q3ZCC3, Q0P5F4	1472	55	8785	2.17020751	0.955650124	0.030083742	1.815979438
GOTERM_BP_FAT	GO:0009144~purine nucleoside triphosphate metabolic process	30	1.399906673	0.0010574	P00570, A5PK70, Q00361, P11019, O97681, Q2HJ33, P31408, Q3T0C6, P32876, P31404, P13620, P13621, P57709, P40682, Q08DA1, P05631, P22027, P05630, P52174, P52175, Q3T0Q4, P21282, Q0VCY0, P56658, P13619, Q59HJ6, P15690, Q28851, Q2KJB6, P00829, Q28852, P61420	1472	98	8785	1.826960404	0.9611105	0.031032854	1.891829724
GOTERM_BP_FAT	GO:0022403~cell cycle phase	39	1.819878675	0.0010576	Q2YDJ0, Q2TA25, Q32PA5, A6QM04, Q3ZBK3, Q17QG2, Q0P5L6, P48734, Q0II23, Q3SZP2, Q2NKY7, Q2HJG1, Q58D97, Q08DR9, A5D7V1, Q3B7M6, A6H7B3, Q0VCV0, Q1RMH9, Q1JQB2, Q3T0M7, P53712, Q2TBN3, Q3HNG7, Q24JY3, Q3SZY3, Q2KJD0, Q05B84, Q2KJI1, Q862I1, Q5E964, P62285, Q3T054, A7YY75, Q0P5A1, Q08DQ9, Q3ZBD9, Q3ZCF0, Q1LZG6	1472	139	8785	1.674494643	0.961132606	0.030744394	1.892157854
GOTERM_BP_FAT	GO:0032970~regulation of actin filament-based process	14	0.653289781	0.0011523	Q32LP2, Q5E997, A4FUA8, Q5E963, Q3T035, Q1LZB2, Q3MHR7, P61585, Q5E9D5, Q58CQ2, Q1JP79, P61157, Q3SYX9, P62998	1472	32	8785	2.61103091	0.970943965	0.033138748	2.05990546
GOTERM_BP_FAT	GO:0006695~cholesterol biosynthetic process	10	0.466635558	0.0011556	P07514, Q5E9T8, Q8WMY2, A6QR14, Q8WMV1, Q3ZBE9, Q1LZ95, A4FUD2, P84466, Q5E9J5	1472	18	8785	3.315594807	0.971235288	0.032923247	2.065710561
GOTERM_BP_FAT	GO:0006997~nucleus organization	10	0.466635558	0.0011556	A6QNZ5, Q95KV7, P45478, A7YY75, Q0VC37, P00442, P38447, O02703, Q08DY9, Q58CZ0	1472	18	8785	3.315594807	0.971235288	0.032923247	2.065710561
GOTERM_BP_FAT	GO:0065003~macromolecular complex assembly	70	3.266448903	0.001211	Q02399, Q3T0N9, Q0II87, Q58D72, Q0P5L6, A1A4I8, Q2HJI2, Q0IIG5, A6H767, P84227, P18246, Q32S29, Q32L01, Q5E9H5, A6QPI6, A2VDN5, Q17QJ0, P80177, Q3MHM5, A7YWD2, Q862I1, Q0VCI6, P42028, Q2KJF9, Q2HJ86, P48427, Q647I9, Q3ZBD3, A1L567, Q3ZC55, Q2TA40, A0JNQ6, Q17QG8, Q5EA65, Q2T9S0, Q1RMV0, Q08E52, Q9TU47, Q3T122, Q17Q89, P52193, A6H784, A5D9D4, A6QR05, A1L5A6, A6QNT3, Q3SZ18, Q32KM1, Q0P5F2, Q5E9F8, Q3HNG7, P01096, Q9TRY0, P00432, Q2KJD0, Q3ZCJ7, Q76LV2, P81947, P79132, A6QLI8, Q02375, A0JNI4, Q08DE0, Q59HJ6, O02703, A1A4J8, A4FUH5, Q148J6, Q2NL24, Q95107	1472	291	8785	1.43561837	0.975739606	0.034159111	2.163767657
GOTERM_BP_FAT	GO:0016054~organic acid catabolic process	21	0.979934671	0.0012565	Q0IIL6, Q5EA40, P25285, Q3SZ00, Q1RMV0, P00366, Q3MHL4, Q3ZCJ2, Q3SZP5, A4IFQ7, O46504, P20821, P33097, P12344, Q0NXR6, P21839, A4FUD0, Q2KIG0, Q2T9V8, Q2HJ73, Q3SZB4	1472	60	8785	2.088824728	0.978901808	0.035096788	2.244106011
GOTERM_BP_FAT	GO:0046395~carboxylic acid catabolic process	21	0.979934671	0.0012565	Q0IIL6, Q5EA40, P25285, Q3SZ00, Q1RMV0, P00366, Q3MHL4, Q3ZCJ2, Q3SZP5, A4IFQ7, O46504, P20821, P33097, P12344, Q0NXR6, P21839, A4FUD0, Q2KIG0, Q2T9V8, Q2HJ73, Q3SZB4	1472	60	8785	2.088824728	0.978901808	0.035096788	2.244106011
GOTERM_BP_FAT	GO:0008219~cell death	54	2.519832011	0.001259	Q95KV7, Q2KIT8, Q5E9L4, Q5E9C2, A0JNK3, P56541, Q3T0K8, Q58CZ0, P45478, Q5E9M9, Q645M6, Q0VC36, Q29RZ9, P43481, Q2HJF8, Q0VC37, Q0II48, Q2HJF6, Q0P5M9, P15690, Q08DY9, Q0V882, Q3SX43, P00442, Q2KJE0, Q3T0P5, Q3ZBT1, Q32KL9, Q32LM0, Q0II26, Q32LE4, Q3T0I5, P38447, Q5E9J6, Q3ZBG5, Q29RU4, Q2NL36, Q0VCX4, P82922, P45879, Q0P5F4, A6QQW8, P00435, A7E3S4, P32007, Q3T169, Q1JQC5, A4IF78, Q08DE0, Q2HJH9, Q5EAC7, O02703, Q2NL24, Q0IIH8	1472	212	8785	1.52016894	0.979062163	0.034848023	2.248492977
GOTERM_BP_FAT	GO:0016265~death	55	2.566495567	0.0012747	Q95KV7, Q2KIT8, Q5E9L4, Q5E9C2, A0JNK3, P56541, Q3T0K8, Q58CZ0, P45478, Q5E9M9, Q645M6, Q0VC36, Q2KJI7, Q29RZ9, P43481, Q2HJF8, Q0VC37, Q0II48, Q2HJF6, Q0P5M9, P15690, Q08DY9, Q0V882, Q3SX43, P00442, Q2KJE0, Q3T0P5, Q3ZBT1, Q32KL9, Q32LM0, Q0II26, Q32LE4, Q3T0I5, P38447, Q5E9J6, Q3ZBG5, Q29RU4, Q2NL36, Q0VCX4, P82922, P45879, Q0P5F4, A6QQW8, P00435, A7E3S4, P32007, Q3T169, Q1JQC5, A4IF78, Q08DE0, Q2HJH9, Q5EAC7, O02703, Q2NL24, Q0IIH8	1472	217	8785	1.512644635	0.980050332	0.034960989	2.276287116
GOTERM_BP_FAT	GO:0006888~ER to Golgi vesicle-mediated transport	9	0.419972002	0.001284	Q17QI1, A2VDN5, Q17QI5, Q3SZN2, Q3T0F2, Q3T000, Q0P5L6, A6QNT8, Q2TBL9	1472	15	8785	3.580842391	0.980611239	0.034899539	2.292679157
GOTERM_BP_FAT	GO:0042375~quinone cofactor metabolic process	8	0.373308446	0.0013186	Q3SZD7, Q3T131, Q58CW1, Q6B4J2, Q2KIL4, Q0P5A2, A6QR05, Q2NL34	1472	12	8785	3.978713768	0.982567199	0.035509574	2.353776816
GOTERM_BP_FAT	GO:0000278~mitotic cell cycle	33	1.53989734	0.0013587	Q2YDJ0, Q2TA06, Q2TA25, Q32PA5, A6QM04, Q3ZBK3, Q17QG2, Q0P5L6, P48734, Q3SZP2, Q2NKY7, Q58D97, Q08DR9, A5D7V1, Q3B7M6, A6H7B3, Q1RMH9, Q1JQB2, P53712, Q2TBN3, Q24JY3, Q3SZY3, Q05B84, Q862I1, P62285, Q3T054, A7YY75, Q0P5A1, Q08DQ9, Q3ZBD9, Q3ZCF0, Q0P5I7, Q1LZG6	1472	113	8785	1.74288789	0.984589765	0.036253278	2.424583376
GOTERM_BP_FAT	GO:0009064~glutamine family amino acid metabolic process	14	0.653289781	0.0016137	A7YWG4, Q58DL1, P00366, Q2KJH7, P33097, A7YWE4, Q1LZA3, P12344, Q58DT4, Q32S38, Q2T9Y6, Q58D08, Q1RMS2, Q3SZJ0	1472	33	8785	2.531908762	0.992961905	0.042545758	2.87335245

Category	Term	Count	%	PValue	Genes	List Total	Pop Hits	Pop Total	Fold Enrichment	Bonferroni	Benjamini	FDR
GOTERM_BP_FAT	GO:0033554~cellular response to stress	58	2.706486234	0.001624	A2VDZ9, P40673, P18493, Q71SS4, O02776, A6QLA6, P23196, P48734, A4FV00, Q29RK4, Q3T0G8, Q58DH8, O41515, A4IFC8, Q0VCV0, Q28141, P68102, Q3ZBH9, A5PJN2, Q3SZY3, Q3ZBW4, Q0VC36, A7YY55, P37359, Q9BGI3, Q9BGI1, Q2TBU9, Q08DY9, Q5E9N5, Q3MQ24, Q3MHE4, Q3MHX6, Q58DB5, Q17QG8, A1A4K3, P35705, P00442, Q3ZBT1, Q08E57, Q3MHJ7, Q1LZA3, Q58DT4, Q3MHF7, Q3SZ43, A5D7V1, Q0VCT3, Q2NL37, A6QQW8, P00435, P00432, Q3T169, Q0P5K3, Q27965, Q5E947, A0JNH9, P10103, A6QLT9, Q59HJ6, O02703	1472	234	8785	1.479265375	0.993181184	0.042447297	2.891433854
GOTERM_BP_FAT	GO:0034220~ion transmembrane transport	17	0.793280448	0.0017622	P40682, P05631, P05630, Q00361, P11019, O97681, P21282, P31408, P13619, P32876, P31404, Q2KJB6, P00829, Q28852, P61420, P13620, P13621	1472	45	8785	2.254604469	0.995541364	0.045591057	3.133804443
GOTERM_BP_FAT	GO:0022402~cell cycle process	47	2.193187121	0.0017961	P00570, Q2YDJ0, Q2TA25, Q32PA5, Q3ZBK3, A6QM04, Q17QG2, P48734, Q0P5L6, Q0II23, Q3SZP2, Q2KI13, Q2NKY7, Q2HJG1, Q58D97, P52193, Q08DR9, A5D7V1, Q3B7M6, A6H7B3, Q0VCV0, Q1RMH9, Q1JQB2, Q3T0M7, P53712, Q0VCX4, Q2TBN3, Q3HNG7, Q24JY3, Q3SZY3, Q2KJD0, Q05B84, Q2KJI1, Q862I1, Q5E964, P62285, Q3SWZ6, Q3T054, P79126, A7YY75, Q08DE0, Q0P5A1, Q08DQ9, Q3ZBD9, Q27991, Q3ZCF0, Q1LZG6	1472	181	8785	1.549720004	0.995982974	0.046061115	3.193215094
GOTERM_BP_FAT	GO:0030833~regulation of actin filament polymerization	12	0.559962669	0.0018597	Q32LP2, Q5E997, Q1LZB2, Q3MHR7, A4FUA8, Q58CQ2, Q5E963, Q3SYX9, P61157, Q1JP79, Q3T035, P62998	1472	26	8785	2.754494147	0.99669612	0.04725916	3.304443388
GOTERM_BP_FAT	GO:0022411~cellular component disassembly	10	0.466635558	0.001872	P45478, Q0VC37, P00442, P38447, A6QNM2, O02703, Q3T116, Q08DY9, Q0VCX5, Q58CZ0	1472	19	8785	3.141089817	0.996819184	0.04717547	3.32603164
GOTERM_BP_FAT	GO:0009161~ribonucleoside monophosphate metabolic process	10	0.466635558	0.001872	Q05927, Q0VCK0, Q2HJ58, P56658, Q59A32, Q2HJ26, A0JNA3, P31754, Q3SWY3, Q0VC13	1472	19	8785	3.141089817	0.996819184	0.04717547	3.32603164
GOTERM_BP_FAT	GO:0051656~establishment of organelle localization	10	0.466635558	0.001872	Q56K10, Q2HJF8, Q2YDJ0, A7YY75, Q08DR9, Q5E9M9, Q3T000, Q5E971, Q27991, A6QLB1	1472	19	8785	3.141089817	0.996819184	0.04717547	3.32603164
GOTERM_BP_FAT	GO:0046034~ATP metabolic process	27	1.259916006	0.0019234	P00570, P40682, Q08DA1, P22027, P05631, P05630, Q00361, P11019, Q2HJ33, O97681, P21282, P31408, Q3T0C6, Q0VCY0, P13619, Q59HJ6, P31404, P32876, P15690, Q2KJB6, Q28851, P00829, Q28852, P61420, P13620, P57709, P13621	1472	88	8785	1.831112586	0.997283802	0.048045161	3.415782593
GOTERM_BP_FAT	GO:0009205~purine ribonucleoside triphosphate metabolic process	29	1.353243117	0.0019338	P00570, A5PK70, Q00361, P11019, O97681, Q2HJ33, P31408, Q3T0C6, P32876, P31404, P13620, P13621, P57709, P40682, Q08DA1, P05631, P22027, P05630, P52174, P52175, Q3T0Q4, P21282, Q0VCY0, P13619, Q59HJ6, P15690, Q28851, Q2KJB6, P00829, Q28852, P61420	1472	97	8785	1.784268545	0.997369676	0.04791053	3.434032595
GOTERM_BP_FAT	GO:0009199~ribonucleoside triphosphate metabolic process	29	1.353243117	0.0019338	P00570, A5PK70, Q00361, P11019, O97681, Q2HJ33, P31408, Q3T0C6, P32876, P31404, P13620, P13621, P57709, P40682, Q08DA1, P05631, P22027, P05630, P52174, P52175, Q3T0Q4, P21282, Q0VCY0, P13619, Q59HJ6, P15690, Q28851, Q2KJB6, P00829, Q28852, P61420	1472	97	8785	1.784268545	0.997369676	0.04791053	3.434032595
GOTERM_BP_FAT	GO:0034660~ncRNA metabolic process	37	1.726551563	0.0019709	A6QNM8, Q3ZBV8, Q05B50, O02691, A7YW98, Q3T087, A6H7F4, Q2KJG3, A5D7A2, A4FUC8, Q2KIF8, Q56JX6, Q3MHH4, A7MBC5, Q1JQB6, Q1RML6, A8E4P2, Q0P5H7, Q9GMB8, Q2KJA6, P17248, Q56K10, Q3T090, Q2TBQ0, Q58DW5, Q3T099, A6H7E1, Q0VC30, Q2KI84, Q3SWZ6, A6QLT9, A7MBD4, Q32LK1, A6QLR2, A5D7G0, Q2T9L8, Q2NL22	1472	134	8785	1.647900105	0.997653182	0.048417269	3.498791192
GOTERM_BP_FAT	GO:0030036~actin cytoskeleton organization	26	1.21325245	0.0020842	Q5E997, A4FUA8, Q3ZCK2, A2VDK3, A2VDK6, Q3B7N2, Q1LZB2, P52193, P63258, Q2TA49, A6H720, A5D7D1, Q0VCF1, P53712, A5D7R9, P57709, Q32L92, Q3ZCL5, P31976, Q09430, Q3SYV4, P61585, Q27991, Q148J6, Q95107, Q1RMT6	1472	84	8785	1.847259964	0.998343342	0.050724796	3.696272307
GOTERM_BP_FAT	GO:0007031~peroxisome organization	9	0.419972002	0.0021935	A1L567, Q0VCP2, Q2KII7, Q148K5, Q1RMV0, Q3SX23, Q3SZD1, P07857, A6QPK4	1472	16	8785	3.357039742	0.998816462	0.052898267	3.886585499
GOTERM_BP_FAT	GO:0043254~regulation of protein complex assembly	14	0.653289781	0.0022179	Q32LP2, Q5E997, A4FUA8, Q5E963, Q3T035, Q1LZB2, P10103, Q3MHR7, Q3ZBD9, Q58CQ2, Q1JP79, P61157, Q3SYX9, P62998	1472	34	8785	2.457440857	0.99890208	0.053055412	3.929028969
GOTERM_BP_FAT	GO:0051493~regulation of cytoskeleton organization	18	0.839944004	0.0022372	Q32LP2, Q5E997, A4FUA8, Q5E963, Q3T035, Q3SWZ6, Q1LZB2, Q3MHR7, P61585, Q5E9D5, Q3ZBD9, Q3T0M7, Q58CQ2, Q3SYX9, Q0VCX4, Q1JP79, P61157, P62998	1472	50	8785	2.148505435	0.998965296	0.053091393	3.962535186
GOTERM_BP_FAT	GO:0009141~nucleoside triphosphate metabolic process	31	1.446570229	0.0022384	P00570, Q2NKU1, A5PK70, Q00361, P11019, O97681, Q2HJ33, P31408, Q3T0C6, P32876, P31404, P13620, P13621, P57709, P40682, Q08DA1, P05631, P22027, P05630, P52174, P52175, Q3T0Q4, P21282, Q0VCY0, P56658, P13619, Q59HJ6, P15690, Q2KJB6, Q28851, P00829, Q28852, P61420	1472	107	8785	1.729067198	0.9989691	0.052712038	3.964615998
GOTERM_BP_FAT	GO:0009108~coenzyme biosynthetic process	17	0.793280448	0.0022892	Q3ZBS0, Q5EAC2, Q1JQD7, Q3T131, Q32S38, Q0II59, Q2T9Y6, Q58CW1, A4FUD0, Q5E9T4, Q0P5C2, Q2KIL4, Q4JIJ3, Q5BIP7, Q0P5A2, A6QR05, Q2NL34	1472	46	8785	2.205591328	0.999118239	0.053467452	4.052849555
GOTERM_BP_FAT	GO:0009116~nucleoside metabolic process	16	0.746616892	0.0023104	Q2NKU1, Q3ZBS0, A5PK70, Q05927, P35705, Q2HJ58, P52174, A7E3T7, A6H7F4, P52175, Q3T0Q4, Q56JW4, A2VDS0, Q3MHF7, P56658, P31754, Q3SZ18, Q08DW2	1472	42	8785	2.273550725	0.999174031	0.05354386	4.089732147
GOTERM_BP_FAT	GO:0033043~regulation of organelle organization	24	1.119925338	0.0024329	Q32LP2, Q2YDJ0, Q5E997, A4FUA8, Q0P5K3, Q5E963, Q3T035, Q3T0G8, Q3SWZ6, Q1LZB2, Q3MHR7, Q0VCT3, P61585, Q5E9D5, Q3ZBD9, Q3T0M7, Q58CQ2, P11023, Q3SYX9, P61157, Q0VCX4, Q2NL24, Q1JP79, P62998	1472	76	8785	1.88465389	0.999433217	0.055881983	4.301947867
GOTERM_BP_FAT	GO:0008637~apoptotic mitochondrial changes	8	0.373308446	0.0024458	P00435, Q0VC36, O02703, Q08DY9, P82922, Q32LM0, Q32LE4, Q0P5F4	1472	13	8785	3.672658863	0.999455333	0.055754403	4.324350849
GOTERM_BP_FAT	GO:0009081~branched chain family amino acid metabolic process	7	0.32664489	0.0024837	Q07536, Q5EA40, Q0NXR6, P21839, Q2HJ73, A4IFQ7, Q9TUM3	1472	10	8785	4.177649457	0.999515316	0.05617856	4.389991605
GOTERM_BP_FAT	GO:0006921~cell structure disassembly during apoptosis	7	0.32664489	0.0024837	P45478, Q0VC37, P00442, P38447, O02703, Q08DY9, Q58CZ0	1472	10	8785	4.177649457	0.999515316	0.05617856	4.389991605
GOTERM_BP_FAT	GO:0006309~DNA fragmentation involved in apoptosis	7	0.32664489	0.0024837	P45478, Q0VC37, P00442, P38447, O02703, Q08DY9, Q58CZ0	1472	10	8785	4.177649457	0.999515316	0.05617856	4.389991605
GOTERM_BP_FAT	GO:0032271~regulation of protein polymerization	13	0.606626225	0.0028822	Q32LP2, Q5E997, A4FUA8, Q5E963, Q3T035, Q1LZB2, Q3MHR7, Q3ZBD9, Q58CQ2, Q1JP79, P61157, Q3SYX9, P62998	1472	31	8785	2.502739306	0.999857815	0.064434755	5.077212091
GOTERM_BP_FAT	GO:0030029~actin filament-based process	26	1.21325245	0.0029682	Q5E997, A4FUA8, Q3ZCK2, A2VDK3, A2VDK6, Q3B7N2, Q1LZB2, P52193, P63258, Q2TA49, A6H720, A5D7D1, Q0VCF1, P53712, A5D7R9, P57709, Q32L92, Q3ZCL5, P31976, Q09430, Q3SYV4, P61585, Q27991, Q148J6, Q95107, Q1RMT6	1472	86	8785	1.80430043	0.999890882	0.065816749	5.224888123
GOTERM_BP_FAT	GO:0042592~homeostatic process	80	3.733084461	0.0031431	Q02399, P18493, Q1LZ90, P23196, Q95KV0, Q3T0G8, A6QNZ5, Q9MYM4, Q0IIG5, P56541, A5PJI5, Q0VCV0, Q2KIL5, A6QL97, Q95KU9, Q9TTJ5, P57709, Q0Z7W6, Q5E9M8, P45478, Q0VCM4, Q3SZK8, Q5E9M9, Q3ZCL8, Q58DA7, Q5E9B4, Q2TBU2, Q29RL9, Q2KJI7, A7MB44, Q9TUM3, P33097, Q05B87, P37359, Q9BGI2, Q2HJF8, Q9BGI3, O46414, Q9BGI1, O46415, P15690, A6H732, Q08DY9, Q5E936, Q5EA61, Q0V8R6, Q5E995, P00442, P35705, Q3T0E0, Q3T058, A6QLU8, Q58D97, Q3MHF7, Q148D3, A6H784, Q0VCT3, A4FV58, Q9N2I8, Q0VCX4, P00435, Q5E946, Q27965, A6QLZ6, Q5E947, P79132, A3KN17, P10575, A5D7E8, Q32S38, O77834, Q3T0L2, Q0VCU1, Q0V8B6, P19035, P00829, O02703, A1A4J8, P20072, P18203	1472	353	8785	1.352537258	0.999936302	0.069064826	5.524497273
GOTERM_BP_FAT	GO:0006631~fatty acid metabolic process	32	1.493233784	0.0032043	Q3T0R7, Q58DK1, Q58DM8, Q1RMV0, P48818, Q5E956, Q0VCA7, O46629, Q3SZP5, Q2KJG4, O19094, Q7YS70, Q3T0G8, P58108, P52505, A5PJC5, P80177, Q0IIL6, Q58DN7, Q3SZ00, O77774, P31322, A6H7G3, Q5EAD4, Q9TUM3, Q3MHR0, Q2KIG0, A4IFM2, Q2KJB7, Q3ZBF7, Q3ZBY7, Q3SZB4	1472	114	8785	1.675247902	0.999947235	0.069863611	5.629086875
GOTERM_BP_FAT	GO:0045116~protein neddylation	5	0.233317779	0.0033937	A3KN22, Q1RMW1, Q0P5I7, Q2HJI9, Q0P5F4	1472	5	8785	5.968070652	0.999970552	0.073325099	5.952279209
GOTERM_BP_FAT	GO:0044272~sulfur compound biosynthetic process	11	0.513299113	0.0034131	Q2NL31, Q58DW2, Q32S38, Q2T9Y6, Q5E9T4, Q4JIJ3, Q5BIP7, A7E3T7, Q0VD27, Q5EAC2, O18756	1472	24	8785	2.735365716	0.999972262	0.073215408	5.985365101
GOTERM_BP_FAT	GO:0009156~ribonucleoside monophosphate biosynthetic process	9	0.419972002	0.0035407	Q0VCK0, Q2HJ58, P56658, Q59A32, Q2HJ26, A0JNA3, P31754, Q3SWY3, Q0VC13	1472	17	8785	3.159566816	0.999981274	0.075325528	6.202376193

Category	Term	Count	%	PValue	Genes	List Total	Pop Hits	Pop Total	Fold Enrichment	Bonferroni	Benjamini	FDR
GOTERM_BP_FAT	GO:0051640~organelle localization	12	0.559962669	0.0037413	Q56K10, Q2HJF8, Q2YDJ0, A7YY75, Q08DR9, Q5E9M9, Q3T000, Q5E971, A5D9D4, Q27991, P62285, A6QLB1	1472	28	8785	2.557744565	0.999989905	0.078882643	6.542681352
GOTERM_BP_FAT	GO:0009126~purine nucleoside monophosphate metabolic process	8	0.373308446	0.0041898	Q05927, Q0VCK0, P56658, Q59A32, Q2HJ26, A0JNA3, Q3SWY3, Q0VC13	1472	14	8785	3.410326087	0.999997466	0.087335386	7.299353413
GOTERM_BP_FAT	GO:0042542~response to hydrogen peroxide	8	0.373308446	0.0041898	Q9BGI3, Q3MHF7, P00435, P00442, P35705, P00432, Q5E946, Q5E947, Q0P594	1472	14	8785	3.410326087	0.999997466	0.087335386	7.299353413
GOTERM_BP_FAT	GO:0034614~cellular response to reactive oxygen species	8	0.373308446	0.0041898	Q9BGI3, Q3MHF7, P00435, Q9BGI1, P00442, P35705, P00432, Q5E947, P37359	1472	14	8785	3.410326087	0.999997466	0.087335386	7.299353413
GOTERM_BP_FAT	GO:0009167~purine ribonucleoside monophosphate metabolic process	8	0.373308446	0.0041898	Q05927, Q0VCK0, P56658, Q59A32, Q2HJ26, A0JNA3, Q3SWY3, Q0VC13	1472	14	8785	3.410326087	0.999997466	0.087335386	7.299353413
GOTERM_BP_FAT	GO:0034622~cellular macromolecular complex assembly	47	2.193187121	0.0049267	Q2TA40, Q02399, Q0II87, A0JNQ6, Q17QG8, Q2T9S0, Q08E52, A1A4I8, Q2HJI2, Q9TU47, Q3T122, Q17Q89, P52193, A6H784, A6H767, A5D9D4, A1L5A6, P84227, A6QNT3, Q32KM1, Q32S29, Q5E9H5, Q0P5F2, Q32L01, Q5E9F8, Q3HNG7, A6QPI6, Q17QJ0, Q9TRY0, Q3MHM5, A7YWD2, Q2KJD0, Q3ZCJ7, Q76LV2, Q862I1, P81947, Q02375, Q0VCI6, Q08DE0, P42028, Q2HJ86, P48427, A1A4J8, A4FUH5, Q148J6, Q2NL24, Q647I9	1472	190	8785	1.476312214	0.999999739	0.101242245	8.529872265
GOTERM_BP_FAT	GO:0006396~RNA processing	62	2.893140457	0.0050636	Q17QI2, Q3ZBV3, Q3T087, Q5E9Z8, Q3SZR8, A5D7H5, Q1JQB6, Q3ZCE0, Q0P5D0, Q0P5D2, Q2HJ60, P61286, Q56K10, Q32PE9, Q29RH4, Q3ZCA2, Q3T147, Q3T090, Q2TBQ0, Q5E9J1, Q2HJ92, P67808, A0JN52, Q3T106, Q3B8S0, P79101, Q2KIR1, Q3MHE2, Q2KJ71, Q5E9E6, Q2HJ88, Q10568, Q2NL22, Q1RMR2, A8E4M9, Q5E959, Q3MHX3, Q05B50, Q24JZ8, Q2KJJ0, Q5E992, O02691, A6H7F4, Q3ZBK6, Q56JX6, P09867, A5D7N5, A6QPQ5, Q1RML6, P10881, Q2KIS2, Q2KJA6, A1A4K8, Q58DW5, Q3T0D0, Q3SWZ6, Q3SYR3, Q3MHR5, A6QLT9, A7MBD2, Q3B7L6, Q0VCY7	1472	266	8785	1.391054062	0.999999829	0.103223804	8.756844209
GOTERM_BP_FAT	GO:0043069~negative regulation of programmed cell death	38	1.773215119	0.006384	Q9XT96, Q0VCJ2, Q0V882, P00442, P35705, P23727, Q5E9C2, P48734, Q1LZA3, Q3T0G8, Q29455, Q3MHF7, A5D7R9, Q0P5F4, Q95M18, P80177, Q0Z7W6, Q5E984, P45478, P00435, P00432, Q27965, Q17QR2, Q3MHM6, Q9TUM3, P43481, Q0P594, Q32S38, Q9BGI3, Q9BGI1, Q0VC37, A6QLT9, P56658, Q5EAC7, P61585, O02703, Q0VCY8, Q08DY9, Q3MQ24	1472	148	8785	1.532342465	0.999999997	0.127589963	10.91827852
GOTERM_BP_FAT	GO:0060548~negative regulation of cell death	38	1.773215119	0.006384	Q9XT96, Q0VCJ2, Q0V882, P00442, P35705, P23727, Q5E9C2, P48734, Q1LZA3, Q3T0G8, Q29455, Q3MHF7, A5D7R9, Q0P5F4, Q95M18, P80177, Q0Z7W6, Q5E984, P45478, P00435, P00432, Q27965, Q17QR2, Q3MHM6, Q9TUM3, P43481, Q0P594, Q32S38, Q9BGI3, Q9BGI1, Q0VC37, A6QLT9, P56658, Q5EAC7, P61585, O02703, Q0VCY8, Q08DY9, Q3MQ24	1472	148	8785	1.532342465	0.999999997	0.127589963	10.91827852
GOTERM_BP_FAT	GO:0009066~aspartate family amino acid metabolic process	8	0.373308446	0.0067324	Q2NL31, Q5I598, Q4JIJ3, Q0VD27, P31228, P33097, Q1LZA3, P12344	1472	15	8785	3.182971014	0.999999999	0.133225852	11.48044646
GOTERM_BP_FAT	GO:0000226~microtubule cytoskeleton organization	19	0.886607559	0.0076122	A2VDN5, Q2YDJ0, Q32KS0, Q3ZBK3, Q2KJD0, A0JNH5, Q0II23, P38584, A0JNH7, P81947, Q2HJG1, A6QNZ5, A7YY75, Q08DR9, Q3T0M7, A5D7P5, Q0VCX4, Q2TBN3, Q32P97	1472	60	8785	1.88988904	1	0.148387931	12.88517976
GOTERM_BP_FAT	GO:0008283~cell proliferation	30	1.399906673	0.0078249	Q08DD6, Q58DV0, A6QNZ5, Q0II59, A0JNA3, Q2TA22, P62871, P23356, Q3SWY3, P61223, P25930, P80177, P00435, O77774, Q3ZBH9, Q5E947, Q2T9P4, Q0VC36, Q6EWQ7, P62285, Q3SWZ6, Q9BGI3, Q2HJF6, O02703, Q3ZCF0, Q27991, Q3SZ07, P20072, P18203	1472	111	8785	1.612992068	1	0.151263514	13.22161523
GOTERM_BP_FAT	GO:0046128~purine ribonucleoside metabolic process	7	0.32664489	0.0080688	Q56JW4, Q3ZBS0, Q05927, P56658, A7E3T7, A6H7F4, Q3SZ18	1472	12	8785	3.481374547	1	0.154643294	13.60591134
GOTERM_BP_FAT	GO:0006458~'de novo' protein folding	7	0.32664489	0.0080688	Q3MI00, A1A4M8, P19120, A5PK75, A5PJN2, Q0IIM3, Q32LM0	1472	12	8785	3.481374547	1	0.154643294	13.60591134
GOTERM_BP_FAT	GO:0009168~purine ribonucleoside monophosphate biosynthetic process	7	0.32664489	0.0080688	Q0VCK0, P56658, Q59A32, Q2HJ26, A0JNA3, Q3SWY3, Q0VC13	1472	12	8785	3.481374547	1	0.154643294	13.60591134
GOTERM_BP_FAT	GO:0042278~purine nucleoside metabolic process	7	0.32664489	0.0080688	Q56JW4, Q3ZBS0, Q05927, P56658, A7E3T7, A6H7F4, Q3SZ18	1472	12	8785	3.481374547	1	0.154643294	13.60591134
GOTERM_BP_FAT	GO:0051084~'de novo' posttranslational protein folding	7	0.32664489	0.0080688	Q3MI00, A1A4M8, P19120, A5PK75, A5PJN2, Q0IIM3, Q32LM0	1472	12	8785	3.481374547	1	0.154643294	13.60591134
GOTERM_BP_FAT	GO:0009127~purine nucleoside monophosphate biosynthetic process	7	0.32664489	0.0080688	Q0VCK0, P56658, Q59A32, Q2HJ26, A0JNA3, Q3SWY3, Q0VC13	1472	12	8785	3.481374547	1	0.154643294	13.60591134
GOTERM_BP_FAT	GO:0060627~regulation of vesicle-mediated transport	14	0.653289781	0.0085914	Q02399, P45478, Q17QF8, Q2TBH7, Q1RMR9, P10948, Q3T0G8, Q58DS9, Q95114, P52193, Q08E63, A5D7D1, P19035, P11023	1472	39	8785	2.142384337	1	0.162826207	14.42390834
GOTERM_BP_FAT	GO:0006308~DNA catabolic process	10	0.466635558	0.008785	P45478, Q3SZ43, Q0VC37, P00442, P56541, P38447, Q0P5K3, O02703, Q08DY9, Q58CZ0	1472	23	8785	2.594813327	1	0.165176621	14.72512293
GOTERM_BP_FAT	GO:0006120~mitochondrial electron transport, NADH to ubiquinone	5	0.233317779	0.0088289	Q02372, Q05752, Q02827, Q6QTH0, P52505	1472	6	8785	4.97339221	1	0.164930449	14.79332074
GOTERM_BP_FAT	GO:0009083~branched chain family amino acid catabolic process	5	0.233317779	0.0088289	Q5EA40, Q0NXR6, P21839, Q2HJ73, A4IFQ7	1472	6	8785	4.97339221	1	0.164930449	14.79332074
GOTERM_BP_FAT	GO:0065002~intracellular protein transmembrane transport	5	0.233317779	0.0088289	Q3SZC1, Q1RMV0, Q32L81, Q0P5N5, Q0G846	1472	6	8785	4.97339221	1	0.164930449	14.79332074
GOTERM_BP_FAT	GO:0043066~negative regulation of apoptosis	37	1.726551563	0.0088815	Q9XT96, Q0VCJ2, Q0V882, P00442, P35705, P23727, Q5E9C2, P48734, Q1LZA3, Q3T0G8, Q29455, Q3MHF7, A5D7R9, Q0P5F4, Q95M18, P80177, Q0Z7W6, Q5E984, P45478, P00435, P00432, Q27965, Q17QR2, Q3MHM6, Q9TUM3, Q0P594, Q32S38, Q9BGI3, Q9BGI1, Q0VC37, A6QLT9, P56658, Q5EAC7, P61585, O02703, Q0VCY8, Q08DY9, Q3MQ24	1472	146	8785	1.512456261	1	0.164834926	14.87490395
GOTERM_BP_FAT	GO:0015931~nucleobase, nucleoside, nucleotide and nucleic acid transport	13	0.606626225	0.0089149	A5D7H5, Q29RH4, P09867, A7YY75, Q3ZBB0, Q3ZBV3, Q3ZBV2, A5PJZ5, Q3ZC83, P18246, Q2NL22, Q2TBP1, Q6EWQ7	1472	35	8785	2.216711957	1	0.164416442	14.92670255
GOTERM_BP_FAT	GO:0006800~oxygen and reactive oxygen species metabolic process	13	0.606626225	0.0089149	P00435, O77774, P35705, P00442, P00432, Q5E946, Q5E947, Q6QTH0, P37359, Q9BGI3, Q02375, Q3MHF7, P15690, P41976	1472	35	8785	2.216711957	1	0.164416442	14.92670255
GOTERM_BP_FAT	GO:0006744~ubiquinone biosynthetic process	6	0.279981335	0.0090978	Q3T131, Q58CW1, Q2KIL4, Q0P5A2, A6QR05, Q2NL34	1472	9	8785	3.978713768	1	0.166511356	15.20956945
GOTERM_BP_FAT	GO:0033108~mitochondrial respiratory chain complex assembly	6	0.279981335	0.0090978	Q2HJI2, Q02375, Q0II87, P42028, A4FUH5, Q5E9H5	1472	9	8785	3.978713768	1	0.166511356	15.20956945
GOTERM_BP_FAT	GO:0006743~ubiquinone metabolic process	6	0.279981335	0.0090978	Q3T131, Q58CW1, Q2KIL4, Q0P5A2, A6QR05, Q2NL34	1472	9	8785	3.978713768	1	0.166511356	15.20956945
GOTERM_BP_FAT	GO:0045426~quinone cofactor biosynthetic process	6	0.279981335	0.0090978	Q3T131, Q58CW1, Q2KIL4, Q0P5A2, A6QR05, Q2NL34	1472	9	8785	3.978713768	1	0.166511356	15.20956945
GOTERM_BP_FAT	GO:0010970~microtubule-based transport	6	0.279981335	0.0090978	Q2HJF8, Q08DH4, A7E2Z6, Q5E9M9, A6H7H2, P23356	1472	9	8785	3.978713768	1	0.166511356	15.20956945
GOTERM_BP_FAT	GO:0009206~purine ribonucleoside triphosphate biosynthetic process	25	1.166588894	0.0098516	A5PK70, Q00361, P11019, O97681, P31408, Q3T0C6, P31404, P32876, P13620, P13621, P57709, P40682, Q08DA1, P05631, P22027, P05630, P52174, P52175, Q3T0Q4, P21282, Q0VCY0, P13619, Q28851, Q2KJB6, P00829, P61420, Q28852	1472	89	8785	1.67642434	1	0.178011	16.36646606

Category	Term	Count	%	PValue	Genes	List Total	Pop Hits	Pop Total	Fold Enrichment	Bonferroni	Benjamini	FDR
GOTERM_BP_FAT	GO:0009145~purine nucleoside triphosphate biosynthetic process	25	1.166588894	0.0098516	A5PK70, Q00361, P11019, O97681, P31408, Q3T0C6, P31404, P32876, P13620, P13621, P57709, P40682, Q08DA1, P05631, P22027, P05630, P52174, P52175, Q3T0Q4, P21282, Q0VCY0, P13619, Q28851, Q2KJB6, P00829, P61420, Q28852	1472	89	8785	1.67642434	1	0.178011	16.36646606
GOTERM_BP_FAT	GO:0009201~ribonucleoside triphosphate biosynthetic process	25	1.166588894	0.0098516	A5PK70, Q00361, P11019, O97681, P31408, Q3T0C6, P31404, P32876, P13620, P13621, P57709, P40682, Q08DA1, P05631, P22027, P05630, P52174, P52175, Q3T0Q4, P21282, Q0VCY0, P13619, Q28851, Q2KJB6, P00829, P61420, Q28852	1472	89	8785	1.67642434	1	0.178011	16.36646606
GOTERM_BP_FAT	GO:0006694~steroid biosynthetic process	14	0.653289781	0.0108219	P07514, Q8WMY2, Q8WMV1, Q1LZ95, A4FUD2, P84466, Q5E9T8, Q58DH7, A6QR14, Q1JQD0, Q3ZBE9, Q3ZCD7, Q5E9J5, Q5E9H7	1472	40	8785	2.088824728	1	0.192699667	17.83366031
GOTERM_BP_FAT	GO:0009063~cellular amino acid catabolic process	14	0.653289781	0.0108219	Q5EA40, P25285, P00366, Q3MHL4, A4IFQ7, O46504, P20821, P33097, P12344, Q0NXR6, P21839, A4FUD0, Q2T9V8, Q2HJ73	1472	40	8785	2.088824728	1	0.192699667	17.83366031
GOTERM_BP_FAT	GO:0009142~nucleoside triphosphate biosynthetic process	25	1.166588894	0.0113599	A5PK70, Q00361, P11019, O97681, P31408, Q3T0C6, P31404, P32876, P13620, P13621, P57709, P40682, Q08DA1, P05631, P22027, P05630, P52174, P52175, Q3T0Q4, P21282, Q0VCY0, P13619, Q28851, Q2KJB6, P00829, P61420, Q28852	1472	90	8785	1.657797403	1	0.200150206	18.63671741
GOTERM_BP_FAT	GO:0046148~pigment biosynthetic process	9	0.419972002	0.0114812	Q56JW4, Q2HJ58, P56658, Q59A32, A5PK65, Q1LZ95, Q2KIN5, P22600, Q58DK5	1472	20	8785	2.685631793	1	0.200925442	18.81672842
GOTERM_BP_FAT	GO:0044087~regulation of cellular component biogenesis	16	0.746616892	0.0116779	Q32LP2, Q5E997, A4FUA8, Q3ZCK2, Q5E963, Q3T035, Q1LZB2, P10103, Q3MHR7, P61585, Q3ZBD9, Q58CQ2, Q3SYX9, Q1JP79, P61157, P62998	1472	49	8785	1.948757764	1	0.202865474	19.10788112
GOTERM_BP_FAT	GO:0051259~protein oligomerization	18	0.839944004	0.01194	A2VDN5, Q3ZC55, P80177, A1L567, Q5EA65, Q1RMV0, P00432, Q58D72, P79132, Q0IIG5, A0JNI4, Q59HJ6, A6QR05, P18246, Q3SZ18, O02703, Q3ZBD3, P01096	1472	58	8785	1.852159858	1	0.205785871	19.49426676
GOTERM_BP_FAT	GO:0009119~ribonucleoside metabolic process	10	0.466635558	0.0120081	Q56JW4, A5PK70, Q3ZBS0, Q05927, P56658, A7E3T7, P52174, A6H7F4, P31754, P52175, Q3SZ18, Q3T0Q4	1472	24	8785	2.486696105	1	0.205693395	19.59443391
GOTERM_BP_FAT	GO:0006754~ATP biosynthetic process	23	1.073261783	0.012035	P40682, Q08DA1, P22027, P05631, P05630, Q00361, P11019, O97681, P21282, P31408, Q3T0C6, Q0VCY0, P13619, P32876, P31404, Q2KJB6, Q28851, P00829, Q28852, P61420, P13620, P57709, P13621	1472	81	8785	1.694637346	1	0.204973365	19.63391672
GOTERM_BP_FAT	GO:0006508~proteolysis	125	5.83294447	0.0120406	Q0VCL3, Q71SS4, P67810, Q3MHZ7, Q3SZN3, A7MBJ5, A0JNK3, Q2KJG2, Q2TBX8, A3KMV5, O41515, A3KMV3, P81425, Q1JPJ8, Q3T0X5, Q3ZC89, A7E2Z6, Q2TA14, Q0P5M8, Q3ZC84, Q2KJI7, Q0IIF7, Q3T108, Q0P594, Q9XSJ0, A6QR14, Q24K02, Q2TBG8, A6QR12, Q1JPJ2, Q3SZ52, Q08DY9, Q58DU5, Q5E9K0, Q3MHX6, Q0IIM6, A3KN22, Q3T0Y5, Q2KI36, Q3ZBT1, Q2T9S7, Q3T0P5, A7MBD9, P62194, Q3T112, Q58DG6, Q0VCX1, Q3SZ43, Q3ZBG0, Q0V8G3, Q32PF3, Q29RU4, Q5E9J6, P61955, Q1RMU2, A6H744, Q3T0I2, Q2KIJ6, Q10741, Q95M18, Q5E987, A4IFN7, P31800, A4FV12, Q95M12, P05689, Q3T0Z3, P61085, Q2KIK0, Q3ZCF7, Q32PG3, Q3ZCF3, Q27970, Q08DE9, Q1LZE1, Q9XT96, Q1RMX2, Q3T134, A6QLA4, A6QR55, Q3SX24, Q3SX23, A1L506, Q0VCP5, P07688, A2VDQ5, Q29RK4, P00730, Q0VD31, Q2HJE4, P23004, P23356, Q3SZC2, P33672, Q3SZU5, Q2TBU0, P00743, Q2KII4, Q29463, Q3MHN0, A6QNM7, Q32KL2, A1A4K3, Q32PA5, Q5E953, Q0P568, Q3ZCK9, Q5E9F9, Q3T168, Q0P5K3, Q1RMW1, A2VE14, P80227, A2VDP1, Q2HJH1, Q3ZCJ8, Q32LN1, Q2NKZ9, Q3SZ71, A2VE20, Q0VCU3, Q59HJ6, Q0V8B6, A2VEA3, Q3T0V2	1472	615	8785	1.21302249	1	0.203938567	19.64212218
GOTERM_BP_FAT	GO:0006733~oxidoreduction coenzyme metabolic process	11	0.513299113	0.0120682	Q3T131, A4FUF0, Q58CW1, Q3T186, Q2KIL4, Q5E956, Q0P5A2, A6QR05, Q2NL34, Q2HJD7, Q3ZCI4	1472	28	8785	2.344599185	1	0.203246844	19.68259017
GOTERM_BP_FAT	GO:0030705~cytoskeleton-dependent intracellular transport	7	0.32664489	0.0128885	Q2HJF8, Q08DH4, A7E2Z6, Q5E9M9, A6H7H2, A2VDK6, P23356	1472	13	8785	3.213576505	1	0.214381244	20.87799569
GOTERM_BP_FAT	GO:0009067~aspartate family amino acid biosynthetic process	6	0.279981335	0.0157118	Q2NL31, Q4JIJ3, Q0VD27, P33097, Q1LZA3, P12344	1472	10	8785	3.580842391	1	0.253817688	24.86521921
GOTERM_BP_FAT	GO:0006536~glutamate metabolic process	6	0.279981335	0.0157118	Q32S38, Q2T9Y6, P00366, P33097, P12344, Q3SZJ0	1472	10	8785	3.580842391	1	0.253817688	24.86521921
GOTERM_BP_FAT	GO:0042440~pigment metabolic process	10	0.466635558	0.016036	A5D7K0, Q56JW4, Q2HJ58, P56658, Q59A32, A5PK65, Q1LZ95, Q2KIN5, P22600, Q58DK5	1472	25	8785	2.387228261	1	0.25702041	25.31076017
GOTERM_BP_FAT	GO:0030100~regulation of endocytosis	10	0.466635558	0.016036	Q3T0G8, P45478, Q58DS9, P52193, Q95114, Q1RMR9, Q2TBH7, Q08E63, A5D7D1, P19035	1472	25	8785	2.387228261	1	0.25702041	25.31076017
GOTERM_BP_FAT	GO:0030150~protein import into mitochondrial matrix	4	0.186654223	0.0163968	Q3SZC1, Q32L81, Q0P5N5, Q0G846	1472	4	8785	5.968070652	1	0.260674738	25.80356631
GOTERM_BP_FAT	GO:0008631~induction of apoptosis by oxidative stress	4	0.186654223	0.0163968	P00435, Q2NL36, Q08DY9, Q0P5F4	1472	4	8785	5.968070652	1	0.260674738	25.80356631
GOTERM_BP_FAT	GO:0046129~purine ribonucleoside biosynthetic process	5	0.233317779	0.0178829	Q56JW4, Q05927, P56658, A6H7F4, Q3SZ18	1472	7	8785	4.262907609	1	0.279412535	27.80142694
GOTERM_BP_FAT	GO:0010257~NADH dehydrogenase complex assembly	5	0.233317779	0.0178829	Q2HJI2, Q02375, P42028, A4FUH5, Q5E9H5	1472	7	8785	4.262907609	1	0.279412535	27.80142694
GOTERM_BP_FAT	GO:0042455~ribonucleoside biosynthetic process	5	0.233317779	0.0178829	Q56JW4, Q05927, P56658, A6H7F4, Q3SZ18	1472	7	8785	4.262907609	1	0.279412535	27.80142694
GOTERM_BP_FAT	GO:0009163~nucleoside biosynthetic process	5	0.233317779	0.0178829	Q56JW4, Q05927, P56658, A6H7F4, Q3SZ18	1472	7	8785	4.262907609	1	0.279412535	27.80142694
GOTERM_BP_FAT	GO:0032981~mitochondrial respiratory chain complex I assembly	5	0.233317779	0.0178829	Q2HJI2, Q02375, P42028, A4FUH5, Q5E9H5	1472	7	8785	4.262907609	1	0.279412535	27.80142694
GOTERM_BP_FAT	GO:0042451~purine nucleoside biosynthetic process	5	0.233317779	0.0178829	Q56JW4, Q05927, P56658, A6H7F4, Q3SZ18	1472	7	8785	4.262907609	1	0.279412535	27.80142694
GOTERM_BP_FAT	GO:0043067~regulation of programmed cell death	65	3.033131125	0.018071	Q9XT96, Q95KV7, Q02399, Q5E9L4, P23727, Q5E9C2, Q2KI72, P48734, A0JNK3, A4FV00, Q3T0G8, A6QLB1, P80177, Q0Z7W6, P45478, Q2HJ92, Q3MHM6, Q0VC36, Q645M6, Q9TUM3, P43481, Q6EWQ7, Q0P594, Q9BGI3, Q9BGI1, Q0VC37, P61585, Q08DY9, Q3MQ24, Q2T9S4, Q3SX02, Q0VCJ2, Q0V882, P35705, P00442, Q3SZ15, Q0P5N4, Q3ZBT1, Q32LM0, Q1LZA3, Q3MHF7, Q29455, P38447, Q2NL36, A5D7R9, Q3ZCC3, P07994, Q0P5F4, Q95M18, Q5E984, P00435, P00432, Q3T169, Q27965, Q17QR2, Q5E947, A5D7E8, Q32S38, Q08DE0, A6QLT9, P56658, Q5EAC7, Q08DE5, O02703, Q0VCY8, Q2NL24	1472	298	8785	1.301760377	1	0.280514306	28.05063128
GOTERM_BP_FAT	GO:0009310~amine catabolic process	15	0.699953336	0.0186087	Q5EA40, P25285, P00366, Q3MHL4, A4IFQ7, O46504, P20821, P33097, P12344, Q0NXR6, P21839, A4FUD0, Q2T9V8, Q2HJ73, Q865R1	1472	47	8785	1.9047034	1	0.286179854	28.75858635
GOTERM_BP_FAT	GO:0010941~regulation of cell death	65	3.033131125	0.0193521	Q9XT96, Q95KV7, Q02399, Q5E9L4, P23727, Q5E9C2, Q2KI72, P48734, A0JNK3, A4FV00, Q3T0G8, A6QLB1, P80177, Q0Z7W6, P45478, Q2HJ92, Q3MHM6, Q0VC36, Q645M6, Q9TUM3, P43481, Q6EWQ7, Q0P594, Q9BGI3, Q9BGI1, Q0VC37, P61585, Q08DY9, Q3MQ24, Q2T9S4, Q3SX02, Q0VCJ2, Q0V882, P35705, P00442, Q3SZ15, Q0P5N4, Q3ZBT1, Q32LM0, Q1LZA3, Q3MHF7, Q29455, P38447, Q2NL36, A5D7R9, Q3ZCC3, P07994, Q0P5F4, Q95M18, Q5E984, P00435, P00432, Q3T169, Q27965, Q17QR2, Q5E947, A5D7E8, Q32S38, Q08DE0, A6QLT9, P56658, Q5EAC7, Q08DE5, O02703, Q0VCY8, Q2NL24	1472	299	8785	1.297406664	1	0.29438485	29.72651152
GOTERM_BP_FAT	GO:0009112~nucleobase metabolic process	7	0.32664489	0.0194125	Q56JW4, Q07536, Q2HJ58, P56658, Q59A32, P31754, Q3SZ18	1472	14	8785	2.984035326	1	0.293732429	29.804511
GOTERM_BP_FAT	GO:0042743~hydrogen peroxide metabolic process	7	0.32664489	0.0194125	Q9BGI3, Q3MHF7, P00435, P00442, P35705, P00432, Q5E946, Q5E947	1472	14	8785	2.984035326	1	0.293732429	29.804511

Category	Term	Count	%	PValue	Genes	List Total	Pop Hits	Pop Total	Fold Enrichment	Bonferroni	Benjamini	FDR
GOTERM_BP_FAT	GO:0043648~dicarboxylic acid metabolic process	7	0.32664489	0.0194125	Q3T145, Q32LG3, Q148D3, Q08DM3, Q9TUM3, P33097, P12344	1472	14	8785	2.984035326	1	0.293732429	29.804511
GOTERM_BP_FAT	GO:0000184~nuclear-transcribed mRNA catabolic process, nonsense-mediated decay	7	0.32664489	0.0194125	A5D7H5, A6QPH1, Q8MJJ7, Q2YDD2, Q3ZBV3, Q3T102, Q2NL22	1472	14	8785	2.984035326	1	0.293732429	29.804511
GOTERM_BP_FAT	GO:0008654~phospholipid biosynthetic process	14	0.653289781	0.0202538	A0JNC1, P10790, Q2KHY9, Q3MHZ7, P20456, Q95JH2, Q2NL29, Q3T103, Q3SZL5, Q2KJ28, Q32LM0, Q0VCY6, Q3SZL3, Q1LZA4	1472	43	8785	1.94309277	1	0.302952499	30.88380135
GOTERM_BP_FAT	GO:0007006~mitochondrial membrane organization	8	0.373308446	0.0210259	Q3T0G8, A6QPI6, Q0VCA3, Q1RMV0, O02703, Q76LV2, Q3SZB3, Q32LM0	1472	18	8785	2.652475845	1	0.311104336	31.86050961
GOTERM_BP_FAT	GO:0051262~protein tetramerization	8	0.373308446	0.0210259	Q3ZC55, A0JNI4, Q1RMV0, P00432, A6QR05, Q3SZ18, Q3ZBD3, P01096	1472	18	8785	2.652475845	1	0.311104336	31.86050961
GOTERM_BP_FAT	GO:0042981~regulation of apoptosis	64	2.986467569	0.0211443	Q9XT96, Q95KV7, Q02399, Q5E9L4, P23727, Q5E9C2, Q2KI72, P48734, A0JNK3, A4FV00, Q3T0G8, A6QLB1, P80177, Q0Z7W6, P45478, Q2HJ92, Q3MHM6, Q0VC36, Q645M6, Q9TUM3, Q6EWQ7, Q0P594, Q9BGI3, Q9BGI1, Q0VC37, P61585, Q08DY9, Q3MQ24, Q2T9S4, Q3SX02, Q0VCJ2, Q0V882, P35705, P00442, Q3SZ15, Q0P5N4, Q3ZBT1, Q32LM0, Q1LZA3, Q3MHF7, Q29455, P38447, Q2NL36, A5D7R9, Q3ZCC3, P07994, Q0P5F4, Q95M18, Q5E984, P00435, P00432, Q3T169, Q27965, Q17QR2, Q5E947, A5D7E8, Q32S38, Q08DE0, A6QLT9, P56658, Q5EAC7, Q08DE5, O02703, Q0VCY8, Q2NL24	1472	295	8785	1.29476787	1	0.311099185	32.00918671
GOTERM_BP_FAT	GO:0012502~induction of programmed cell death	21	0.979934671	0.0226791	Q95KV7, P00435, Q2HJ92, Q3T169, Q5E947, Q2KI72, Q0VC36, Q0P5N4, A4FV00, A0JNK3, Q6EWQ7, Q32LM0, Q0VC37, Q08DE0, Q08DE5, O02703, Q2NL36, Q08DY9, P07994, A6QLB1, Q0P5F4	1472	76	8785	1.649072154	1	0.328176756	33.90812587
GOTERM_BP_FAT	GO:0006917~induction of apoptosis	21	0.979934671	0.0226791	Q95KV7, P00435, Q2HJ92, Q3T169, Q5E947, Q2KI72, Q0VC36, Q0P5N4, A4FV00, A0JNK3, Q6EWQ7, Q32LM0, Q0VC37, Q08DE0, Q08DE5, O02703, Q2NL36, Q08DY9, P07994, A6QLB1, Q0P5F4	1472	76	8785	1.649072154	1	0.328176756	33.90812587
GOTERM_BP_FAT	GO:0051260~protein homooligomerization	12	0.559962669	0.0236937	A1L567, P80177, Q3ZC55, A2VDN5, A0JNI4, Q59HJ6, Q3SZ18, Q58D72, O02703, P79132, Q3ZBD3, P01096	1472	35	8785	2.046195652	1	0.338624756	35.13584424
GOTERM_BP_FAT	GO:0006260~DNA replication	23	1.073261783	0.0239092	Q58DC8, Q2KIZ8, Q05B83, Q29RS9, A6QLA6, Q3ZBH9, Q1KZG4, Q3MHL3, Q32PJ3, Q3ZBW4, Q2T9P4, Q5BIP4, Q0VC30, P28339, Q58DH8, P49004, Q2TBV1, Q0V8B7, A4IFC8, Q32PI3, Q0VCV0, Q1RMH9, Q3SWX8, Q3T165	1472	86	8785	1.596111919	1	0.339599813	35.39383369
GOTERM_BP_FAT	GO:0051085~chaperone mediated protein folding requiring cofactor	6	0.279981335	0.0248936	Q3MI00, A1A4M8, P19120, A5PK75, A5PJN2, Q0IIM3	1472	11	8785	3.255311265	1	0.349365889	36.55997997
GOTERM_BP_FAT	GO:0051028~mRNA transport	10	0.466635558	0.0269091	A5D7H5, Q29RH4, P09867, A7YY75, Q3ZBV3, Q3ZBV2, A5PJZ5, Q2NL22, Q2TBP1, Q6EWQ7	1472	27	8785	2.210396538	1	0.370303484	38.88594226
GOTERM_BP_FAT	GO:0000075~cell cycle checkpoint	10	0.466635558	0.0269091	A6QQW8, Q17QG8, A1A4K3, A5D7V1, A6QM04, Q0P5L6, P48734, Q2NL24, Q08E57, Q862I1	1472	27	8785	2.210396538	1	0.370303484	38.88594226
GOTERM_BP_FAT	GO:0007265~Ras protein signal transduction	10	0.466635558	0.0269091	P19803, Q0IIM6, A5PKL2, Q5E9C0, A6H7G2, P19035, A2VDK6, Q2KI13, A7YY55, Q3SZA6	1472	27	8785	2.210396538	1	0.370303484	38.88594226
GOTERM_BP_FAT	GO:0031400~negative regulation of protein modification process	10	0.466635558	0.0269091	Q9XT96, Q3T0G8, Q32S38, Q02399, Q3T178, O02703, P18203, P79132, P62261, Q3MQ24	1472	27	8785	2.210396538	1	0.370303484	38.88594226
GOTERM_BP_FAT	GO:0000737~DNA catabolic process, endonucleolytic	7	0.32664489	0.0278658	P45478, Q0VC37, P00442, P38447, O02703, Q08DY9, Q58CZ0	1472	15	8785	2.785099638	1	0.379085708	39.96153177
GOTERM_BP_FAT	GO:0008629~induction of apoptosis by intracellular signals	7	0.32664489	0.0278658	P00435, Q0VC36, O02703, Q2NL36, Q08DY9, A0JNK3, Q0P5F4	1472	15	8785	2.785099638	1	0.379085708	39.96153177
GOTERM_BP_FAT	GO:0007018~microtubule-based movement	19	0.886607559	0.0280126	Q2NL05, Q3MHM5, A7E2Z6, Q5E9M9, Q2T9S0, Q2KJD0, Q3ZCJ7, P81947, Q2HJF8, Q08DH4, A5D7N6, Q2HJ86, A6QNT3, A6H7H2, A6QPE8, P23356, Q3ZBD2, A7MBA1, Q29RT6	1472	68	8785	1.667549153	1	0.379041333	40.12499079
GOTERM_BP_FAT	GO:0017038~protein import	16	0.746616892	0.0284074	A1L567, Q1RMV0, A6QLL2, Q32L81, Q1LZB5, Q0P5N5, A7YWD2, Q2TBS1, A1A4I8, Q3SZC1, C1JZ67, Q2KII7, Q3SYV6, Q0G846, Q32KP9, A6QPK4	1472	54	8785	1.76831723	1	0.381635568	40.56250515
GOTERM_BP_FAT	GO:0000097~sulfur amino acid biosynthetic process	5	0.233317779	0.0310795	Q2NL31, Q58DW2, Q4JIJ3, A7E3T7, Q0VD27	1472	8	8785	3.730044158	1	0.407713604	43.44521128
GOTERM_BP_FAT	GO:0043574~peroxisomal transport	5	0.233317779	0.0310795	A1L567, Q2KII7, Q1RMV0, Q3SZD1, A6QPK4	1472	8	8785	3.730044158	1	0.407713604	43.44521128
GOTERM_BP_FAT	GO:0042744~hydrogen peroxide catabolic process	5	0.233317779	0.0310795	Q9BGI3, Q3MHF7, P00435, P35705, P00432, Q5E947	1472	8	8785	3.730044158	1	0.407713604	43.44521128
GOTERM_BP_FAT	GO:0070301~cellular response to hydrogen peroxide	5	0.233317779	0.0310795	Q9BGI3, Q3MHF7, P00435, P35705, P00432, Q5E947	1472	8	8785	3.730044158	1	0.407713604	43.44521128
GOTERM_BP_FAT	GO:0006625~protein targeting to peroxisome	5	0.233317779	0.0310795	A1L567, Q2KII7, Q1RMV0, Q3SZD1, A6QPK4	1472	8	8785	3.730044158	1	0.407713604	43.44521128
GOTERM_BP_FAT	GO:0006098~pentose-phosphate shunt	5	0.233317779	0.0310795	A4FUF0, Q3T186, Q5E956, Q2HJD7, Q3ZCI4	1472	8	8785	3.730044158	1	0.407713604	43.44521128
GOTERM_BP_FAT	GO:0006887~exocytosis	16	0.746616892	0.0332149	Q17QI5, Q17QF8, A0JN38, Q5E971, P83939, A5D7A2, Q3T0D2, Q0V8C2, P61763, A6QLD1, Q3T000, Q27991, A4FV54, P11023, Q0P5F3, A6QLB1	1472	55	8785	1.736166008	1	0.427277742	45.65353959
GOTERM_BP_FAT	GO:0032535~regulation of cellular component size	21	0.979934671	0.0336212	Q32LP2, Q95KV7, Q5E997, P45478, A4FUA8, Q0VC36, Q5E963, Q3T035, Q05B87, Q1LZB2, Q3MHR7, Q08DE0, Q9GK68, Q5E9D5, Q08DE5, Q58CQ2, Q3SYX9, Q1JP79, P61157, Q10741, P62998	1472	79	8785	1.586449161	1	0.429516841	46.06431991
GOTERM_BP_FAT	GO:0051258~protein polymerization	10	0.466635558	0.0339391	Q3MHM5, Q2T9S0, Q2HJ86, A5D9D4, A6QNT3, Q32KM1, Q2KJD0, Q3ZCJ7, Q148J6, P81947	1472	28	8785	2.131453804	1	0.430876268	46.38373703
GOTERM_BP_FAT	GO:0010608~posttranscriptional regulation of gene expression	22	1.026598227	0.0341089	A6QPH1, P68102, Q863B3, Q9N0T5, Q2HJ92, Q2TA06, A5D9H5, Q3ZBV3, A2VDR8, Q2T9S7, Q5E988, Q6EWQ7, A5D7H5, A3KN48, Q0VCU1, A7MB16, Q3ZCE2, Q95L46, Q2NL22, Q2TBP1, Q647I9, Q3T0V3	1472	84	8785	1.563066123	1	0.430803358	46.55359758
GOTERM_BP_FAT	GO:0016567~protein ubiquitination	14	0.653289781	0.0347915	Q3MHX6, Q0VCL3, Q1RMX2, P63048, A1A4K3, Q32PA5, Q0VCP5, A7MBJ5, A2VDP1, Q32LN1, P62992, Q08E63, Q32PF3, Q3ZCC3	1472	46	8785	1.816369329	1	0.43559473	47.23136642
GOTERM_BP_FAT	GO:0006465~signal peptide processing	4	0.186654223	0.0359015	Q3T134, P67810, Q2KI36, Q3SZU5	1472	5	8785	4.774456522	1	0.444272634	48.3162064
GOTERM_BP_FAT	GO:0007023~post-chaperonin tubulin folding pathway	4	0.186654223	0.0359015	Q3SZE9, Q32KS0, P48427, Q5E951	1472	5	8785	4.774456522	1	0.444272634	48.3162064
GOTERM_BP_FAT	GO:0006534~cysteine metabolic process	4	0.186654223	0.0359015	A5PKG4, Q58DW2, Q32S38, Q3SZU4	1472	5	8785	4.774456522	1	0.444272634	48.3162064
GOTERM_BP_FAT	GO:0006561~proline biosynthetic process	4	0.186654223	0.0359015	Q58DT4, Q58D08, Q2KJH7, A7YWE4	1472	5	8785	4.774456522	1	0.444272634	48.3162064
GOTERM_BP_FAT	GO:0046165~alcohol biosynthetic process	9	0.419972002	0.0361288	Q5EA65, Q5E956, Q3SZB7, Q3SZJ9, Q58CR4, Q2NL29, Q29RK2, P33097, Q3ZCI4	1472	24	8785	2.238026495	1	0.444666518	48.53572504

Category	Term	Count	%	PValue	Genes	List Total	Pop Hits	Pop Total	Fold Enrichment	Bonferroni	Benjamini	FDR
GOTERM_BP_FAT	GO:0051098~regulation of binding	15	0.699953336	0.0368908	A1L567, P62157, Q24JZ4, P35705, P00432, Q3T169, P23196, Q0P5K3, Q3MHX1, Q9BGI3, Q3MHF7, Q08DE0, Q3T0V7, O02703, Q32L01, Q3ZBD3	1472	51	8785	1.755314898	1	0.449933093	49.26528678
GOTERM_BP_FAT	GO:0006195~purine nucleotide catabolic process	6	0.279981335	0.0369113	Q05927, P56658, Q59HJ6, A7MBI8, P00829, Q2HJ33	1472	12	8785	2.984035326	1	0.448421512	49.28478108
GOTERM_BP_FAT	GO:0006749~glutathione metabolic process	6	0.279981335	0.0369113	Q9XSG3, Q32S38, P00435, Q2T9Y6, P00442, Q5EAC2	1472	12	8785	2.984035326	1	0.448421512	49.28478108
GOTERM_BP_FAT	GO:0006401~RNA catabolic process	8	0.373308446	0.0378104	A5D7H5, Q58DH8, A6QPH1, Q8MJJ7, Q2YDD2, Q3ZBV3, Q3T102, Q2NL22	1472	20	8785	2.387228261	1	0.454809194	50.13269966
GOTERM_BP_FAT	GO:0016125~sterol metabolic process	14	0.653289781	0.040979	P07514, Q3T0N9, Q8WMY2, P00432, Q8WMV1, Q1LZ95, A4FUD2, P84466, Q5E9T8, Q58DH7, A6QR14, P19035, Q3ZBE9, Q5E9J5	1472	47	8785	1.777723173	1	0.480648147	53.01544355
GOTERM_BP_FAT	GO:0006720~isoprenoid metabolic process	10	0.466635558	0.0421379	P56966, Q5E9T8, Q8WMY2, Q5EA65, Q5PXY7, A6QR05, Q58CR4, Q1LZ95, P02694, Q3ZC42	1472	29	8785	2.057955397	1	0.488641411	54.02987234
GOTERM_BP_FAT	GO:0008203~cholesterol metabolic process	13	0.606626225	0.045522	P07514, Q3T0N9, Q8WMY2, Q8WMV1, P00432, Q1LZ95, A4FUD2, P84466, Q5E9T8, A6QR14, P19035, Q3ZBE9, Q5E9J5	1472	43	8785	1.80430043	1	0.514295593	56.87510981
GOTERM_BP_FAT	GO:0008344~adult locomotory behavior	9	0.419972002	0.0455842	Q3T0G8, Q0V8R6, P06623, P45478, Q32KS0, Q5E946, P23356, A0JNK3, Q05B87	1472	25	8785	2.148505435	1	0.513019741	56.92584028
GOTERM_BP_FAT	GO:0033365~protein localization in organelle	17	0.793280448	0.0457767	Q3MHE8, A6QLL2, Q32L81, Q1LZB5, Q0P5N5, A2VDR8, A7YWD2, Q3ZC32, Q2TBS1, A1A4I8, Q3SZC1, C1JZ67, Q3SYV6, Q0G846, Q32KP9, Q5EA68, Q2T9U1	1472	62	8785	1.636406469	1	0.512774783	57.08237882
GOTERM_BP_FAT	GO:0051017~actin filament bundle formation	5	0.233317779	0.0486648	P61585, A5D7D1, Q0VCF1, P31976, Q3B7N2	1472	9	8785	3.315594807	1	0.533144392	59.36780821
GOTERM_BP_FAT	GO:0009154~purine ribonucleotide catabolic process	5	0.233317779	0.0486648	Q05927, Q59HJ6, A7MBI8, P00829, Q2HJ33	1472	9	8785	3.315594807	1	0.533144392	59.36780821
GOTERM_BP_FAT	GO:0009261~ribonucleotide catabolic process	5	0.233317779	0.0486648	Q05927, Q59HJ6, A7MBI8, P00829, Q2HJ33	1472	9	8785	3.315594807	1	0.533144392	59.36780821
GOTERM_BP_FAT	GO:0046112~nucleobase biosynthetic process	5	0.233317779	0.0486648	Q56JW4, Q2HJ58, P56658, Q59A32, P31754	1472	9	8785	3.315594807	1	0.533144392	59.36780821
GOTERM_BP_FAT	GO:0006544~glycine metabolic process	5	0.233317779	0.0486648	P25285, O46504, P20821, P00376, Q3SZ20	1472	9	8785	3.315594807	1	0.533144392	59.36780821
GOTERM_BP_FAT	GO:0051289~protein homotetramerization	5	0.233317779	0.0486648	Q3ZC55, A0JNI4, Q3SZ18, Q3ZBD3, P01096	1472	9	8785	3.315594807	1	0.533144392	59.36780821

Table S3. List of significant differentially expressed proteins between different groups.

				MII G vs GVO		MII G vs MII B	
#	Accession number	Gene symbol	Discription	Ratio	p-value	Ratio	p-value
1	F1MGJ7	AUH	Uncharacterized protein	11.34	9.00E-15	1.35	9.00E-15
2	P13909	SERPINE1	Plasminogen activator inhibitor 1	4.63	2.70E-06	1.00	2.70E-06
3	F1MZD1	WEE2	Uncharacterized protein	4.51	1.85E-11	0.30	9.97E-02
4	Q32PG3	WDR48	WD repeat-containing protein 48	4.26	4.91E-06	3.81	4.91E-06
5	Q3SZY3	PTTG1	Securin	4.21	1.22E-07	1.05	1.22E-07
6	Q0VCQ5	KLHL7	Kelch-like 7 (Drosophila)	3.19	1.79E-03	0.99	3.38E-01
7	E1B7X9	SMARCAD1	SWI/SNF-related matrix-associated actin-dependent regulator of chromatin subfamily A containing DEAD/H box 1	3.72	2.59E-04	1.93	2.59E-04
8	A9Q1J6	RNF18	Ring finger protein 18	3.58	8.35E-05	1.22	8.35E-05
9	Q24K09	DNMT1	DNA (cytosine-5)-methyltransferase 1	3.56	2.69E-05	0.57	3.27E-01
10	Q9TTJ5	RGN	Regucalcin	10.69	2.76E-08	0.18	3.42E-02
11	E1BIJ1	DEPDC7	Uncharacterized protein	3.50	3.63E-07	0.40	1.54E-01
12	Q2KJA6	INTS9	Integrator complex subunit 9	3.39	6.21E-06	1.53	6.21E-06
13	A2VDY4	C8H4orf27	UPF0609 protein C4orf27 homolog	3.27	8.17E-04	1.11	8.17E-04
14	F1MLM3		Uncharacterized protein (Fragment)	3.72	4.28E-04	1.16	2.80E-01
15	A7E320	UHRF1	E3 ubiquitin-protein ligase UHRF1	3.11	1.78E-06	0.55	2.93E-01
16	F1MMD5	XRCC6	Uncharacterized protein	3.09	6.40E-05	0.63	3.75E-01
17	Q32PA5	UBE2C	Ubiquitin-conjugating enzyme E2 C	3.05	3.72E-04	1.58	3.72E-04
18	E1BDG7	USP28	Ubiquitin carboxyl-terminal hydrolase	3.04	8.14E-04	1.77	8.14E-04
19	F1MRT3	PRC1	Uncharacterized protein	2.96	7.84E-06	0.74	3.91E-01
20	Q0VCJ5	MOBKL1B	MOB1, Mps One Binder kinase activator-like 1B (Yeast)	2.94	1.24E-03	1.18	1.24E-03
21	Q56JV1	RPS26	40S ribosomal protein S26	2.92	1.01E-02	1.27	3.02E-01
22	Q32LB0	WDR70	WD repeat-containing protein 70	2.81	2.45E-03	1.06	2.45E-03
23	Q2KIP8	PTPLB	Very-long-chain (3R)-3-hydroxyacyl-[acyl-carrier protein] dehydratase 2	2.78	1.17E-02	4.03	3.31E-03
24	F1MW01	ESPL1	Uncharacterized protein	2.77	1.66E-04	0.80	1.66E-04
25	E1BP79	WDR47	Uncharacterized protein	2.75	2.12E-04	0.61	3.79E-01

				MII G vs GVO		MII G vs MII B	
#	Accession number	Gene symbol	Discription	Ratio	p-value	Ratio	p-value
26	Q32LN1	UBE2H	Ubiquitin-conjugating enzyme E2 H	2.72	1.46E-04	1.39	1.46E-04
27	F1N7S0	TULP3	Uncharacterized protein	2.68	2.46E-03	1.20	2.46E-03
28	Q1LZ76	RTN1	Reticulon	2.67	1.55E-02	3.39	4.70E-03
29	F6RJG0	HMGCS1	Uncharacterized protein (Fragment)	2.66	4.79E-05	0.26	5.86E-02
30	Q2KJE0	TAX1BP1	Tax1-binding protein 1 homolog	2.95	8.31E-03	0.92	5.00E-01
31	E1BN70	INTS3	Uncharacterized protein	2.62	1.27E-03	1.23	1.27E-03
32	P61955	SUMO2	Small ubiquitin-related modifier 2	2.62	2.39E-04	0.31	9.79E-02
33	E1BIU3		Uncharacterized protein	2.61	3.94E-04	1.65	3.94E-04
34	G3MXK7		Uncharacterized protein (Fragment)	0.27	3.49E-03	0.23	4.20E-02
35	F1N155	CLPX	Uncharacterized protein (Fragment)	2.79	6.20E-03	1.04	3.10E-01
36	P48644	ALDH1A1	Retinal dehydrogenase 1	2.58	1.17E-04	2.06	1.17E-04
37	F1MBE7	ACCSL	Uncharacterized protein (Fragment)	2.54	1.29E-05	0.78	3.94E-01
38	F1MYQ1	TXNDC9	Thioredoxin domain-containing protein 9	2.51	4.38E-04	0.59	3.54E-01
39	Q58D05	TRAFD1	TRAF-type zinc finger domain-containing protein 1	2.45	9.38E-04	0.68	9.38E-04
40	Q08DS5	NMD3	60S ribosomal export protein NMD3	2.42	1.58E-03	1.62	1.58E-03
41	Q29RT6	KIF20A	Kinesin-like protein KIF20A	2.38	4.32E-03	2.28	4.32E-03
42	F1MW73	MGC148692	Uncharacterized protein	2.38	4.18E-03	0.43	2.24E-01
43	Q08DX5	CDK7	Cyclin-dependent kinase 7	2.35	6.77E-03	1.58	6.77E-03
44	F1N779	CLPTM1	Cleft lip and palate transmembrane protein 1 homolog	2.34	2.04E-02	2.05	3.63E-02
45	Q5E9T5	TMEM35	Transmembrane protein 35	2.34	3.20E-02	22.61	1.60E-08
46	A6QPE8	KIF2C	KIF2C protein	2.33	5.46E-03	0.71	3.83E-01
47	F1N6W2	PPIL4	Uncharacterized protein	2.29	8.19E-03	0.05	1.36E-04
48	E1BFB5	KIAA0391	Uncharacterized protein	2.29	2.97E-03	1.76	2.97E-03
49	Q02367	NDUFB6	NADH dehydrogenase [ubiquinone] 1 beta subcomplex subunit 6	2.28	2.28E-02	2.70	2.25E-02
50	F2Z4J6	GTF2B	Transcription initiation factor IIB	2.28	1.41E-03	1.28	1.41E-03
51	F1N678	AKR1B10	Uncharacterized protein	2.24	1.76E-04	2.99	1.76E-04
52	F1MZT3	MPHOSPH9	Uncharacterized protein	2.23	6.66E-03	0.49	2.44E-01
53	Q32PI1	VRK1	Serine/threonine-protein kinase VRK1	2.22	3.33E-03	0.34	1.35E-01
54	A9Q1J5	MELK	Maternal embryonic leucine zipper kinase	2.22	9.98E-03	1.22	9.98E-03

				MII G vs GVO		MII G vs MII B	
#	Accession number	Gene symbol	Discription	Ratio	p-value	Ratio	p-value
55	E1BF29	KIF11	Uncharacterized protein	3.01	6.88E-03	1.16	2.73E-01
56	Q3SZ73	ABHD11	Alpha/beta hydrolase domain-containing protein 11	2.19	4.16E-03	2.15	4.16E-03
57	Q5E9J6	RNF34	E3 ubiquitin-protein ligase RNF34	2.16	2.42E-03	0.92	2.42E-03
58	Q08DL0	SLC3A2	SLC3A2 protein	2.15	4.86E-02	1.49	1.80E-01
59	E1BQ11	CTNND1	Uncharacterized protein	2.11	1.22E-03	0.57	2.93E-01
60	Q5E9J1	HNRNPF	Heterogeneous nuclear ribonucleoprotein F	2.09	1.36E-03	0.95	4.09E-01
61	A6QLG6	FXR1	FXR1 protein	2.09	3.83E-02	1.41	2.27E-01
62	P62958	HINT1	Histidine triad nucleotide-binding protein 1	2.08	1.78E-03	2.01	1.78E-03
63	G3MYS0	LOC508073	Uncharacterized protein	2.07	7.43E-04	0.49	2.20E-01
64	F1MTR1	IQGAP2	Uncharacterized protein	2.07	2.50E-03	0.59	2.98E-01
65	P21839	BCKDHB	2-oxoisovalerate dehydrogenase subunit beta, mitochondrial	2.07	1.67E-02	2.01	1.67E-02
66	Q3MHR3	DYNLL2	Dynein light chain 2, cytoplasmic	2.06	7.03E-04	0.75	3.73E-01
67	Q08DR9	SPDL1	Protein Spindly	2.06	4.96E-03	1.14	4.96E-03
68	F1MRP8	PLEKHG1	Uncharacterized protein	2.06	4.26E-03	1.70	4.26E-03
69	Q2YDJ0	NUSAP1	Nucleolar and spindle-associated protein 1	2.04	5.19E-03	0.43	2.01E-01
70	Q32LN7	WDR61	WD repeat-containing protein 61	2.04	8.08E-03	0.81	4.43E-01
71	Q05B58	CCDC53	WASH complex subunit CCDC53	2.04	1.38E-02	0.85	4.63E-01
72	F1MV39		Uncharacterized protein (Fragment)	2.02	1.95E-03	0.94	4.88E-01
73	Q3ZBW4	PCNA	Proliferating cell nuclear antigen	2.01	1.21E-03	0.41	1.64E-01
74	F1N7F8	PIGU	Uncharacterized protein	1.98	4.94E-02	2.12	3.46E-02
75	F1MNK8	KIF26B	Uncharacterized protein (Fragment)	1.92	7.94E-03	2.09	7.94E-03
76	F1MUU2	STK35	Uncharacterized protein	3.80	4.88E-04	0.79	4.38E-01
77	F1MSQ9	TMPO	Uncharacterized protein	2.59	2.18E-02	1.14	3.17E-01
78	Q32KL5	EMC8	ER membrane protein complex subunit 8	1.85	6.84E-02	1.99	4.70E-02
79	F1MSE7	SUSD2	Uncharacterized protein	1.81	8.02E-02	2.06	4.44E-02
80	P80724	BASP1	Brain acid soluble protein 1	1.76	1.20E-01	2.68	1.77E-02
81	P23934	NDUFS6	NADH dehydrogenase [ubiquinone] iron-sulfur protein 6, mitochondrial	1.76	1.50E-01	2.65	2.70E-02
82	P21327	INPP1	Inositol polyphosphate 1-phosphatase	0.34	3.27E-02	0.18	3.54E-02
83	A7MB57	YES1	Uncharacterized protein	2.63	1.39E-02	1.07	2.83E-01

				MII G vs GVO		MII G vs MII B	
#	Accession number	Gene symbol	Discription	Ratio	p-value	Ratio	p-value
84	A6QLV6	KCTD10	KCTD10 protein	1.72	3.08E-02	2.27	3.08E-02
85	Q3SZU4	CDO1	Cysteine dioxygenase type 1	0.51	6.66E-02	0.24	4.52E-02
86	Q2NKY7	SEPT2	Septin-2	0.59	1.51E-01	2.19	3.56E-02
87	Q02827	NDUFC2	NADH dehydrogenase [ubiquinone] 1 subunit C2	1.65	1.46E-01	2.13	4.40E-02
88	F1N0F7	EIF5	Uncharacterized protein	0.43	4.41E-02	0.28	8.70E-02
89	Q3ZBD3	PCBD1	Pterin-4-alpha-carbinolamine dehydratase	0.40	5.29E-02	0.19	4.25E-02
90	P10790	FABP3	Fatty acid-binding protein, heart	0.34	4.86E-02	0.31	1.18E-01
91	Q58DC0	CPPED1	Calcineurin-like phosphoesterase domain-containing protein 1	0.53	7.64E-02	0.22	3.87E-02
92	Q08DQ1	CAMK1	Calcium/calmodulin-dependent protein kinase I	0.44	6.04E-02	2.24	4.54E-02
93	Q32KL9	BCAP29	B-cell receptor-associated protein 29	1.85	6.44E-02	3.39	4.71E-03
94	P55052	FABP5	Fatty acid-binding protein, epidermal	0.36	3.83E-02	0.38	1.37E-01
95	F1MC72	UBE2L3	Ubiquitin-conjugating enzyme E2 L3 (Fragment)	0.45	3.89E-02	0.37	1.27E-01
96	Q58DK5	ALAD	Delta-aminolevulinic acid dehydratase	0.32	1.67E-02	0.19	3.54E-02
97	P56966	GGPS1	Geranylgeranyl pyrophosphate synthase	0.42	4.46E-02	0.27	6.52E-02
98	Q32PA4	PHPT1	14 kDa phosphohistidine phosphatase	0.39	4.05E-02	0.28	8.51E-02
99	A5PK65	DDT	D-dopachrome decarboxylase	0.30	2.05E-02	0.19	3.83E-02
100	E1B7T3	NUDT15	Uncharacterized protein	0.22	2.73E-03	0.18	1.72E-02
101	Q288C4	NLRP9	NACHT, LRR and PYD domains-containing protein 9	1.35	2.90E-01	3.85	4.13E-03
102	P11023	RAB3A	Ras-related protein Rab-3A	2.11	3.46E-02	2.93	7.61E-03
103	F1MNP4	RPS6KB1	Ribosomal protein S6 kinase	0.41	3.78E-02	0.23	4.48E-02
104	P56658	ADA	Adenosine deaminase	0.24	3.62E-03	0.26	7.50E-02
105	Q3ZC07	ACTC1	Actin, alpha cardiac muscle 1	0.76	2.65E-01	33.61	4.75E-14
106	P80177	MIF	Macrophage migration inhibitory factor	0.39	4.76E-02	2.23	5.64E-02
107	Q32KP9	NUTF2	Nuclear transport factor 2	0.35	3.47E-02	0.22	4.64E-02
108	Q32L10	TMEM205	Transmembrane protein 205	1.88	7.51E-02	2.84	1.55E-02
109	Q0IIB1	PHYHD1	Phytanoyl-CoA dioxygenase domain-containing protein 1	0.43	4.61E-02	0.27	6.00E-02
110	Q2T9S0	TUBB3	Tubulin beta-3 chain	0.93	3.35E-01	2.48	2.00E-02
111	Q0P5D9	ALG8	Probable dolichyl pyrophosphate Glc1Man9GlcNAc2 alpha-1,3-glucosyltransferase	2.07	3.69E-02	2.49	1.71E-02

				MII G vs GVO		MII G vs MII B	
#	Accession number	Gene symbol	Discription	Ratio	p-value	Ratio	p-value
112	Q02380	NDUFB5	NADH dehydrogenase [ubiquinone] 1 beta subcomplex subunit 5, mitochondrial	1.75	1.03E-01	7.60	2.65E-05
113	G3X705	MRPS6	Uncharacterized protein	1.22	4.50E-01	2.98	1.85E-02
114	E1BE86	CCS	Uncharacterized protein	0.42	3.79E-02	0.23	5.13E-02
115	A2VDW4	TMED3	Transmembrane emp24 protein transport domain containing 3	2.57	1.83E-02	2.04	8.64E-02
116	F1MJW8	CALB2	Calretinin	0.43	4.77E-02	0.53	2.72E-01
117	Q6B4J2	VKORC1	Vitamin K epoxide reductase complex subunit 1	2.21	5.21E-02	2.35	4.68E-02
118	E1BF18	RAB3C	Ras-related protein Rab-3C	1.67	1.78E-01	3.24	7.69E-03
119	A5D977	RALB	RALB protein	1.72	1.31E-01	3.08	1.50E-02
120	Q0II26	AIMP2	Aminoacyl tRNA synthase complex-interacting multifunctional protein 2	1.30	3.65E-01	2.24	4.57E-02
121	Q3SZ18	HPRT1	Hypoxanthine-guanine phosphoribosyltransferase	0.37	2.53E-02	0.28	8.78E-02
122	Q95142	PDE6D	Retinal rod rhodopsin-sensitive cGMP 3,5-cyclic phosphodiesterase subunit delta	0.27	7.64E-03	0.14	1.26E-02
123	Q5E9K0	PSMB2	Proteasome subunit beta type-2	1.17	4.86E-01	6.66	3.41E-05
124	Q148J4	RAB2A	RAB2A, member RAS oncogene family	1.72	1.67E-01	2.40	2.68E-02
125	H9GW43	RNH1	Uncharacterized protein	0.41	3.98E-02	0.22	4.02E-02
126	P52505	NDUFAB1	Acyl carrier protein, mitochondrial	1.15	4.84E-01	4.04	4.13E-03
127	Q4F894	CLCN4	Chloride channel 4	2.36	2.11E-02	1.69	1.01E-01
128	F1MTN1	ITGB3	Integrin beta	2.61	1.81E-02	1.37	1.83E-01
129	A0JNQ6	OOEP	Oocyte-expressed protein homolog	1.27	3.69E-01	2.35	4.29E-02
130	P61085	UBE2K	Ubiquitin-conjugating enzyme E2 K	0.52	6.59E-02	0.24	4.95E-02
131	Q32KU6	TSPAN6	Tetraspanin-6	2.97	7.55E-03	3.59	3.09E-03
132	Q0VCI6	PIH1D1	PIH1 domain-containing protein 1	0.47	6.04E-02	0.23	4.86E-02
133	Q3MHJ8	STMN2	Stathmin	0.43	4.88E-02	0.32	9.68E-02
134	Q3T025	RPL17	60S ribosomal protein L17	2.09	3.50E-02	2.35	3.79E-02
135	F1ML72	RPL34	Uncharacterized protein (Fragment)	1.79	8.52E-02	2.16	4.09E-02
136	Q58DM8	ECHS1	Enoyl-CoA hydratase, mitochondrial	1.36	3.23E-01	3.28	7.96E-03
137	Q29463		Anionic trypsin	0.24	3.05E-03	0.23	4.80E-02
138	F1MBM4	ARL6IP1	Uncharacterized protein	2.05	4.85E-02	1.95	9.66E-02

				MII G vs GVO		MII G vs MII B	
**#**	**Accession number**	**Gene symbol**	**Discription**	**Ratio**	**p-value**	**Ratio**	**p-value**
139	Q2TBQ5	RPL7A	60S ribosomal protein L7a	2.57	1.73E-02	1.81	1.04E-01
140	E1BNJ0	TMEM33	Uncharacterized protein	1.90	1.05E-01	2.60	3.14E-02
141	Q3ZBF9	PDXP	Pyridoxal phosphate phosphatase	0.35	3.59E-02	0.17	3.32E-02
142	A6QLS5	MGC143209	MGC143209 protein	2.00	4.66E-02	1.96	7.76E-02
143	Q58DA0	PSMD4	26S proteasome non-ATPase regulatory subunit 4	1.06	4.37E-01	2.68	2.71E-02
144	G3X6Y2	CXorf38	Uncharacterized protein	0.42	3.67E-02	0.24	3.94E-02
145	Q148J1	TSPAN7	Tetraspanin 7	1.86	1.13E-01	11.68	2.81E-06
146	G3MZV0		Uncharacterized protein	1.05	4.69E-01	2.44	3.30E-02
147	Q1LZB2	NCK1	NCK adaptor protein 1	0.41	4.41E-02	0.42	2.15E-01
148	F1MWB9	GNL1	Uncharacterized protein	0.47	4.71E-02	0.19	2.79E-02
149	Q3SZI6	RPN2	Dolichyl-diphosphooligosaccharide--protein glycosyltransferase subunit 2	2.10	4.84E-02	1.86	1.17E-01
150	P82916	MRPS17	28S ribosomal protein S17, mitochondrial	1.71	9.34E-02	2.14	3.75E-02
151	Q17R06	RAB21	Ras-related protein Rab-21	1.50	2.33E-01	2.31	4.49E-02
152	Q2KHW4	SLC25A15	Solute carrier family 25 (Mitochondrial carrier; ornithine transporter) member 15	1.98	6.43E-02	2.97	1.74E-02
153	Q85BD6	ND5	NADH-ubiquinone oxidoreductase chain 5	2.43	8.00E-03	2.41	2.63E-02
154	Q05204	LAMP1	Lysosome-associated membrane glycoprotein 1	1.50	2.52E-01	2.56	3.36E-02
155	Q2KHX4	SEC61A2	Protein transport protein Sec61 subunit alpha isoform 2	2.23	1.97E-02	2.50	2.69E-02
156	F1MYN2	CGN	Uncharacterized protein	2.28	2.19E-02	1.81	8.32E-02
157	Q58DH7	SIGMAR1	Sigma non-opioid intracellular receptor 1	1.71	1.13E-01	3.15	5.71E-03
158	P12234-2		Isoform B of Phosphate carrier protein, mitochondrial	1.76	1.41E-01	2.21	4.31E-02
159	F1MX01	PNLDC1	Uncharacterized protein	1.71	1.49E-01	2.46	3.19E-02
160	Q3T0F4	RPS10	40S ribosomal protein S10	1.31	3.17E-01	2.61	2.44E-02
161	E1BAK9	SYVN1	Uncharacterized protein	1.62	1.87E-01	4.50	2.20E-03
162	F1MYW7	HM13	Uncharacterized protein	1.88	9.31E-02	3.12	1.91E-02
163	Q3SZA6	SDCBP	Syndecan binding protein (Syntenin)	1.69	1.75E-01	9.08	2.32E-05
164	F1MBS4	MRPL12	39S ribosomal protein L12, mitochondrial (Fragment)	1.78	1.36E-01	2.40	3.73E-02
165	F1N5H3	PEX3	Peroxisomal biogenesis factor 3	2.05	3.42E-02	2.10	3.69E-02
166	E1BEI2	SPCS2	Uncharacterized protein	2.74	1.25E-02	4.03	2.57E-03

				MII G vs GVO		MII G vs MII B	
#	Accession number	Gene symbol	Discription	Ratio	p-value	Ratio	p-value
167	P17694	NDUFS2	NADH dehydrogenase [ubiquinone] iron-sulfur protein 2, mitochondrial	2.34	3.42E-02	1.65	1.54E-01
168	G3N220		Uncharacterized protein	0.94	4.11E-01	4.05	1.54E-03
169	Q5EAE0	ERGIC3	Endoplasmic reticulum-Golgi intermediate compartment protein 3	1.78	1.21E-01	2.66	2.78E-02
170	A7YY65	MTCH1	MTCH1 protein	1.95	8.07E-02	2.51	2.85E-02
171	Q17QI9	TMEM55B	Transmembrane protein 55B	2.06	3.50E-02	1.76	9.43E-02
172	F1N3U0	HSD11B2	Corticosteroid 11-beta-dehydrogenase isozyme 2	1.68	1.77E-01	2.25	4.41E-02
173	Q2HJD7	HIBADH	3-hydroxyisobutyrate dehydrogenase, mitochondrial	1.40	3.25E-01	3.36	7.97E-03
174	F1MQ37	MYH9	Uncharacterized protein	9.28	2.44E-08	4.94	1.43E-03
175	E1BCH6	ISPD	Isoprenoid synthase domain-containing protein	0.46	4.74E-02	0.26	6.23E-02
176	F2Z4I9	PAM16	Magmas-like protein	0.91	3.84E-01	2.50	1.34E-02
177	Q3ZCD7	TECR	Very-long-chain enoyl-CoA reductase	1.92	1.00E-01	2.77	1.75E-02
178	F1MNS5	ENPP1	Uncharacterized protein	3.08	4.53E-03	3.13	6.57E-03
179	Q3ZBB0	SLC35B2	Solute carrier family 35, member B2	2.27	2.18E-02	2.33	2.28E-02
180	Q05752	NDUFA7	NADH dehydrogenase [ubiquinone] 1 alpha subcomplex subunit 7	1.68	1.53E-01	2.45	2.99E-02
181	Q1LZB5	TOMM40	Mitochondrial import receptor subunit TOM40 homolog	1.78	9.52E-02	6.74	5.08E-05
182	G3N348	WFS1	Uncharacterized protein	2.28	1.57E-02	2.39	3.07E-02
183	P18246	GJA1	Gap junction alpha-1 protein	1.76	9.43E-02	2.47	2.09E-02
184	G5E5V7		Uncharacterized protein (Fragment)	0.88	3.38E-01	9.45	1.44E-06
185	Q58DW0	RPL4	60S ribosomal protein L4	1.29	4.14E-01	2.28	4.98E-02
186	Q2KHZ9	GCDH	Glutaryl-CoA dehydrogenase, mitochondrial	1.26	3.58E-01	9.94	3.66E-05
187	O02691	HSD17B10	3-hydroxyacyl-CoA dehydrogenase type-2	1.25	3.61E-01	2.52	3.12E-02
188	F1MS81	RMND1	Uncharacterized protein	1.81	8.92E-02	5.89	1.12E-04
189	Q17QB3	ASAH1	Acid ceramidase	1.41	2.87E-01	3.70	4.91E-03
190	P48830	ZP3	Zona pellucida sperm-binding protein 3	1.76	1.02E-01	2.76	2.57E-02
191	F1MLS8	KIAA1217	Uncharacterized protein	0.85	3.55E-01	0.17	2.60E-02
192	Q3SZU5	SPCS3	Signal peptidase complex subunit 3	1.62	1.82E-01	2.43	3.89E-02
193	Q0P5D6	RARRES1	Retinoic acid receptor responder (Tazarotene induced) 1	1.78	1.32E-01	2.76	1.84E-02
194	A6QL85	RCC2	RCC2 protein	0.48	8.47E-02	0.20	4.70E-02
195	C3V9V7	TMED2	RNP24	1.69	1.69E-01	2.51	2.82E-02

				MII G vs GVO		MII G vs MII B	
#	Accession number	Gene symbol	Discription	Ratio	p-value	Ratio	p-value
196	E1BH40	MYCBP2	Uncharacterized protein	1.71	1.12E-01	2.91	9.24E-03
197	Q0II48	BCL2L15	Bcl-2-like protein 15	0.30	7.71E-03	0.17	1.10E-02
198	F1N6P2	SURF1	Uncharacterized protein	1.48	2.35E-01	3.13	1.12E-02
199	F6QF86	ALG6	Uncharacterized protein	2.01	8.32E-02	2.37	4.29E-02
200	G8JKY0	RPS8	40S ribosomal protein S8 (Fragment)	2.31	3.59E-02	1.93	7.77E-02
201	F1MYY9	UBASH3A	Uncharacterized protein	0.36	2.25E-02	0.30	7.33E-02
202	P83095	LACTB	Serine beta-lactamase-like protein LACTB, mitochondrial	1.75	1.53E-01	6.08	3.47E-04
203	Q3T003	RPL18A	60S ribosomal protein L18a	1.73	1.22E-01	2.48	2.99E-02
204	Q08D83-2		Isoform 2 of Reticulon-3	1.85	1.00E-01	3.02	1.27E-02
205	P00125	CYC1	Cytochrome c1, heme protein, mitochondrial	1.66	1.59E-01	2.16	4.78E-02
206	Q02375	NDUFS4	NADH dehydrogenase [ubiquinone] iron-sulfur protein 4, mitochondrial	2.16	2.60E-02	4.75	7.53E-04
207	Q05927	NT5E	5-nucleotidase	1.91	1.08E-01	2.28	4.66E-02
208	Q3T0D5	RPL30	60S ribosomal protein L30	1.26	3.96E-01	2.66	3.36E-02
209	Q3SZ90	RPL13A	60S ribosomal protein L13a	1.56	2.06E-01	3.37	1.04E-02
210	Q58DT1	RPL7	60S ribosomal protein L7	1.53	2.17E-01	2.34	3.96E-02
211	P61257	RPL26	60S ribosomal protein L26	2.29	3.95E-02	3.14	1.01E-02
212	F1ML49	KIAA0319L	Uncharacterized protein (Fragment)	2.25	2.02E-02	2.00	7.26E-02
213	Q27991	MYH10	Myosin-10	7.54	3.27E-09	4.47	4.27E-04
214	Q2KJ28	PGS1	CDP-diacylglycerol--glycerol-3-phosphate 3-phosphatidyltransferase, mitochondrial	1.63	1.90E-01	2.88	1.48E-02
215	Q5E9P6	BSCL2	Seipin	1.05	4.85E-01	2.06	3.69E-02
216	E1BA82	UBAP2	Uncharacterized protein	0.76	2.71E-01	2.30	2.38E-02
217	F1N5T9	SERINC5	Uncharacterized protein (Fragment)	2.17	2.30E-02	2.47	3.03E-02
218	E1BKT9	DSP	Uncharacterized protein	0.73	2.32E-01	0.19	2.88E-02
219	A7MB95	CUGBP1	CUGBP1 protein	1.13	4.88E-01	2.51	4.44E-02
220	Q9N285	MTCH2	Mitochondrial carrier homolog 2	1.56	2.07E-01	3.05	1.40E-02
221	Q5EA68	SEC61A1	Protein transport protein Sec61 subunit alpha isoform 1	2.12	4.01E-02	1.77	1.29E-01
222	Q1JQD0	HSD17B1	Hydroxysteroid (17-beta) dehydrogenase 1	2.21	4.55E-02	1.76	1.31E-01

				MII G vs GVO		MII G vs MII B	
**#**	**Accession number**	**Gene symbol**	**Discription**	**Ratio**	**p-value**	**Ratio**	**p-value**
223	Q3ZCA7	GNAI3	Guanine nucleotide binding protein (G protein), alpha inhibiting activity polypeptide 3	1.57	2.20E-01	2.36	3.87E-02
224	A6QNY1	CD320	CD320 antigen	2.50	1.58E-02	2.78	2.45E-02
225	F1N2X1	SLC12A6	Uncharacterized protein	2.08	4.41E-02	1.41	2.42E-01
226	F1MYX5	LCP1	Uncharacterized protein	0.61	3.92E-02	0.49	3.92E-02
227	F1MYS7	EIF4B	Uncharacterized protein	0.42	3.49E-02	0.17	1.47E-02
228	Q5E9Q0	NIP30	NEFA-interacting nuclear protein NIP30	0.48	7.70E-02	0.22	3.67E-02
229	F6RFX1	CHST12	Uncharacterized protein	1.72	1.49E-01	2.85	1.34E-02
230	A4IF97	MYL12B	Myosin regulatory light chain 12B	1.28	3.40E-01	2.44	3.26E-02
231	Q32L96	PHYHIPL	Phytanoyl-CoA hydroxylase interacting protein-like	0.34	1.43E-02	0.30	1.03E-01
232	C4T8B4	CRP	C-reactive protein	0.50	6.42E-03	0.72	6.42E-03
233	Q3T075	STX8	Syntaxin-8	0.49	5.16E-03	1.02	3.53E-01
234	F1N405	RTN4	Reticulon	0.48	6.22E-05	1.23	2.85E-01
235	E1BMJ5	PNPLA6	Uncharacterized protein	0.47	5.74E-03	0.98	4.63E-01
236	F1MUS6	RELN	Reelin (Fragment)	0.47	8.25E-03	0.43	8.25E-03
237	F1MHU9	CNOT1	Uncharacterized protein	0.46	6.62E-03	0.61	6.62E-03
238	Q0VCY6	MBOAT7	Lysophospholipid acyltransferase 7	0.46	2.91E-03	1.58	9.40E-02
239	E1BEV2	MTMR7	Uncharacterized protein	0.45	4.99E-02	0.31	8.46E-02
240	Q0V8E7	STRA6	Stimulated by retinoic acid gene 6 protein homolog	0.44	2.06E-06	1.66	1.59E-01
241	Q3T0V2	ACY1	Aminoacylase 1	0.44	3.64E-02	0.24	4.76E-02
242	F1MCP8	CAPRIN2	Uncharacterized protein (Fragment)	0.43	4.81E-05	1.02	4.36E-01
243	F1MT25	HDLBP	Uncharacterized protein	0.43	9.98E-04	0.73	4.50E-01
244	E1BPY5	MFSD8	Uncharacterized protein	0.43	9.79E-04	0.45	9.79E-04
245	F1N3H0	DCAF8	Uncharacterized protein	0.43	4.81E-04	0.99	3.25E-01
246	F1MBV6	TPD52	Uncharacterized protein	0.42	3.81E-02	0.29	9.26E-02
247	E1BNN6	NLRP1	Uncharacterized protein	0.42	1.67E-03	0.70	1.67E-03
248	F1MVS4	NOL3	Uncharacterized protein	0.37	3.45E-02	0.41	8.42E-04
249	E1BPE9	KHDC3L	Uncharacterized protein	0.41	5.44E-08	1.59	2.06E-01
250	Q9TS87	TAGLN	Transgelin	0.40	4.55E-02	0.40	1.82E-01

				MII G vs GVO		MII G vs MII B	
**#**	**Accession number**	**Gene symbol**	**Discription**	**Ratio**	**p-value**	**Ratio**	**p-value**
251	Q3T0V7	EDF1	Endothelial differentiation-related factor 1	0.38	2.40E-02	0.35	1.16E-01
252	E1BCN1	LPIN2	Uncharacterized protein	0.38	5.46E-07	0.72	3.72E-01
253	F1MNG7	LZTFL1	Leucine zipper transcription factor-like protein 1 (Fragment)	0.37	3.28E-02	0.24	6.52E-02
254	Q3T0S5	ALDOB	Fructose-bisphosphate aldolase B	0.37	2.48E-02	0.27	6.43E-02
255	F1N214	ENAH	Uncharacterized protein	0.37	1.52E-05	0.90	5.00E-01
256	Q58DT6	ARHGDIA	Rho GDP dissociation inhibitor (GDI) alpha	0.35	3.13E-02	0.17	3.05E-02
257	Q1PSA0	ZAR1	Uncharacterized protein	0.34	7.40E-08	1.22	4.22E-01
258	Q17Q89	PFDN6	Prefoldin subunit 6	0.31	1.12E-02	0.29	7.89E-02
259	Q2KJJ1	SLC25A17	Solute carrier family 25 (Mitochondrial carrier; peroxisomal membrane protein, 34kDa), member 17	0.28	1.79E-05	0.21	1.79E-05
260	P37359	MT3	Metallothionein-3	0.25	1.40E-03	0.19	2.69E-02
261	Q5KR47-2		Isoform 2 of Tropomyosin alpha-3 chain	0.20	2.58E-11	0.87	4.35E-01
262	G3N0V0		Uncharacterized protein (Fragment)	0.20	3.44E-03	0.13	1.60E-02
263	P81282-4		Isoform V3 of Versican core protein	0.16	2.19E-04	1.21	2.95E-01
264	Q3T0C8	PDLIM2	PDZ and LIM domain protein 2	0.14	5.83E-14	0.73	4.61E-01
265	F1MSH0	CPEB1	Uncharacterized protein (Fragment)	0.11	1.15E-30	0.36	1.15E-30

Table S4. GO categorization clustering of differentially expressed proteins.

Cluster 1

Category	Term	Count	%	P Value	Genes	List Total	Pop Hits	Pop Total	Fold Enrichment	Bonferroni	Benjamini	FDR
GOTERM_BP_FAT	GO:0022900~electron transport chain	7	12.5	1.99E-06	Q02375, P00125, Q02380, Q05752, Q02827, P52505, P23934	40	87	8785	17.67098	5.15E-04	5.15E-04	0.002599
GOTERM_BP_FAT	GO:0055114~oxidation reduction	11	19.64286	5.76E-05	Q02375, P00125, Q2KHZ9, Q02380, Q05752, O02691, Q02827, P52505, Q3ZCD7, Q2HJD7, P23934	40	513	8785	4.709308	0.014817	0.007436	0.075262
GOTERM_BP_FAT	GO:0006120~mitochondrial electron transport, NADH to ubiquinone	3	5.357143	2.85E-04	Q05752, Q02827, P52505	40	6	8785	109.8125	0.071131	0.024296	0.371472
GOTERM_BP_FAT	GO:0006091~generation of precursor metabolites and energy	7	12.5	3.01E-04	Q02375, P00125, Q02380, Q05752, Q02827, P52505,	40	211	8785	7.286137	0.074958	0.019291	0.392215

					P23934							
GOTERM_BP_FAT	GO:0045333~cellular respiration	4	7.142857	0.001219	Q02375, Q05752, Q02827, P52505	40	48	8785	18.30208	0.270821	0.061213	1.580365
GOTERM_BP_FAT	GO:0006412~translation	7	12.5	0.001412	Q58DW0, Q3T003, Q3SZ90, P82916, Q58DT1, Q3T0D5, Q0II26	40	283	8785	5.43242	0.306423	0.05916	1.828538
GOTERM_BP_FAT	GO:0042775~mitochondrial ATP synthesis coupled electron transport	3	5.357143	0.00169	Q05752, Q02827, P52505	40	14	8785	47.0625	0.354683	0.060656	2.184988
GOTERM_BP_FAT	GO:0042773~ATP synthesis coupled electron transport	3	5.357143	0.00250	Q05752, Q02827, P52505	40	17	8785	38.75735	0.477639	0.077967	3.222314
GOTERM_BP_FAT	GO:0015980~energy derivation by oxidation of organic compounds	4	7.142857	0.00346	Q02375, Q05752, Q02827, P52505	40	69	8785	12.73188	0.592711	0.094985	4.42934
GOTERM_BP_FAT	GO:0022904~respiratory electron transport chain	3	5.357143	0.00382	Q05752, Q02827, P52505	40	21	8785	31.375	0.629253	0.09446	4.881381
GOTERM_BP_FAT	GO:0007264~small GTPase mediated signal transduction	5	8.928571	0.00657	Q17R06, Q148J4, A5D977, P11023, Q3SZA6	40	170	8785	6.459559	0.818562	0.143728	8.248668
GOTERM_BP_FAT	GO:0006119~oxidative phosphorylation	3	5.357143	0.02891	Q05752, Q02827, P52505	40	60	8785	10.98125	0.999499	0.469133	31.83699

GOTERM_BP_FAT	GO:0007005~mitochondrion organization	3	5.357143	0.03738	Q02375, Q1LZB5, P11023	40	69	8785	9.548913	0.999948	0.531873	39.20567
GOTERM_BP_FAT	GO:0008610~lipid biosynthetic process	4	7.142857	0.03855	Q58DH7, P52505, Q3ZCD7, Q2KJ28	40	169	8785	5.198225	0.999962	0.516819	40.16661
GOTERM_BP_FAT	GO:0070271~protein complex biogenesis	4	7.142857	0.045116	Q02375, A0JNQ6, Q2T9S0, P18246	40	180	8785	4.880556	0.999994	0.549376	45.28699
GOTERM_BP_FAT	GO:0006461~protein complex assembly	4	7.142857	0.04512	Q02375, A0JNQ6, Q2T9S0, P18246	40	180	8785	4.880556	0.999994	0.549376	45.28699

Cluster 2

Category	Term	Count	%	P Value	Genes	List Total	Pop Hits	Pop Total	Fold Enrichment	Bonferroni	Benjamini	FDR
GOTERM_BP_FAT	GO:0008610~lipid biosynthetic process	2	18.18182	0.07477	P80177, Q0VCY6	5	169	8785	20.7929	0.992523	0.992523	54.47378

Cluster 3

Category	Term	Count	%	P Value	Genes	List Total	Pop Hits	Pop Total	Fold Enrichment	Bonferroni	Benjamini	FDR
GOTERM_BP_FAT	GO:0018130~heterocycle biosynthetic process	4	19.04762	3.41E-05	P56658, Q3SZ18, Q58DK5, Q3ZBD3	17	36	8785	57.4183	0.012883	0.012883	0.047228
GOTERM_BP_FAT	GO:0046148~pigment biosynthetic process	3	14.28571	5.80E-04	P56658, A5PK65, Q58DK5	17	20	8785	77.51471	0.19777	0.104327	0.799586
GOTERM_BP_FAT	GO:0042440~pigment metabolic process	3	14.28571	9.11E-04	P56658, A5PK65, Q58DK5	17	25	8785	62.01176	0.292594	0.108976	1.253155
GOTERM_BP_FAT	GO:0046100~hypoxanthine metabolic process	2	9.52381	0.005455	P56658, Q3SZ18	17	3	8785	344.5098	0.874869	0.405241	7.292311
GOTERM_BP_FAT	GO:0043101~purine salvage	2	9.52381	0.007267	P56658, Q3SZ18	17	4	8785	258.3824	0.937423	0.425509	9.603453
GOTERM_BP_FAT	GO:0044271~nitrogen compound biosynthetic process	4	19.04762	0.008965	P56658, Q3SZ18, Q58DK5, Q3ZBD3	17	243	8785	8.506415	0.96736	0.434684	11.72173
GOTERM_BP_FAT	GO:0043094~cellular metabolic compound salvage	2	9.52381	0.010881	P56658, Q3SZ18	17	6	8785	172.2549	0.984353	0.447846	14.05507
GOTERM_BP_FAT	GO:0009163~nucleoside biosynthetic process	2	9.52381	0.012684	P56658, Q3SZ18	17	7	8785	147.6471	0.992177	0.454655	16.19834
GOTERM_BP_FAT	GO:0042455~ribonucleoside biosynthetic process	2	9.52381	0.012684	P56658, Q3SZ18	17	7	8785	147.6471	0.992177	0.454655	16.19834
GOTERM_BP_FAT	GO:0046129~purine ribonucleoside biosynthetic process	2	9.52381	0.012684	P56658, Q3SZ18	17	7	8785	147.6471	0.992177	0.454655	16.19834
GOTERM_BP_FAT	GO:0042451~purine	2	9.52381	0.012684	P56658,	17	7	8785	147.6471	0.992177	0.454655	16.19834

	nucleoside biosynthetic process				Q3SZ18							
GOTERM_BP_FAT	GO:0051289~protein homotetramerization	2	9.52381	0.01628	Q3SZ18, Q3ZBD3	17	9	8785	114.8366	0.998045	0.499944	20.32657
GOTERM_BP_FAT	GO:0006144~purine base metabolic process	2	9.52381	0.019864	P56658, Q3SZ18	17	11	8785	93.95722	0.999512	0.533467	24.2523
GOTERM_BP_FAT	GO:0042278~purine nucleoside metabolic process	2	9.52381	0.021651	P56658, Q3SZ18	17	12	8785	86.12745	0.999756	0.530538	26.14234
GOTERM_BP_FAT	GO:0046128~purine ribonucleoside metabolic process	2	9.52381	0.021651	P56658, Q3SZ18	17	12	8785	86.12745	0.999756	0.530538	26.14234
GOTERM_BP_FAT	GO:0009112~nucleobase metabolic process	2	9.52381	0.025217	P56658, Q3SZ18	17	14	8785	73.82353	0.999939	0.554595	29.78273
GOTERM_BP_FAT	GO:0051262~protein tetramerization	2	9.52381	0.032311	Q3SZ18, Q3ZBD3	17	18	8785	57.4183	0.999996	0.617138	36.53625
GOTERM_BP_FAT	GO:0070271~protein complex biogenesis	3	14.28571	0.041501	Q17Q89, Q3SZ18, Q3ZBD3	17	180	8785	8.612745	1	0.683519	44.38936
GOTERM_BP_FAT	GO:0006461~protein complex assembly	3	14.28571	0.041501	Q17Q89, Q3SZ18, Q3ZBD3	17	180	8785	8.612745	1	0.683519	44.38936
GOTERM_BP_FAT	GO:0009119~ribonucleoside metabolic process	2	9.52381	0.042862	P56658, Q3SZ18	17	24	8785	43.06373	1	0.670377	45.47311

Cluster 4

Category	Term	Count	%	P Value	Genes	List Total	Pop Hits	Pop Total	Fold Enrichment	Bonferroni	Benjamini	FDR
GOTERM_BP_FAT	GO:0007059~chromosome segregation	3	10.71429	0.001558	Q2YDJ0, Q08DR9, Q3SZY3	19	29	8785	47.83122	0.212242	0.212242	1.851031
GOTERM_BP_FAT	GO:0007017~microtubule-based process	4	14.28571	0.002344	Q2YDJ0, Q08DR9, Q3MHR3, A6QPE8	19	133	8785	13.90582	0.301642	0.164322	2.772618
GOTERM_BP_FAT	GO:0040001~establishment of mitotic spindle localization	2	7.142857	0.006135	Q2YDJ0, Q08DR9	19	3	8785	308.2456	0.609973	0.269369	7.108575
GOTERM_BP_FAT	GO:0051653~spindle localization	2	7.142857	0.008172	Q2YDJ0, Q08DR9	19	4	8785	231.1842	0.715054	0.269382	9.364486
GOTERM_BP_FAT	GO:0051293~establishment of spindle localization	2	7.142857	0.008172	Q2YDJ0, Q08DR9	19	4	8785	231.1842	0.715054	0.269382	9.364486
GOTERM_BP_FAT	GO:0007067~mitosis	3	10.71429	0.011129	Q2YDJ0, Q08DR9, Q3SZY3	19	79	8785	17.55829	0.819541	0.289971	12.54965
GOTERM_BP_FAT	GO:0000280~nuclear division	3	10.71429	0.011129	Q2YDJ0, Q08DR9, Q3SZY3	19	79	8785	17.55829	0.819541	0.289971	12.54965
GOTERM_BP_FAT	GO:0000087~M phase of mitotic cell cycle	3	10.71429	0.012233	Q2YDJ0, Q08DR9, Q3SZY3	19	83	8785	16.71211	0.847887	0.269375	13.71218
GOTERM_BP_FAT	GO:0048285~organelle fission	3	10.71429	0.012516	Q2YDJ0, Q08DR9, Q3SZY3	19	84	8785	16.51316	0.854416	0.240645	14.00818
GOTERM_BP_FAT	GO:0051276~chromosome organization	4	14.28571	0.013252	Q2YDJ0, Q08DR9, Q3SZY3, Q24K09	19	248	8785	7.457555	0.870113	0.225189	14.77309

GOTERM_BP_FAT	GO:0000070~mitotic sister chromatid segregation	2	7.142857	0.020312	Q2YDJ0, Q08DR9	19	10	8785	92.47368	0.956705	0.294506	21.79951
GOTERM_BP_FAT	GO:0000819~sister chromatid segregation	2	7.142857	0.020312	Q2YDJ0, Q08DR9	19	10	8785	92.47368	0.956705	0.294506	21.79951
GOTERM_BP_FAT	GO:0000279~M phase	3	10.71429	0.021216	Q2YDJ0, Q08DR9, Q3SZY3	19	111	8785	12.49644	0.962409	0.279708	22.65991
GOTERM_BP_FAT	GO:0000278~mitotic cell cycle	3	10.71429	0.021938	Q2YDJ0, Q08DR9, Q3SZY3	19	113	8785	12.27527	0.966422	0.265478	23.34076
GOTERM_BP_FAT	GO:0022403~cell cycle phase	3	10.71429	0.032225	Q2YDJ0, Q08DR9, Q3SZY3	19	139	8785	9.979175	0.99334	0.341399	32.46327
GOTERM_BP_FAT	GO:0051656~establishment of organelle localization	2	7.142857	0.038259	Q2YDJ0, Q08DR9	19	19	8785	48.67036	0.997442	0.368159	37.33951

Cluster 5

Category	Term	Count	%	P Value	Genes	List Total	Pop Hits	Pop Total	Fold Enrichment	Bonferroni	Benjamini	FDR
GOTERM_BP_FAT	GO:0055114~oxidation reduction	7	19.44444	0.002099	P17694, Q85BD6, P21839, P48644, Q6B4J2, Q1JQD0, Q02367	25	513	8785	4.794932	0.372799	0.372799	2.64316
GOTERM_BP_FAT	GO:0055085~transmembrane transport	6	16.66667	0.00351	Q3ZBB0, Q4F894, Q5EA68, Q2KHX4, P12234, A7YY65	25	389	8785	5.420051	0.541859	0.323139	4.383358
GOTERM_BP_FAT	GO:0022900~electron transport chain	3	8.333333	0.023234	P17694, Q85BD6, Q02367	25	87	8785	12.11724	0.994587	0.824413	25.89471
GOTERM_BP_FAT	GO:0006487~protein amino acid N-linked glycosylation	2	5.555556	0.029661	Q0P5D9, Q3SZI6	25	11	8785	63.89091	0.99875	0.811956	31.87555

Table S5. List of primer sequences for Real-time Quantitative RT-PCR analysis.

Gene	Primer name	Sequences(5'-3')	Fragments size (bp)
**MYH10**	Forward	ATGCCTCCACACATCTACGC	201
	Reverse	CCGTTCAAGTTCCCCAGGAAT	
**MYH9**	Forward	TTTTGTGCACGGGTGAGTCT	247
	Reverse	GCTCCCACGATGTAGCCATT	
**KIF20A**	Forward	TGGAGTCACCAACTCAGGGA	238
	Reverse	GGACAGCTCCTCCTCTTGGA	
**KIF2C**	Forward	CAGTTTACGCGGCGTTAGGA	152
	Reverse	AATTATGCCATTACTGCGCTGG	
**DYNLL2**	Forward	ACTCGCCTCTGTGAAGTGTC	206
	Reverse	ATTTCGGCCCACGATACAGT	
**β-actin**	Forward	ACCGCAAATGCTTCTAGG	200
	Reverse	ATCCAACCGACTGCTGTC	
